# Supplementary material for: Diversity and evolution of the vertebrate chemoreceptor gene repertoire
Source: Nat Commun. 2024 Feb 15;15:1421. doi: 10.1038/s41467-024-45500-y (PMC10869828; doi:10.1038/s41467-024-45500-y)

This PDF file includes:

Supplementary Tables 1-7
Supplementary Figures 1-56

Other supplementary materials for this manuscript include the following:

Supplementary Data 1

Supplementary Table 1 | Summary statistics of the number of genome assemblies used for this study. Note that the total number of genomes analyzed for the BUSCO80 dataset was 1,531 genomes, which corresponds to the 1,527 genomes with >80% complete BUSCO genes plus the five agnathan genomes (Supplementary Fig. 2) minus the genome of *S. occidentalis* (Supplementary Fig. 3). For the BUSCO90 dataset, the total number was 1,180 genomes, which corresponds to the 1,176 genomes with >90% complete BUSCO genes plus the five agnathan genomes minus the genome of *S. occidentalis*. Nb of species: number of genome assemblies downloaded from NCBI; Nb of species in tree: number of species for which phylogenetic information was available; Nb of species >80% BUSCO: number of species that passed the >80% complete BUSCO filter; Nb of species >80% BUSCO and present in the tree: number of species that passed the >80% complete BUSCO filter and for which phylogenetic information was available; Nb of species >90% BUSCO: number of species that passed the >90% complete BUSCO filter; Nb of species >90% BUSCO and present in the tree: number of species that passed the >90% complete BUSCO filter and for which phylogenetic information was available. BUSCO raw results be found in Supplementary Data 1. Source data are provided as a Source Data file.

Class	Nb of species	Nb of species in tree	Nb of species >80% BUSCO	Nb of species >80% BUSCO and present in the tree	Nb of species >90% BUSCO	Nb of species >90% BUSCO and present in the tree
Agnatha	5	5	0	0	0	0
Chondrichtyes	14	14	10	10	7	7
Actinopterygii	900	764	483	449	369	345
Coelacanth	1	1	1	1	0	0
Dinoi	2	2	1	1	1	1
Amphibia	32	32	20	20	13	13
Mammals	555	501	440	392	387	343
Lepidosauria	66	64	54	53	45	44
Testudines	27	27	26	26	25	25
Crocodilia	4	4	4	4	3	3
Aves	604	574	488	467	326	312
Total	2210	1988	1527	1423	1176	1093

Supplementary Table 2 | Correlations between the number of complete genes across chemoreceptor gene families taking genome size into account. The output of multiple two-sided pGLS models for comparisons between the complete gene number in each chemoreceptor family, with interactions between predictors (gene family * genome size) or without (gene family + genome size), is reported for mammals, birds and ray-finned fishes. All *P*-values between the number of complete genes across chemoreceptor families remained significant when taking the genome size into account, except between the number of complete *OR* and complete *TAAR* genes of birds (in the BUSCO80 and the BUSCO90 datasets). In some cases, the interaction between gene families and the genome size was significant, but the model R^2 did not increase, or increased only slightly compared to the additive model or compared to the simple models that did not take the genome size into account (Supplementary Fig. 6). Source data are provided as a Source Data file.

Species	Response ~ pred1+pred2	Dataset	Adjusted R²	pred1 p-value	pred2 p-value	pred interaction p-value	pGLS model p-value
Mammalia	TAAR ~ OR + Genome	BUSCO80	0.1804	<2e-16	0.9533	/	<2e-16
Mammalia	TAAR ~ OR * Genome size	BUSCO80	0.1788	0.0009764	0.18	0.118	<2e-16
Actinoptery	TAAR ~ OR + Genome	BUSCO80	0.3937	<2e-16	0.03317	/	<2e-16
Actinoptery	TAAR ~ OR * Genome size	BUSCO80	0.4231	1.512e-06	0.133	0.003	<2e-16
Aves	TAAR ~ OR + Genome	BUSCO80	0.008198	0.075	0.77	/	0.1457
Aves	TAAR ~ OR * Genome size	BUSCO80	0.03862	0.0001373	0.0741752	3.599e-05	8.674e-05
Mammalia	V1R ~ OR + Genome size	BUSCO80	0.09941	1.319e-10	0.63754	/	5.278e-10
Mammalia	V1R ~ OR * Genome size	BUSCO80	0.1113	0.299821	0.042383	0.115	1.418e-10
Actinoptery	V1R ~ OR + Genome size	BUSCO80	0.1311	2.700e-13	0.06878	/	8.971e-15
Actinoptery	V1R ~ OR * Genome size	BUSCO80	0.2313	0.01018	1.406e-07	1.619e-13	<2e-16
Mammalia	V2R ~ OR + Genome size	BUSCO80	0.02262	0.0014	0.845	/	0.004306
Mammalia	V2R ~ OR * Genome size	BUSCO80	0.1788	0.0009764	0.1790147	0.593	<2e-16
Actinoptery	V2R ~ OR + Genome size	BUSCO80	0.4405	<2e-16	0.6578	/	<2e-16
Actinoptery	V2R ~ OR * Genome size	BUSCO80	0.4393	<2e-16	0.7956	0.977	<2e-16
Mammalia	V1R ~ TAAR + Genome	BUSCO80	0.03065	0.0004048	0.2484310	/	0.0008663
Mammalia	V1R ~ TAAR * Genome	BUSCO80	0.05201	0.022262	0.053932	0.001913	2.83e-05
Actinoptery	V1R ~ TAAR + Genome	BUSCO80	0.1107	5.462e-11	0.03456	/	1.582e-12
Actinoptery	V1R ~ TAAR * Genome	BUSCO80	0.1668	0.6915	0.1262	5.822e-07	<2e-16
Mammalia	V2R ~ TAAR + Genome	BUSCO80	0.03149	0.0002006	0.5250194	/	0.0007312
Mammalia	V2R ~ TAAR * Genome	BUSCO80	0.04901	0.052041	0.046559	0.004808	5.093e-05
Actinoptery	V2R ~ TAAR + Genome	BUSCO80	0.2975	<2e-16	0.6146	/	<2e-16
Actinoptery	V2R ~ TAAR * Genome	BUSCO80	0.3047	<2e-16	0.06051	0.01826	<2e-16
Mammalia	V1R ~ V2R + Genome size	BUSCO80	0.4199	<2e-16	0.33656	/	<2e-16
Mammalia	V1R ~ V2R * Genome size	BUSCO80	0.42	0.12913	0.48893	0.30542	<2e-16
Actinoptery	V1R ~ V2R + Genome size	BUSCO80	0.1079	1.110e-10	0.008812	/	3.222e-12
Actinoptery	V1R ~ V2R * Genome size	BUSCO80	0.1895	0.007245	0.003780	1.017e-10	<2e-16
Actinoptery	T1R ~ T2R + Genome size	BUSCO80	0.05012	0.01705	5.067e-05	/	3.863e-06
Actinoptery	T1R ~ T2R * Genome size	BUSCO80	0.04806	0.32310	0.05152	0.87148	1.593e-05
Mammalia	OLR ~ TR + Genome size	BUSCO80	0.1535	4.441e-16	0.8915	/	3.086e-15
Mammalia	OLR ~ TR * Genome size	BUSCO80	0.1519	0.02931	0.62726	0.63432	1.895e-14
Actinoptery	OLR ~ TR + Genome size	BUSCO80	0.1919	<2e-16	0.002527	/	<2e-16
Actinoptery	OLR ~ TR * Genome size	BUSCO80	0.1917	9.825e-09	0.008183	0.320127	<2e-16
Aves	OLR ~ TR + Genome size	BUSCO80	0.1586	4.441e-16	0.1327	/	<2e-16
Aves	OLR ~ TR * Genome size	BUSCO80	0.1735	8.581e-06	0.007953	0.002957	<2e-16
Mammalia	TAAR ~ OR + Genome	BUSCO90	0.1557	3.02e-14	0.9586	/	1.182e-13
Mammalia	TAAR ~ OR * Genome size	BUSCO90	0.1602	0.001406	0.125979	0.082593	1.918e-13
Actinoptery	TAAR ~ OR + Genome	BUSCO90	0.3621	<2e-16	0.1084	/	<2e-16
Actinoptery	TAAR ~ OR * Genome size	BUSCO90	0.3802	8.839e-05	0.063678	0.001495	<2e-16
Aves	TAAR ~ OR + Genome	BUSCO90	-0.003152	0.3730	0.3477	/	0.603
Aves	TAAR ~ OR * Genome size	BUSCO90	0.02891	0.0010067	0.1064870	0.0008717	0.006958
Mammalia	V1R ~ OR + Genome size	BUSCO90	0.06024	8.169e-06	0.2390	/	9.551e-06
Mammalia	V1R ~ OR * Genome size	BUSCO90	0.06401	0.54712	0.34203	0.12676	1.243e-05
Actinoptery	V1R ~ OR + Genome size	BUSCO90	0.1001	5.580e-09	0.2787	/	5.413e-09
Actinoptery	V1R ~ OR * Genome size	BUSCO90	0.1692	0.02081	6.008e-09	8.781e-09	2.673e-14
Mammalia	V2R ~ OR + Genome size	BUSCO90	0.02183	0.01346	0.15062	/	0.008664
Mammalia	V2R ~ OR * Genome size	BUSCO90	0.02065	0.7903	0.9080	0.4448	0.01793
Actinoptery	V2R ~ OR + Genome size	BUSCO90	0.3616	<2e-16	0.3037	/	<2e-16
Actinoptery	V2R ~ OR * Genome size	BUSCO90	0.3598	5.048e-12	0.4618	0.8290	<2e-16
Mammalia	V1R ~ TAAR + Genome	BUSCO90	0.03311	0.001356	0.089331	/	0.001204
Mammalia	V1R ~ TAAR * Genome	BUSCO90	0.06111	0.0087516	0.0464930	0.0009118	2.062e-05
Actinoptery	V1R ~ TAAR + Genome	BUSCO90	0.08733	9.372e-08	0.1811	/	6.035e-08
Actinoptery	V1R ~ TAAR * Genome	BUSCO90	0.3598	5.048e-12	0.4618	0.8290	<2e-16
Mammalia	V2R ~ TAAR + Genome	BUSCO90	0.04345	0.0002121	0.0923738	/	0.0001938
Mammalia	V2R ~ TAAR * Genome	BUSCO90	0.06254	0.045240	0.121786	0.004944	1.608e-05
Actinoptery	V2R ~ TAAR + Genome	BUSCO90	0.2566	<2e-16	0.6521	/	<2e-16
Actinoptery	V2R ~ TAAR * Genome	BUSCO90	0.2606	1.554e-14	0.47423	0.08965	<2e-16
Mammalia	V1R ~ V2R + Genome size	BUSCO90	0.4571	<2e-16	0.364350	/	<2e-16
Mammalia	V1R ~ V2R * Genome size	BUSCO90	0.457	0.10848	0.50291	0.33257	<2e-16
Actinoptery	V1R ~ V2R + Genome size	BUSCO90	0.08018	2.897e-07	0.06637	/	2.29e-07
Actinoptery	V1R ~ V2R * Genome size	BUSCO90	0.1791	0.005514	0.002108	6.851e-10	3.524e-15
Actinoptery	T1R ~ T2R + Genome size	BUSCO90	0.02473	0.083513	0.007731	/	0.005096
Actinoptery	T1R ~ T2R * Genome size	BUSCO90	0.02333	0.17700	0.04967	0.44883	0.01144
Mammalia	OLR ~ TR + Genome size	BUSCO90	0.1238	1.92e-11	0.7441	/	6.39e-11
Mammalia	OLR ~ TR * Genome size	BUSCO90	0.1213	0.1560	0.8596	0.9583	3.688e-10
Actinoptery	OLR ~ TR + Genome size	BUSCO90	0.1311	1.644e-10	0.03801	/	1.362e-11
Actinoptery	OLR ~ TR * Genome size	BUSCO90	0.1307	7.7e-06	0.04501	0.35867	5.368e-11
Aves	OLR ~ TR + Genome size	BUSCO90	0.1454	1.378e-10	0.2144	/	8.282e-12
Aves	OLR ~ TR * Genome size	BUSCO90	0.166	4.574e-05	0.005403	0.003057	7.346e-13

Supplementary Table 3 | BayesTraits analysis of *T1R* gene loss in mammals. Three models were tested for each T1R gene (*T1R1*, *T1R2*, *T1R3*). Model 1 corresponds to an independent evolution of the gene and of the diet preference, model 2 corresponds to a dependent evolution of the gene and the diet preference, and model 3 corresponds to an evolution of the gene depending on diet preference, but not the other way around. In the three models tested, the transition rate of *T1R2* from 0 to 1 (from pseudogene to functional gene) was set to 0 (alpha1 for the model1 and q13 + q24 for model2 and model3). In model3, transition rates from carnivorous to herbivorous/omnivorous were forced to be equal, irrespective of whether *T1R2* was functional or not, just as the transition rates from herbivorous/omnivorous to carnivorous (q12 = q34 , q21 = q43). The log-likelihood (10,000 runs of the maximum likelihood algorithm) given by BayesTraits for the three models, as well as all the parameters values, are displayed. Log-likelihood ratio test to compare Model 2 and Model 3 to Model 1 are also displayed. Model 2 and Model 3 were not significantly better than the Model 1 for *T1R1* and *T1R3*. Model 2 and Model 3 were both significantly better than model 1 for *T1R2*, with the transition rate from a functional *T1R2* gene to a non-functional *T1R2* gene being higher in carnivores than in herbivores/omnivores (q42 > q31).

Model 1 - Independent evolution of T1R1 and diet preference

Lh	alpha1	beta1	alpha2	beta2	Root - P(0,0)	Root - P(0,1)	Root - P(1,0)	Root - P(1,1)
-202.370602	0.000000	0.003313	0.003255	0.008835	0.000000	0.000000	0.143021	0.856979

Model 2 - Dependent evolution of T1R1 and diet preference

Lh	q12	q13	q21	q24	q31	q34	q42	q43	Root - P(0,0)	Root - P(0,1)	Root - P(1,0)	Root - P(1,1)
-200.9068 32	0.016352	0.000000	0.000000	0.000000	0.003552	0.004905	0.002673	0.013110	0.000000	0.000000	0.385670	0.614330

Model 3 - Evolution of T1R1 dependent on the diet preference

Lh	q12	q13	q21	q24	q31	q34	q42	q43	Root - P(0,0)	Root - P(0,1)	Root - P(1,0)	Root - P(1,1)
-202.2167 33	0.003345	0.000000	0.008886	0.000000	0.002997	0.003345	0.004044	0.008886	0.000000	0.000000	0.182840	0.817160

**Model 2 vs Model 1**	Log BF = 2(-200.906832- -202.370602) = 2.92754	p-value (3df) = 0.4029325
**Model 3 vs Model 1**	Log BF = 2(-202.216733- -202.370602) = 0.307738	p-value (1df) = 0.5790716

Model 1 - Independent evolution of T1R2 and diet preference

**Lh**	**alpha1**	**beta1**	**alpha2**	**beta2**	**Root - P(0,0)**	**Root - P(0,1)**	**Root - P(1,0)**	**Root - P(1,1)**
-207.910576	0.000000	0.003238	0.003255	0.008835	0.000000	0.000000	0.143013	0.856987

Model 2 - Dependent evolution of T1R2 and diet preference

**Lh**	**q12**	**q13**	**q21**	**q24**	**q31**	**q34**	**q42**	**q43**	**Root - P(0,0)**	**Root - P(0,1)**	**Root - P(1,0)**	**Root - P(1,1)**
-199.2943 51	0.031542	0.000000	0.003249	0.000000	0.001902	0.003470	0.006493	0.011544	0.000000	0.000000	0.382606	0.617394

Model 3 - Evolution of T1R2 dependent on the diet preference

Lh	q12	q13	q21	q24	q31	q34	q42	q43	Root - P(0,0)	Root - P(0,1)	Root - P(1,0)	Root - P(1,1)
-202.9071 38	0.003719	0.000000	0.009015	0.000000	0.001549	0.003719	0.007261	0.009015	0.000000	0.000000	0.417552	0.582448

**Model 2 vs Model 1**	Log BF = 2(-199.294351- -207.910576) = 17.23245	p-value (3df) = 0.0006330509
**Model 3 vs Model 1**	Log BF = 2(-202.907138- -207.910576) = 10.00688	p-value (1df) = 0.001559565

Model 1 - Independent evolution of T1R3 and diet preference

Lh	alpha1	beta1	alpha2	beta2	Root - P(0,0)	Root - P(0,1)	Root - P(1,0)	Root - P(1,1)
-208.202037	0.000000	0.002803	0.003255	0.008835	0.000000	0.000000	0.143019	0.856981

Model 2 - Dependent evolution of T1R3 and diet preference

Lh	q12	q13	q21	q24	q31	q34	q42	q43	Root - P(0,0)	Root - P(0,1)	Root - P(1,0)	Root - P(1,1)
-204.7272 61	0.042161	0.000000	0.000626	0.000000	0.004006	0.003047	0.000768	0.009596	0.000000	0.000000	0.080975	0.919025

Model 3 - Evolution of T1R3 dependent on the diet preference

Lh	q12	q13	q21	q24	q31	q34	q42	q43	Root - P(0,0)	Root - P(0,1)	Root - P(1,0)	Root - P(1,1)
-207.5990 65	0.003192	0.000000	0.008762	0.000000	0.003339	0.003192	0.001782	0.008762	0.000000	0.000000	0.105178	0.894822

**Model 2 vs Model 1**	Log BF = 2(-204.727261 - -208.202037) = 6.949552	p-value (3df) = 0.07352322
**Model 3 vs Model 1**	Log BF = 2(-207.599065 - -208.202037) = 1.205944	p-value (1df) = 0.2721369

Supplementary Table 4 | Contingency tables for *T1R* gene losses in mammals. These contingency tables were used to infer whether or not the number of *T1R* gene losses (for *T1R1*, *T1R2* and T1R3) is significantly different between diet categories. Theoretical examples are shown in the Supplementary Fig. 28. Both one-sided Chi-squared test and one-sided Fisher's exact test *P*-values are reported. Significant correlations were only found for *T1R2*, with more loss events in carnivores than in herbivores and omnivores. This stays true when removing cetacean and pinnipeds from the analysis. *T1R* genes loss event numbers were based on shared LoF (loss-of-function) mutations.

	T1R1 intact	T1R1 loss event
Carnivore	138	8
Herbivore	328	9
Omnivore	131	5

Chi-squared test p.value = 0.3085
Fisher's Exact Test p.value = 0.3023

	T1R2 intact	T1R2 loss event
Carnivore	129	17
Herbivore	347	6
Omnivore	145	2

Chi-squared test p.value = 2.183e-07
Fisher's Exact Test p.value = 2.9e-06

Removing Pinnipeds and Cetacean clades :

	T1R2 intact	T1R2 loss event
Carnivore	127	13
Herbivore	347	6
Omnivore	145	2

Chi-squared test p.value = 3.725e-05
Fisher's Exact Test p.value = 0.0001818

	T1R3 intact	T1R3 loss event
Carnivore	176	6
Herbivore	346	9
Omnivore	141	6

Chi-squared test p.value = 0.6446
Fisher's Exact Test p.value = 0.558

Supplementary Table 5 | Blastp results for cartilaginous fish *T2R* genes against the NCBI nr database. Results of blastp results are shown with or without cartilaginous fish sequences. The best matches of all these *T2R* genes are *T2R* genes found in *S. canicula* and *P. pectinata* (annotated in their reference genome assemblies). When removing Chondrichthyes sequences (taxid:7777) from the nr database, all the best matches were *T2R* genes from the coelacanth.

Blastp against NCBI NR database

Query	Match accession	Match description	Query cover (%)	E-value	Perc Identity (%)
Ginglymostoma_cirratum---JAHRHZ010000132.1-202980-203993---1_exons---phobius-tmhmm	XP_038632475.1	taste receptor type 2 member 13-like [Scyliorhinus canicula]	100	4,00E-109	55.62
Stegostoma_fasciatum---CM039255.1-41488116-41489120---1_exons---phobius-tmhmm	XP_038632475.1	taste receptor type 2 member 13-like [Scyliorhinus canicula]	100	3,00E-105	55.03
Rhincodon_typus---CM038826.1-278077-279270---1_exons---phobius-tmhmm	XP_038632475.1	taste receptor type 2 member 13-like [Scyliorhinus canicula]	81	4,00E-112	58.33
Chiloscyllium_plagiosum---CM012986.1-4461908-4462924---1_exons---phobius-tmhmm	XP_038632475.1	taste receptor type 2 member 13-like [Scyliorhinus canicula]	94	6,00E-112	59.01
Chiloscyllium_punctatum---BEZZ01000514.1-303085-304101---1_exons---phobius-tmhmm	XP_038632475.1	taste receptor type 2 member 13-like [Scyliorhinus canicula]	94	1,00E-113	59.56
Hemiscyllium_ocellatum---CM036686.1-43667640-43668656---1_exons---phobius-tmhmm	XP_038632475.1	taste receptor type 2 member 13-like [Scyliorhinus canicula]	94	9,00E-117	59.94
Scyliorhinus_torazame---BFAA01014499.1-26256-27266---1_exons---phobius-tmhmm	XP_038632475.1	taste receptor type 2 member 13-like [Scyliorhinus canicula]	100	0	89.02
Scyliorhinus_canicula---LR744047.1-71451999-71453033---1_exons---phobius-tmhmm	XP_038632475.1	taste receptor type 2 member 13-like [Scyliorhinus canicula]	100	0	100
Pristis_pectinata---CM019896.1-3658922-3659917---1_exons---phobius-tmhmm	XP_051864870.1	taste receptor type 2 member 143 [Pristis pectinata]	100	0	100

Blastp against NCBI NR database excluding Chondrichthyes (taxid:7777)

Query	Match accession	Match description	Query cover	E-value	Perc Identity
Ginglymostoma_cirratum---JAHRHZ010000132.1-202980-203993---1_exons---phobius-tmhmm	XP_006014041.1	taste receptor type 2 member 40-like [Latimeria chalumnae]	87	7,00E-16	28.71
Stegostoma_fasciatum---CM039255.1-41488116-41489120---1_exons---phobius-tmhmm	XP_006010403.1	taste receptor type 2 member 40-like [Latimeria chalumnae]	87	3,00E-13	26.20
Rhincodon_typus---CM038826.1-278077-279270---1_exons---phobius-tmhmm	XP_006010403.1	taste receptor type 2 member 40-like [Latimeria chalumnae]	74	2,00E-17	27.74
Chiloscyllium_plagiosum---CM012986.1-4461908-4462924---1_exons---phobius-tmhmm	XP_006010403.1	taste receptor type 2 member 40-like [Latimeria chalumnae]	87	7,00E-15	27.04
Chiloscyllium_punctatum---BEZZ01000514.1-303085-304101---1_exons---phobius-tmhmm	XP_006010403.1	taste receptor type 2 member 40-like [Latimeria chalumnae]	87	3,00E-17	27.92
Hemiscyllium_ocellatum---CM036686.1-43667640-43668656---1_exons---phobius-tmhmm	XP_006010403.1	taste receptor type 2 member 40-like [Latimeria chalumnae]	87	4,00E-16	27.69
Scyliorhinus_torazame---BFAA01014499.1-26256-27266---1_exons---phobius-tmhmm	XP_006014041.1	taste receptor type 2 member 40-like [Latimeria chalumnae]	88	5,00E-17	30.23
Scyliorhinus_canicula---LR744047.1-71451999-71453033---1_exons---phobius-tmhmm	XP_006014041.1	taste receptor type 2 member 40-like [Latimeria chalumnae]	86	2,00E-15	30.23
Pristis_pectinata---CM019896.1-3658922-3659917---1_exons---phobius-tmhmm	XP_006007462.2	taste receptor type 2 member 40-like [Latimeria chalumnae]	87	1,00E-12	25.66

Supplementary Table 6 | Blastp results for bird *V1R* genes against the NCBI nr database. Results of blastp searches between complete *V1R* genes retrieved in birds against the NCBI nr database are shown, with or without bird sequences (taxid:8782). With the complete database, the same best match was found for all genes. This match was the *V1R* gene we found in *G. californianus* (annotated in its reference genome assembly). In the second blastp search, all best matches were *V1R* genes from lizards or turtles.

Blastp against NCBI NR database

Query	Match accession	Match description	Query cover (%)	E-value	Perc Identity (%)
Pelecanus_crispus---JJRG01012536.1-6398-7351---1_exons---phobius	XP_050751436.1	vomeronasal type-1 receptor 92-like [Gymnogyps californianus]	85	1,00E-160	83.39
Recurvirostra_avosetta---SAYO01028558.1-95480-96460---1_exons---phobius	XP_050751436.1	vomeronasal type-1 receptor 92-like [Gymnogyps californianus]	82	8,00E-165	86.35
Charadrius_alexandrinus---VUYV01000244.1-1075064-1076044---1_exons---phobius	XP_050751436.1	vomeronasal type-1 receptor 92-like [Gymnogyps californianus]	82	3,00E-168	85.98
Balaeniceps_rex---VYZW01041512.1-4739-5722---1_exons---phobius	XP_050751436.1	vomeronasal type-1 receptor 92-like [Gymnogyps californianus]	82	3,00E-161	83.82
Pluvialis_apricaria---CM030010.1-39778926-39779906---1_exons---phobius-tmhmm	XP_050751436.1	vomeronasal type-1 receptor 92-like [Gymnogyps californianus]	82	2,00E-178	90.41
Gymnogyps_californianus---CM030805.1-2929681-2930661---1_exons---phobius	XP_050751436.1	vomeronasal type-1 receptor 92-like [Gymnogyps californianus]	82	0,00E+00	100
Podiceps_cristatus---KL284334.1-10240-11064---1_exons	XP_050751436.1	vomeronasal type-1 receptor 92-like [Gymnogyps californianus]	84	3,00E-127	79.57
Himantopus_leucocephalus---RSEF01000004.1-96440577-96441515---1_exons	XP_050751436.1	vomeronasal type-1 receptor 92-like [Gymnogyps californianus]	71	2,00E-120	78.17
Himantopus_himantopus---VXBK01010287.1-455584-456522---1_exons	XP_050751436.1	vomeronasal type-1 receptor 92-like [Gymnogyps californianus]	71	6,00E-118	77.29

Blastp against NCBI NR database excluding Birds (taxid:8782)

Query	Match accession	Match description	Query cover (%)	E-value	Perc Identity (%)
Pelecanus_crispus---JJRG01012536.1-6398-7351---1_exons---phobius	XP_048364726.1	vomeronasal type-1 receptor 92-like [Sphaerodactylus townsendi]	98	4,00E-145	64.58
Recurvirostra_avosetta---SAYO01028558.1-95480-96460---1_exons---phobius	XP_048364726.1	vomeronasal type-1 receptor 92-like [Sphaerodactylus townsendi]	94	1,00E-151	68.81
Charadrius_alexandrinus---VUYV01000244.1-1075064-1076044---1_exons---phobius	XP_048364726.1	vomeronasal type-1 receptor 92-like [Sphaerodactylus townsendi]	96	3,00E-153	67.81
Balaeniceps_rex---VYZW01041512.1-4739-5722---1_exons---phobius	XP_048364726.1	vomeronasal type-1 receptor 92-like [Sphaerodactylus townsendi]	94	2,00E-147	67.31
Pluvialis_apricaria---CM030010.1-39778926-39779906---1_exons---phobius-tmhmm	XP_048364726.1	vomeronasal type-1 receptor 92-like [Sphaerodactylus townsendi]	94	3,00E-165	73.14
Gymnogyps_californianus---CM030805.1-2929681-2930661---1_exons---phobius	XP_048364726.1	vomeronasal type-1 receptor 92-like [Sphaerodactylus townsendi]	94	1,00E-157	69.90
Podiceps_cristatus---KL284334.1-10240-11064---1_exons	XP_024049569.1	vomeronasal type-1 receptor A14-like [Terrapene carolina triunguis]	82	4,00E-96	63.76
Himantopus_leucocephalus---RSEF01000004.1-96440577-96441515---1_exons	XP_048364726.1	vomeronasal type-1 receptor 92-like [Sphaerodactylus townsendi]	76	8,00E-119	71.90
Himantopus_himantopus---VXBK01010287.1-455584-456522---1_exons	XP_048364726.1	vomeronasal type-1 receptor 92-like [Sphaerodactylus townsendi]	76	1,00E-115	70.25

Supplementary Table 7 | Ecological parameters for mammals, birds and ray-finned fishes. For each chemoreceptor families, we performed a two-sided pGLS between the number of complete genes against each ecological variable (see Supplementary Data 1).

	Predictor	Source	Type	Description
Actinopterygii	AnaCat	fishbase	Discrete	non-migratory;oceanodromous;potamodromous;anadromous;amphidromous;catadromous;oceano-estuarine
Actinopterygii	DemersPelag	fishbase	Discrete	benthopelagic;demersal;pelagic;reef-associated;pelagic-oceanic;pelagic-neritic;bathypelagic;bathydemersal
Actinopterygii	DepthRangeDeep	fishbase	Continuous	Living depth
Actinopterygii	EnvTemp	fishbase	Discrete	Climate zone. boreal;deep-water;high altitude;polar;subtropical;temperate;tropical
Actinopterygii	Habitat	fishbase	Discrete	Possible values : Fresh_Brack_Salt;Fresh_Brack;Salt_Brack;Fresh_Salt;Fresh;Brack,Salt
Actinopterygii	TempPreferred	fishbase	Continuous	Mean preferred temperature
Actinopterygii	Diet	fishbase	Discrete	trophic level ≤ 2.19 were classified as herbivores, species with a trophic level ≥ 2.2 and ≤ 2.79 were classified as omnivores while species with a trophic level ≥ 2.8 were classified as carnivores
Aves	Migration	AVONET	Discrete	1 = Sedentary. ; 2 = Partially migratory, i.e. minority of population migrates long distances, or most of population undergoes short-distance migration, nomadic movements, distinct altitudinal migration, etc.; 3 = Migratory, i.e. majority of population undertakes long-distance migration
Aves	Trophic.Niche	AVONET	Discrete	Frugivore = species obtaining at least 60% of food resources from fruit; Granivore = species obtaining at least 60% of food resources from seeds or nuts; Nectarivore = species obtaining at least 60% of food resources from nectar;<br>Herbivore = species obtaining at least 60% of food resources from other plant materials in non-aquatic systems, including leaves, buds, whole flowers etc.; Herbivore aquatic = species obtaining at least 60% of food resources from plant materials in aquatic systems, including algae and aquatic plant leaves; Invertivore = species obtaining at least 60% of food resources from invertebrates in terrestrial systems, including insects, worms, arachnids, etc.;<br>Vertivore = species obtaining at least 60% of food resources from vertebrate animals in terrestrial systems, including mammals, birds, reptiles etc.; Aquatic Predator = species obtaining at least 60% of food resources from vertebrate and invertebrate animals in aquatic systems, including fish, crustacea, molluscs, etc; Scavenger = species obtaining at least 60% of food resources from carrion, offal or refuse; Omnivore = Species using multiple niches, within or across trophic levels, in relatively equal proportions
Aves	Diet	AVONET	Discrete	Herbivore = species obtaining at least 70% of food resources from plants; Carnivore = species obtaining at least 70% of food resources by consuming live invertebrate or vertebrate animals; Omnivore = species obtaining resources from multiple trophic level in roughly equal proportion. Scavenger species were classified as carnivores
Aves	Habitat.Density	AVONET	Discrete	1 = Dense habitats. Species primarily lives in the lower or middle storey of forest, or in dense thickets, dense shrubland etc.; 2 = Semi-open habitats. Species primarily lives in open shrubland, scattered bushes, parkland, low dry or deciduous forest, thorn forest. ; 3 = Open habitats. Species primarily lives in desert, grassland, open water, low shrubs, rocky habitats, seashores, cities. Also applies to species living mainly on top of forest canopy (i.e. mostly in the open)
Aves	Habitat	AVONET	Discrete	Desert (= drylands and other open arid habitats, often sandy with very sparse vegetation);<br>Rock (= rocky substrate typically with no or very little vegetation, including rocky outcrops, rocky coastlines, arid stony steppes, rocky mountaintops and mountain slopes);<br>Grassland (= open dry to moist grass-dominated landscapes, at all elevations);<br>Shrubland (= low stature bushy habitats, included thornscrub, thorny or arid savanna, caatinga, xerophytic shrubland and coastal scrub);<br>Woodland (= medium stature tree-dominated habitats, including Acacia woodland, riparian woodlands, mangrove forests, forest edges, also more open parkland with scattered taller trees);<br>Forest (= tall tree-dominated vegetation with more or less closed canopy, including palm forest);<br>Human modified (urban landscapes, intensive agriculture, gardens);<br>Wetland (= wide range of freshwater aquatic habitats including lakes, marshes, swamps and reedbeds);<br>Riverine (= associated with rivers and streams at all elevations);<br>Coastal (= intertidal zones within immediate vicinity of beaches, estuaries, brackish to salty marshes, including mudflats, lagoons, alkaline wetlands, coastal dunes and harbours);<br>Marine (= pelagic, on sea near coasts, including species in the intertidal zone on beaches, and those pelagic species nesting near the sea on cliffs, islets and islands).
Aves	Primary.Lifestyle	AVONET	Discrete	Aerial = species spends much of the time in flight, and hunts or forages predominantly on the wing;<br>Terrestrial = species spends majority of its time on the ground, where it obtains food while either walking or hopping (note this includes species that also wade in water with their body raised above the water);<br>Insessorial = species spends much of the time perching above the ground, either in branches of trees and other vegetation (i.e. arboreal), or on other raised substrates including rocks, buildings, posts, and wires;<br>Aquatic = species spends much of the time sitting on water, and obtains food while afloat or when diving under the water's surface; Generalist = species has no primary lifestyle because it spends time in different lifestyle classes
Mammals	ActivityCycle	Pantheria	Discrete	Activity cycle of each species measured for non-captive populations; adult or age unspecified individuals, male, female, or sex unspecified individuals; primary, secondary, or extrapolated sources Species were defined as (1) nocturnal only, (2) nocturnal/crepuscular, cathemeral, crepuscular or diurnal/crepuscular and (3) diurnal only.
Mammals	ForStrat.Value	Elton	Discrete	M - marine, G - ground level, including aquatic foraging (see ForStrat-Comment), S- scansorial, Ar - arboreal, A- aerial.
Mammals	HomeRange_Indiv_km2	Pantheria	Continuous	Size of the area within which everyday activities of individuals are typically restricted, estimated by either direct observation, radio telemetry, trapping or unspecified methods over any duration of time, using non-captive populations; male, female, or sex unspecified individuals; primary, secondary, or extrapolated sources; all measures of central tendency; in all localities
Mammals	HomeRange_km2	Pantheria	Continuous	ln(home range) = species + sex + estimation method + range measure [across class]
Mammals	MaxLongevity_m	Pantheria	Continuous	Maximum adult age measured either through direct observation, capture-recapture estimates, projected from physical wear or unspecified, using captive, wild, provisioned, or unspecified populations; male, female, or sex unspecified individuals; primary, secondary, or extrapolated sources; in all localities.
Mammals	PopulationDensity_n.km2	Pantheria	Continuous	ln(population density) = species + estimation method + study area type [across class]
Mammals	PopulationGrpSize	Pantheria	Continuous	Number of individuals, adults or definition unspecified in a group that spends the majority of their time in a 24 hour cycle together, measured over any duration of time, using non-captive populations; male, female, or sex unspecified individuals; primary, secondary, or extrapolated sources; all measures of central tendency; in all localities.
Mammals	Diet	Mammal Diet	Discrete	Species were defined as (1) herbivore (not vertebrate and/or invertebrate), (2) omnivore (vertebrate and/or invertebrate plus any of the other categories) and (3) carnivore (vertebrate and/or invertebrate only)
Mammals	WeaningAge_d	Pantheria	Continuous	Age when primary nutritional dependency on the mother ends and independent foraging begins to make a major contribution to the offspring's energy requirements, measured as either weaning/lactation length, nutritionally independent, first solid food, last observed nursing, age at first flight (bats only), age at pouch exit or length of teat Attachment (marsupials only) or unspecified definition, using captive, wild, provisioned, or unspecified populations; male, female, or sex unspecified individuals; primary, secondary, or extrapolated sources; all measures of central tendency; in all localities.

Supplementary Fig. 1 | BUSCO completeness assessment. Number of genomes in relation to the completeness threshold (% of complete BUSCO genes). BUSCO raw results can be found in Supplementary Data 1. Source data are provided as a Source Data file.

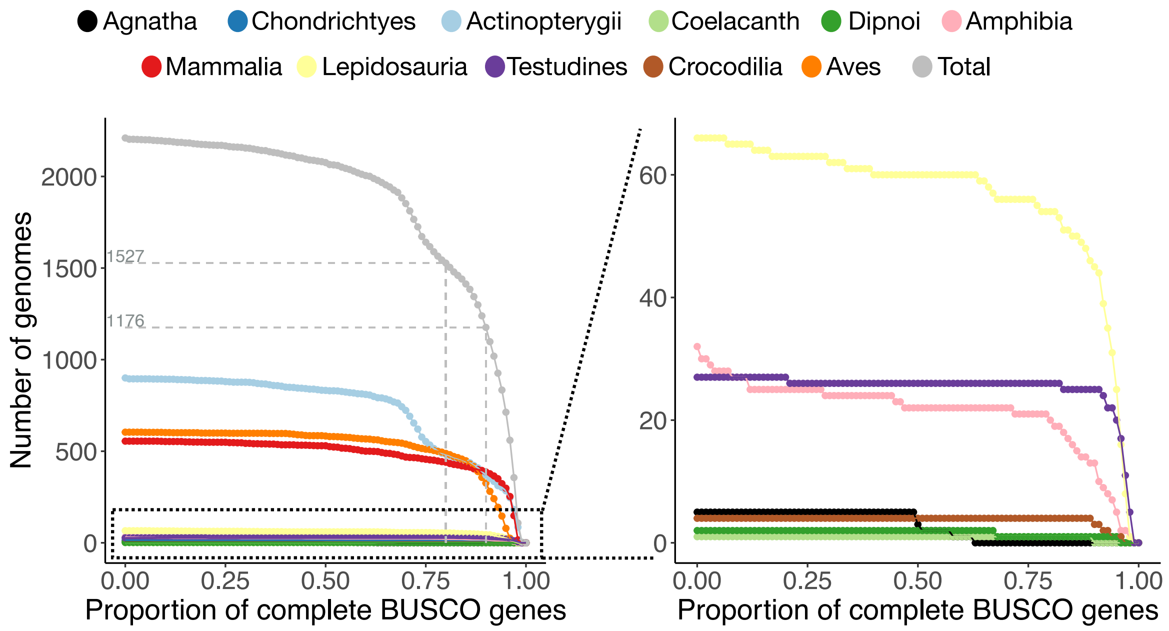

Supplementary Fig. 2 | Low BUSCO scores in agnathan genomes. a, Number of common fragmented/missing genes predicted by BUSCO in the five agnathan genomes. **b**, Distribution of the number of common fragmented/missing genes expected at random, considering gene lengths (red) or not (blue). The mean number of extracted genes in common was 208 and 69 when considering the gene length or not, respectively, which is much less than the observed 1,014 genes. We thus hypothesize that these low scores are a consequence of applying the vertebrata_odb10 database, which may have been built without taking into consideration, or with an underrepresentation, of agnathan species. Source data are provided as a Source Data file.

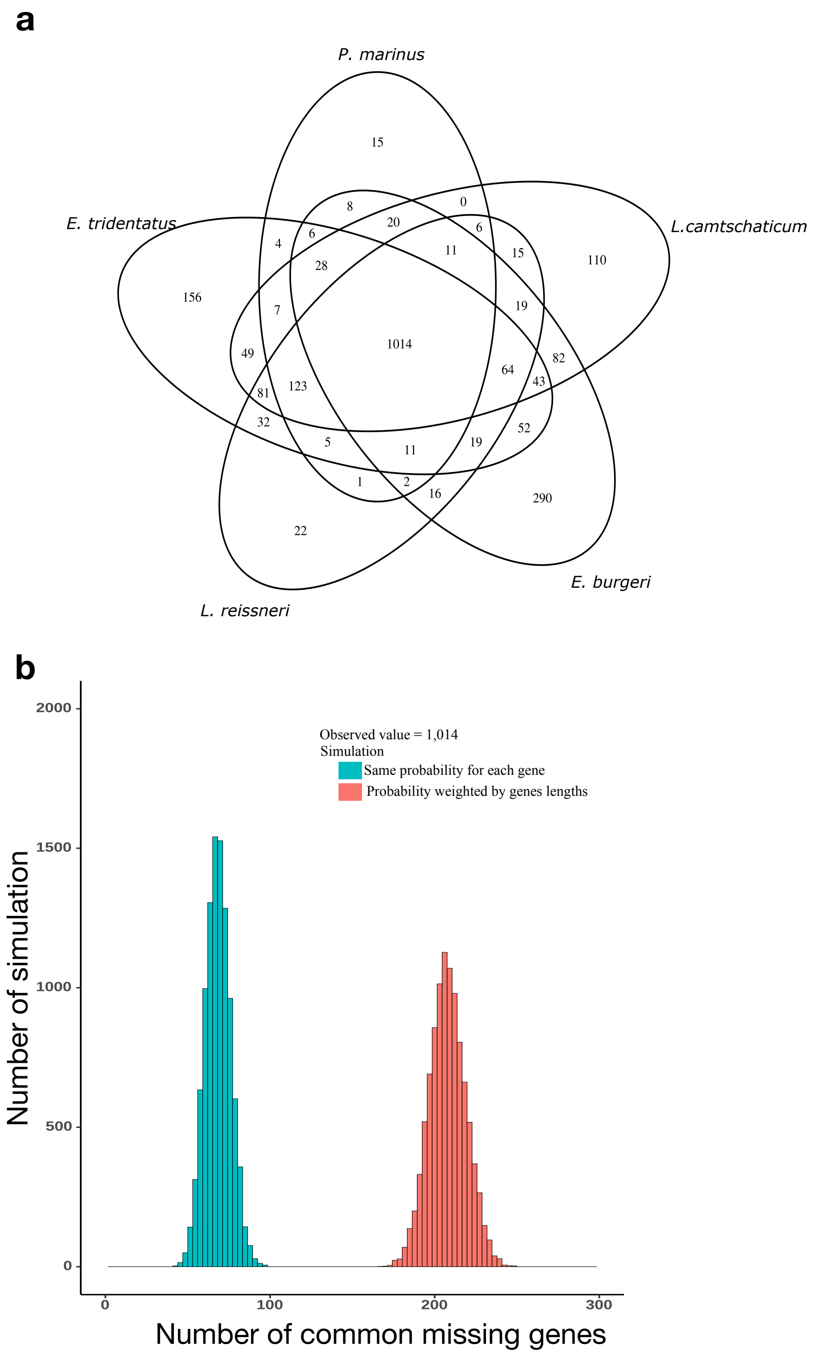

Supplementary Fig. 3 | The case of *Sceloporus occidentalis*. DNA percentage identity between lepidosaurs chemoreceptors and their best blastn match among amphibian chemoreceptors. Boxplot showing the DNA identity for each lepidosaur species (BUSCO80 dataset) per *OR* (**a**), *TAAR* (**c**), *V1R* (**e**), *V2R* (**g**), *T1R* (**i**), *T2R* (**k**) and for *S. occidentalis* and close species (*Sceloporus tristichus, Sceloporus undulates and Phrynosoma platyrhinos*) per *OR* (**b**), *TAAR* (**d**), *V1R* (**f**), *V2R* (**h**), *T1R* (**j**), *T2R* (**l**); species are missing if no blastn match (e-value < 10) was found. The colors of the dots correspond to the chemoreceptor subclade. Chemoreceptors in subclades that are only present in *S. occidentalis* always have a very high DNA identity with amphibian chemoreceptors. All these blastn results and pairwise DNA identity values are reported in Supplementary Data 1. Source data are provided as a Source Data file.

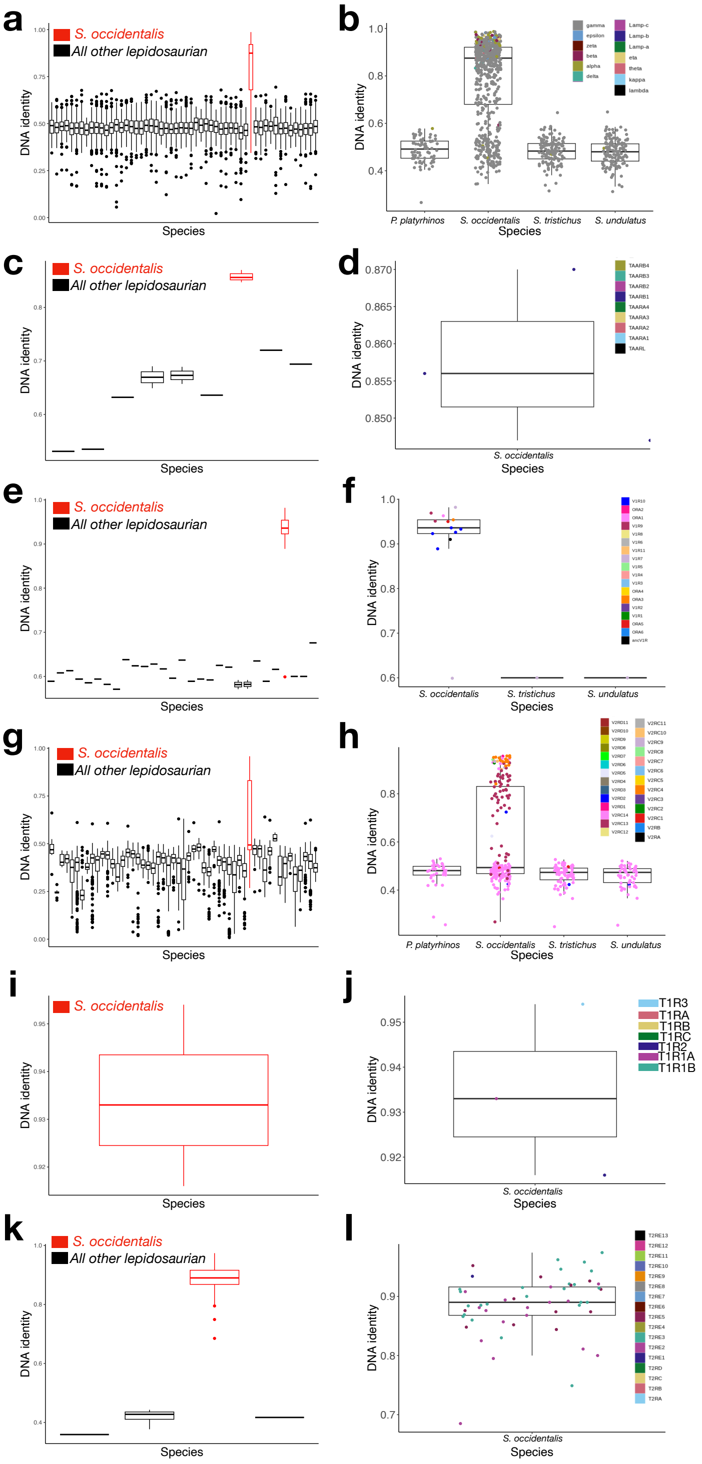

Supplementary Fig. 4 | Comparison between previous studies and this work regarding the number of chemoreceptor genes retrieved from genome assemblies. a, *OR* genes; **b**, *TAAR* genes; **c**, *V1R* genes; **d**, *V2R* genes; **e**, *T1R* genes; **f**, *T2R* genes. Pearson correlation coefficients and *P*-values are indicated for each comparison. The solid black lines represent the linear regression lines and the grey area represents confidence intervals. Dashed lines indicate slope = 1. In order not to underestimate the number of genes from previous studies, we only considered studies in which both the number of functional and the number of pseudogenes were reported. Since the different studies used different criteria to differentiate between incomplete and complete genes, we only compared the total number of genes (complete and incomplete genes). The only exception was *Neoceratodus forsteri*, for which we only retrieved the number of functional genes, but we considered that this species was of a particular interest given its phylogenetic position. Thus, for this species, the numbers reported below only correspond to complete genes (in both axis). The numbers of *OR* genes retrieved in Christmas et al. 2023 are not included in this figure (see Supplementary Fig. 5). References of previous studies considered here and the number of genes are reported in Supplementary Data 1. Source data are provided as a Source Data file.

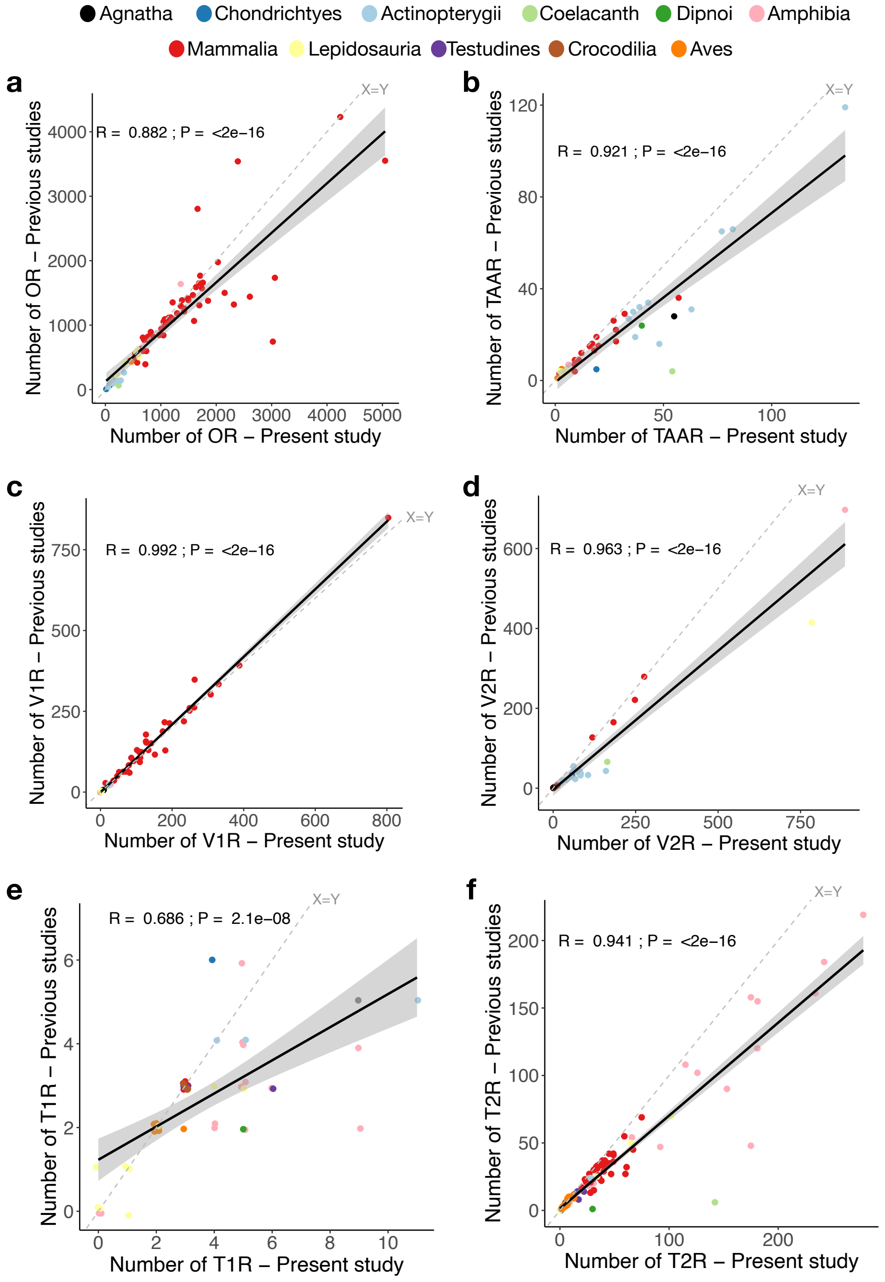

Supplementary Fig. 5 | Comparison of the mammalian *OR* gene repertoire between Christmas et al. 2023 and our study. Comparison of the number of *OR* genes retrieved in Christmas et al. 2023[59] and our study (n=249). **a**, Complete *OR* genes; **b**, Incomplete *OR* genes; **c**, Total *OR* genes. Pearson correlation coefficients and *P*-values are indicated for each comparison. The solid black lines represent the linear regression lines and the grey area represents confidence intervals. Dashed lines indicate slope = 1. **d**, Two-sided pGLS results between the number of total *OR* genes (this study) and the number of turbinals (Christmas et al. 2023) (BUSCO80 dataset, n=49). The pGLS $R^{2,}$ p-value and λ are indicated. This correlation was even higher when considering the number of complete *OR* genes instead of the total number of OR genes (pGLS: $R^2 = 0.37$, P = 3e-6, λ=0.946), but no correlation was found with TAAR, V1R or V2R genes. A blue point indicates that the same genome assembly was used in both studies, whereas a red point indicates that different genome assemblies for the same species were used in the two studies. The number of OR genes and the number of turbinals retrieved in Christmas et al. 2023 can be found in Supplementary Data 1. Source data are provided as a Source Data file.

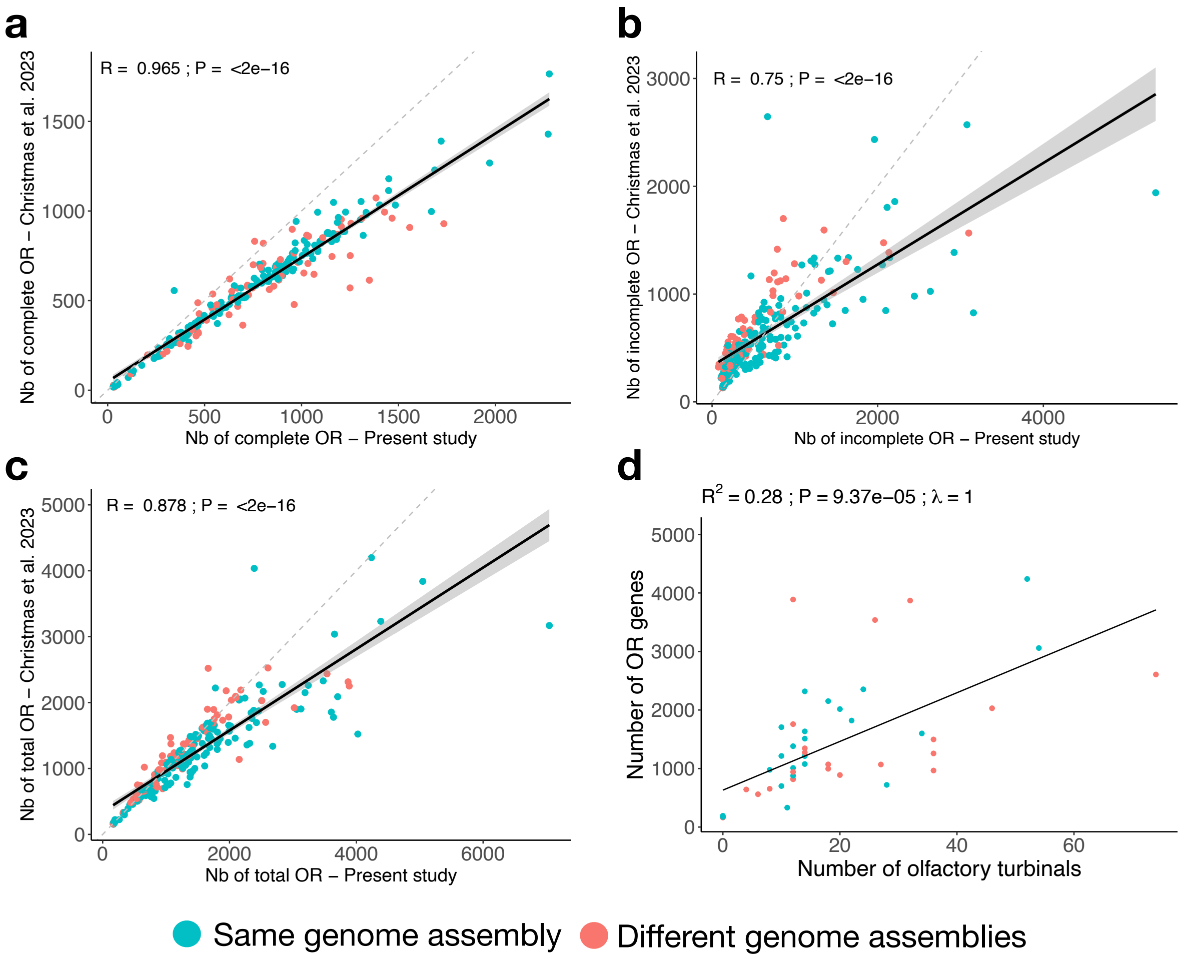

Supplementary Fig. 6 | Correlations between the numbers of complete chemoreceptor genes across chemoreceptor families. Correlations (BUSCO80 dataset) are shown for *OR* versus *TAAR* (**a**), *OR* versus *V2R* (**b**), *OR* versus *V1R* (**c**), *TAAR* versus *V2R* (**d**), *TAAR* versus *V1R* (**e**), *V1R* versus *V2R* (**f**), between the two taste receptor families *T1R* and *T2R* (**g**), between the number of complete olfactory receptors (OLR) and the number of complete taste receptors (TR) (**h**), and between *V1R* and *V2R* of mammals that have at least one *V1R* and one *V2R* (**i**). Phylogenetic linear regression lines, as well as their slope-intercept equation, R-squared and *P*-values are shown with the colors associated to the species classes (only reported if significant). Dashed lines represent slope = 1. The number of chemoreceptors per species can be found in Supplementary Data 1. Source data are provided as a Source Data file.

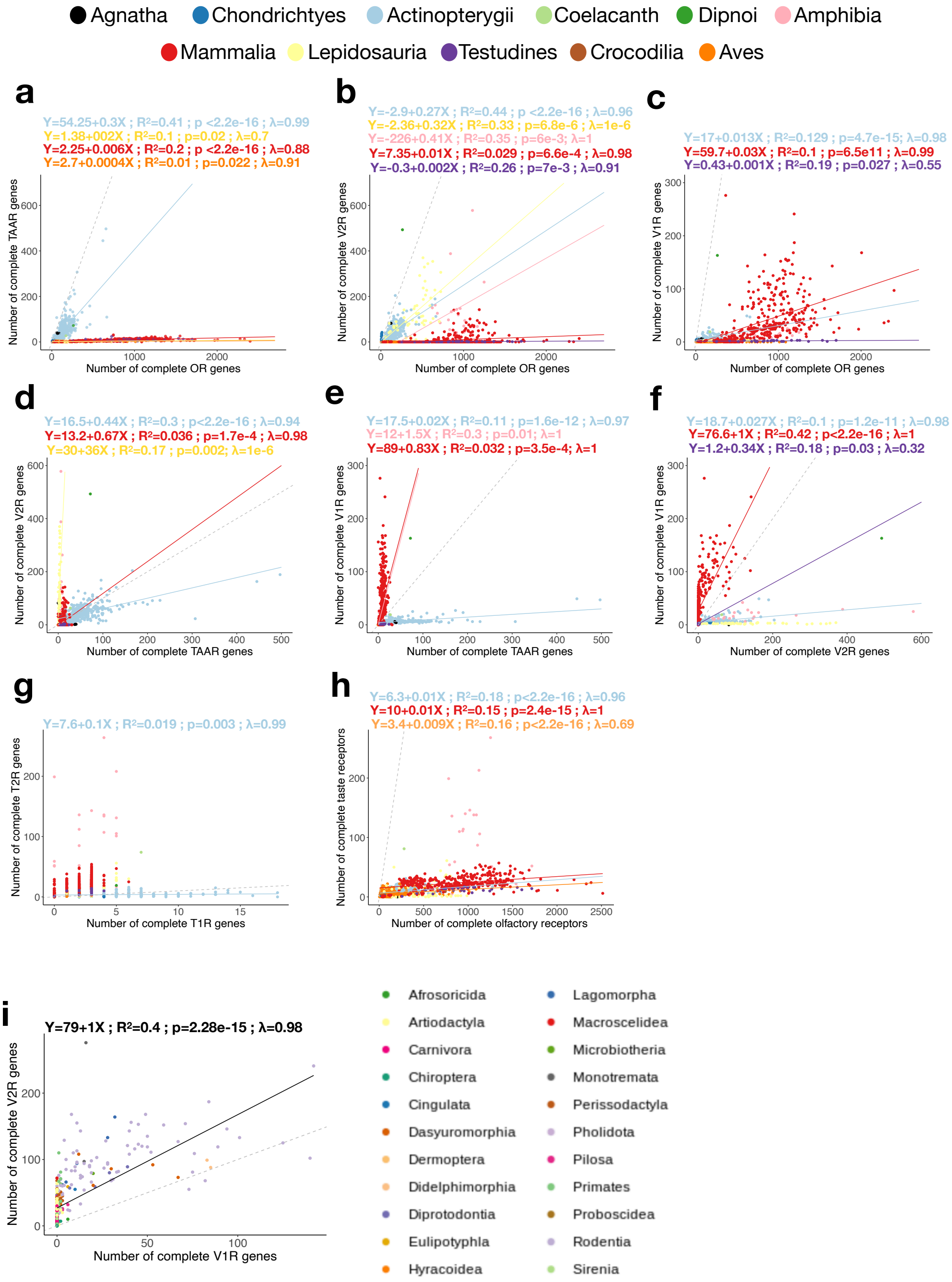

Supplementary Fig. 7 | Summary of correlations between the number of complete genes across chemoreceptor families. Correlations were assessed using a two-sided pGLS test. **a**, Results from the BUSCO80 dataset (also shown in Fig. 1b). **b**, Results from the BUSCO90 dataset. **c**, Results when considering only chromosome-scale assemblies for ray-finned fishes, mammals and birds. Presence of a circle indicates P_{PGLS} <0.05 and the circle is colored according to the pGLS R^2-value. Correlations observed in mammals, birds and ray-finned fishes hold true when taking only chromosome-scale assemblies into account (N = 126, N = 83 and N = 210 respectively), except for the correlation between *OR* and *V2R* genes in mammals, for which the *P*-value become non-significant (pGLS: P = 0.09, R^2 = 0.2). Source data are provided as a Source Data file.

a) BUSCO80

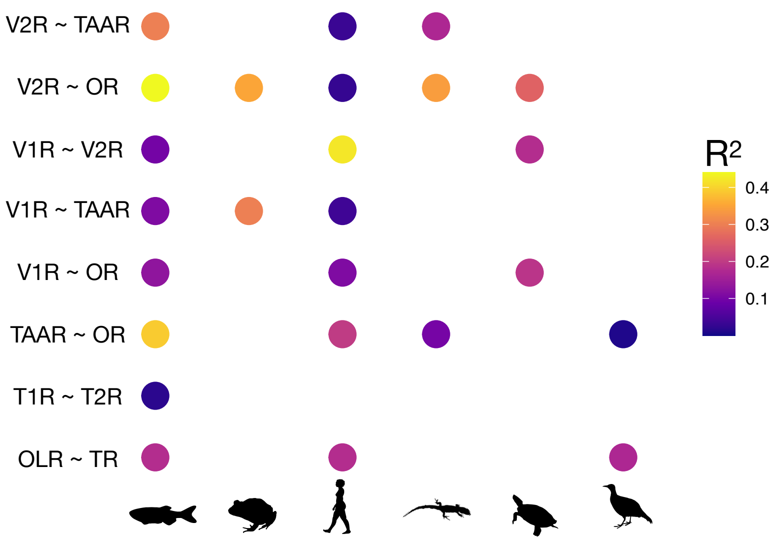

b) BUSCO90

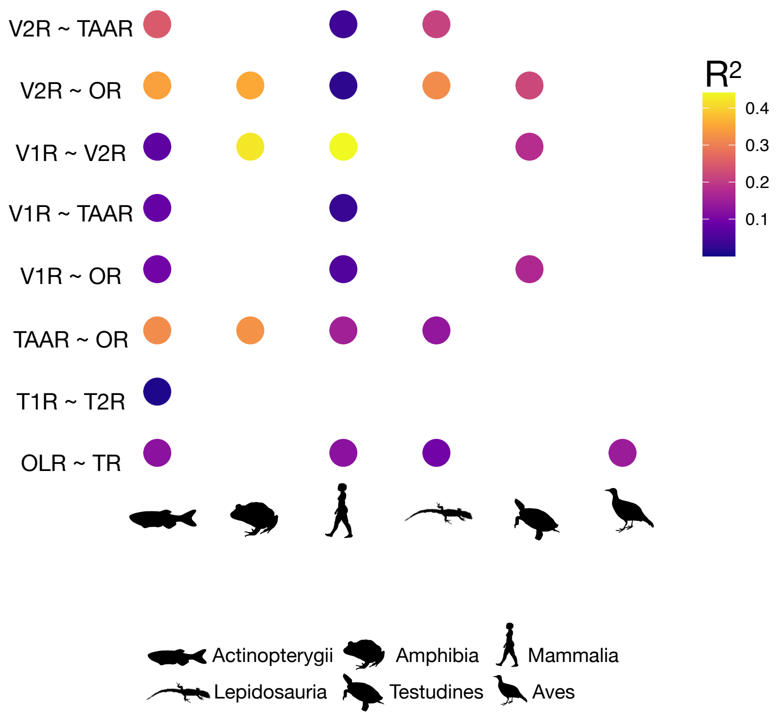

c) Chromosome-scale assemblies

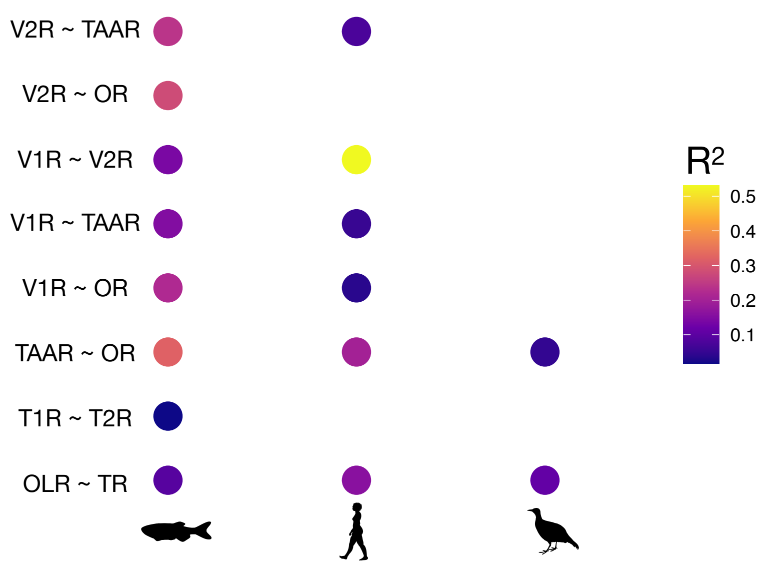

Supplementary Fig. 8 | Correlations between the number of chemoreceptors and genome size (BUSCO80). Two-sided pGLS results between the number of complete genes or the total number of genes (complete genes and incomplete genes) in each chemoreceptor gene family and genome size (in Mb) are shown for the BUSCO80 dataset. Significant correlations (P_{PGLS} <0.05) are highlighted in green. The number of complete and total genes was significantly correlated to the genome size for all chemoreceptor families in birds, mammals and ray-finned fishes, except for the *V2R* family of mammals, but pGLS R^2-values were low (close or below 0.1). Thus, correlation between gene numbers across chemoreceptor families were re-computed taking the genome size into account (see Supplementary Table. 2). We removed *Tachyglossus aculeatus* from the pGLS analysis between the total number of *OR* genes and genome size, as we retrieved 8,952 incomplete genes in this species, far more than in any other mammal, which most likely results from an assembly artifact. In a blastx seach against the NCBI nr database, all these incomplete genes best matched with known *OR* genes. Source data are provided as a Source Data file.

BUSCO80

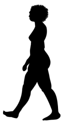

Response	R²	pvalue	lambda	regression
Complete OR	0.013	0.022	0.99	Y= 0.076 + 945.98
Total OR	0.016	0.0112	0.99	Y= 0.144X + 862.16
Complete TAAR	0.023	0.34	0.89	Y= 0.001X + 7.81
Total TAAR	0.072	0.0927	0.88	Y= 0.002X + 8.58
Complete V1R	0.004	0.209	1	Y= 0.004X + 85.58
Total V1R	0.014	0.0179	1	Y= 0.02X + 263.67
Complete V2R	0.0016	0.425	0.98	Y= 0.002X + 14.71
Total V2R	0.0018	0.398	1	Y= 0.006X + 73.13
Complete T2R	0.023	0.342	0.98	Y= 0.001X + 16.44
Total T2R	0.0058	0.131	0.98	Y= 0.002X + 25.94

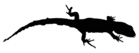

Response variable	R²	pvalue	lambda	regression
Complete OR	2.6e-05	0.971	1	Y= -0.003X + 391.14
Total OR	0.012	0.439	1	Y= 0.06X + 310.42
Complete TAAR	0.018	0.338	0.8	Y= 0X + 3.27
Total TAAR	0.0046	0.632	0.67	Y= 0X + 4
Complete V1R	0.035	0.185	0	Y= 0X + 1.98
Total V1R	0.053	0.1	0.66	Y= 0X + 2.09
Complete V2R	0.021	0.303	0.33	Y= -0.037X + 172
Total V2R	0.005	0.618	0.21	Y= -0.037X + 273.18
Complete T2R	0.11	0.0178	1	Y= 0.009X + -4.05
Total T2R	0.2	0.000996	1	Y= 0.019X + -14.36

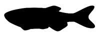

Response variable	R²	pvalue	lambda	regression
Complete OR	0.047	3.25e-06	0.96	Y= 0.043X + 219.82
Total OR	0.072	7.03e-09	0.97	Y= 0.068X + 243.09
Complete TAAR	0.051	1.5e-06	1	Y= 0.023X + 101.36
Total TAAR	0.076	2.89e-09	1	Y= 0.043X + 149.84
Complete V1R	0.025	0.000833	0.98	Y= 0.001X + 18.81
Total V1R	0.067	2.44e-08	0.99	Y= 0.002X + 20.04
Complete V2R	0.016	0.00718	0.93	Y= 0.01X + 60.81
Total V2R	0.037	4.1e-05	0.94	Y= 0.018X + 63.71
Complete T1R	0.023	0.00137	0.89	Y= 0.001X + 3.86
Total T1R	0.033	0.000112	0.94	Y= 0.002X + 4.21
Complete T2R	0.042	1.1e-05	0.99	Y= 0.001X + 5.7
Total T2R	0.13	3.77e-15	0.98	Y= 0.003X + 5.49

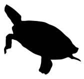

Response variable	R²	pvalue	lambda	regression
Complete OR	0.05	0.273	0.59	Y= 0.48X + -97.56
Total OR	0.11	0.0988	0	Y= 0.942X + -674.42
Complete TAAR	0.00087	0.887	0.96	Y= -0.001X + 12.47
Total TAAR	0.082	0.156	0.49	Y= 0.01X + -8.24
Complete V1R	0.092	0.133	0.23	Y= 0.002X + -1.93
Total V1R	0.03	0.395	0.92	Y= 0.001X + 1.3
Complete V2R	0.017	0.529	1	Y= -0.001X + 2.4
Total V2R	0.0066	0.694	0.98	Y= 0.001X + 1.14
Complete T2R	0.0088	0.649	0.92	Y= 0.002X + 5.86
Total T2R	0.039	0.334	0	Y= 0.007X + 5.85

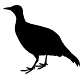

Response variable	R²	pvalue	lambda	regression
Complete OR	0.97	5.29e-12	0.55	Y= 0.411X + -457.05
Total OR	0.091	2.33e-11	0.5	Y= 0.642X + -716.77
Complete TAAR	0.0014	0.411	0.95	Y= 0.001X + 2.41
Total TAAR	0.034	0.204	0.86	Y= 0.001X + 3.41
Complete T2R	0.03	1.62e-04	0.69	Y= 0.005X + -5.2
Total T2R	0.03	1.41e-04	0.67	Y= 0.006X + -4.72

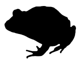

Response variable	R²	pvalue	lambda	regression
Complete OR	0.02	0.556	0	Y= 0.004X + 878.64
Total OR	0.036	0.422	0	Y= 0.007X + 1080.28
Complete TAAR	0.25	0.0249	0	Y= 0X + 4.26
Total TAAR	0.016	0.596	0.9	Y= 0X + 6.54
Complete V1R	0.3	0.013	0.87	Y= 0.001X + 13.4
Total V1R	0.072	0.252	0.25	Y= 0.001X + 20.13
Complete V2R	0.011	0.663	0.65	Y= -0.003X + 170.47
Total V2R	0.00035	0.937	0.48	Y= -0.001X + 329.07
Complete T2R	0.033	0.447	0.96	Y= 0.002X + 59.82
Total T2R	0.087	0.207	0.95	Y= 0.004X + 78.31

Supplementary Fig. 9 | Correlations between the number of chemoreceptors and genome size (BUSCO90). Two-sided pGLS results between the number of complete genes or the total number of genes (complete genes and incomplete genes) in each chemoreceptor gene family and genome size (in Mb) are shown for the BUSCO90 dataset. Significant correlations (P_{PGLS} <0.05) are highlighted in green. The number of complete and total genes was significantly correlated to the genome size for all chemoreceptor families in birds, mammals and ray-finned fishes, except for the *V2R* gene family in mammals, but pGLS R^2-values were low (close or below 0.1). Thus, correlation between gene numbers across chemoreceptor families were re-computed taking the genome size into account. As in Supplementary Fig. 1, *Tachyglossus aculeatus* was removed from the analysis due to the unusually high number of incomplete genes retrieved in its genome. In a blastx seach against the NCBI nr database, all these incomplete genes best matched with known *OR* genes. Source data are provided as a Source Data file.

BUSCO90

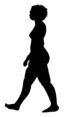

Response variable	R²	pvalue	lambda	regression
Complete OR	0.21	0.00657	0.99	Y= 0.097X + 892.2
Total OR	0.03	0.00121	0.99	Y= 0.199X + 707.4
Complete TAAR	0.0048	0.2	0.88	Y= 0.001X + 7.25
Total TAAR	0.01	0.0588	0.87	Y= 0.002X + 7.3
Complete V1R	0.0093	0.0747	0.99	Y= 0.006X + 80.2
Total V1R	0.03	0.00126	1	Y= 0.028X + 244.95
Complete V2R	0.0099	0.0658	0.97	Y= 0.004X + 8.26
Total V2R	0.0071	0.119	1	Y= 0.013X + 56.3
Complete T2R	0.0033	0.289	0.99	Y= 0.001X + 16.46
Total T2R	0.022	0.00622	0.99	Y= 0.003X + 21.82

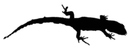

Response variable	R²	pvalue	lambda	regression
Complete OR	0.01	0.52	1	Y= -0.053X + 516.23
Total OR	4.8e-07	0.996	1	Y= 0X + 459.28
Complete TAAR	0.011	0.504	0.71	Y= 0X + 2.97
Total TAAR	0.00055	0.881	0.59	Y= 0X + 3.12
Complete V1R	0.042	0.189	0	Y= 0X + 1.96
Total V1R	0.004	0.688	1	Y= 0X + 2.98
Complete V2R	0.058	0.118	0.37	Y= -0.06X + 225.01
Total V2R	0.033	0.245	0.2	Y= -0.085X + 388.58
Complete T2R	0.037	0.214	1	Y= 0.005X + 6.79
Total T2R	0.14	0.0153	0.99	Y= 0.016X + -4.58

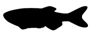

Response variable	R²	pvalue	lambda	regression
Complete OR	0.029	0.00158	0.96	Y= 0.031X + 244.3
Total OR	0.051	2.4e-05	0.97	Y= 0.052X + 272.01
Complete TAAR	0.029	0.00164	1	Y= 0.017X + 112.9
Total TAAR	0.053	1.72e-05	1	Y= 0.037X + 161.59
Complete V1R	0.012	0.0427	0.97	Y= 0.001X + 19.54
Total V1R	0.037	0.000356	1	Y= 0.001X + 21.29
Complete V2R	0.0041	0.237	0.94	Y= 0.005X + 70.93
Total V2R	0.02	0.00898	0.95	Y= 0.012X + 75.47
Complete T1R	0.013	0.032	0.89	Y= 0.001X + 4.45
Total T1R	0.024	0.036	0.97	Y= 0.001X + 4.76
Complete T2R	0.022	0.00622	0.99	Y= 0.003X + 21.82
Total T2R	0.12	8.77e-11	0.99	Y= 0.003X + 5.8

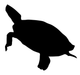

Response variable	R²	pvalue	lambda	regression
Complete OR	0.041	0.333	0.6	Y= 0.438X + -2.64
Total OR	0.11	0.109	0	Y= 0.934X + -650.49
Complete TAAR	0.00038	0.927	0.96	Y= 0X + 10.22
Total TAAR	0.072	0.194	0.46	Y= 0.009X + -6.88
Complete V1R	0.091	0.144	0.22	Y= 0.002X + -1.92
Total V1R	0.035	0.368	0.91	Y= 0.001X + 1.1
Complete V2R	0.065	0.219	1	Y= -0.001X + 3.77
Total V2R	0.0061	0.711	0.97	Y= 0.001X + 1.11
Complete T2R	0.0039	0.765	1	Y= -0.001X + 11.46
Total T2R	0.038	0.353	0	Y= 0.007X + 6.26

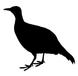

Response variable	R²	pvalue	lambda	regression
Complete OR	0.11	2.83e-9	0.58	Y= 0.417X + -402.6
Total OR	0.12	5.04e-10	0.56	Y= 0.635X + -613.86
Complete TAAR	0.0007	0.641	1	Y= 0.001X + 2.87
Total TAAR	0.012	0.056	0.86	Y= 0.001X + 3.15
Complete T2R	0.028	0.0031	0.84	Y= 0.005X + -4.16
Total T2R	0.023	0.0074	0.77	Y= 0.006X + -2.47

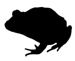

Response variable	R²	pvalue	lambda	regression
Complete OR	0.054	0.445	0	Y= 0.03X + 787.14
Total OR	5.9e-06	0.994	0	Y= 0X + 1125.52
Complete TAAR	0.011	0.731	1	Y= 0X + 4.92
Total TAAR	0.076	0.363	0.99	Y= 0.001X + 4.53
Complete V1R	0.0082	0.768	0.93	Y= 0X + 15.52
Total V1R	0.012	0.726	1	Y= 0.002X + 24.08
Complete V2R	0.041	0.508	1	Y= -0.018X + 247.35
Total V2R	0.0037	0.844	1	Y= 0.007X + 365.49
Complete T2R	0.015	0.689	0.99	Y= 0.004X + 57.66
Total T2R	0.019	0.656	0.97	Y= 0.004X + 82.42

Supplementary Fig. 10 | Phylogenies with full species names. Vertebrate (sub)class phylogenies with species names displayed (BUSCO80 dataset). **a**, Agnatha; **b**, Chondrichthyes; **c**, Actinopterygii; **d**, Amphibia; **e**, Mammalia; **f**, Lepidosauria; **g**, Testudines; **h**, Aves and Crocodilia. Terminal branches and species names are colored according to order based on the NCBI taxonomy database.

a) Agnatha

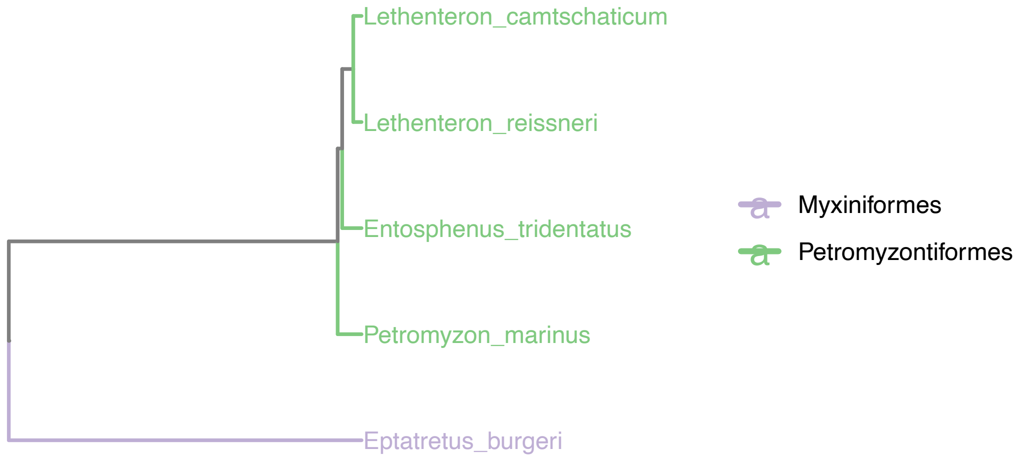

b) Chondrichthyes

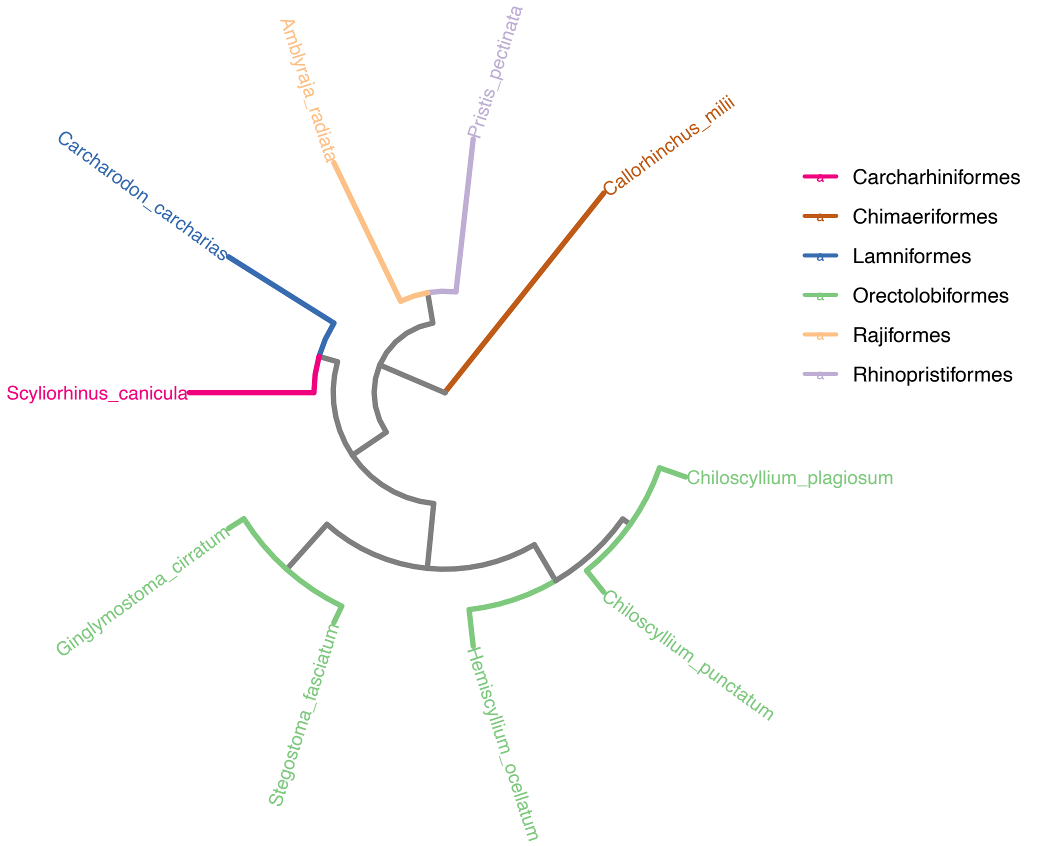

c) Actinopterygii

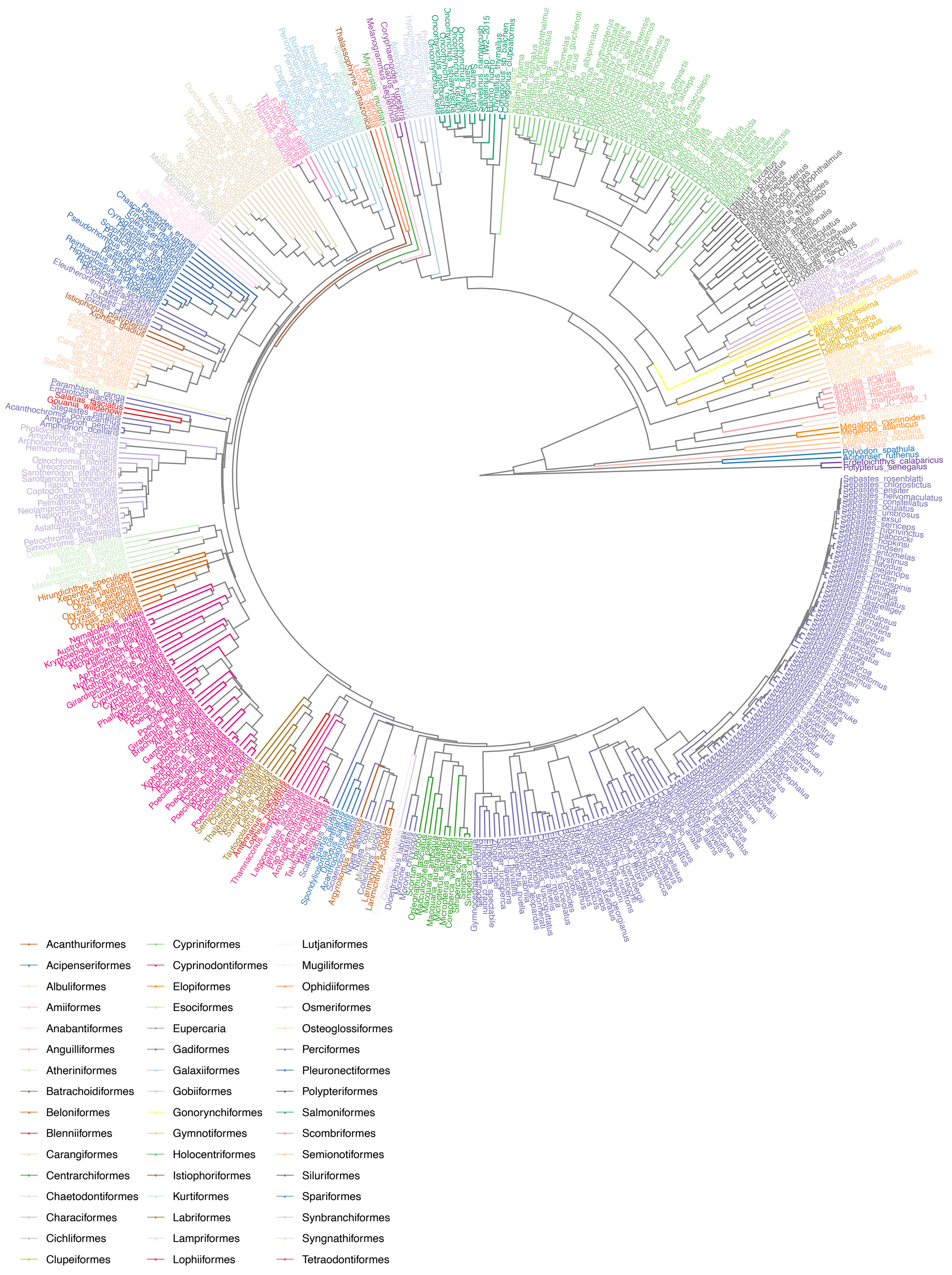

d) Amphibia

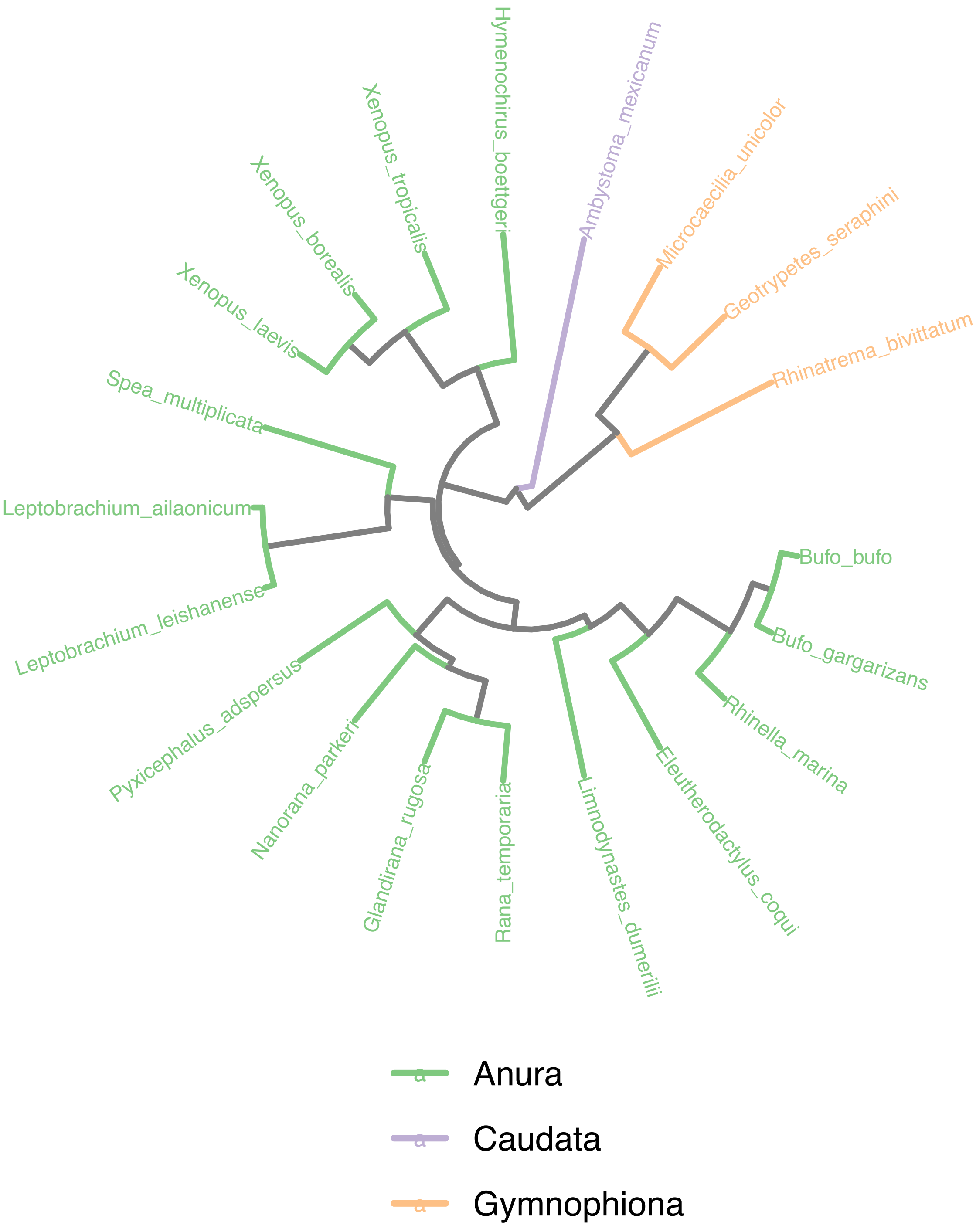
Hymenochirus_boettgeri
Ambystoma_mexicanum
Xenopus_tropicalis
Microcaecilia_unicolor
Xenopus_borealis
Geotrypetes_seraphini
Xenopus_laevis
Rhinatrema_bivittatum
Spea_multiplicata
Leptobrachium_ailaonicum
Bufo_bufo
Bufo_gargarizans
Leptobrachium_leishanense
Rhinella_marina
Pyxicephalus_adspersus
Nanorana_parkeri
Eleutherodactylus_coqui
Glandirana_rugosa
Rana_temporaria
Limnodynastes_dumerilii
Anura
Caudata
Gymnophiona

e) Mammalia

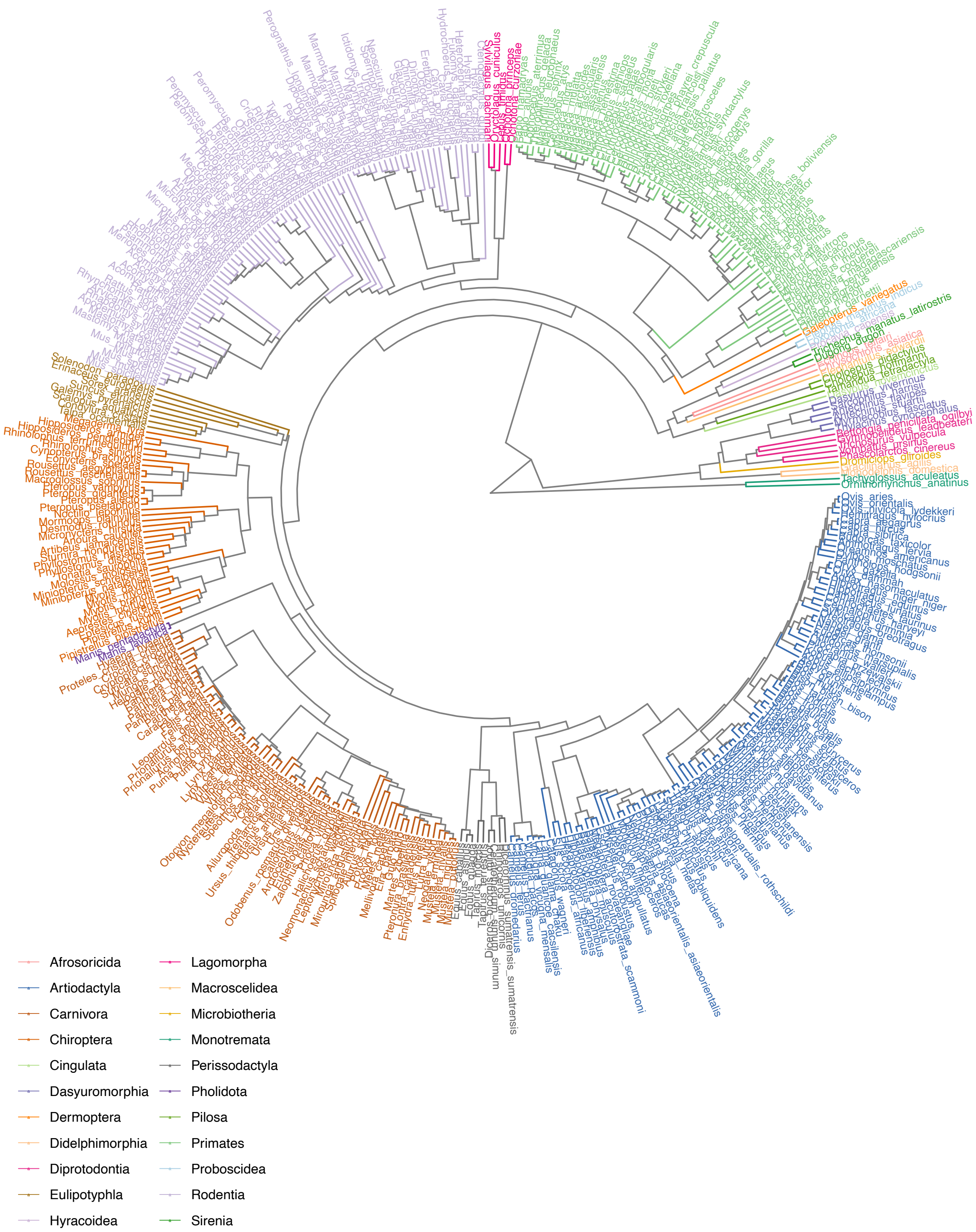
Afrosoricida
Artiodactyla
Carnivora
Chiroptera
Cingulata
Dasyuromorphia
Dermoptera
Didelphimorphia
Diprotodontia
Eulipotyphla
Hyracoidea
Lagomorpha
Macroscelidea
Microbiotheria
Monotremata
Perissodactyla
Pholidota
Pilosa
Primates
Proboscidea
Rodentia
Sirenia

f) Lepidosauria

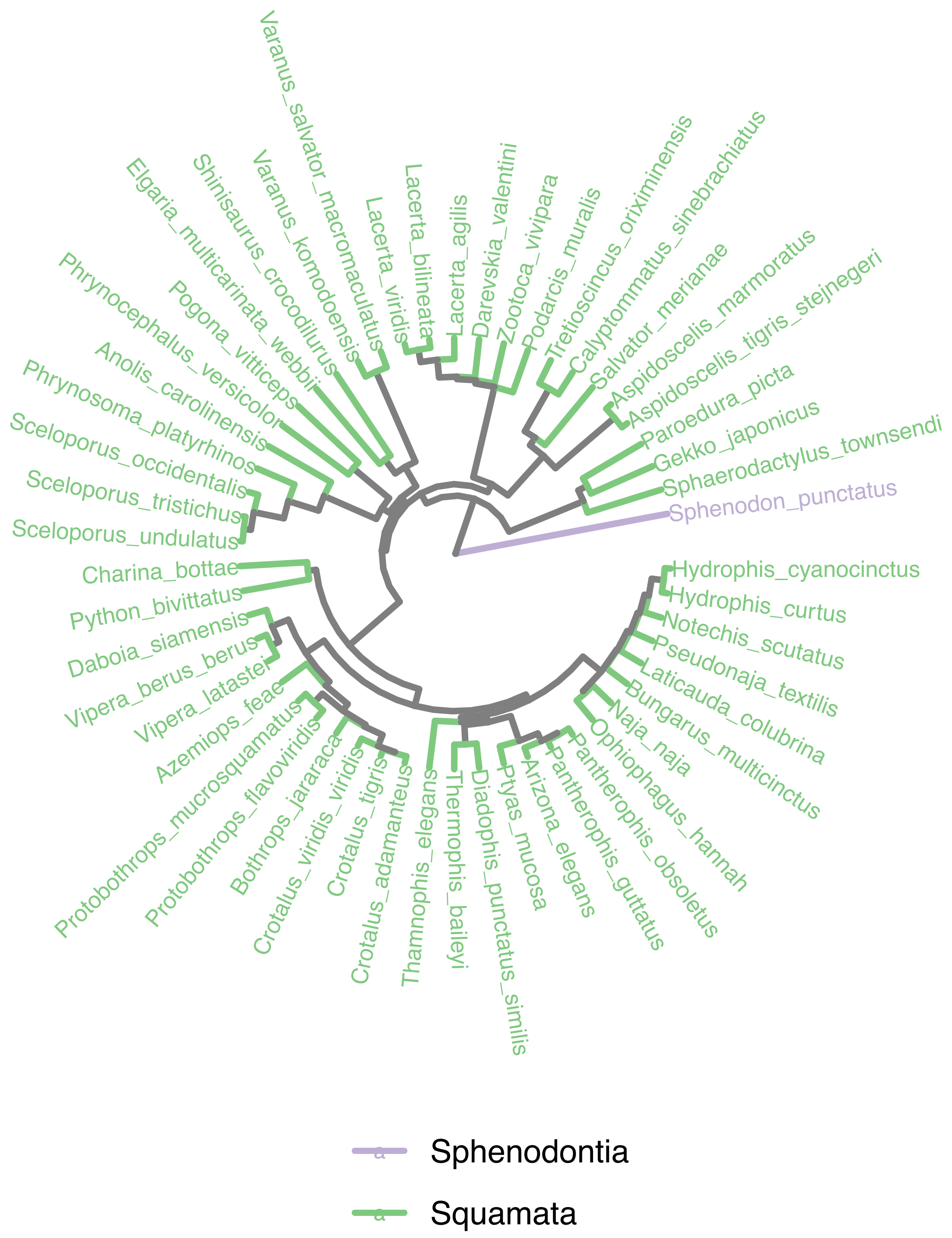

g) Testudines

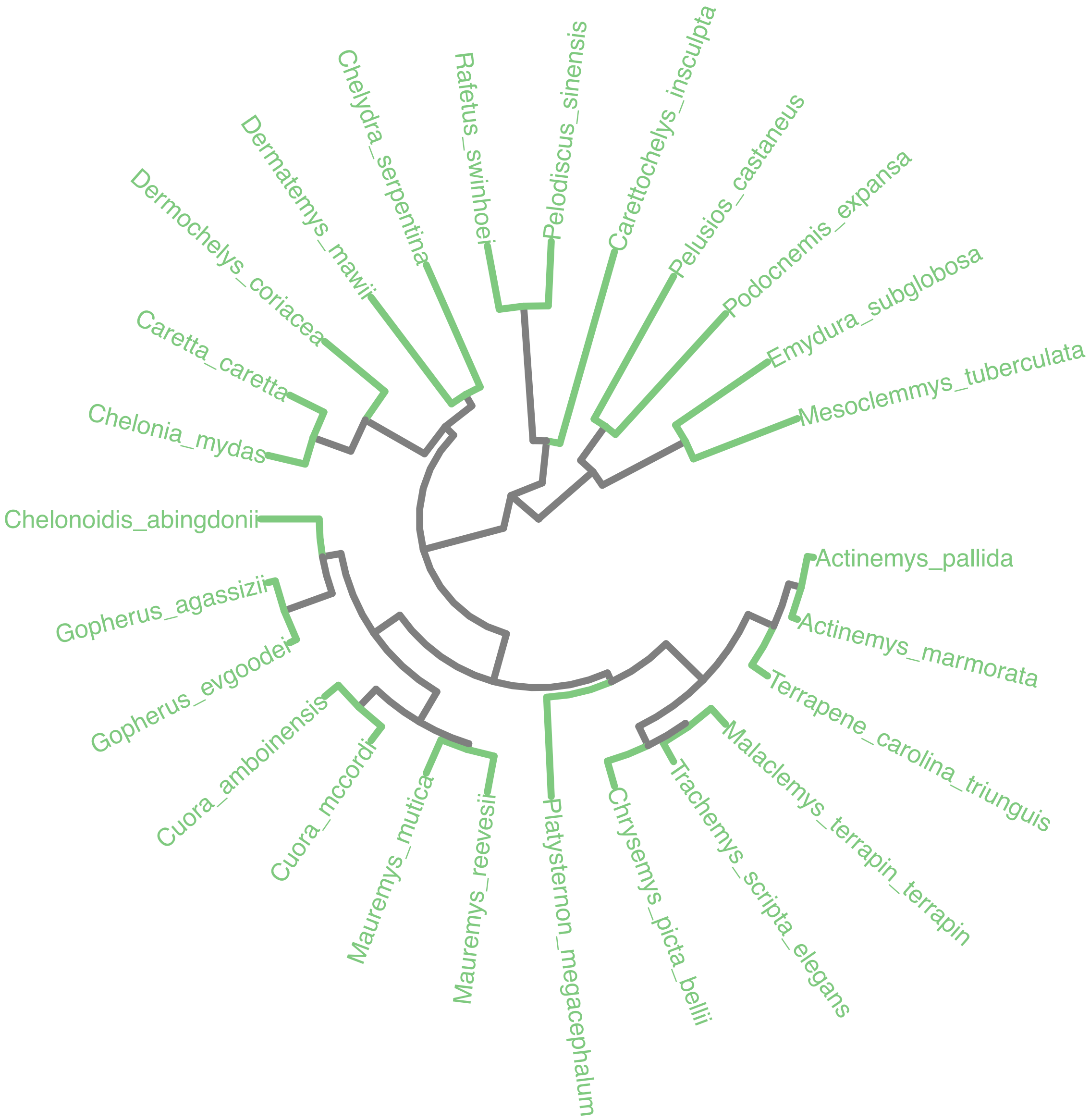

h) Aves (with Crocodilia as outgroup)

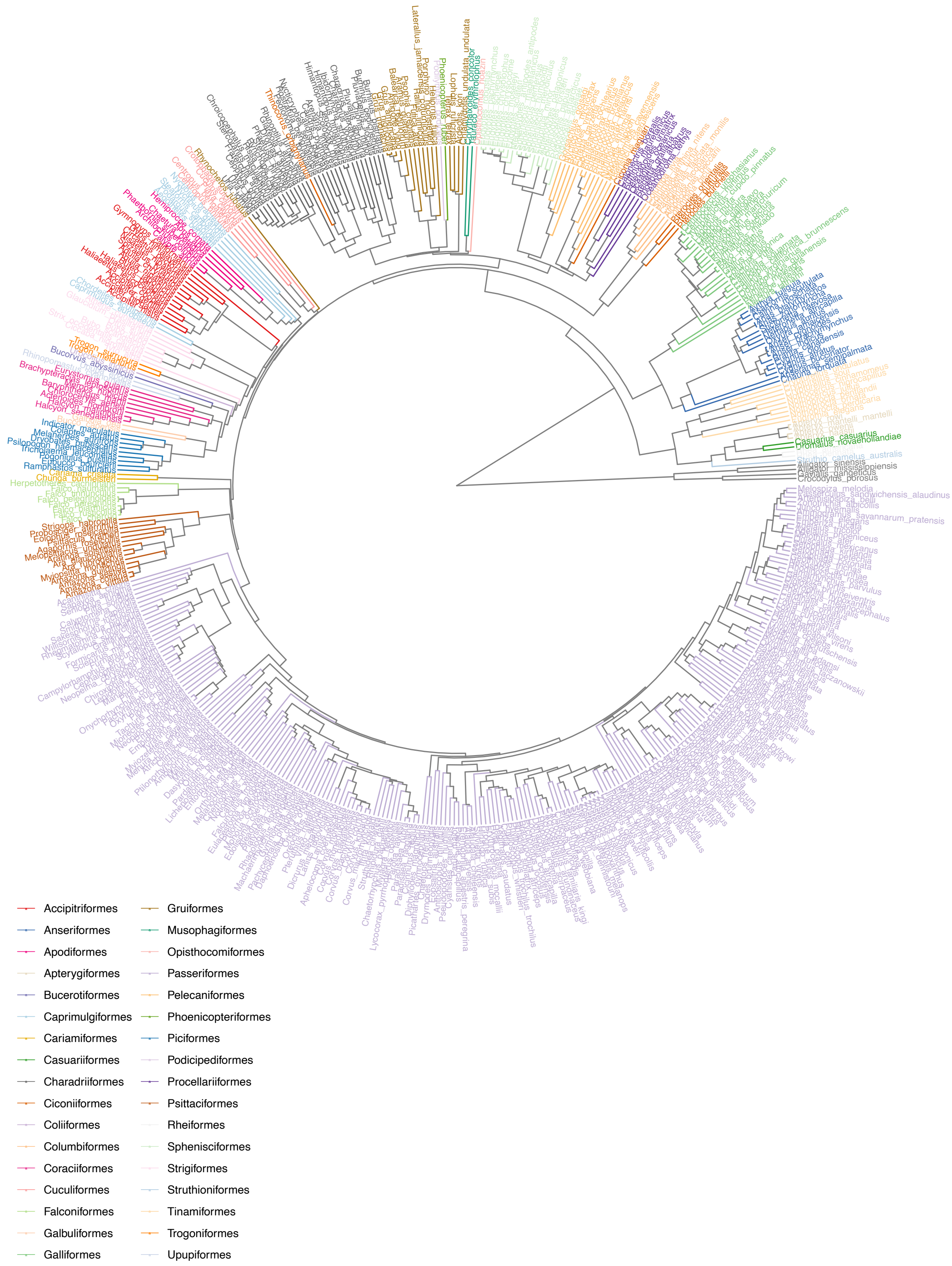

Supplementary Fig. 11 | Phylogenies showing the number of complete chemoreceptors reported. Vertebrate (sub)class phylogenies displaying the number of chemoreceptors (similar to the Fig. 1 in the main text, BUSCO80 dataset). **a**, Agnatha; **b**, Chondrichthyes; **c**, Actinopterygii; **d**, Amphibia; **e**, Mammalia; **f**, Lepidosauria; **g**, Testudines; **h**, Aves and Crocodilia. Terminal branches and species names are colored according to order based on the NCBI taxonomy database. Source data are provided as a Source Data file.

a) Agnatha

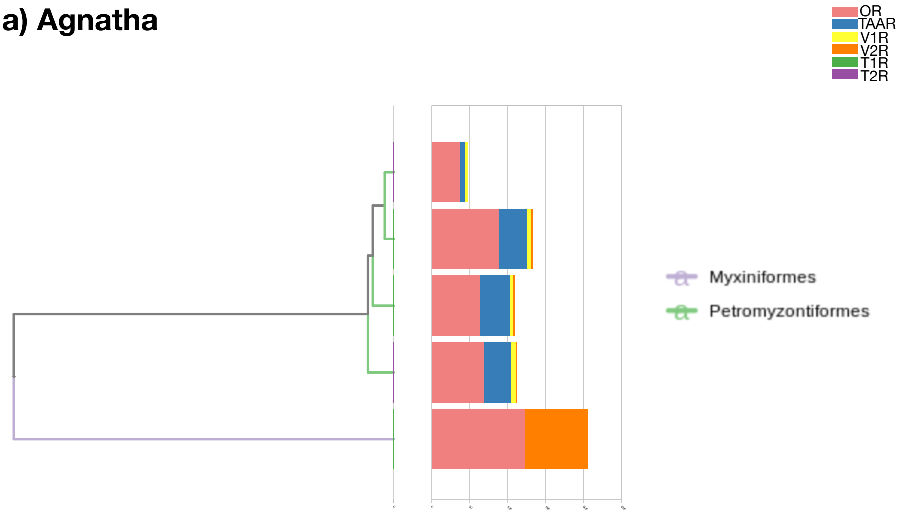

b) Chondrichthyes

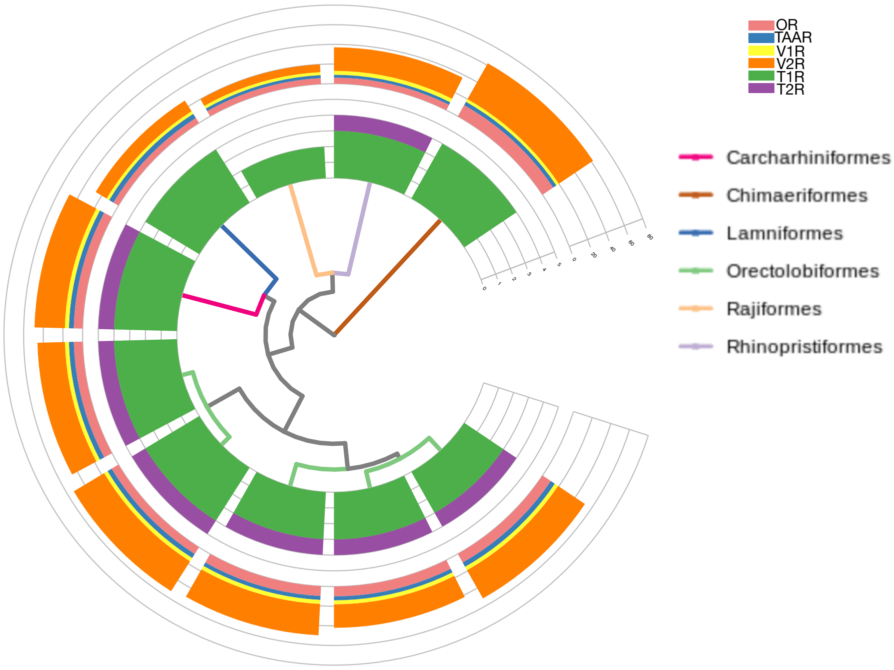

c) Actinopterygii

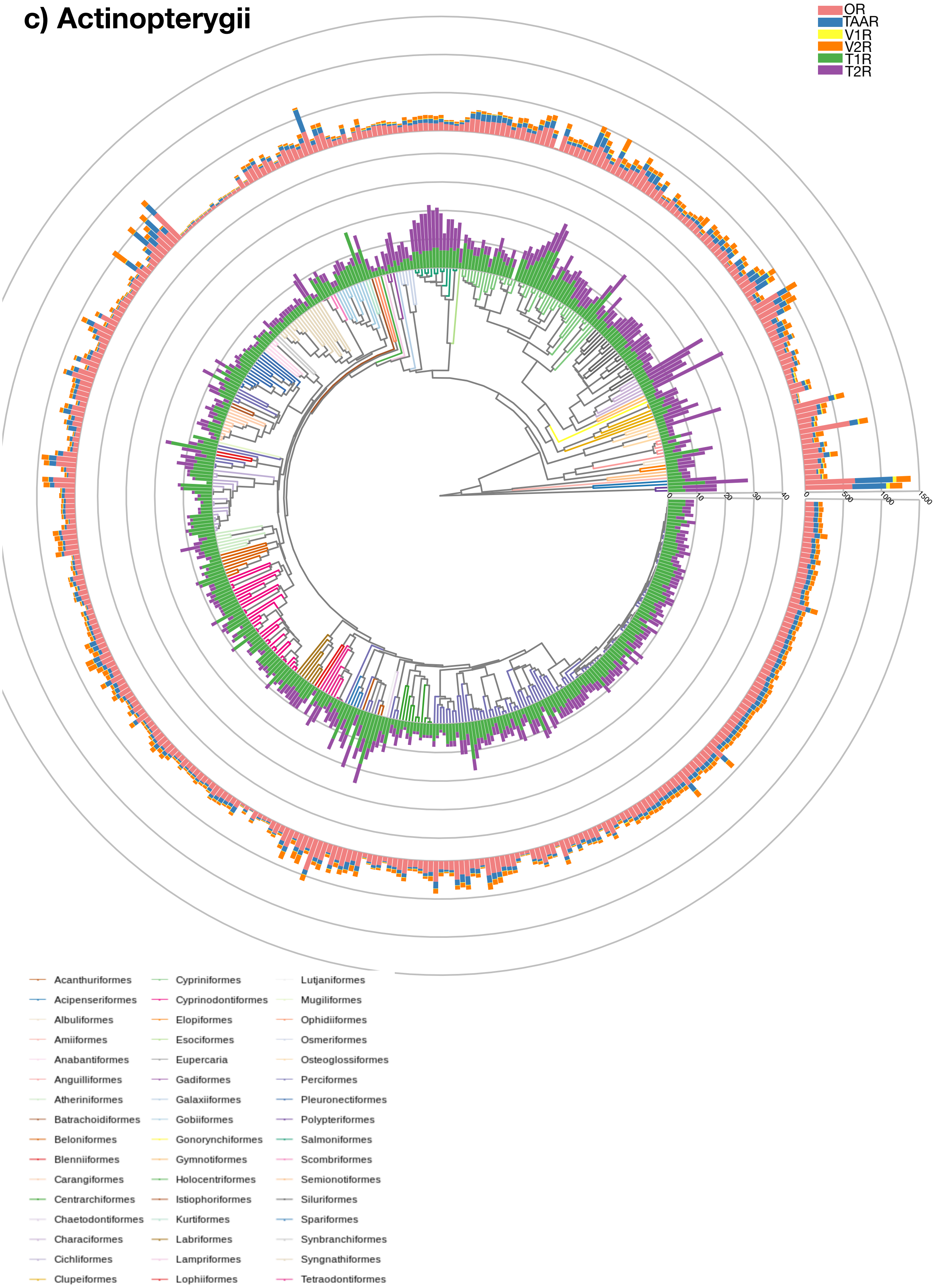

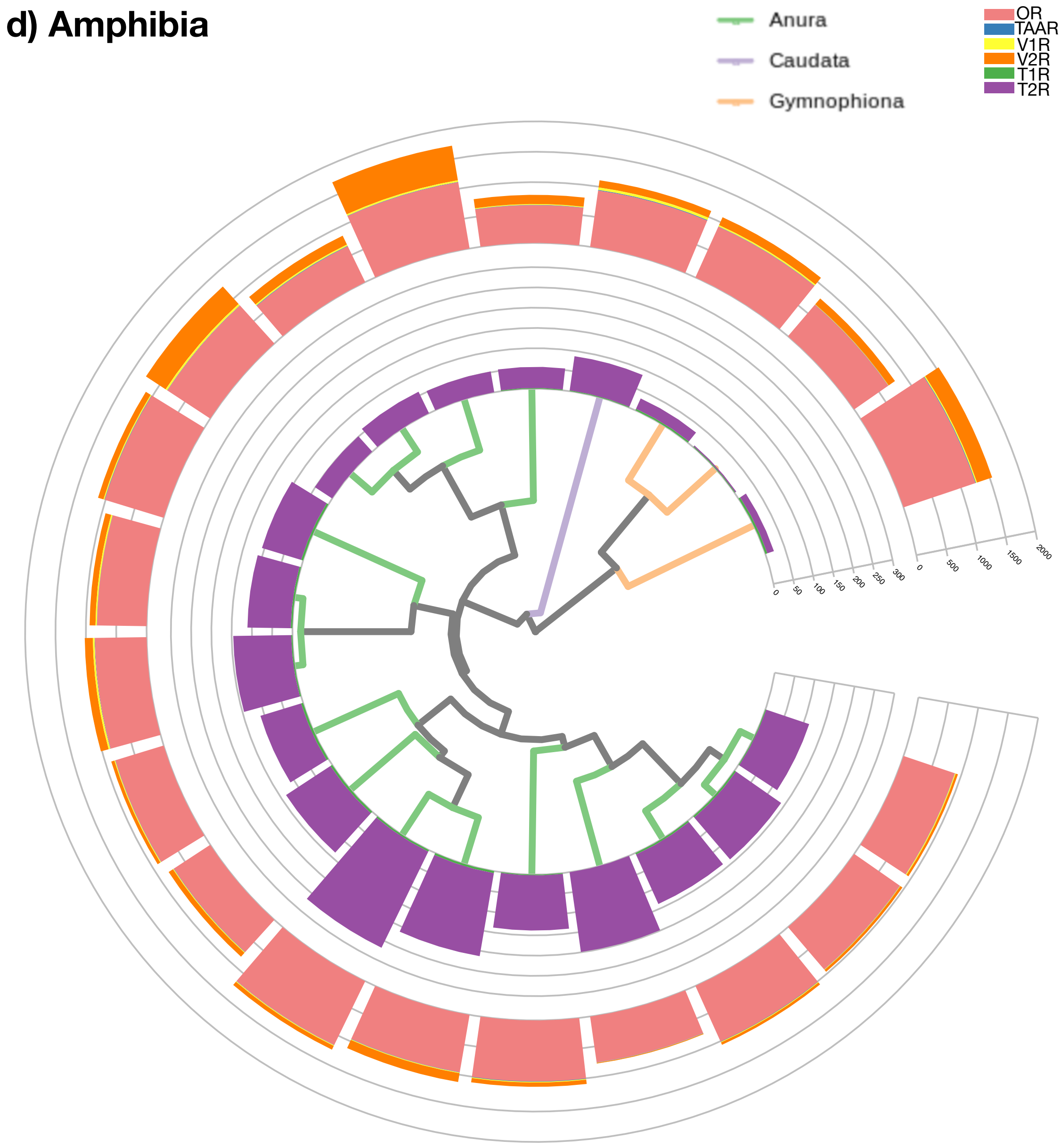
d) Amphibia
Anura
Caudata
Gymnophiona
OR
TAAR
V1R
V2R
T1R
T2R
0
50
100
150
200
250
300
0
500
1000
1500
2000

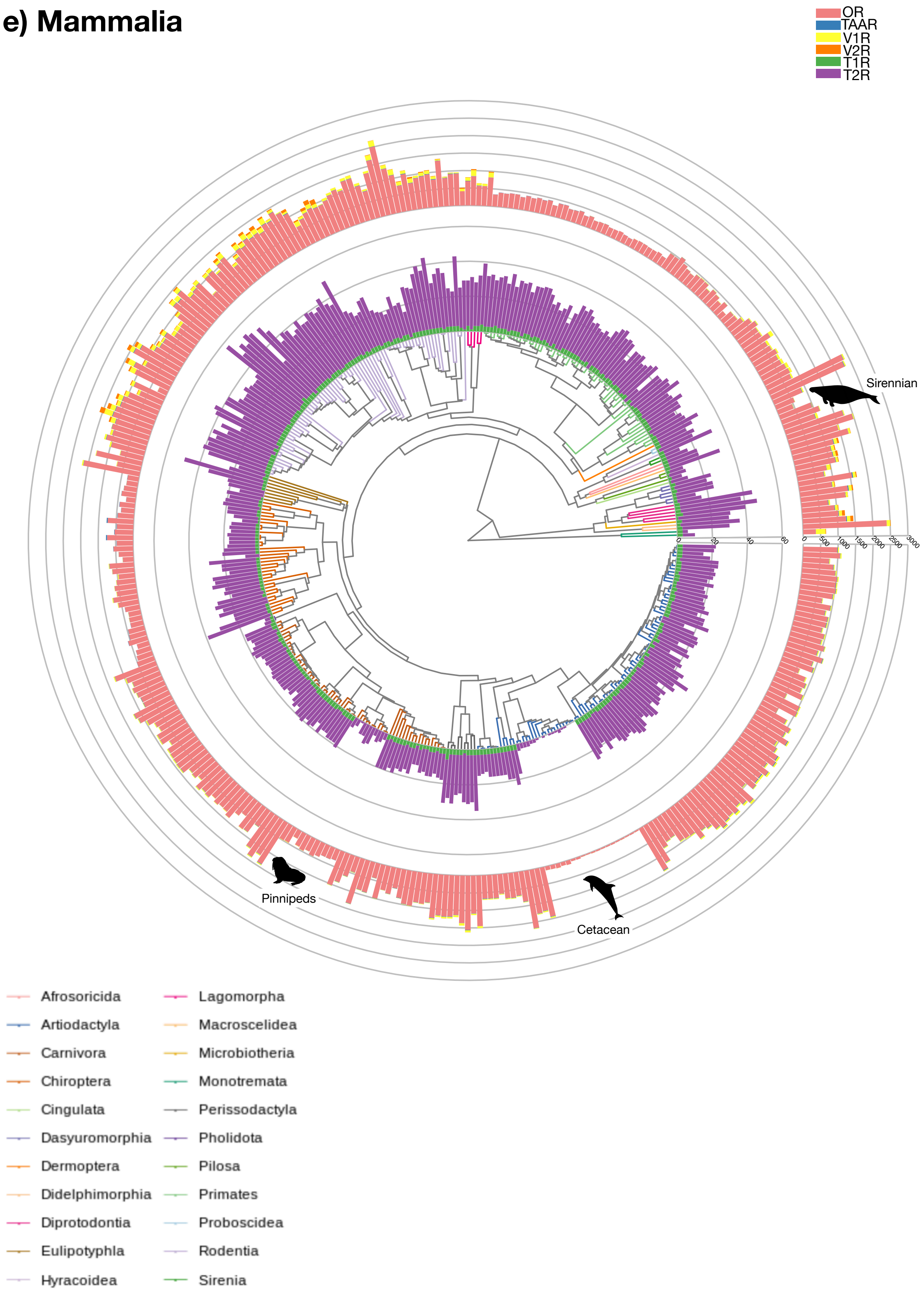
e) Mammalia
OR
TAAR
V1R
V2R
T1R
T2R
Sirennian
Pinnipeds
Cetacean
0
20
40
60
0
500
1000
1500
2000
2500
3000
Afrosoricida
Artiodactyla
Carnivora
Chiroptera
Cingulata
Dasyuromorphia
Dermoptera
Didelphimorphia
Diprotodontia
Eulipotyphla
Hyracoidea
Lagomorpha
Macroscelidea
Microbiotheria
Monotremata
Perissodactyla
Pholidota
Pilosa
Primates
Proboscidea
Rodentia
Sirenia

f) Lepidosauria

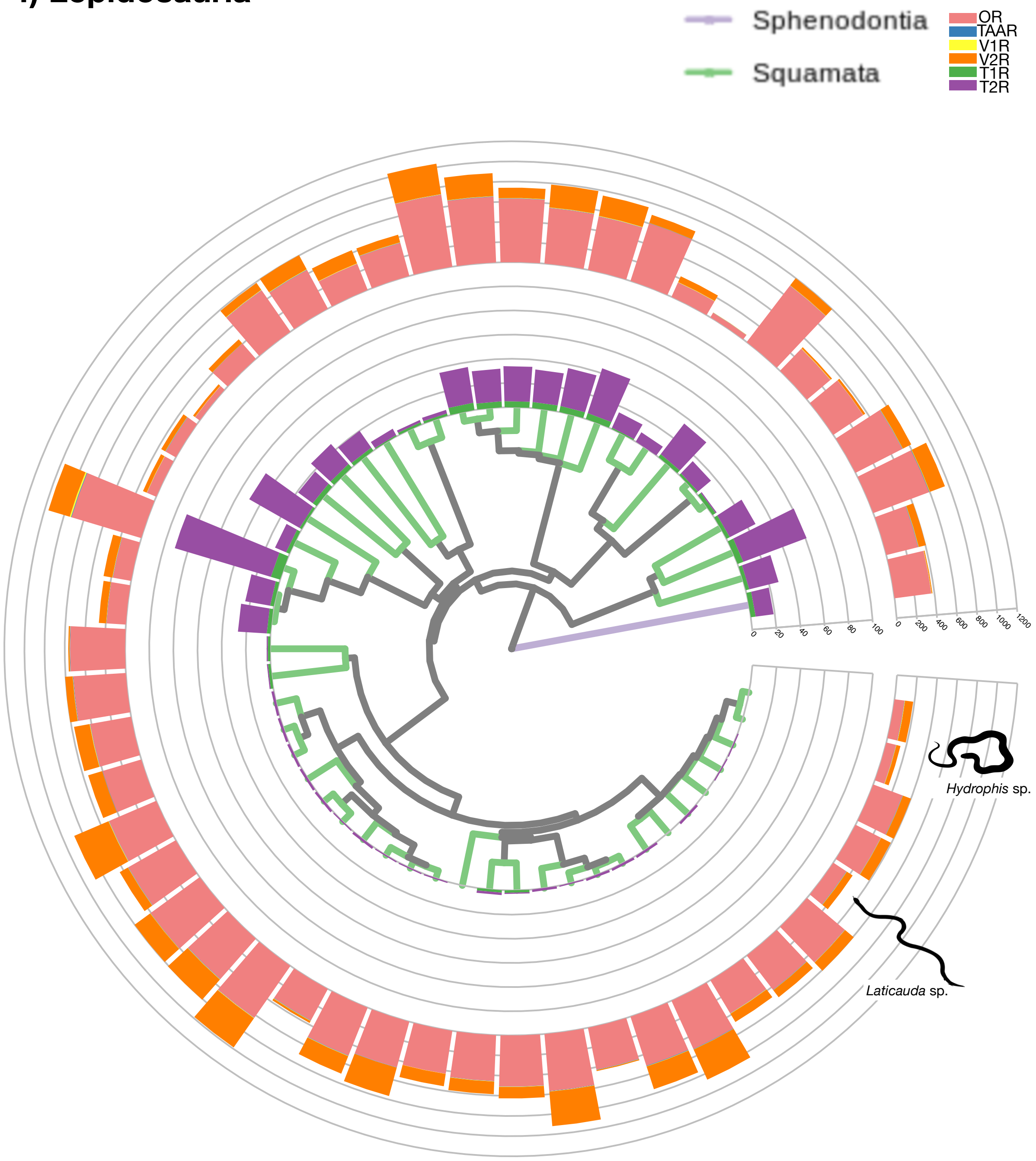

g) Turtles

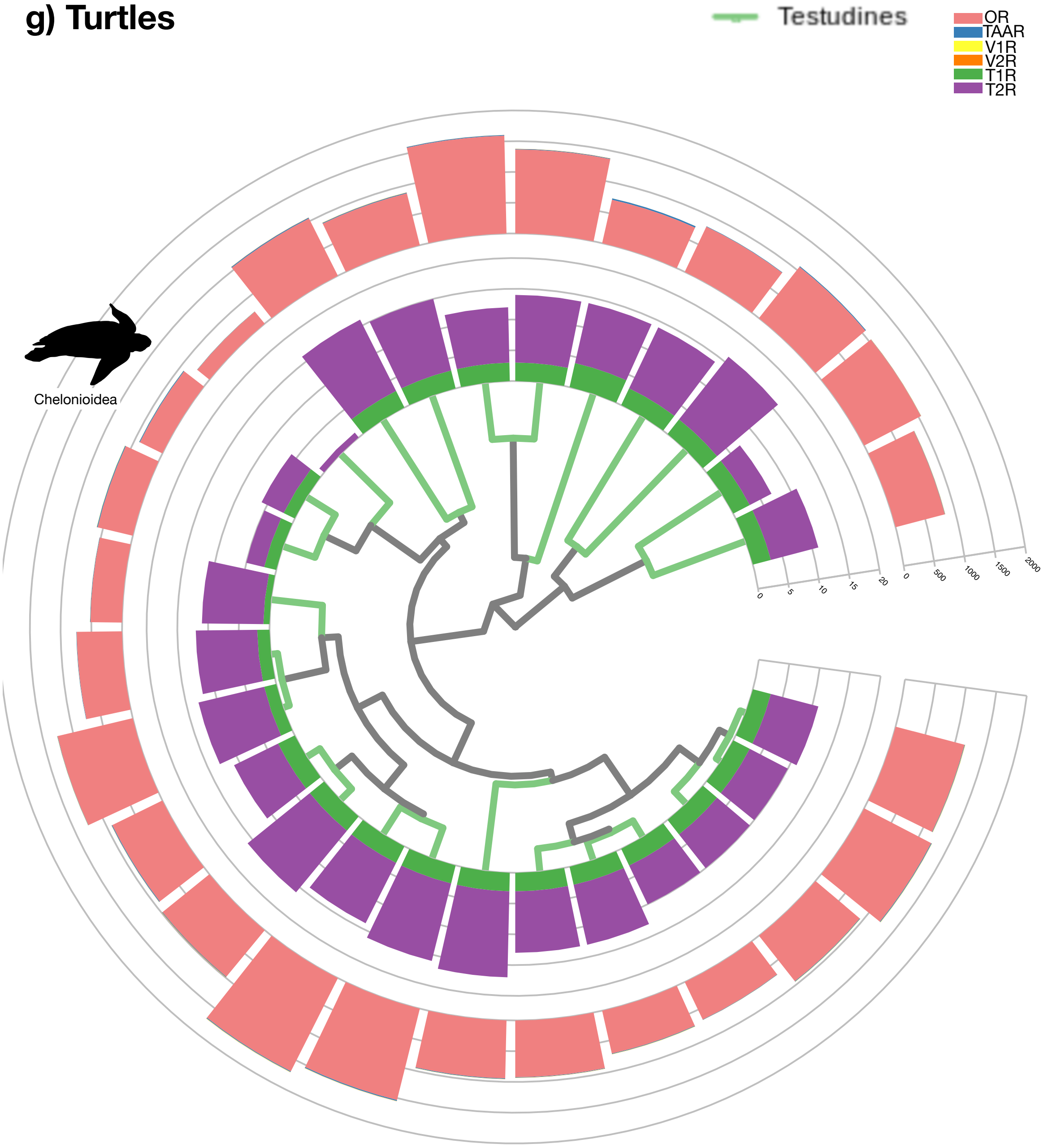

Testudines
OR
TAAR
V1R
V2R
T1R
T2R
Chelonioidea
0
5
10
15
20
0
500
1000
1500
2000

h) Aves (with Crocodilia as outgroup)

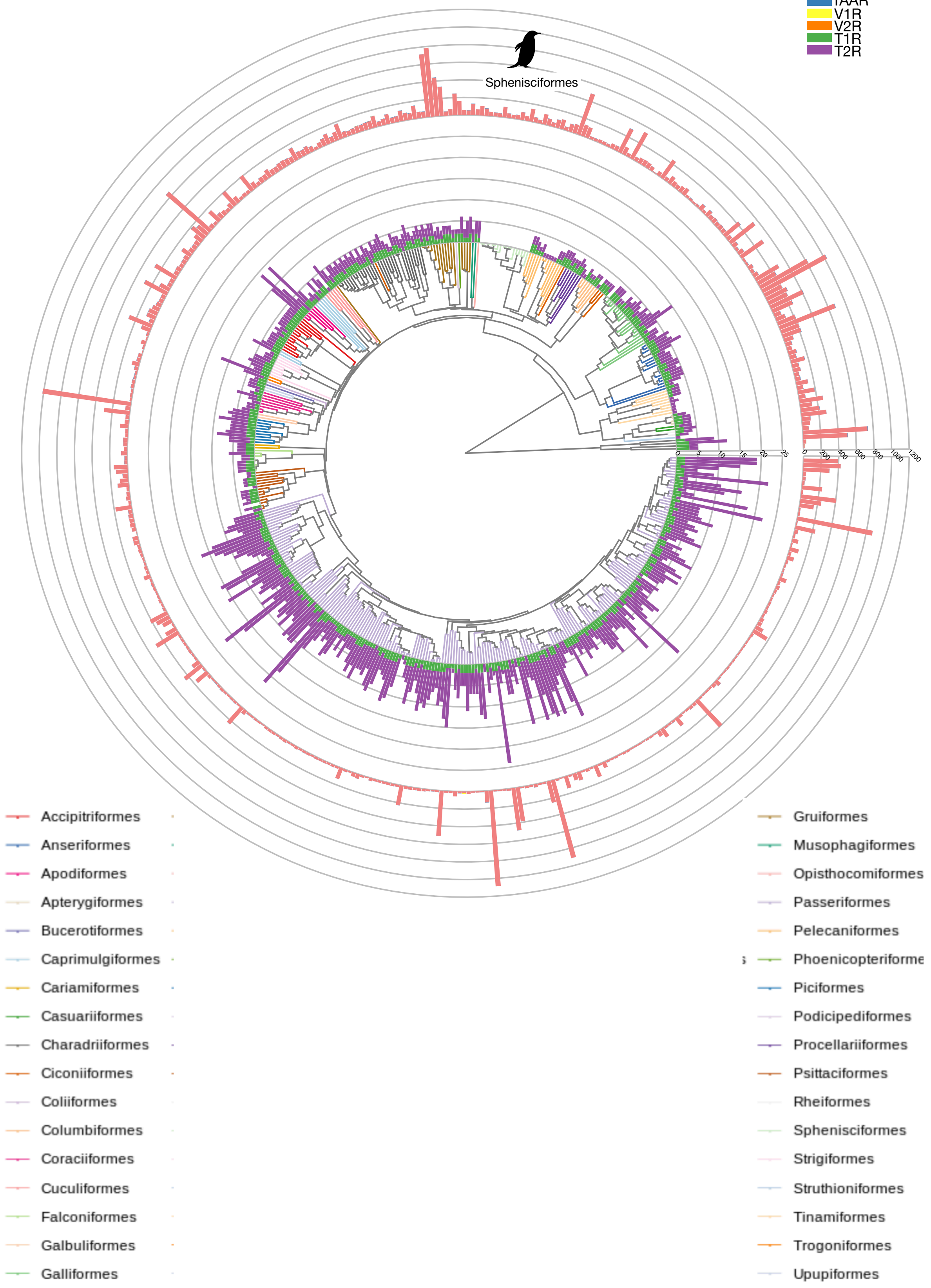

Supplementary Fig. 12 | Phylogenies showing the number of complete *OR* genes. Vertebrate phylogeny and (sub)class phylogenies displaying the number of complete *OR* genes (BUSCO80 dataset). **a**, Vertebrata; **b**, Agnatha; **c**, Chondrichthyes; **d**, Actinopterygii; **e**, Amphibia; **f**, Mammalia; **g**, Lepidosauria; **h**, Testudines; **i**, Aves and Crocodilia. Terminal branches and species names are colored according to (sub)class (**a**) or according to order based on the NCBI taxonomy database (**b-i**). *OR* subclades are indicated. Source data are provided as a Source Data file.

a) Vertebrates

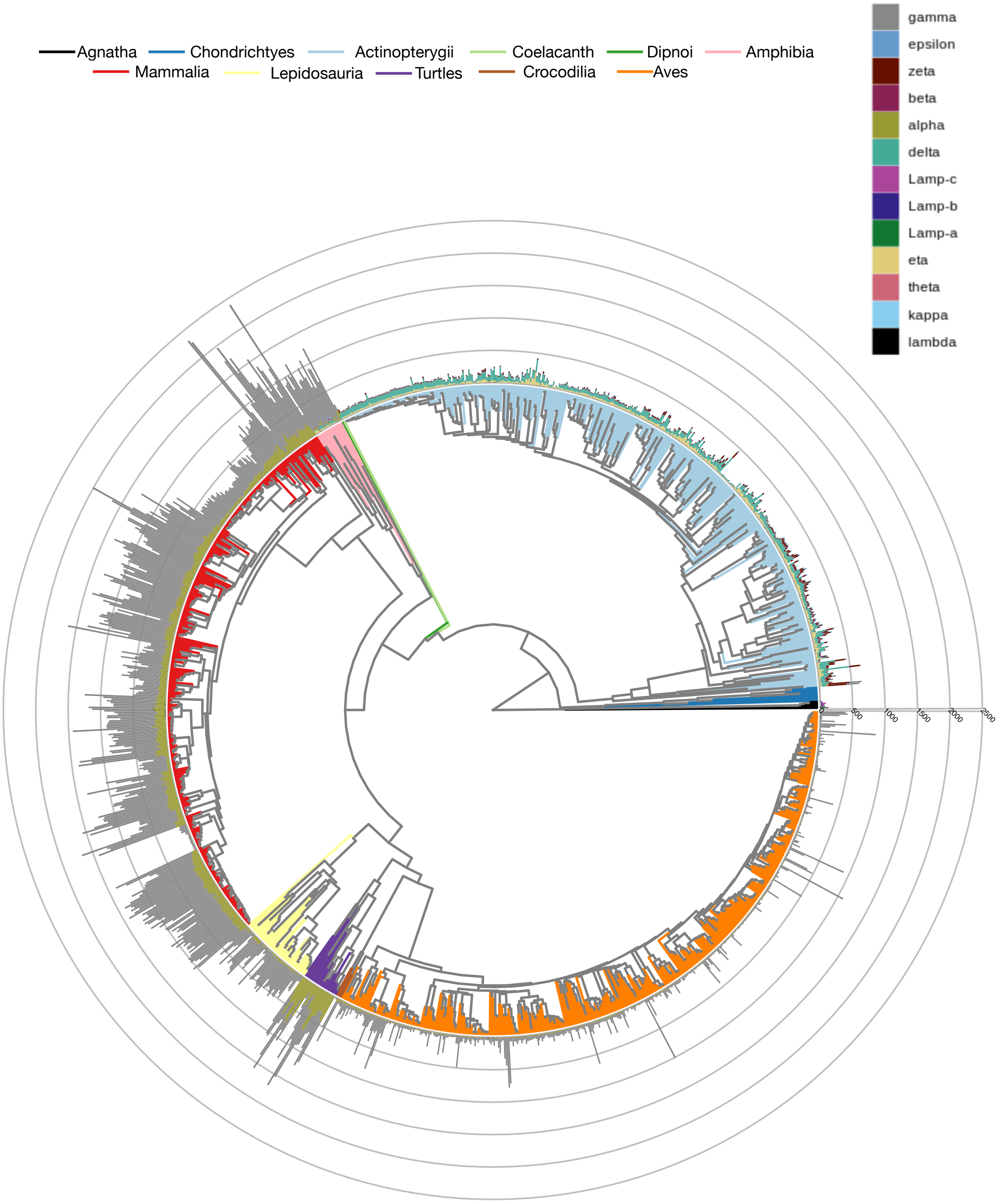

b) Agnatha

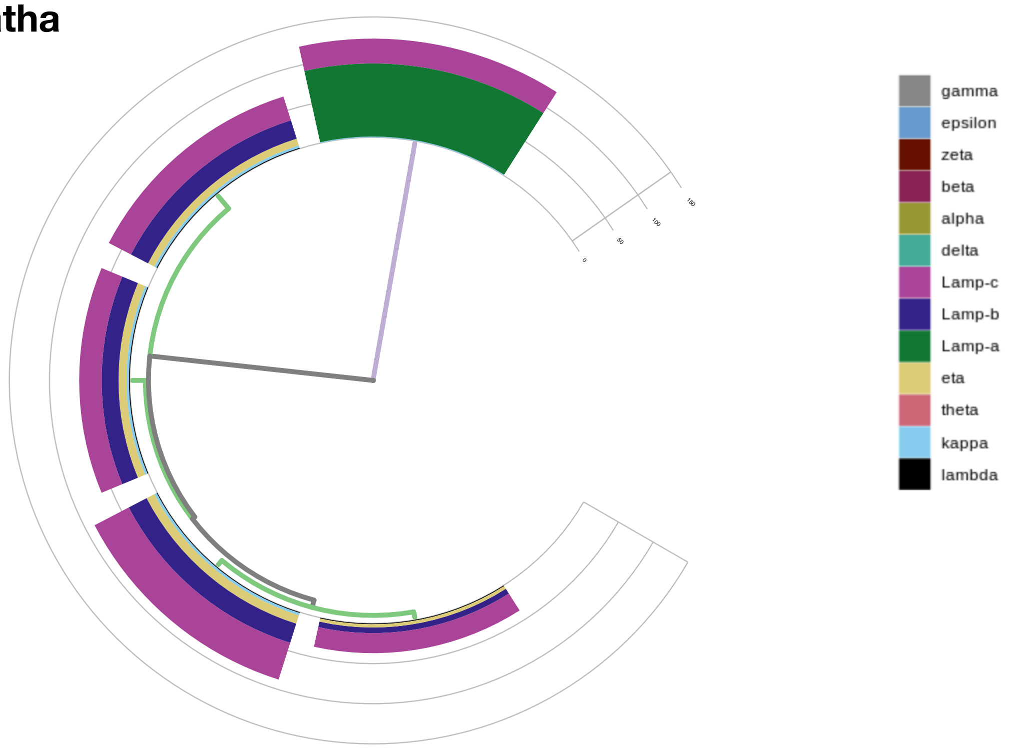

c) Chondrichthyes

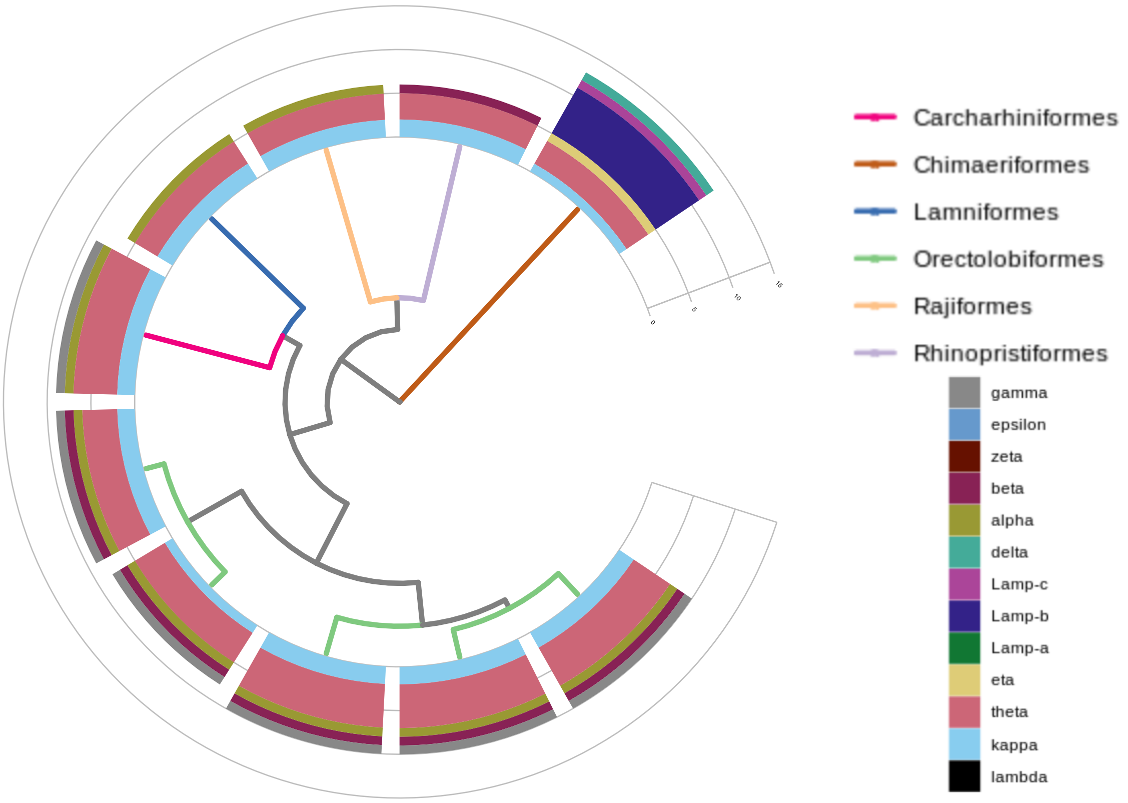

d) Actinopterygii

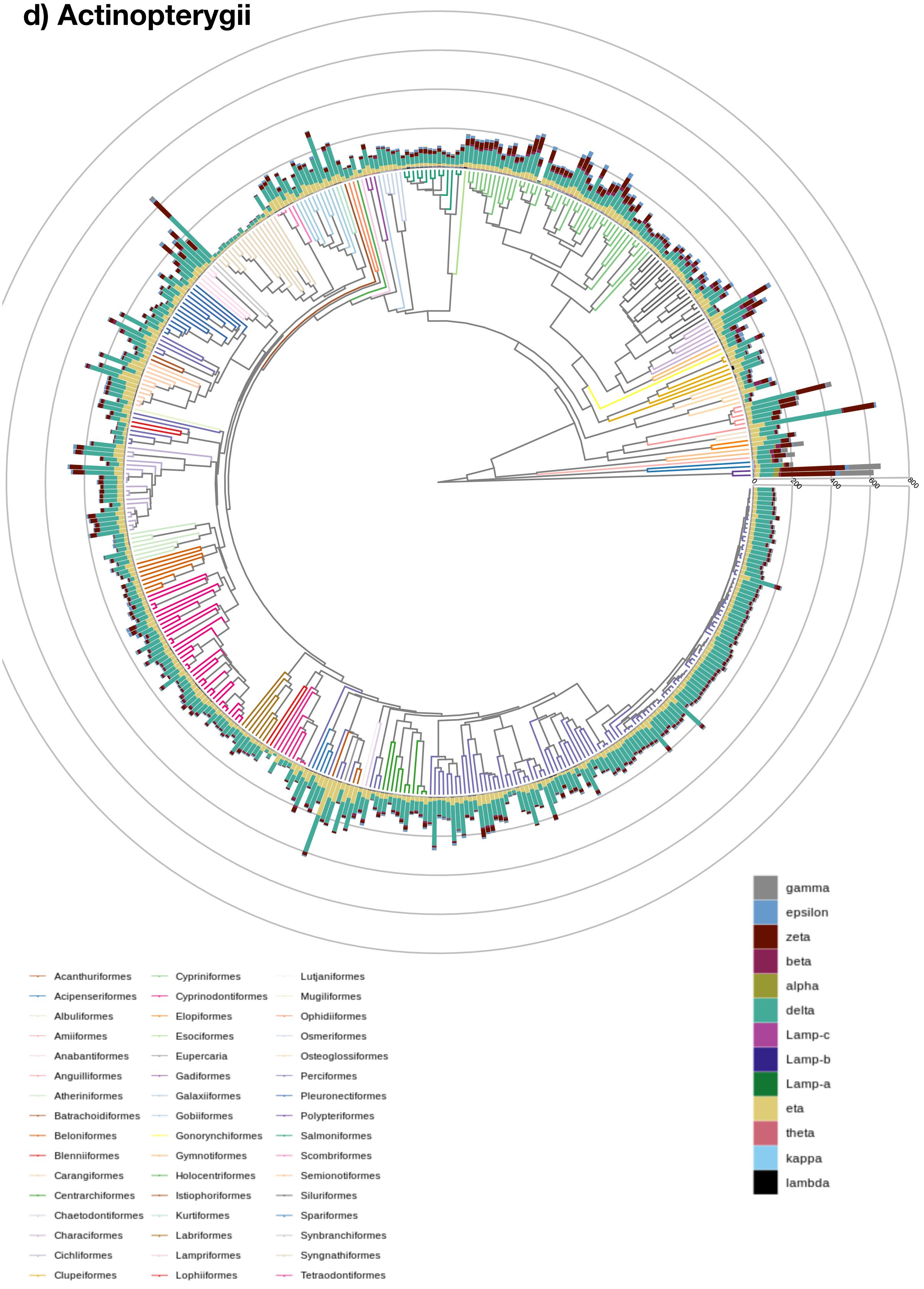

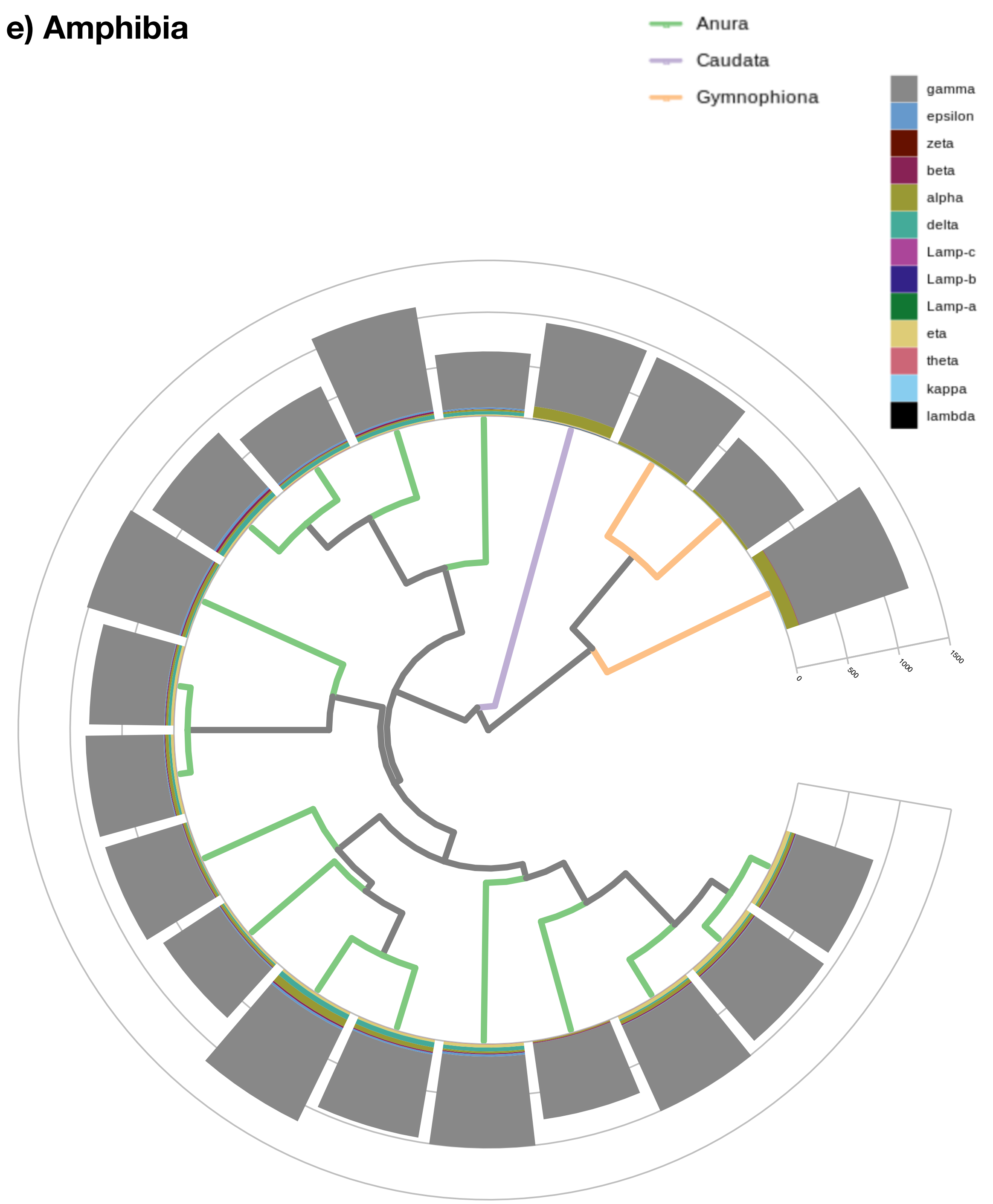

e) Amphibia
Anura
Caudata
Gymnophiona
gamma
epsilon
zeta
beta
alpha
delta
Lamp-c
Lamp-b
Lamp-a
eta
theta
kappa
lambda
0
500
1000
1500

f) Mammalia

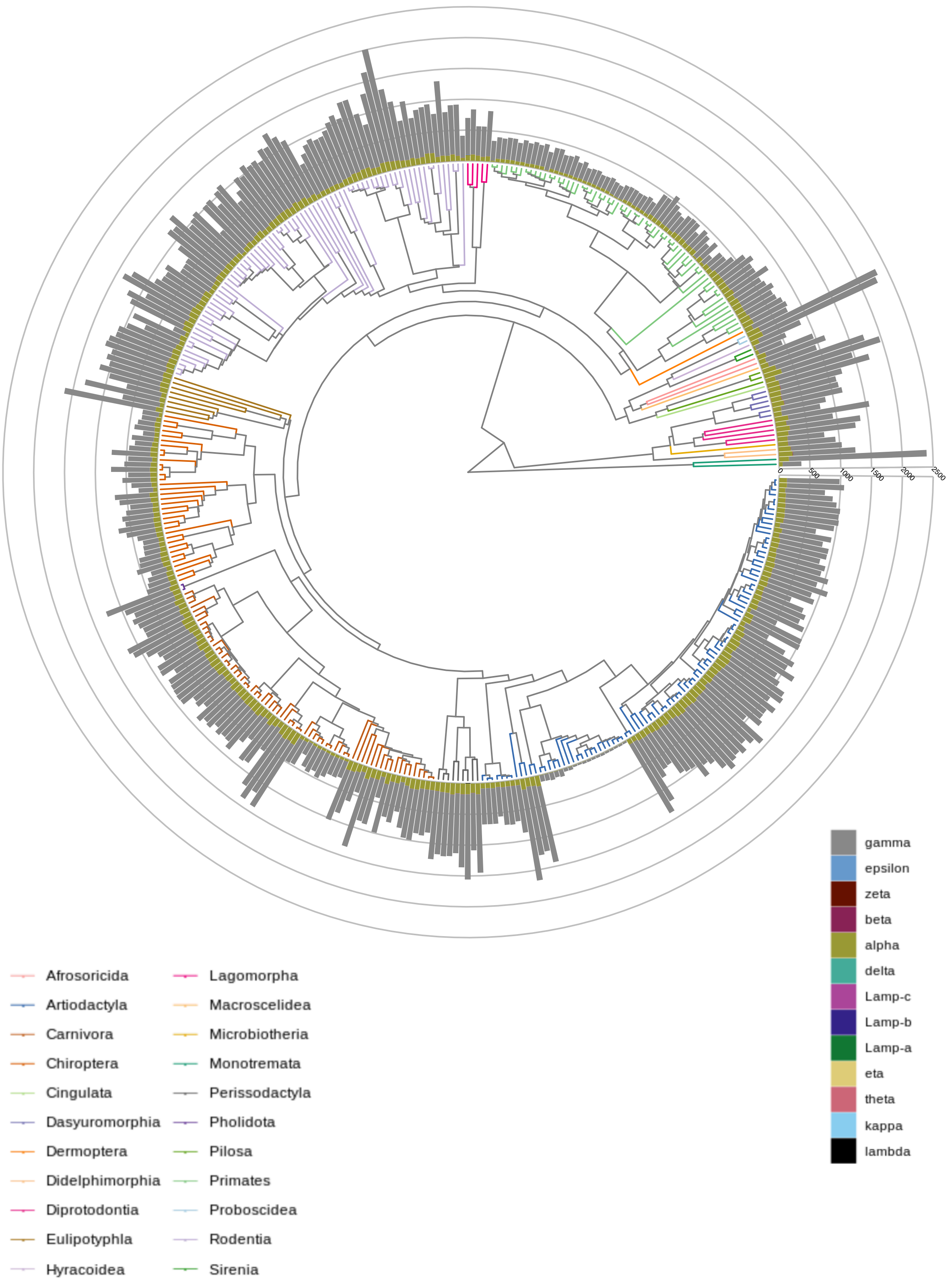
0
500
1000
1500
2000
2500
Afrosoricida
Artiodactyla
Carnivora
Chiroptera
Cingulata
Dasyuromorphia
Dermoptera
Didelphimorphia
Diprotodontia
Eulipotyphla
Hyracoidea
Lagomorpha
Macroscelidea
Microbiotheria
Monotremata
Perissodactyla
Pholidota
Pilosa
Primates
Proboscidea
Rodentia
Sirenia
gamma
epsilon
zeta
beta
alpha
delta
Lamp-c
Lamp-b
Lamp-a
eta
theta
kappa
lambda

g) Lepidosauria

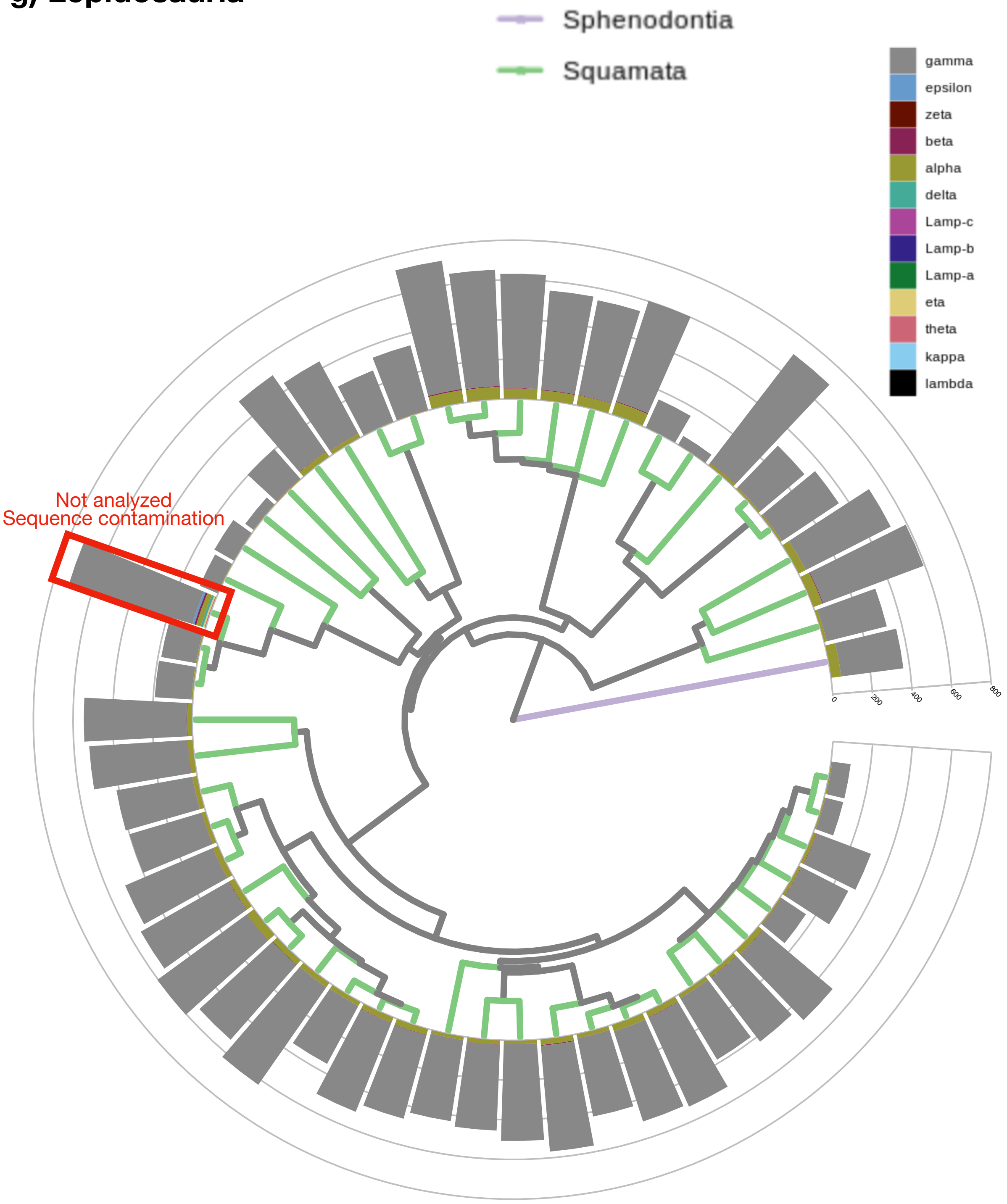

h) Turtles

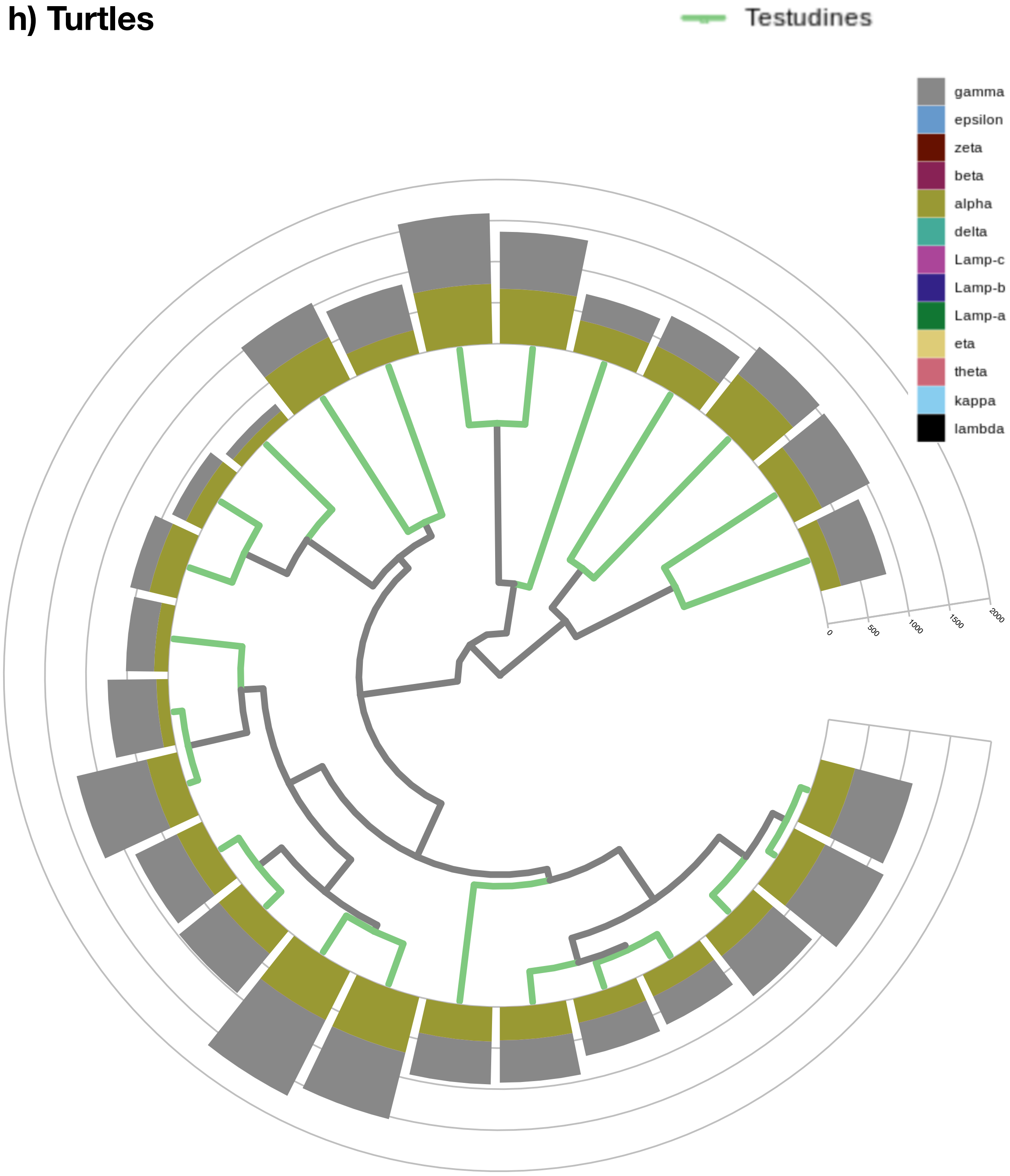

Testudines
gamma
epsilon
zeta
beta
alpha
delta
Lamp-c
Lamp-b
Lamp-a
eta
theta
kappa
lambda
0
500
1000
1500
2000

i) Aves (with Crocodilia as outgroup)

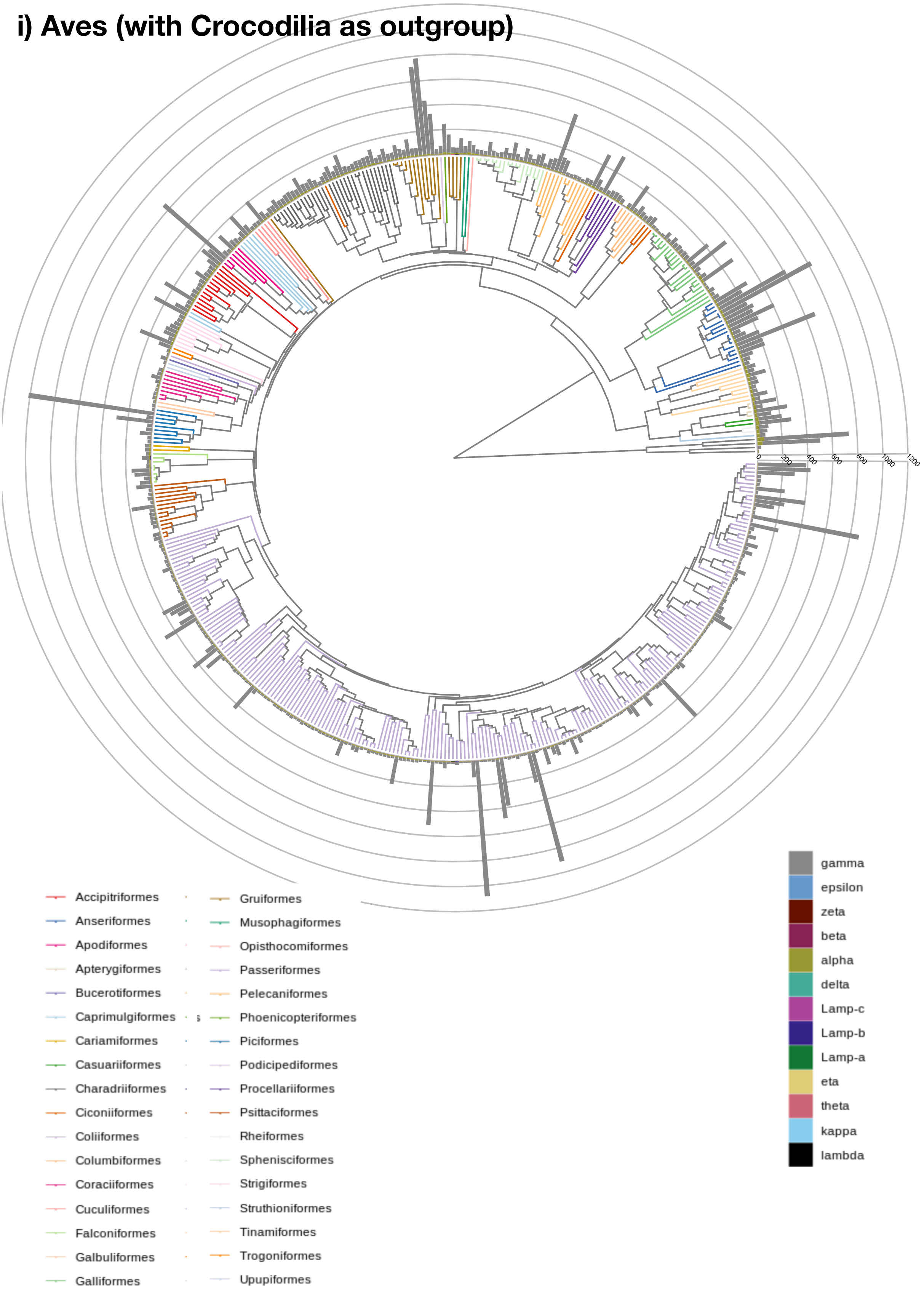

Supplementary Fig. 13 | Vertebrate *OR* gene tree. a, Near maximum likelihood phylogeny of *OR* genes in vertebrates with branches colored according to the vertebrate (sub)class. **b**, Clade tree generated from (**a**) with local support values indicated at each node (according to a Shimodaira-Hasegawa test). All clades defined by Niimura[21] in his earlier study of vertebrate *OR* genes were retrieved as monophyletic. We thus kept the previously proposed clade nomenclature, with *Type 1 OR genes* being composed of δ, α, β, ζ, ε and γ genes, and *Type 2 OR* genes being composed of λ, κ, θ and η genes. Three additional *Type 2 OR* clades were found in this study: clade A, which is restricted to agnathans; clade B, which is restricted to agnathans, the Australian ghostshark (*Callorhinchus milii*) and the coelacanth; and clade C, which is restricted to agnathans and the ghostshark. The tree in newick version, as well as all sequences and the alignment file can be found in FigShare. Sub-trees of each vertebrate (sub)class with the number of OR genes are shown in Supplementary Fig. 12. Maximum likelihood phylogeny of *OR* genes extracted from one species per vertebrate (sub)class is shown in Supplementary Figs. 14-15.

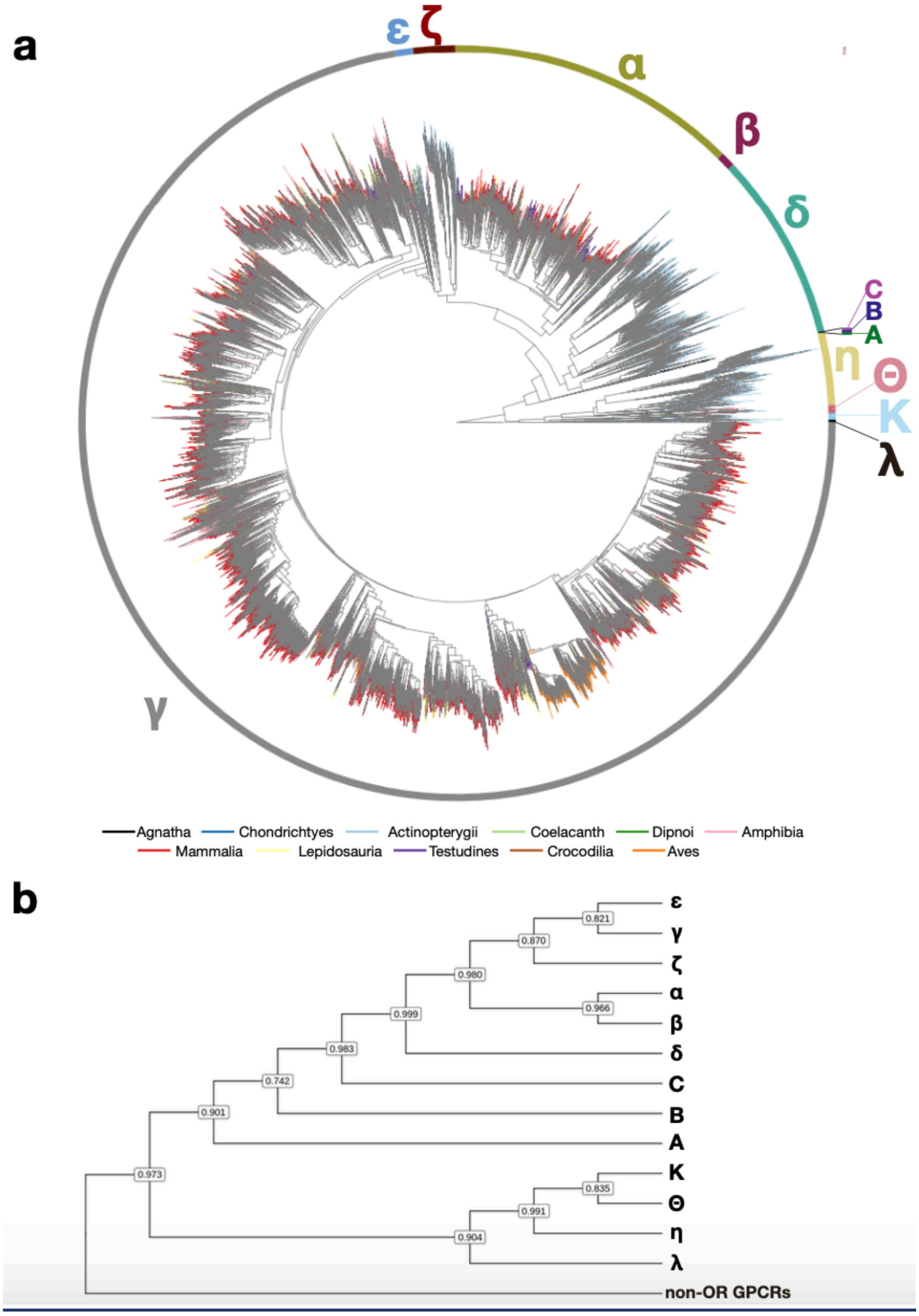

Supplementary Fig. 14 | Maximum likelihood phylogeny of *OR* genes. Maximum likelihood phylogeny of OR genes extracted from one representative species per vertebrate subclass. Branches are colored according to the vertebrate (sub)class. *OR* subclades are indicated. The tree was computed using the optimal model found by ModelFinder (JTT+F+R6). The robustness of the nodes was evaluated with 1,000 ultrafast bootstraps and these bootstrap values are reported for each node in Supplementary Fig. 15. All the clades retrieved using the near-ML method were retrieved as monophyletic, and the exact same topology was retrieved.

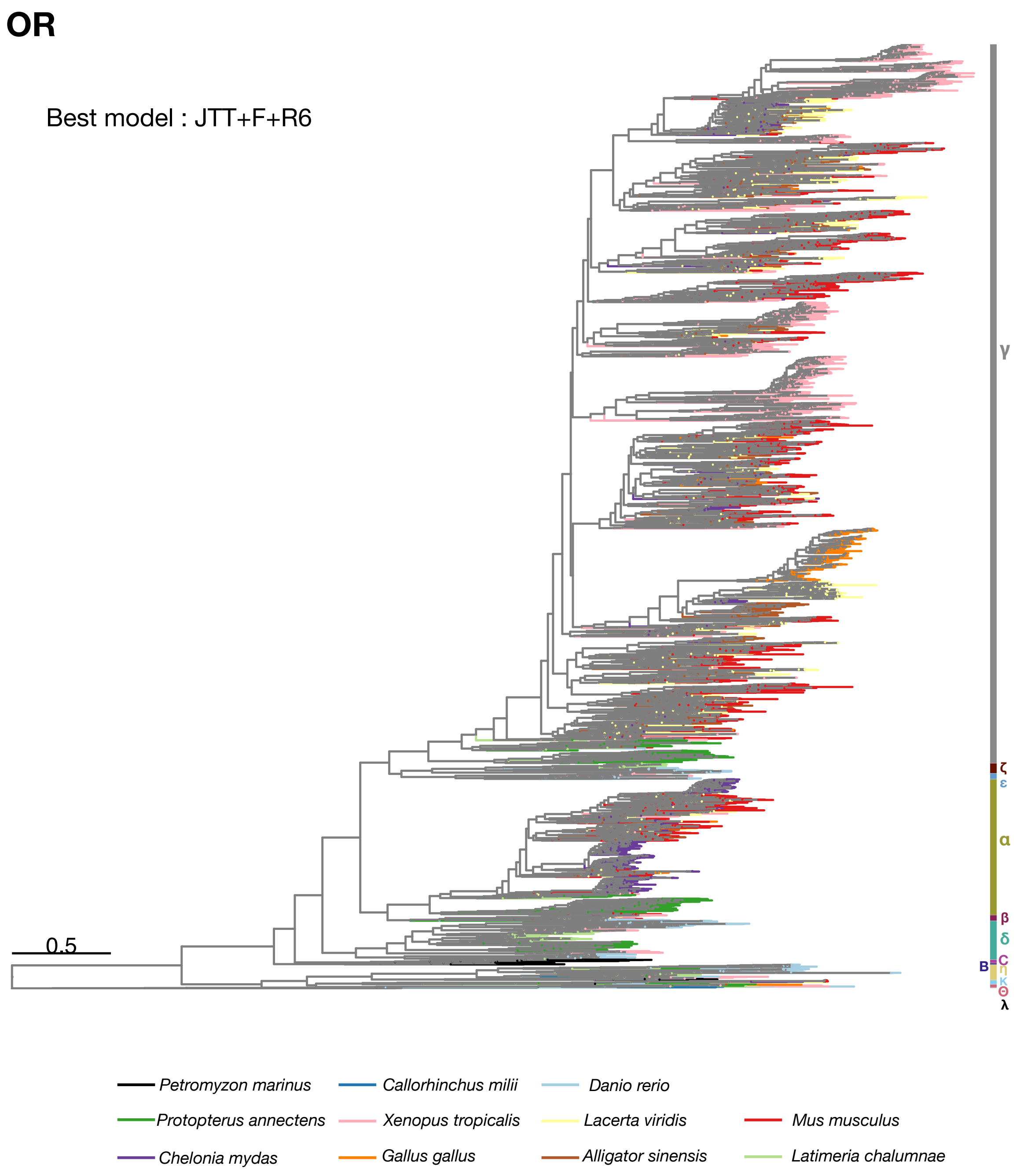

Supplementary Fig. 15 | Maximum likelihood phylogeny of *OR* genes. Same maximum likelihood phylogeny of *OR* genes as in Supplementary Fig. 14 but with bootstrap values indicated at each node.

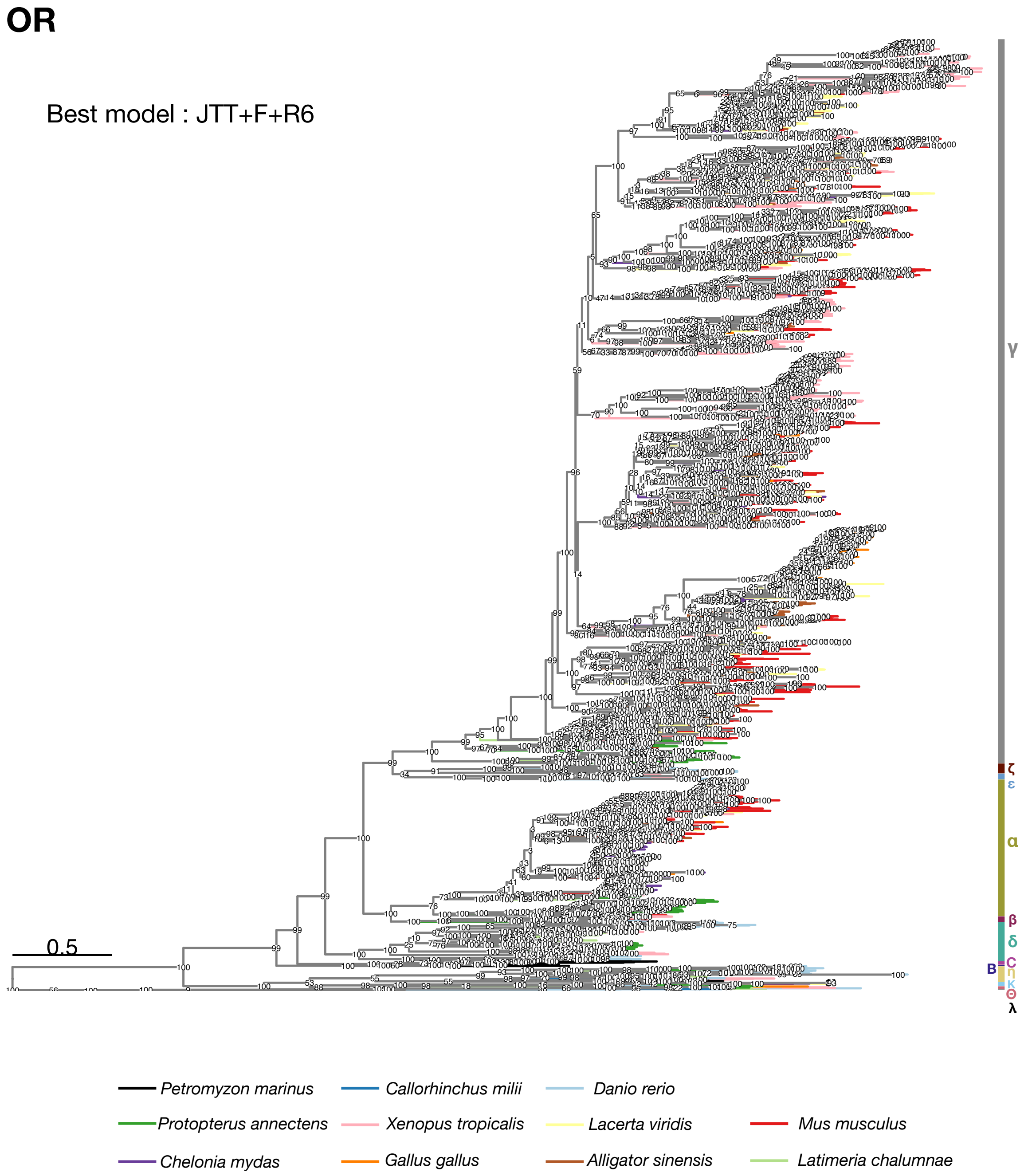

Supplementary Fig. 16 | Evolutionary history of *OR* and *TAAR* genes in vertebrates. a, Schematic of the OR gene tree (top) indicating the subclades within the OR gene family in vertebrates. Gene duplication events leading to subclade divergence are indicated as triangles on the respective branches in the schematic vertebrate phylogeny (left), subclade losses are indicated with the cross symbol below the branches. The numbers refer to nodes in the gene tree. Circles on the right indicate the presence of a particular subclade in a given evolutionary lineage, whereby the size of each circle corresponds to the mean number of complete OR genes per species in the respective subclade and lineage. The *OR* gene tree is shown in Supplementary Fig. 13. **b**, Schematic of the *TAAR* gene tree (top) indicating the subclades within the TAAR gene family in vertebrates. Gene duplication events are indicated as triangles on the respective branches in the schematic vertebrate phylogeny (left), subclade losses are indicated with the cross symbol. The numbers refer to nodes in the gene tree. Circles on the right indicate the presence of a particular subclade in a given evolutionary lineage, whereby the size of each circle corresponds to the mean number of complete *TAAR* genes per species in the respective subclade and lineage. The *TAAR* gene tree is shown in Supplementary Fig. 17. Subclades present in non-teleost fish but absent in teleosts (α-subclade for *OR*s and B3-clade for *TAAR*s) are marked with an "*".Source data are provided as a Source Data file.

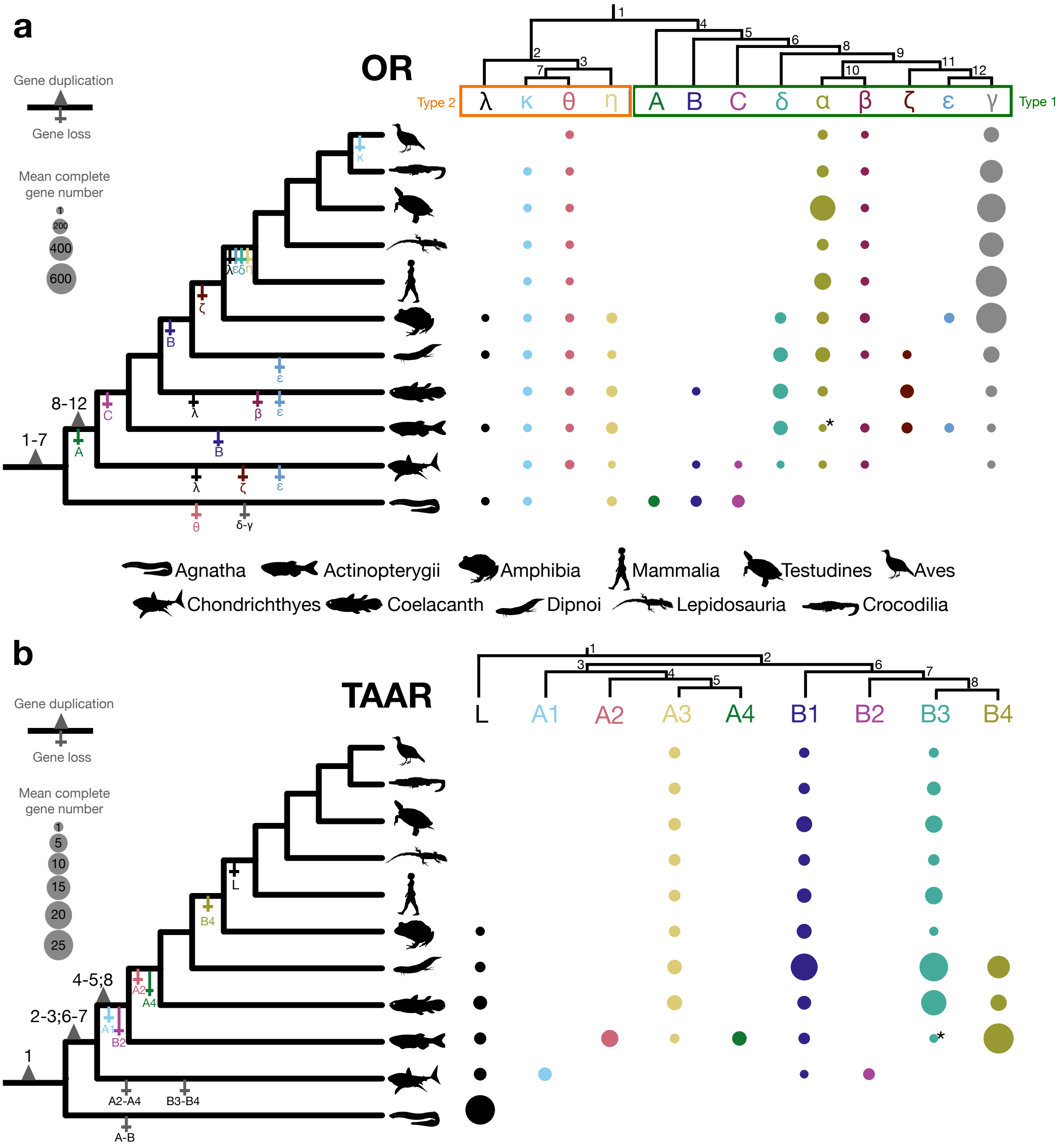

Supplementary Fig. 17 | Vertebrate *TAAR* gene tree. a, Near maximum likelihood phylogeny of *TAAR* genes in vertebrates with branches colored according to the vertebrate (sub)class. **b**, Clade tree generated from (**a**) with local support values indicated at each node (according to a Shimodaira-Hasegawa test). The gene tree topology was similar to the one presented by Dieris et al.[22]. However, we renamed the *TAAR* gene clades, as the former “class II” clade was not monophyletic according to our analyses. Thus, class I genes are split into four clades, named A1 to A4, while “class II” genes are split into three clades, named B1 to B3. Note that former “class III” clade was retrieved as monophyletic, but we renamed it as B4 in coherence with our new nomenclature. We also kept the name for the *TAAR-like* genes, which is the sister clade of all other vertebrate *TAAR* genes. The tree in newick version, as well as all sequences and the alignment file can be found in FigShare. Sub-trees of each vertebrate (sub)class with the number of *TAAR* genes are shown in Supplementary Fig. 18. Maximum likelihood phylogeny of *TAAR* genes extracted from one species per vertebrate (sub)class is shown in Supplementary Figs. 19-20.

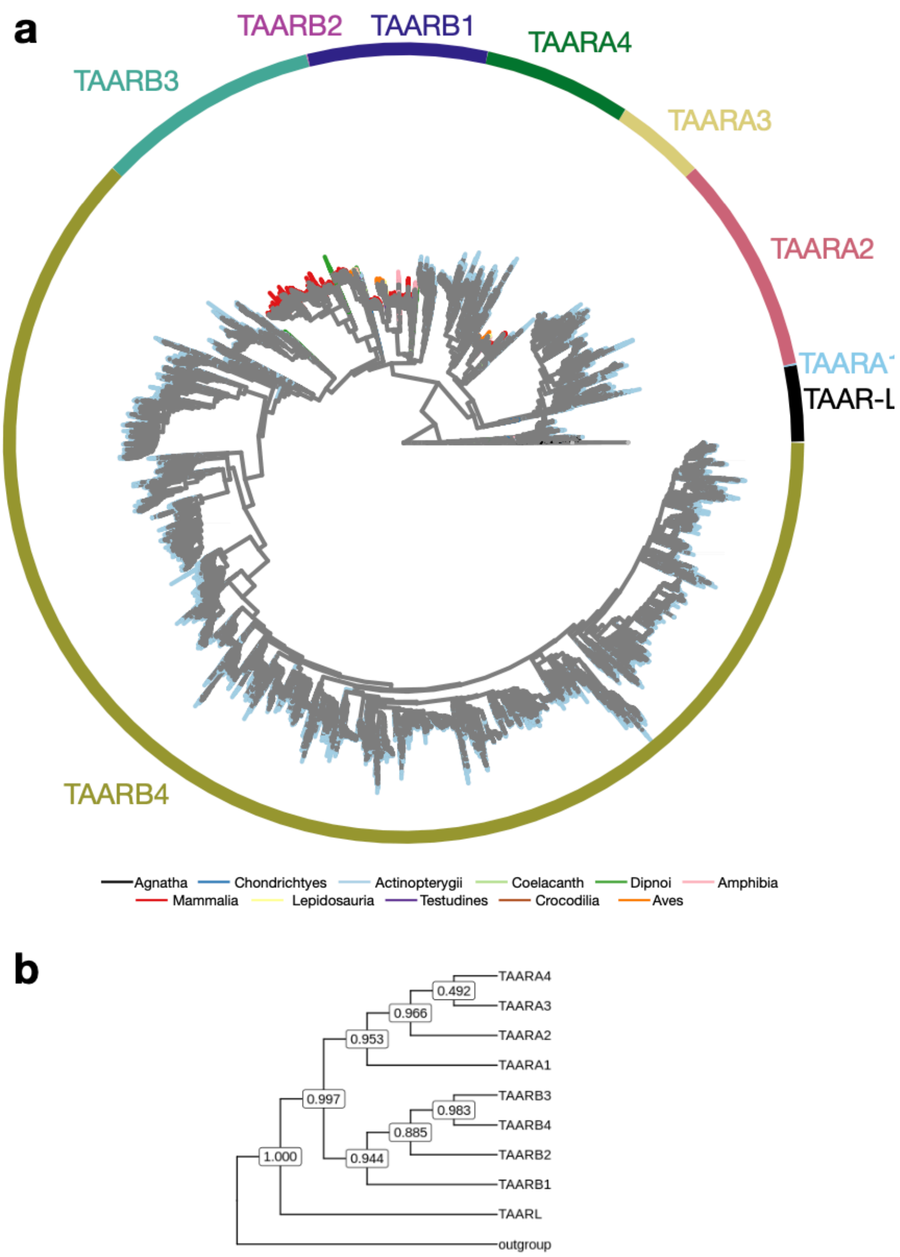

Supplementary Fig. 18 | Phylogenies showing the number of complete *TAAR* genes. Vertebrate phylogeny and (sub)class phylogenies displaying the number of complete *TAAR* genes (BUSCO80 dataset). **a**, Vertebrata; **b**, Agnatha; **c**, Chondrichthyes; **d**, Actinopterygii; **e**, Amphibia; **f**, Mammalia; **g**, Lepidosauria; **h**, Testudines; **i**, Aves and Crocodilia. Terminal branches and species names are colored according to (sub)class (**a**) or according to order based on the NCBI taxonomy database (**b**-**i**). *TAAR*-subclades are indicated. Source data are provided as a Source Data file.

a) Vertebrates

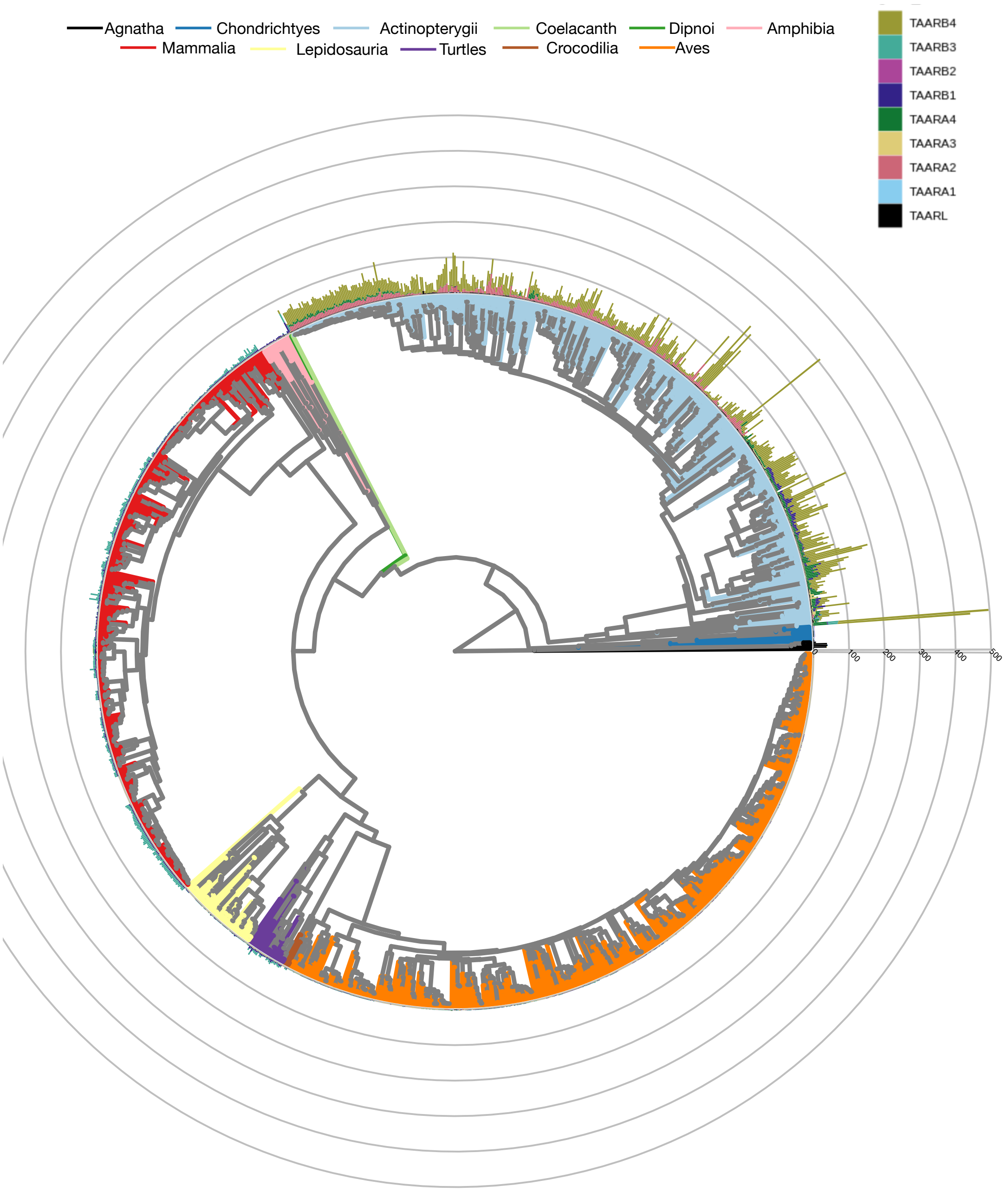

b) Agnatha

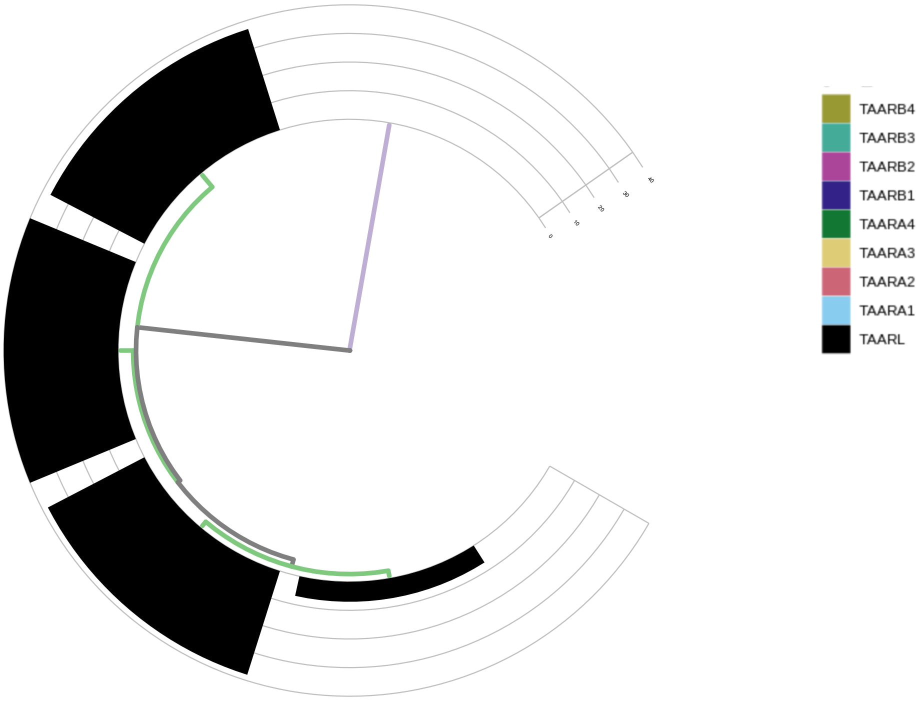

c) Chondrichthyes

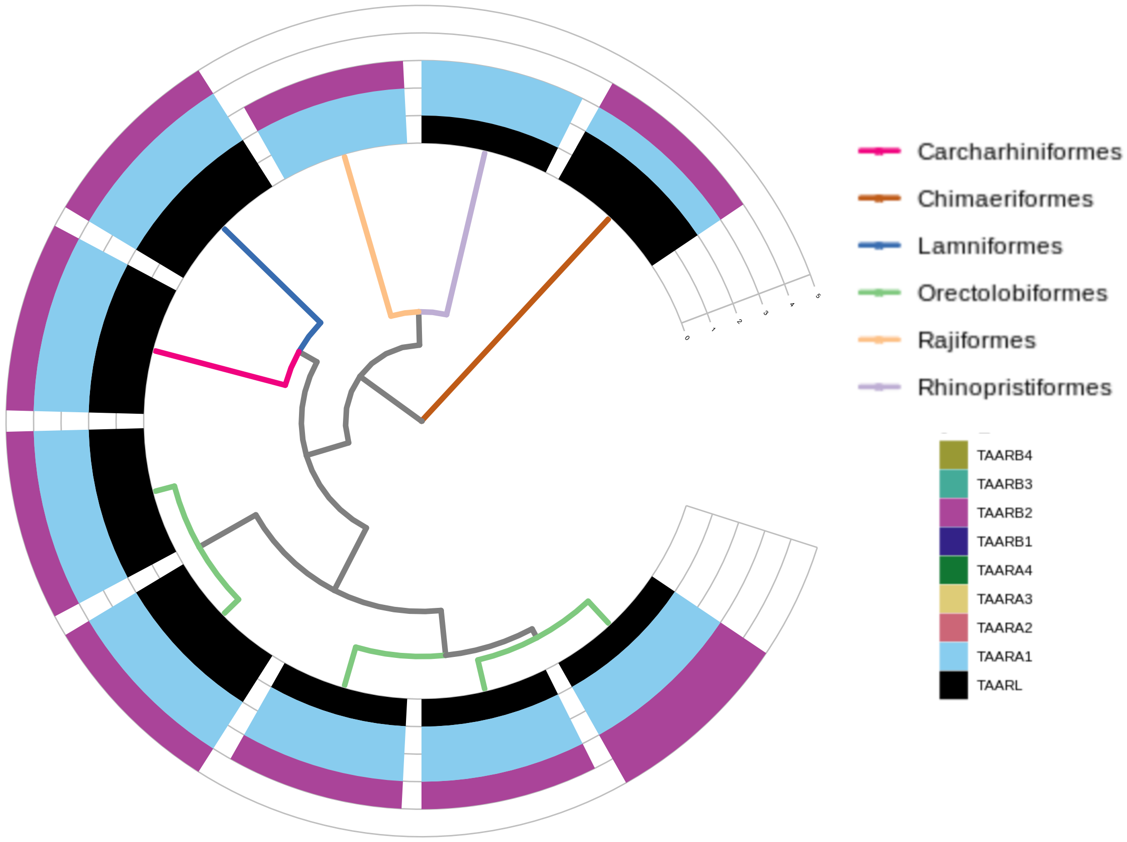

d) Actinopterygii

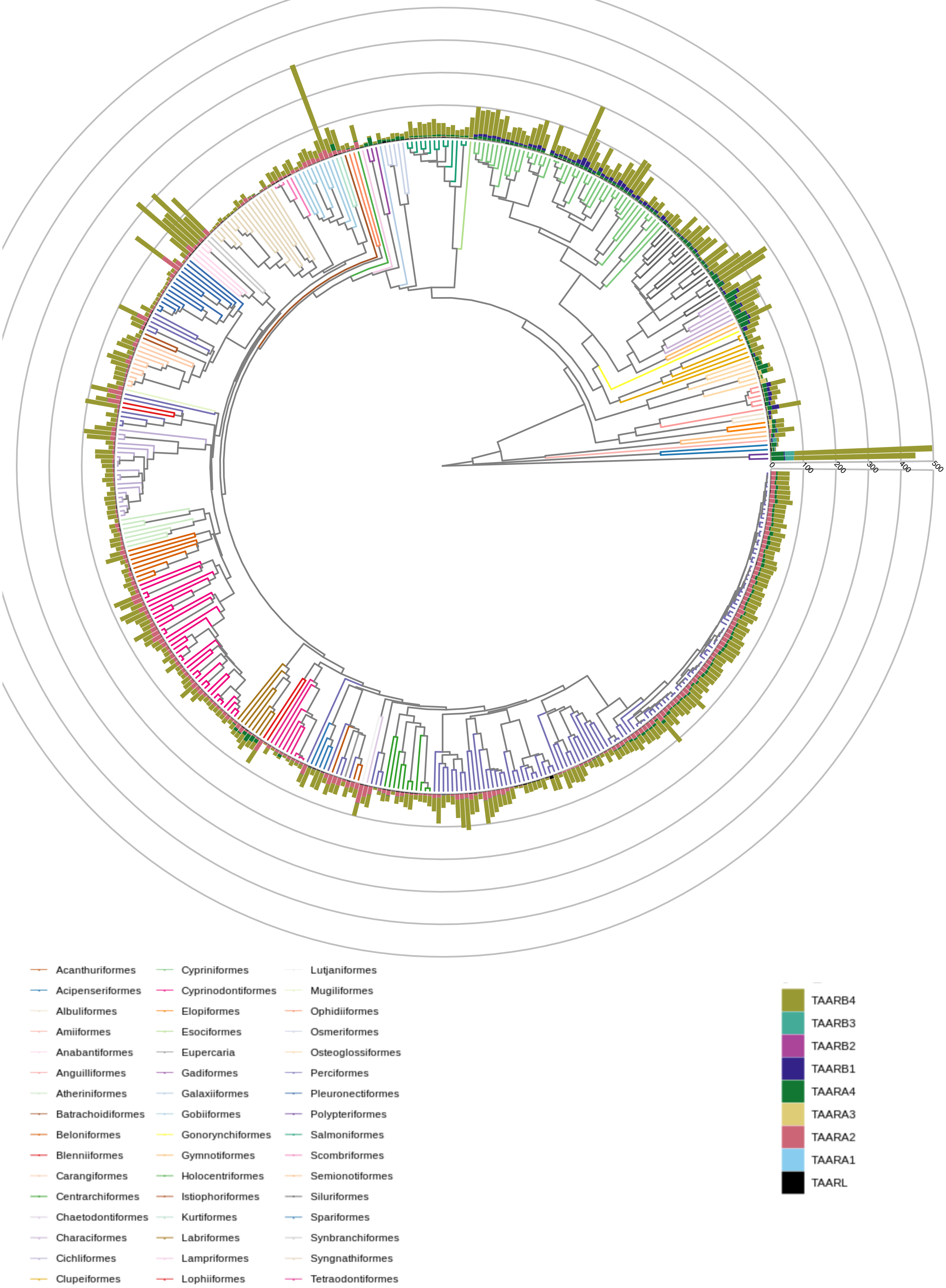

e) Amphibia

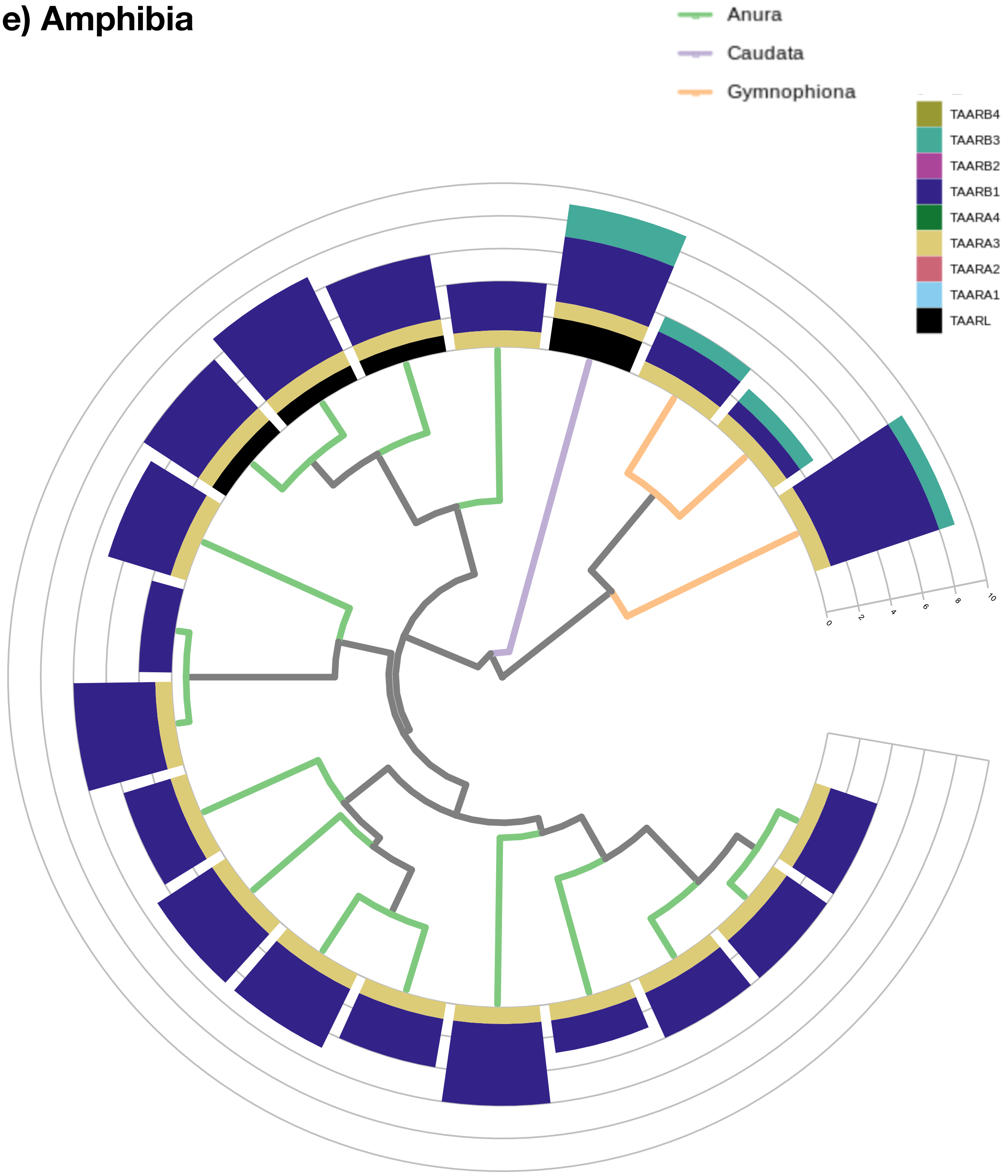
Anura
Caudata
Gymnophiona
TAARB4
TAARB3
TAARB2
TAARB1
TAARA4
TAARA3
TAARA2
TAARA1
TAARL

f) Mammalia

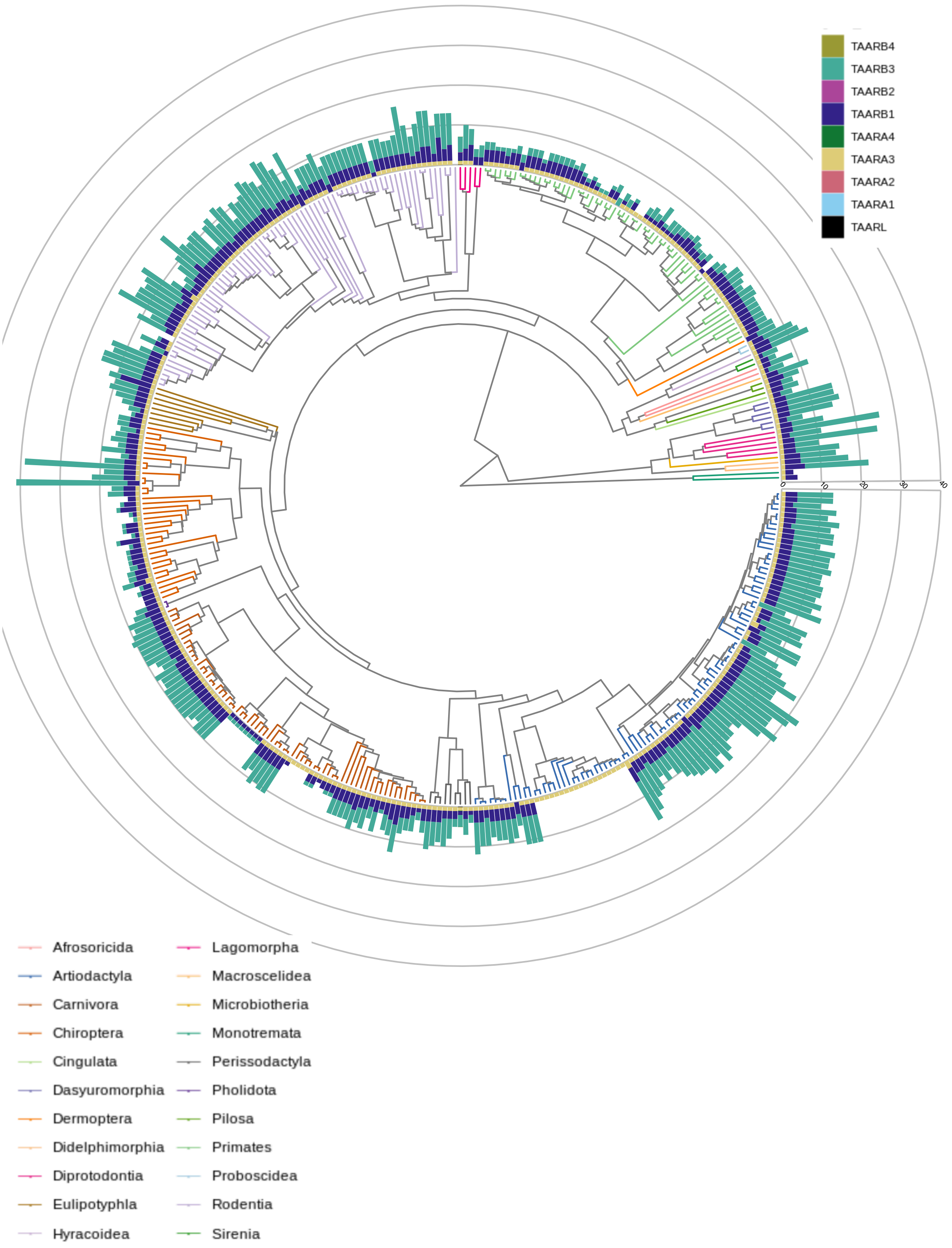

TAARB4
TAARB3
TAARB2
TAARB1
TAARA4
TAARA3
TAARA2
TAARA1
TAARL
0
10
20
30
40
Afrosoricida
Artiodactyla
Carnivora
Chiroptera
Cingulata
Dasyuromorphia
Dermoptera
Didelphimorphia
Diprotodontia
Eulipotyphla
Hyracoidea
Lagomorpha
Macroscelidea
Microbiotheria
Monotremata
Perissodactyla
Pholidota
Pilosa
Primates
Proboscidea
Rodentia
Sirenia

g) Lepidosauria

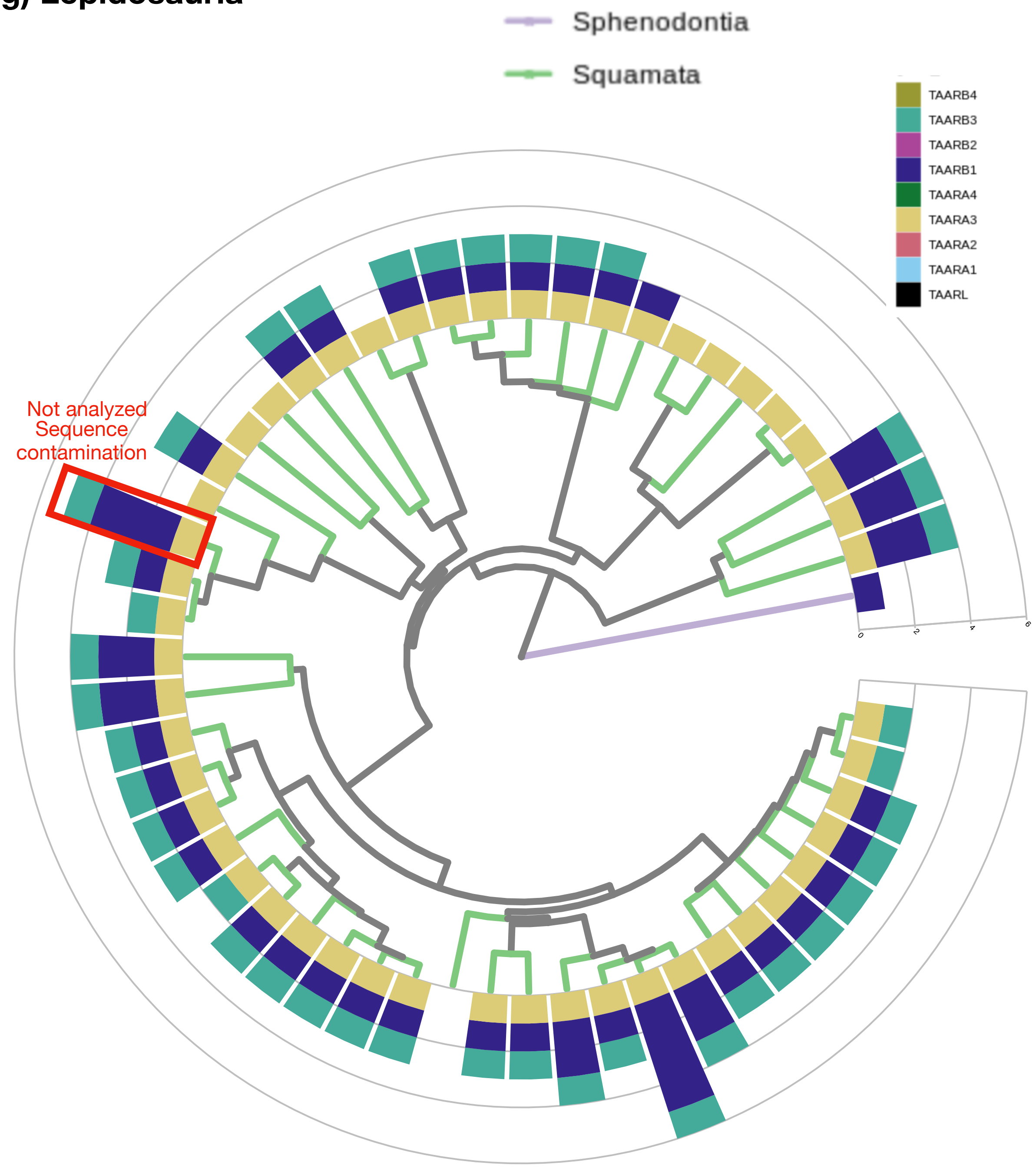

h) Turtles

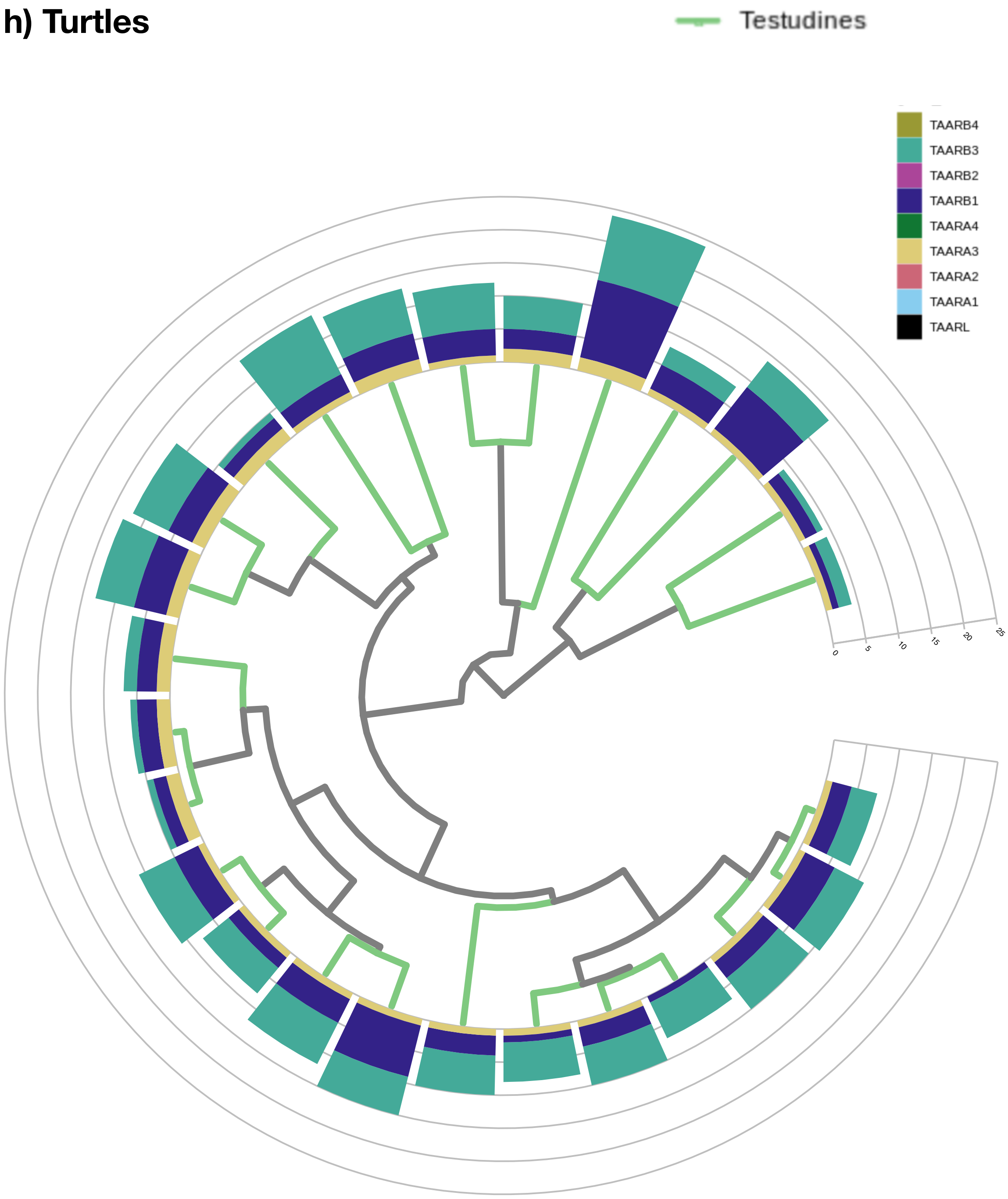

Testudines
TAARB4
TAARB3
TAARB2
TAARB1
TAARA4
TAARA3
TAARA2
TAARA1
TAARL
0
5
10
15
20
25

i) Aves (with Crocodilia as outgroup)

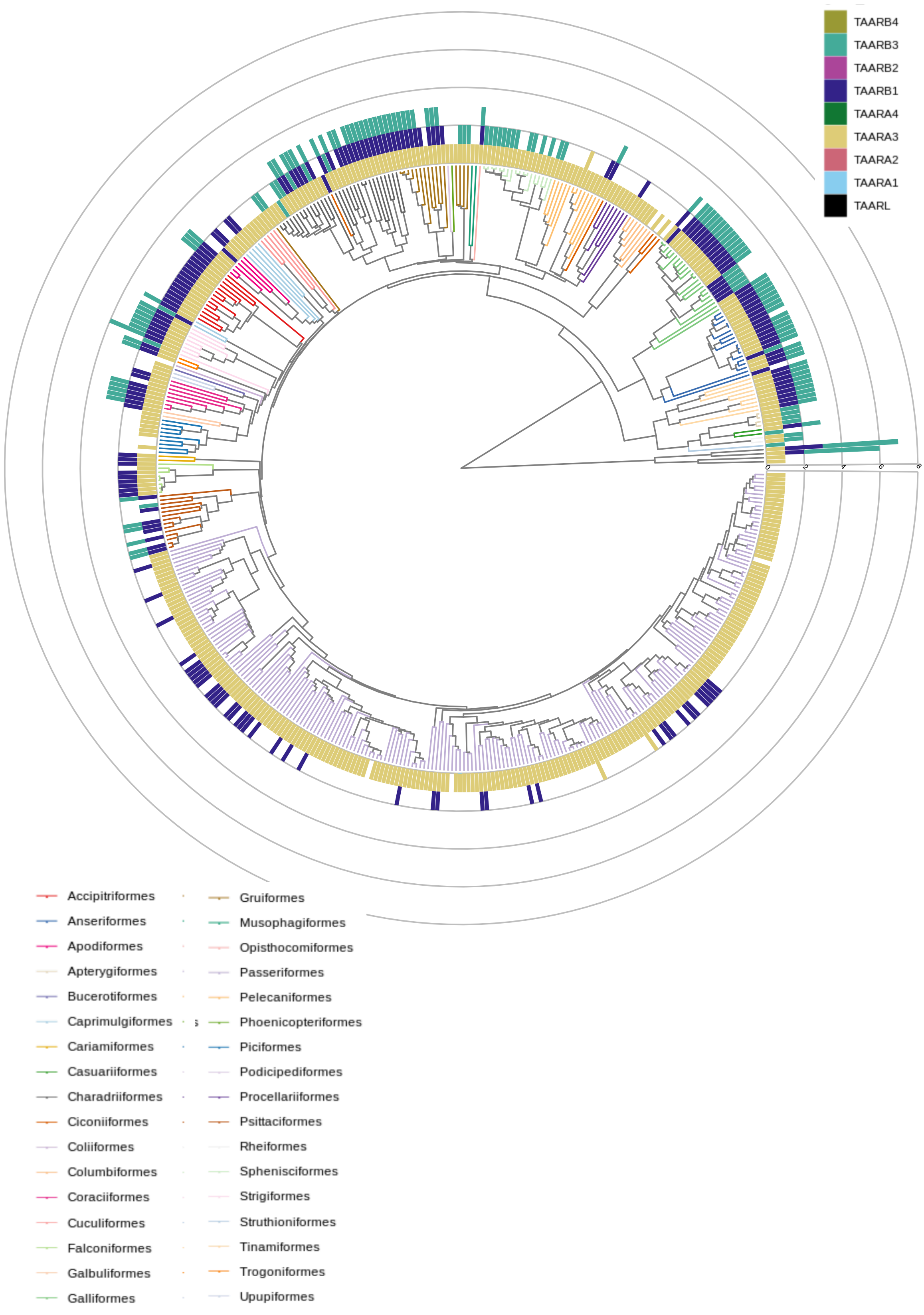

Supplementary Fig. 19 | Maximum likelihood phylogeny of *TAAR* genes. Maximum likelihood phylogeny of *TAAR* genes extracted from one representative species per vertebrate subclass. Branches are colored according to the vertebrate (sub)class. *TAAR* subclades are indicated. *TAARA1* is labeled directly in the phylogenetic tree as there was only one sequence in this clade. The tree was computed using the optimal model found by ModelFinder (LG+I+G). The robustness of the nodes was evaluated with 1,000 ultrafast bootstraps and these bootstrap values are reported for each node in Supplementary Fig. 20. All the clades retrieved using the near-ML method were retrieved as monophyletic, and the exact same topology was retrieved.

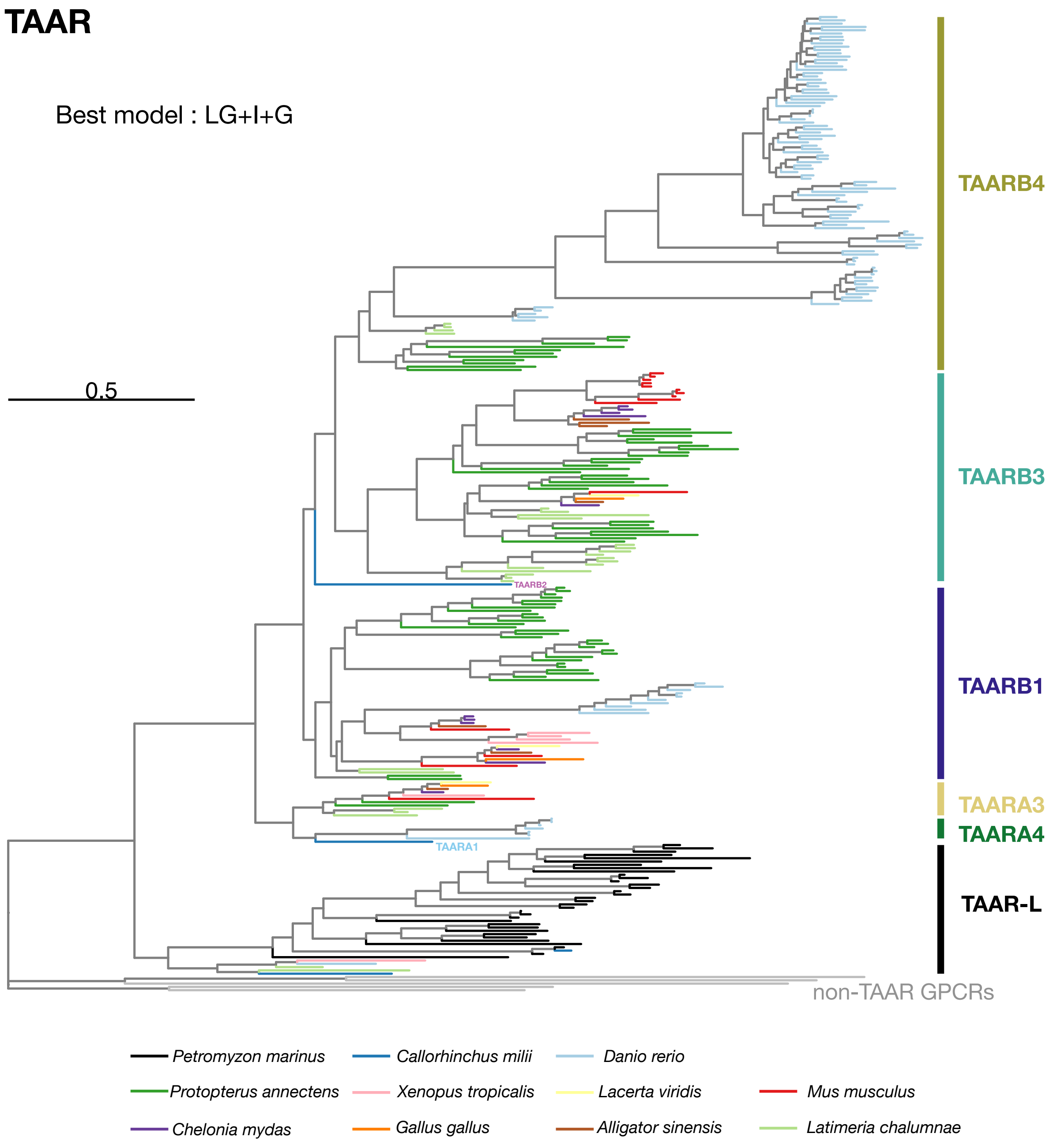

Supplementary Fig. 20 | Maximum likelihood phylogeny of *TAAR* genes. Same maximum likelihood phylogeny of *TAAR* genes as in Supplementary Fig. 19 but with bootstrap values indicated at each node.

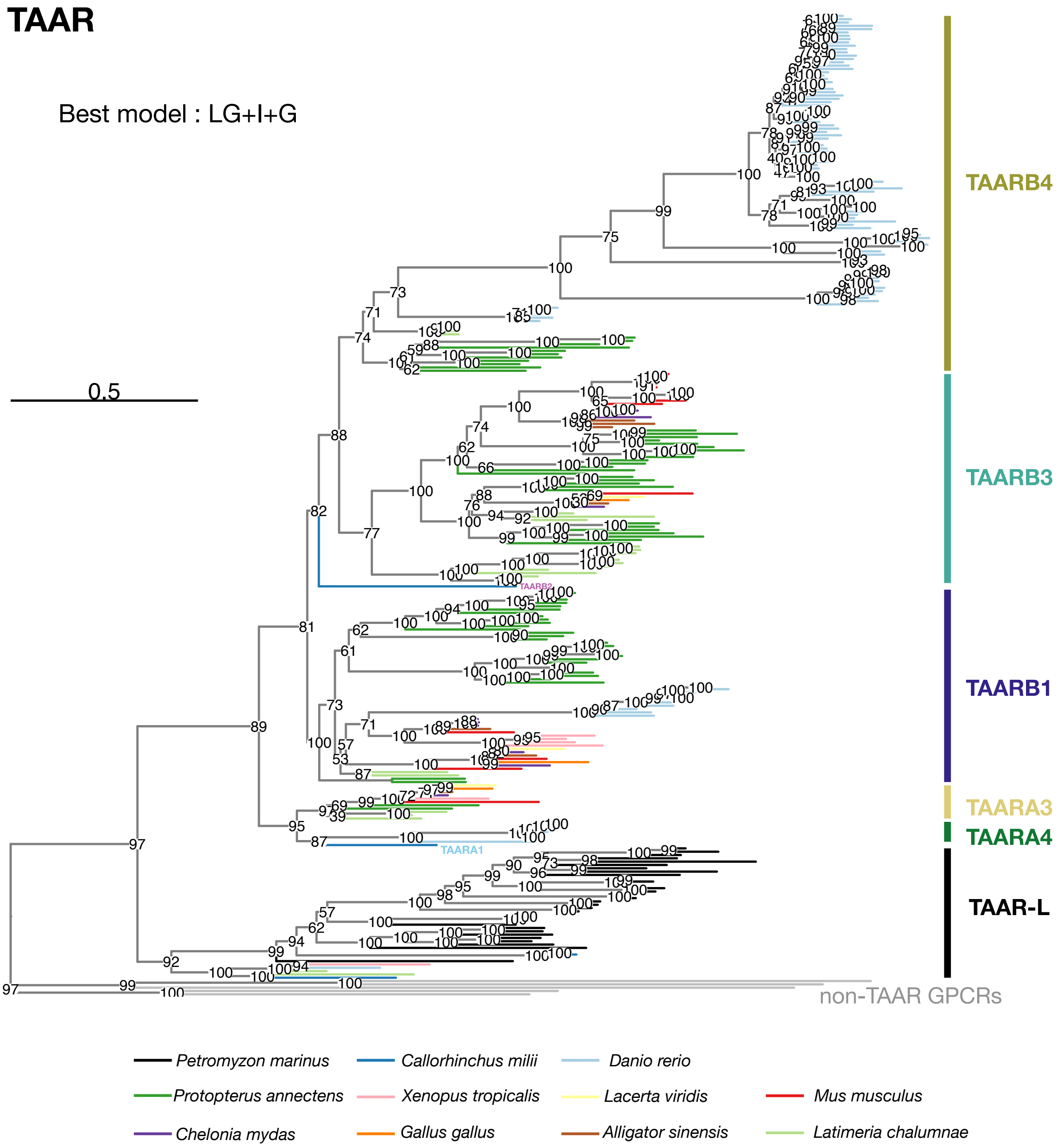

Supplementary Fig. 21 | Phylogenies showing the number of complete *V1R* genes. Vertebrate phylogeny and (sub)class phylogenies displaying the number of complete *V1R* genes (BUSCO80 dataset). **a**, Vertebrata; **b**, Agnatha; **c**, Chondrichthyes; **d**, Actinopterygii; **e**, Amphibia; **f**, Mammalia; **g**, Lepidosauria; **h**, Testudines; **i**, Aves and Crocodilia. Terminal branches and species names are colored according to (sub)class (**a**) or according to order based on the NCBI taxonomy database (**b-i**). *V1R*-subclades are indicated. Source data are provided as a Source Data file.

a) Vertebrates

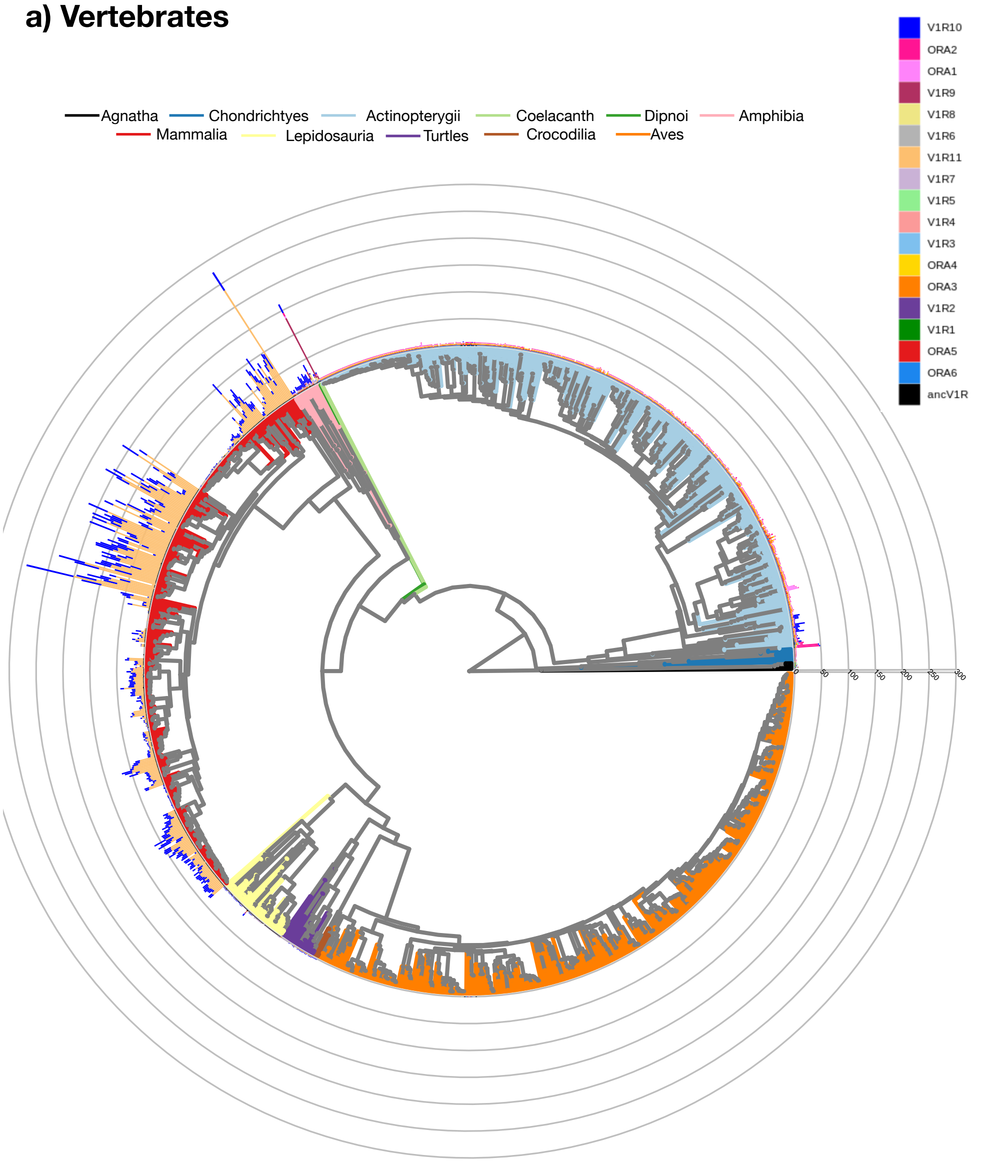

b) Agnatha

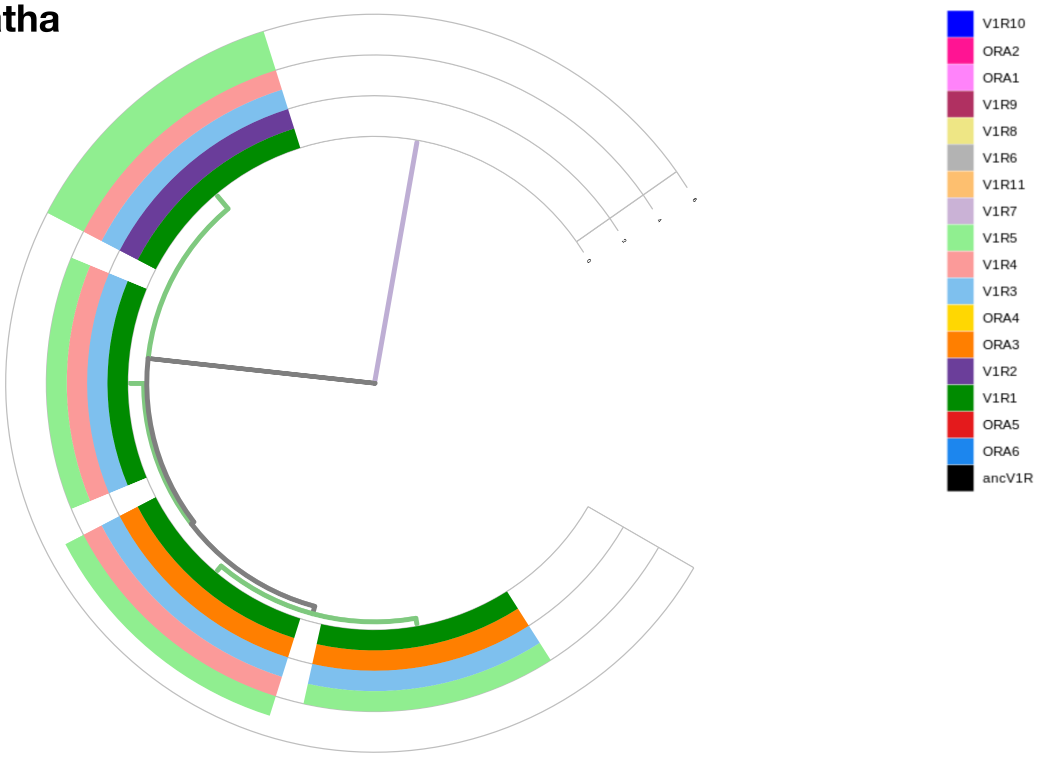

c) Chondrichthyes

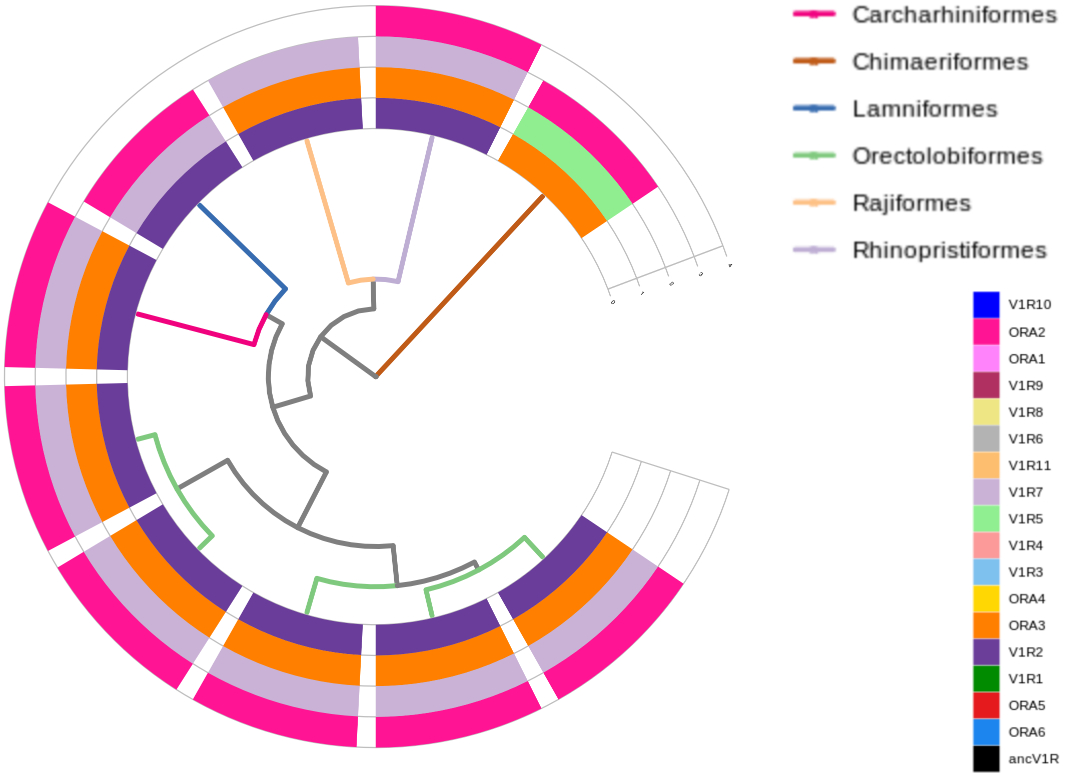

d) Actinopterygii

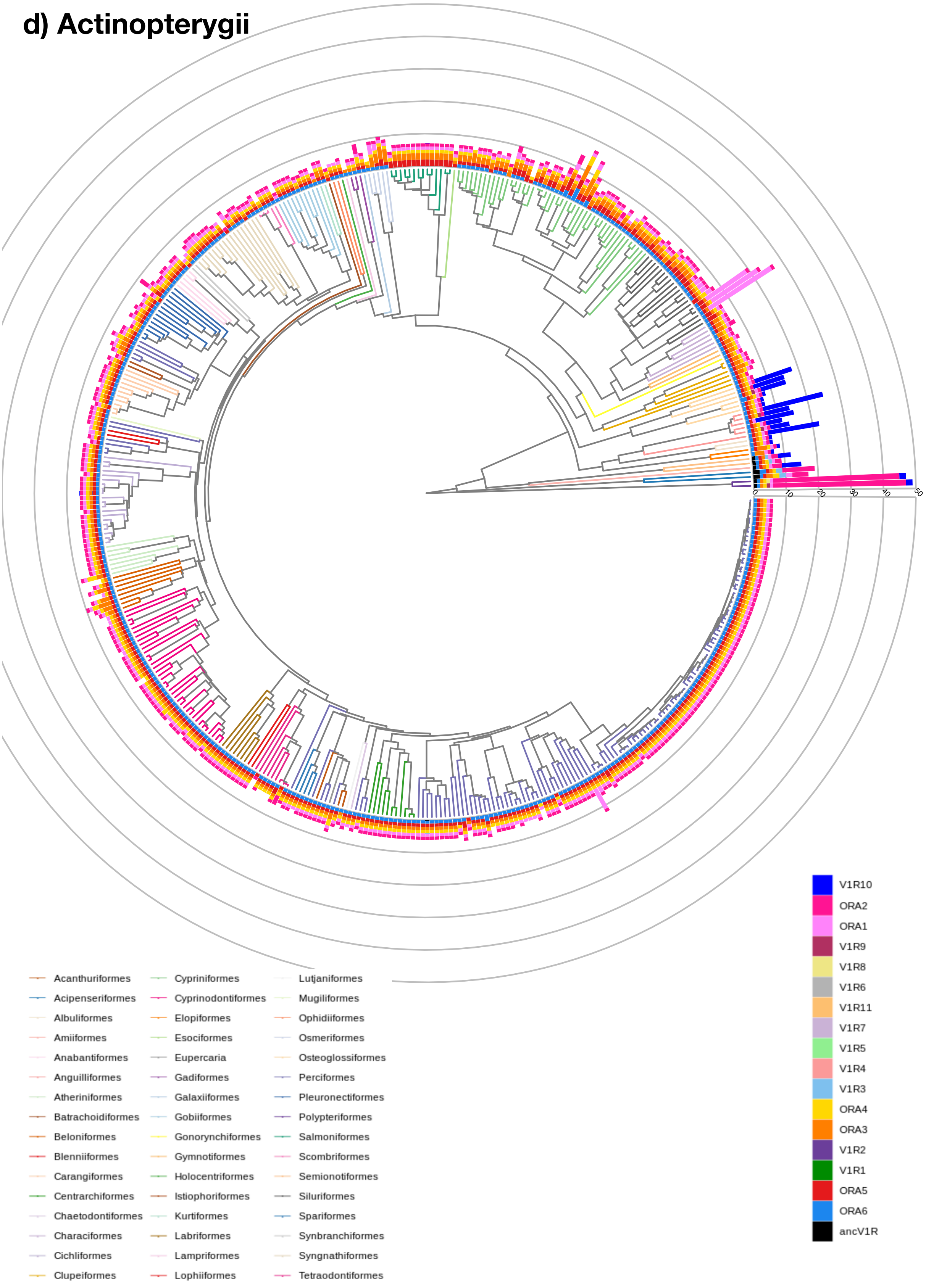

e) Amphibia

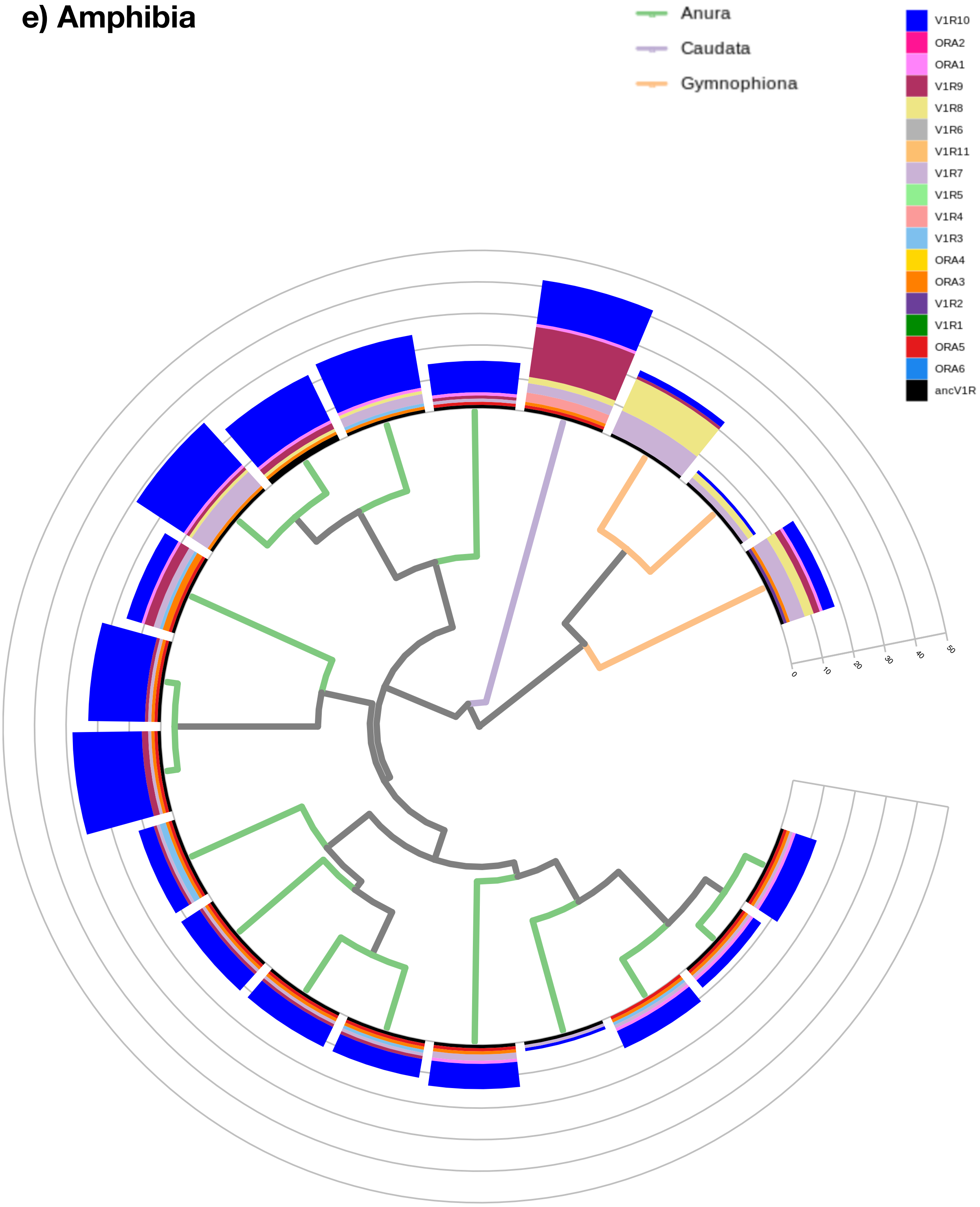
Anura
Caudata
Gymnophiona
V1R10
ORA2
ORA1
V1R9
V1R8
V1R6
V1R11
V1R7
V1R5
V1R4
V1R3
ORA4
ORA3
V1R2
V1R1
ORA5
ORA6
ancV1R
0
10
20
30
40
50

f) Mammalia

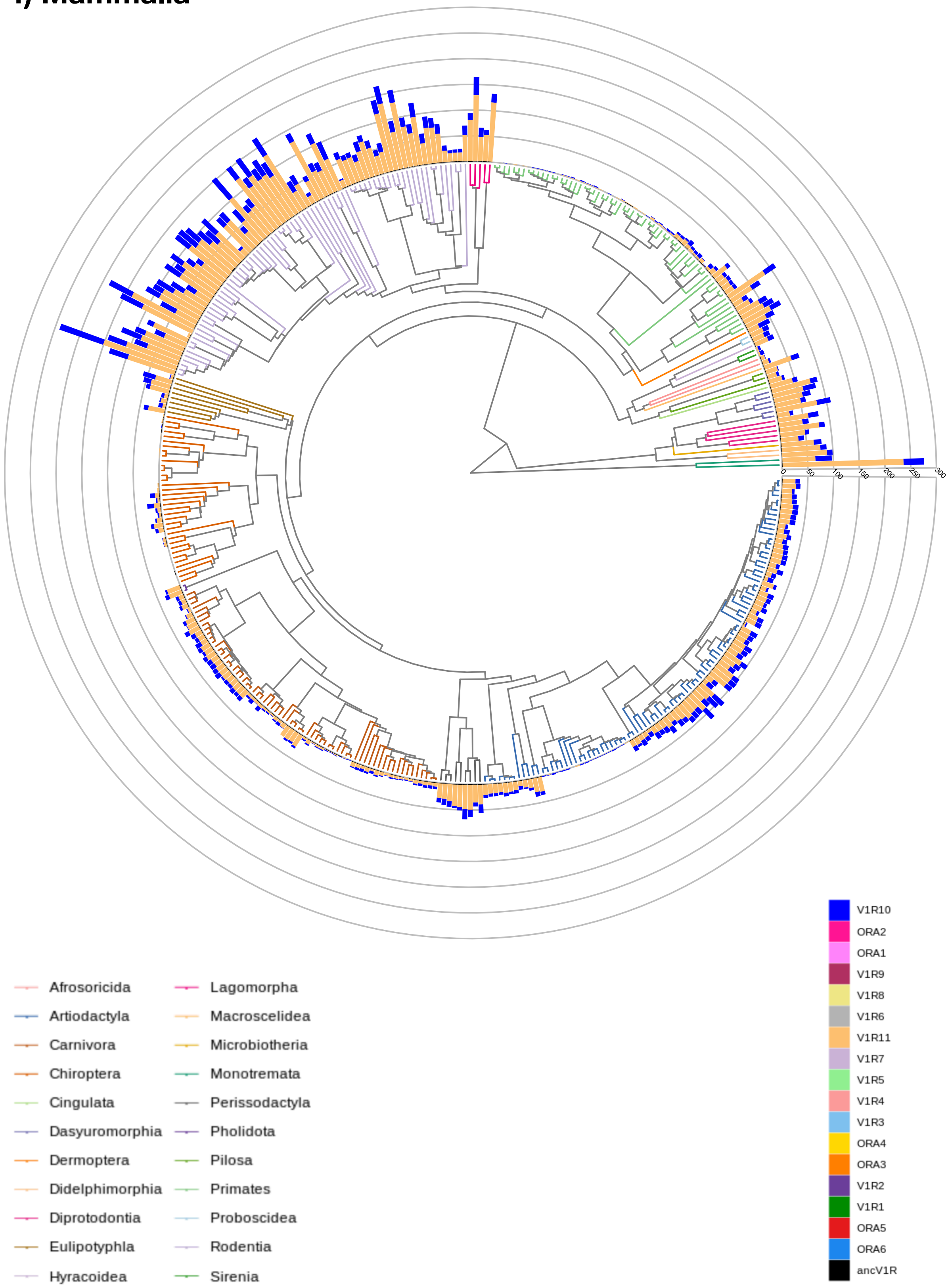
0
50
100
150
200
250
300
Afrosoricida
Artiodactyla
Carnivora
Chiroptera
Cingulata
Dasyuromorphia
Dermoptera
Didelphimorphia
Diprotodontia
Eulipotyphla
Hyracoidea
Lagomorpha
Macroscelidea
Microbiotheria
Monotremata
Perissodactyla
Pholidota
Pilosa
Primates
Proboscidea
Rodentia
Sirenia
V1R10
ORA2
ORA1
V1R9
V1R8
V1R6
V1R11
V1R7
V1R5
V1R4
V1R3
ORA4
ORA3
V1R2
V1R1
ORA5
ORA6
ancV1R

g) Lepidosauria

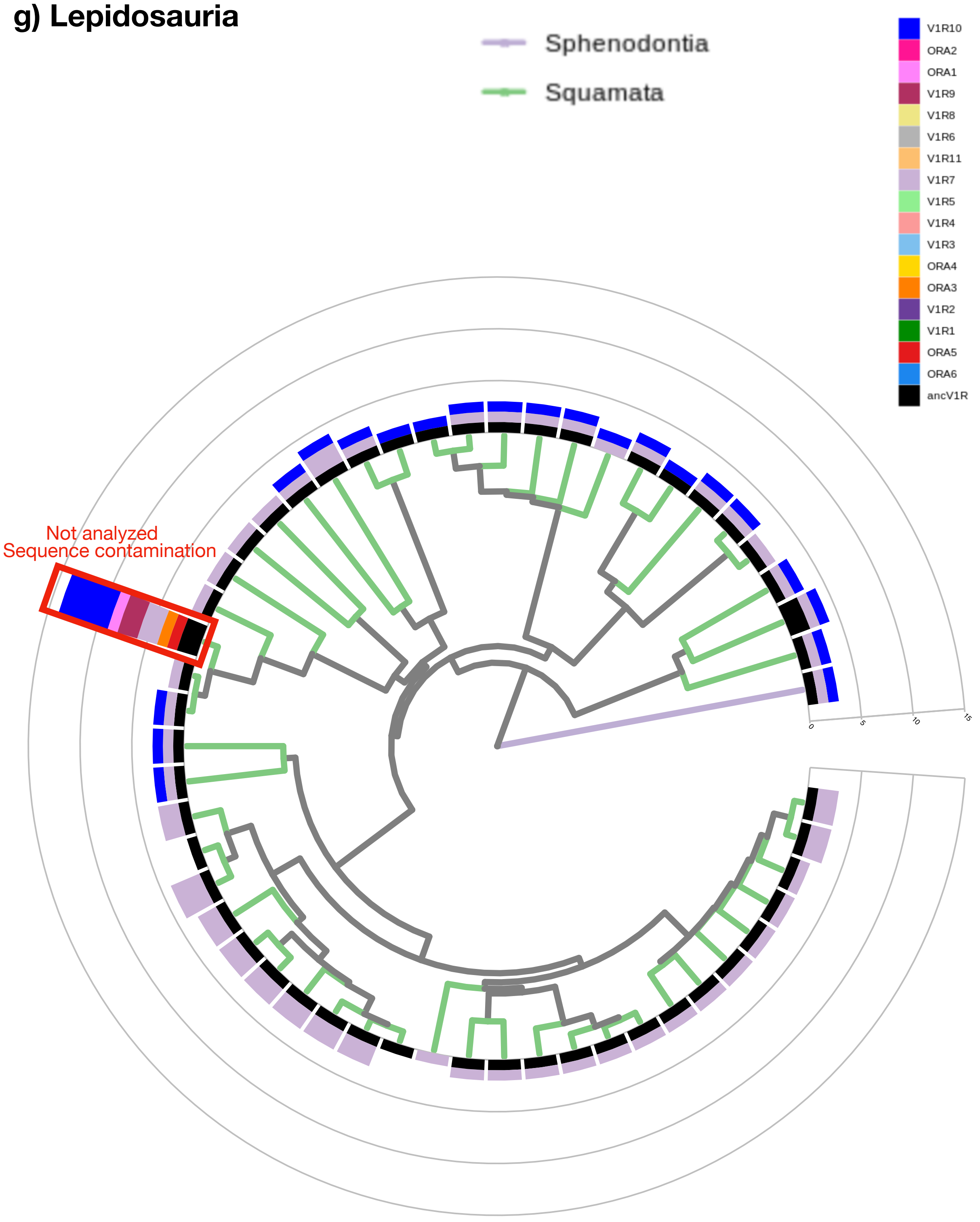

h) Turtles

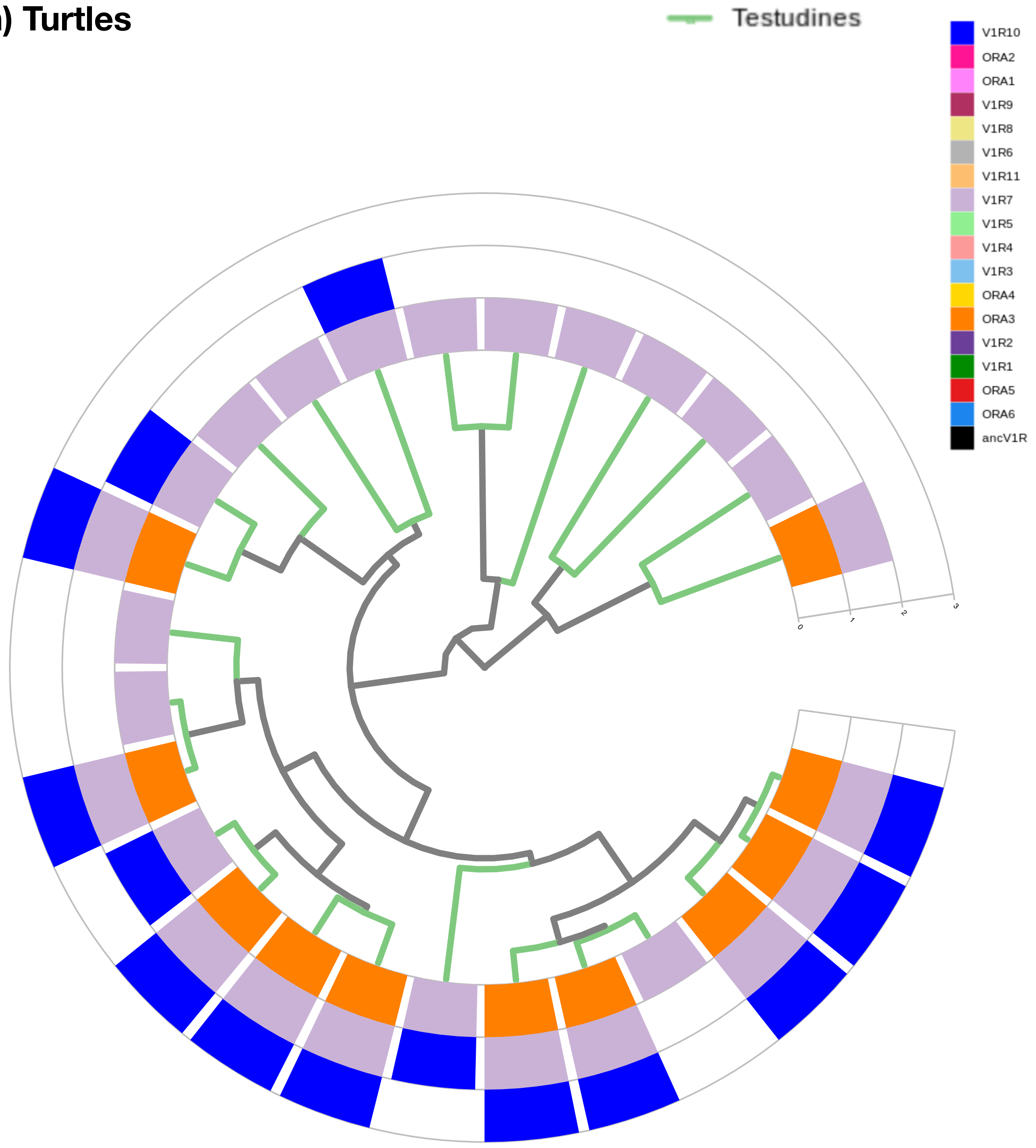

Testudines
V1R10
ORA2
ORA1
V1R9
V1R8
V1R6
V1R11
V1R7
V1R5
V1R4
V1R3
ORA4
ORA3
V1R2
V1R1
ORA5
ORA6
ancV1R
0
1
2
3

i) Aves (with Crocodilia as outgroup)

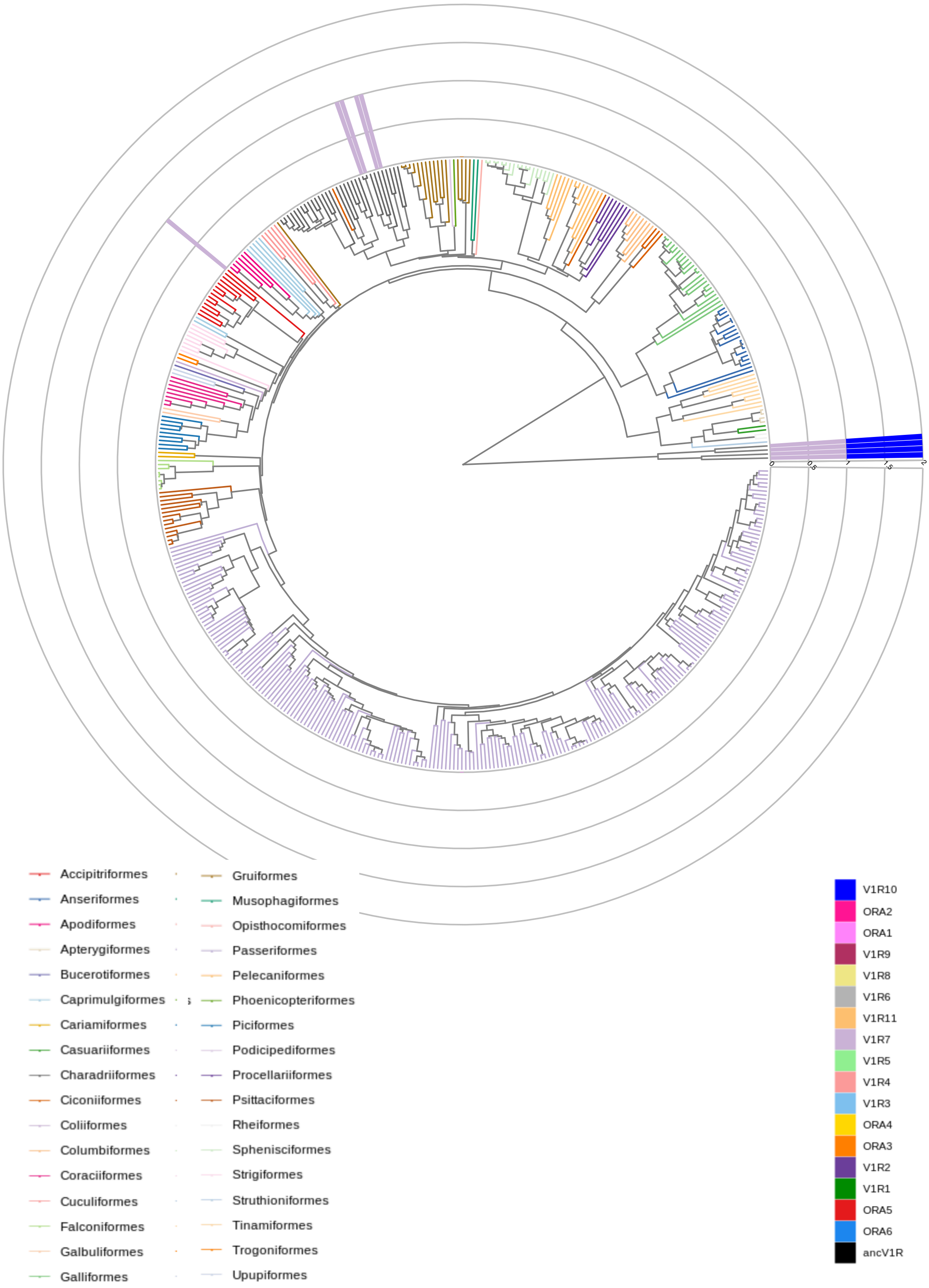

Supplementary Fig. 22 | Vertebrate *V1R* gene tree. a, Near maximum likelihood phylogeny of *V1R* genes in vertebrates with branches colored according to the vertebrate (sub)class. **b**, Clade tree generated from (**a**) with local support values indicated at each node (according to a Shimodaira-Hasegawa test). We kept the clade name for *ancV1R*, which denotes "ancestral *V1R*" as proposed by Suzuki et al.[23]. In our gene tree, this clade is the sister clade to all other *V1R* genes, while in the latter study, *ancV1R* genes were clustered together with the *ORA5* and *ORA6* clades. While Suzuki et al. suggested that *ancV1R* originated before the MRCA of bony vertebrates, we found that *ancV1R* genes most likely originated before the MRCA of all vertebrates, followed by a loss in jawless and in cartilaginous fishes. All other *V1R* clades, which were only poorly characterized in previous studies, were renamed *V1R1* to *V1R11*, while we kept the former *ORA* clade nomenclature (*ORA1* to *ORA6*) for teleosts. The tree in newick version, as well as all sequences and the alignment file can be found in FigShare. Sub-trees of each vertebrate (sub)class with the number of *V1R* genes are shown in Supplementary Fig. 21. Maximum likelihood phylogeny of *V1R* genes extracted from one species per vertebrate (sub)class is shown in Supplementary Figs. 23-24.

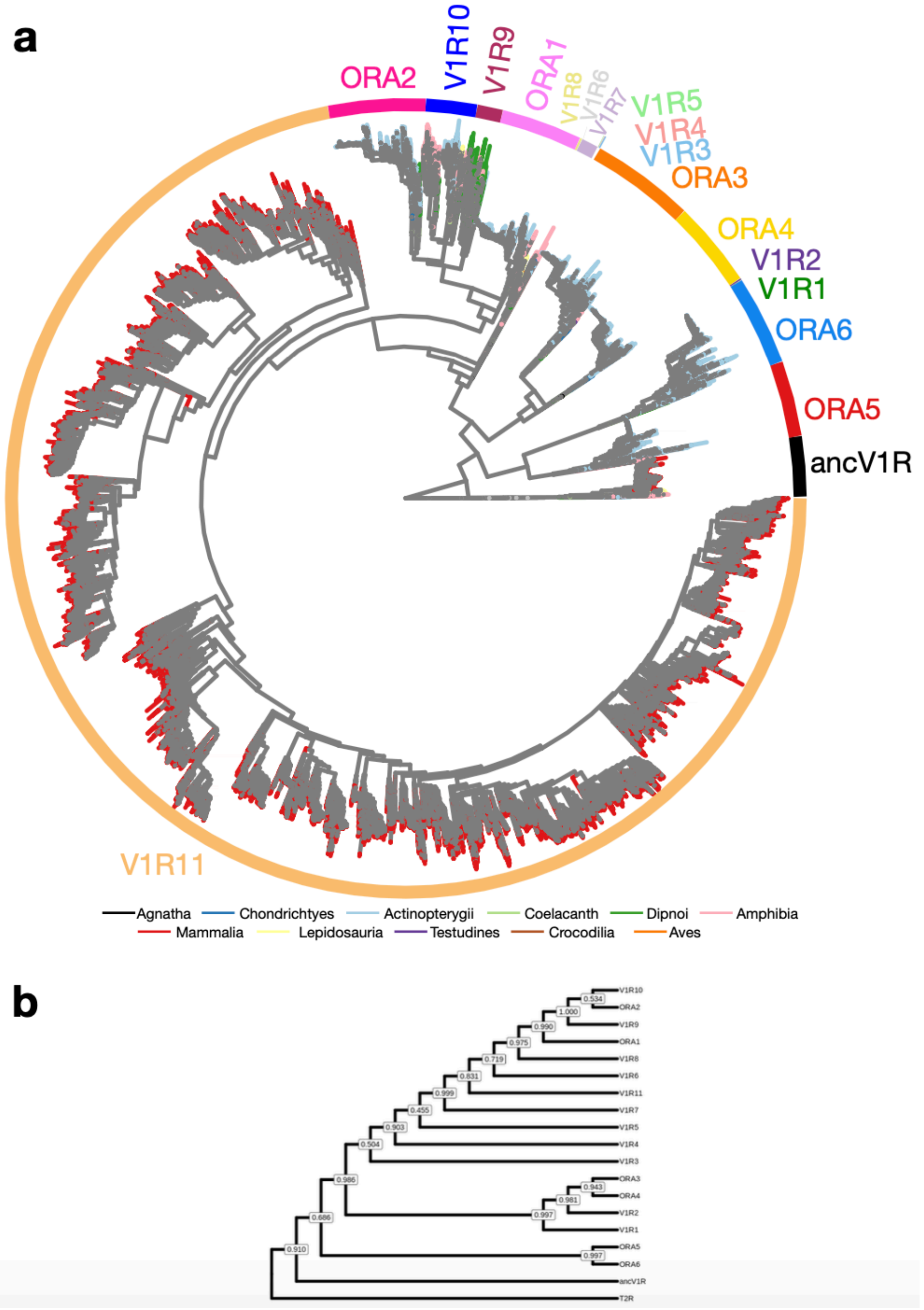

Supplementary Fig. 23 | Maximum likelihood phylogeny of *V1R* genes. Maximum likelihood phylogeny of *V1R* genes extracted from one representative species per vertebrate subclass. Branches are colored according to the vertebrate (sub)class. V1R subclades are indicated. R8 clade is directly indicated inside the phylogenetic tree as there was only one sequence in this clade. The tree was computed using the optimal model found by ModelFinder (JTT+F+R8). The robustness of the nodes was evaluated with 1,000 ultrafast bootstraps and these bootstrap values are reported for each node in Supplementary Fig. 24. While the overall topology was similar to the *V1R* near-ML tree, the *ORA2* clade found by the near-ML method was retrieved as paraphyletic, and divided in three distinct clades in the ML tree. We also retrieved a sister relationship between the *ancV1R* clade and the *ORA5*/*ORA6* clade, similar to Suzuki et al.[23], while *ancV1R* was the sister clade to all other *V1R* genes in our near-ML topology.

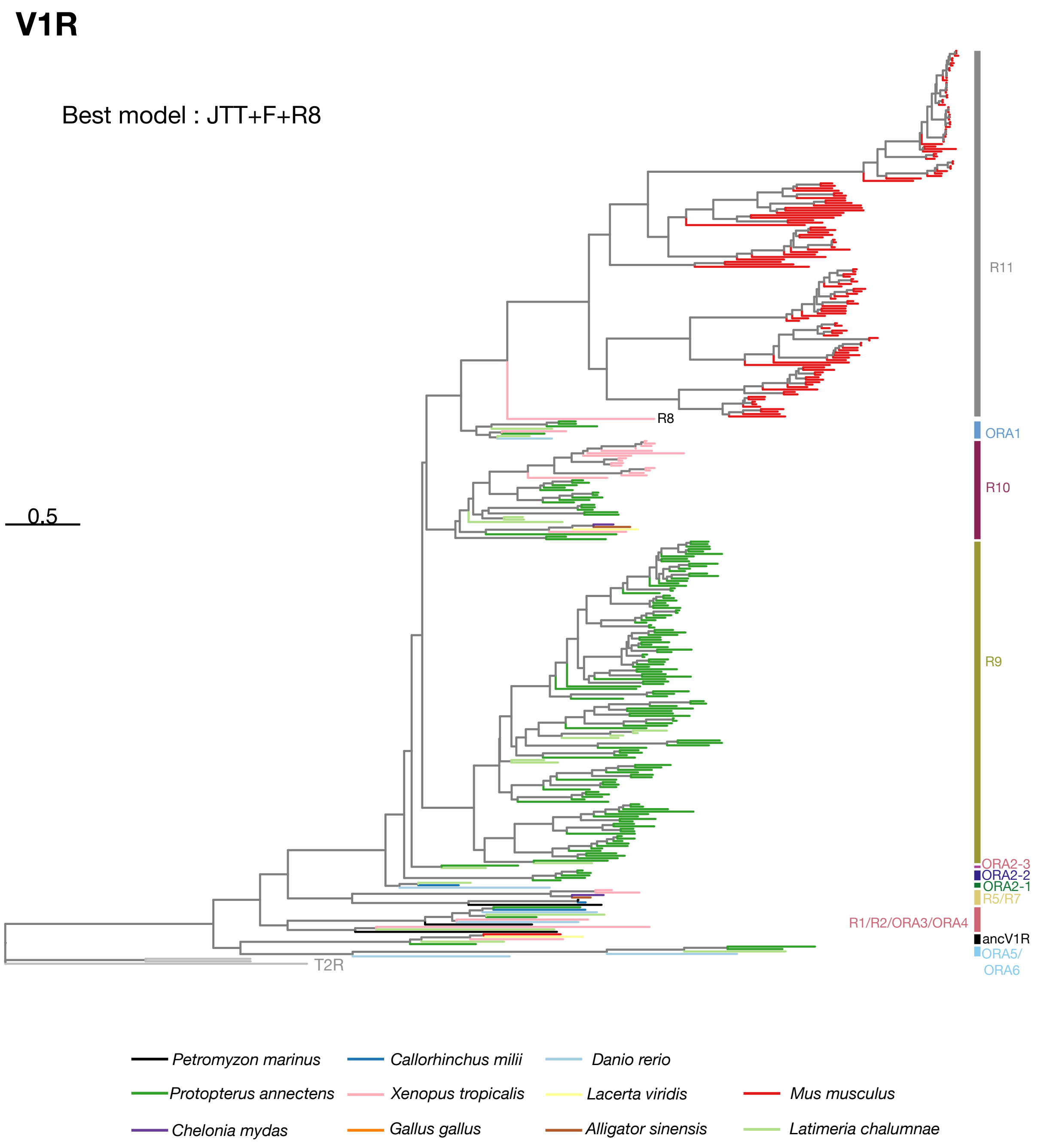

Supplementary Fig. 24 | Maximum likelihood phylogeny of *V1R* genes. Same maximum likelihood phylogeny of *V1R* genes as in Supplementary Fig. 23 but with bootstrap values indicated at each node.

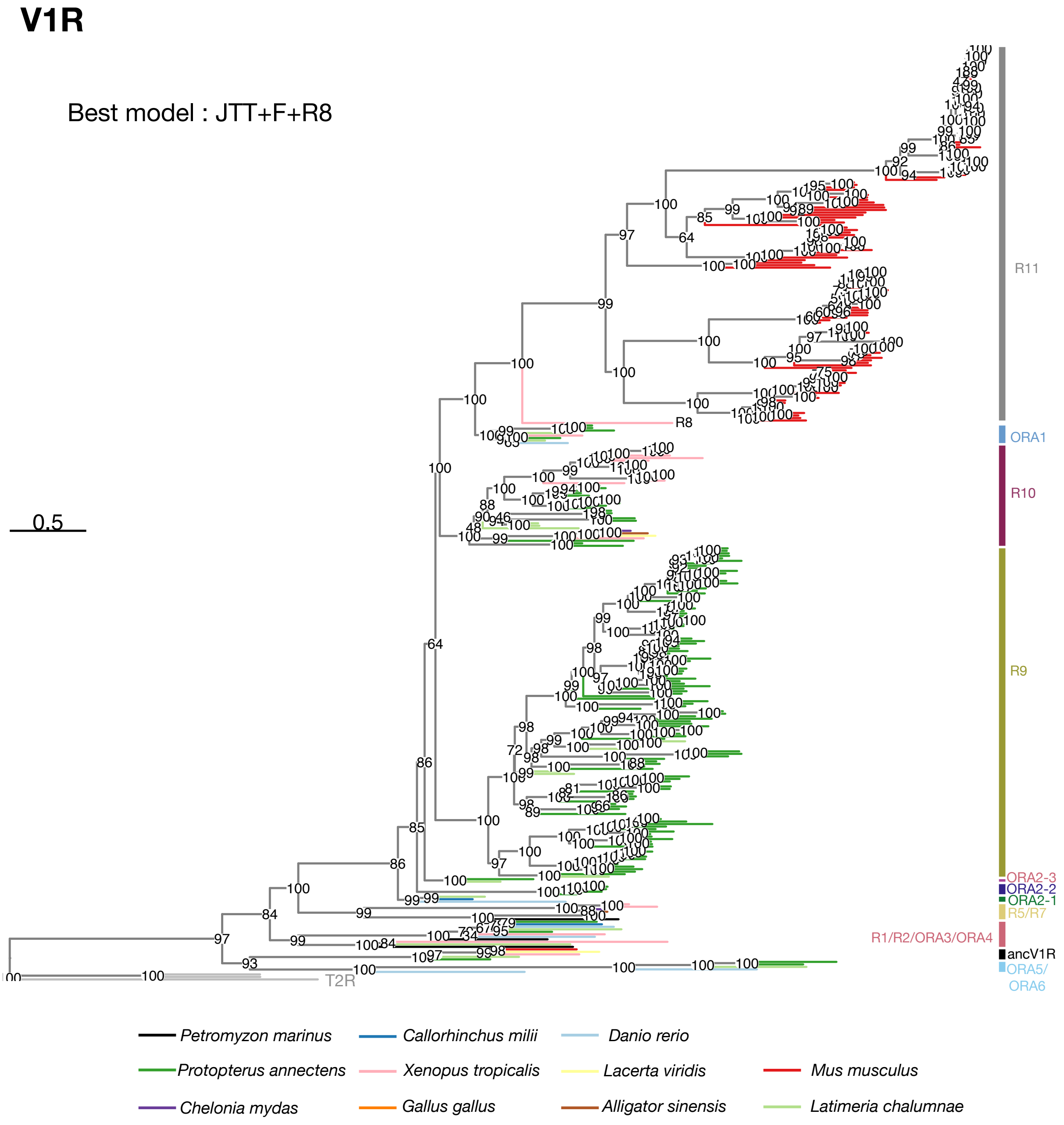

Supplementary Fig. 25 | Evolutionary history of *V1R* and *V2R* genes in vertebrates. a, Schematic of the *V1R* gene tree (top) indicating the subclades within the *V1R* gene family in vertebrates. Gene duplication events leading to subclade divergence are indicated as triangles on the respective branches in the schematic vertebrate phylogeny (left), subclade losses are indicated with the cross symbol below the respective branches. The numbers refer to nodes in the gene tree. Circles on the right indicate the presence of a particular subclade in a given evolutionary lineage, whereby the size of each circle corresponds to the mean number of complete *V1R* genes per species in the respective subclade and lineage. The *V1R* gene tree is shown in Supplementary Fig. 22. **b**, Schematic of the *V2R* gene tree (top) indicating the subclades within the *V2R* gene family in vertebrates. Gene duplication events are indicated as triangles on the respective branches in the schematic vertebrate phylogeny (left), subclade losses are indicated with the cross symbol. The numbers refer to nodes in the gene tree. Circles on the right indicate the presence of a particular subclade in a given evolutionary lineage, whereby the size of each circle corresponds to the mean number of complete V2R genes per species in the respective subclade and lineage. The *V2R* gene tree is shown in Supplementary Fig. 28. Subclades present in non-teleost fishes but absent in teleosts are marked with an "*" (*ancV1R*, *V1R3*, *V1R8*, *V1R9*, *V2RC3* and *V2RD1*). Subclades present in osteoglossomorph and elopomorph but absent in clupeocephalans (*V1R10*) are marked with an "#". Source data are provided as a Source Data file.

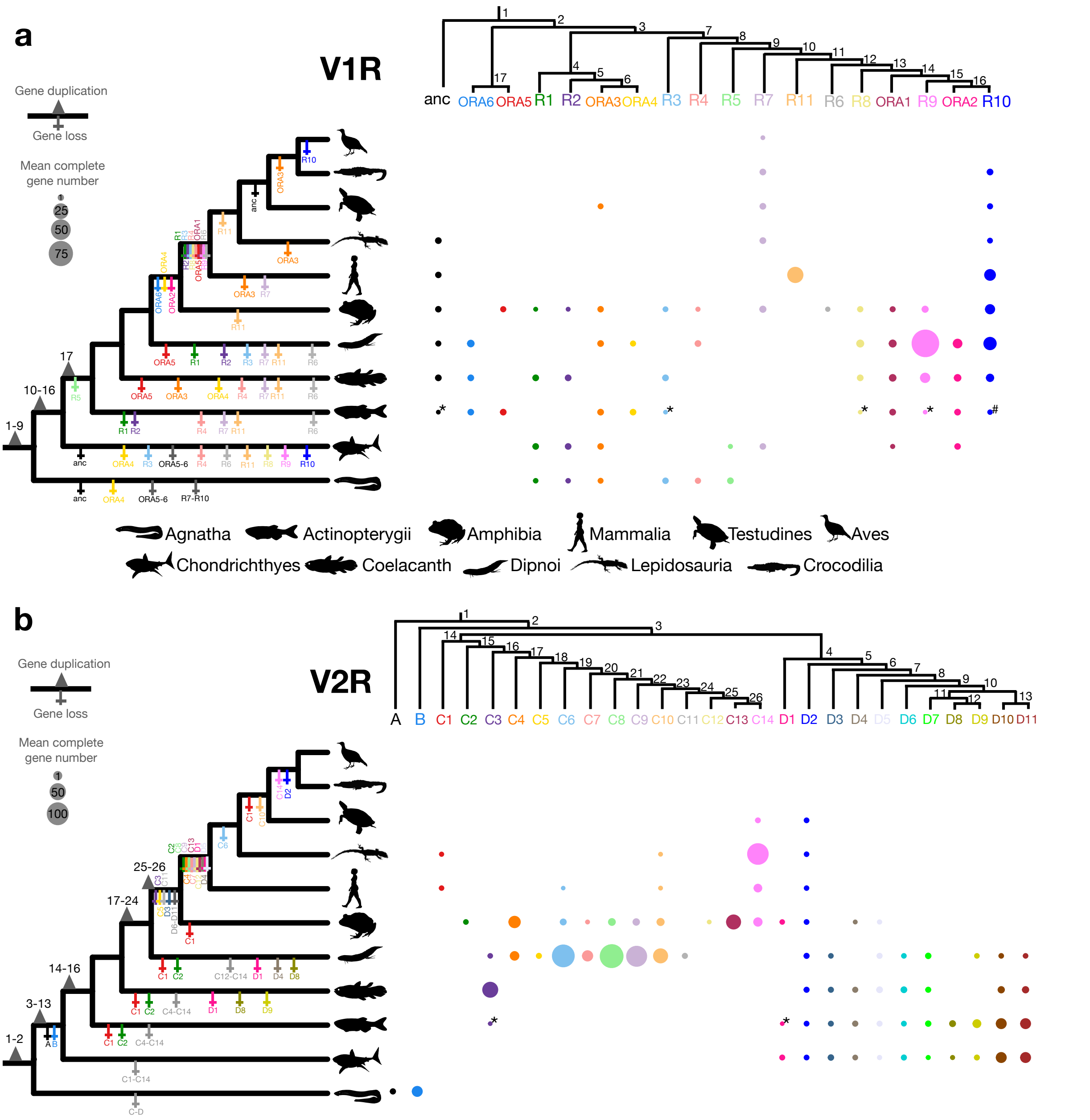

Supplementary Fig. 26 | Neutral evolution of bird *V1R* genes. PAML was used to compute the dN/dS (= ω) per branch under four different models. **a**, Model 1: Free-ratio model which assume one ω ratio per branch **b**, Model 2: Two-ratio model which assume one ω ratio for birds and one ω ratio for all other branches **c**, Model 3 : Two-ratio model with one ω ratio for birds and crocodiles and one ω for all other branches **d**, Model 4 : Two-ratio model with one ω ratio for birds, crocodiles and turtles and one ω for all other branches. For the free-ratio model, the mean ω ratio per clade is indicated. While the mean ω ratio was low for turtles (0.16), crocodiles (0.35), lepidosaurs (0.18) and amphibian (0.26), indicative of negative selection on *V1R7* in these clades, the mean ω ratio of birds (1.05) suggest a relaxation of selection. **e**, Log likelihood (Lnl) and number of parameters (np) of each model. The log-likelihood, as well as the ω value found under a null model (one-ratio for all the branches) is also indicated. **f**, Log likelihood ratio test between model 2 and the null model and between model 1 and model 2. The free-ratio model was the most likely model and significantly better than the two-ratio models. The best two-ratio model tested, and which is significantly better than the null model, is the two-ratio model where birds evolve under a more elevated ω ratio (ω=0.47) compared to all other branches (ω=0.15). **g**, Phylogenetic tree used in RELAX. Bird branches, colored in orange, were assigned as test branches while all other branches (blue) were assigned as reference. **h**, Results of the RELAX analysis. Significant relaxation of selection (K < 1, *P*-value < 0.05) was detected in the bird clade. Source data are provided as a Source Data file.

a

b

c

d

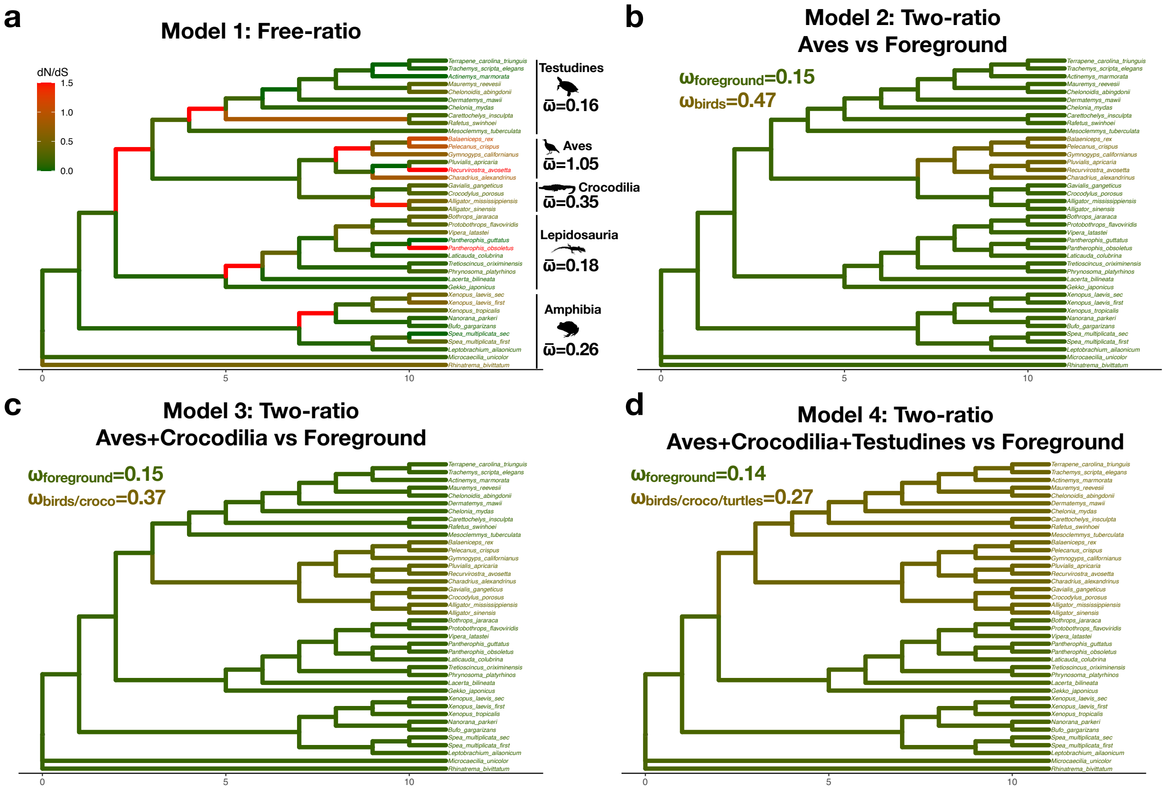

e

Model	np	Lnl
Model 1	155	-15723.48011
Model 2	80	-15860.67583
Model 3	80	-15862.20789
Model 4	80	-15872.18150
Null model (ω=0.176)	79	-15899.00447

f

Models compared	LRT	df	p-value
Model 2 vs Null model	76.66	1	2E-18
Model 1 vs Model 2	274.39	75	1.6E-24

g

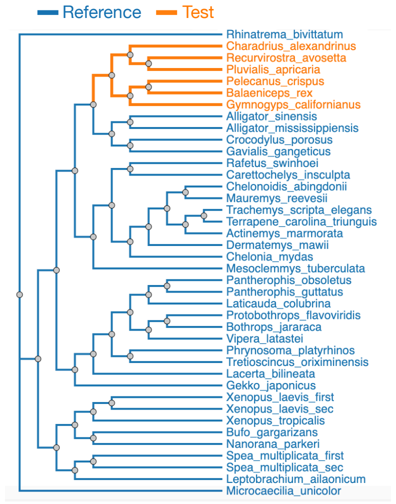

h

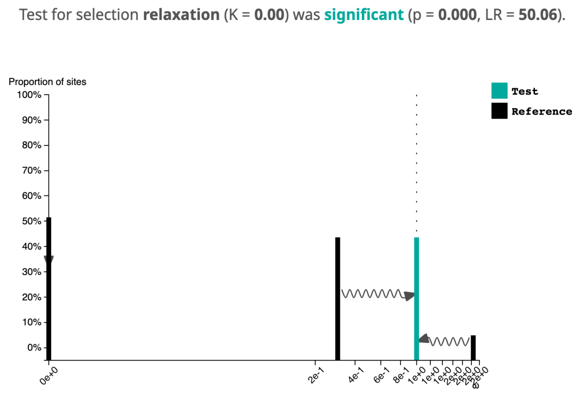

Supplementary Fig. 27 | Phylogenies showing the number of complete *V2R* genes. Vertebrate phylogeny and (sub)class phylogenies displaying the number of complete *V2R* genes (BUSCO80 dataset). **a**, Vertebrata; **b**, Agnatha; **c**, Chondrichthyes; **d**, Actinopterygii; **e**, Amphibia; **f**, Mammalia; **g**, Lepidosauria; **h**, Testudines; **i**, Aves and Crocodilia. Terminal branches and species names are colored according to (sub)class (**a**) or according to order based on the NCBI taxonomy database (**b-i**). *V2R*-subclades are indicated. Source data are provided as a Source Data file.

a) Vertebrates

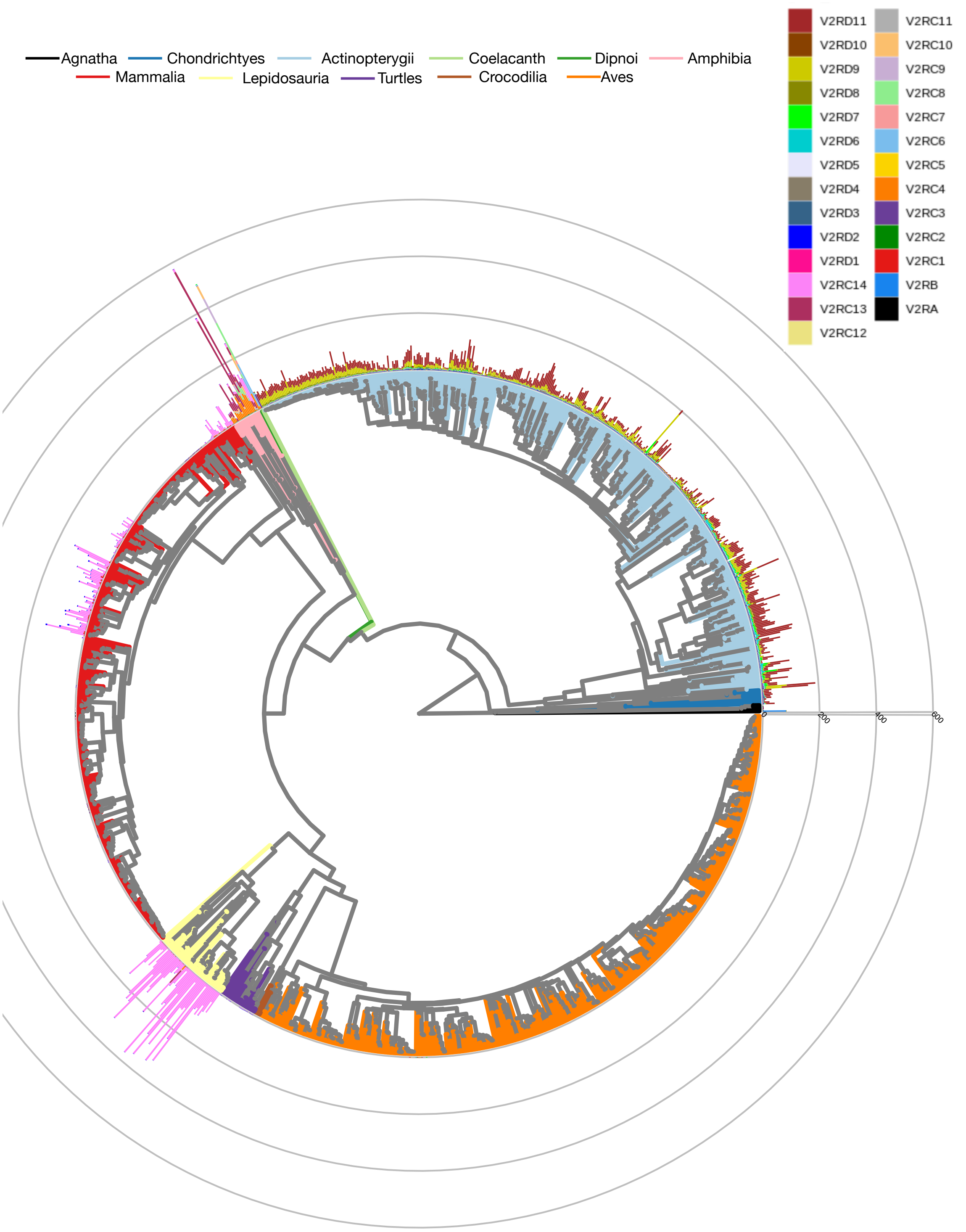

b) Agnatha

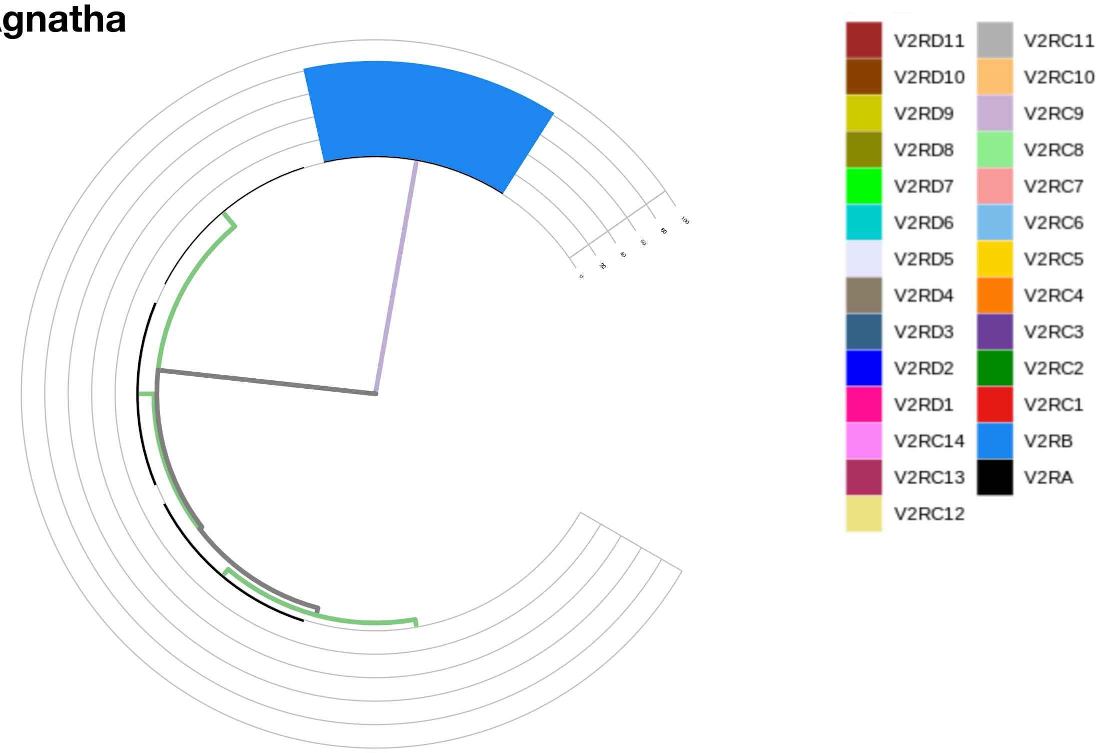

c) Chondrichthyes

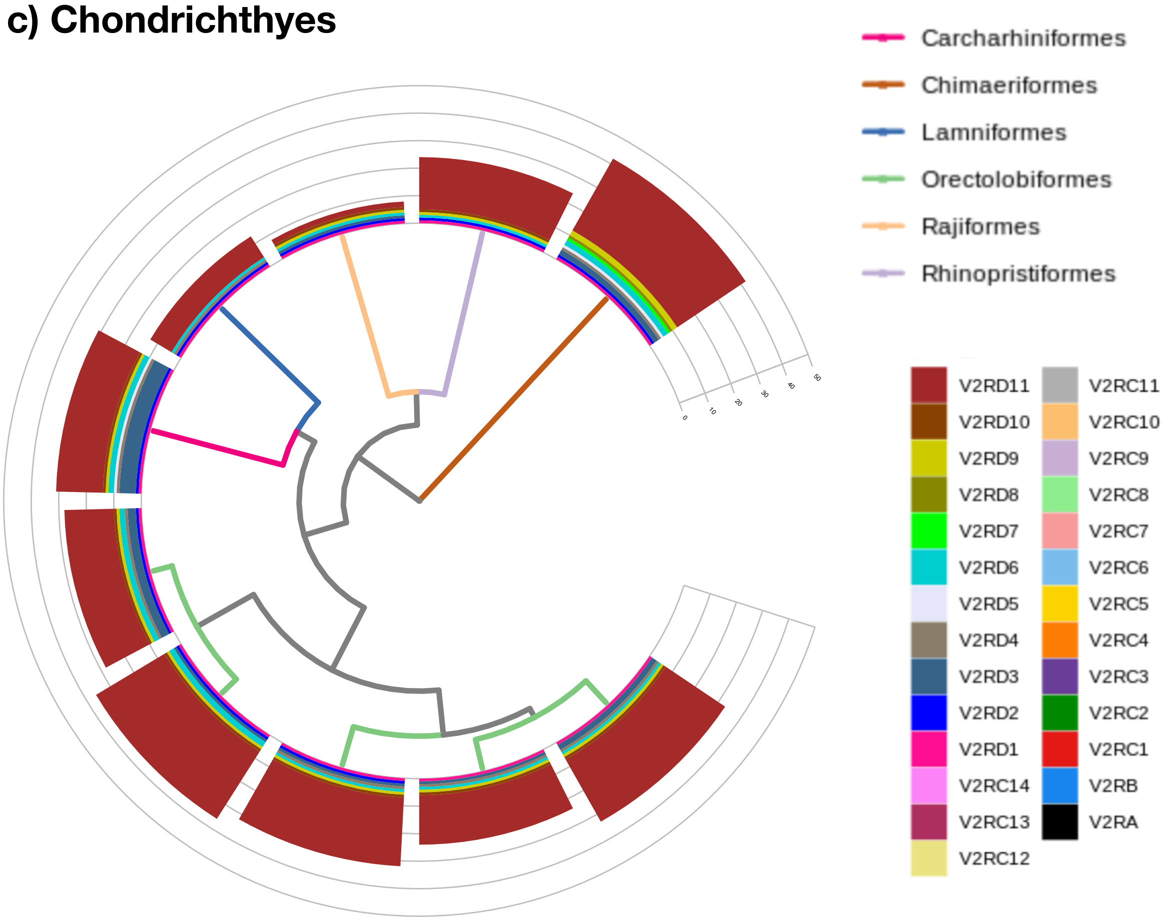

d) Actinopterygii

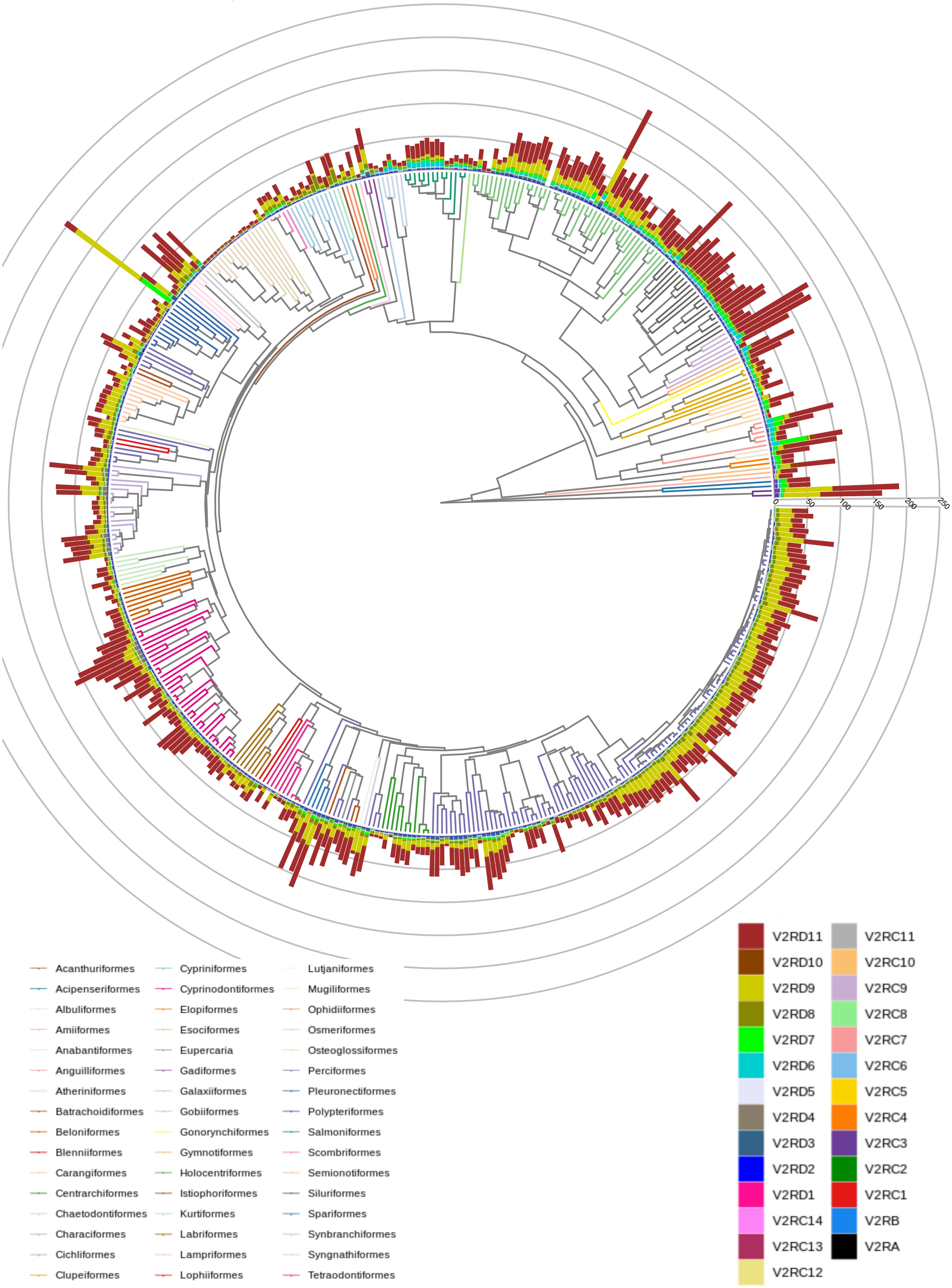

e) Amphibia

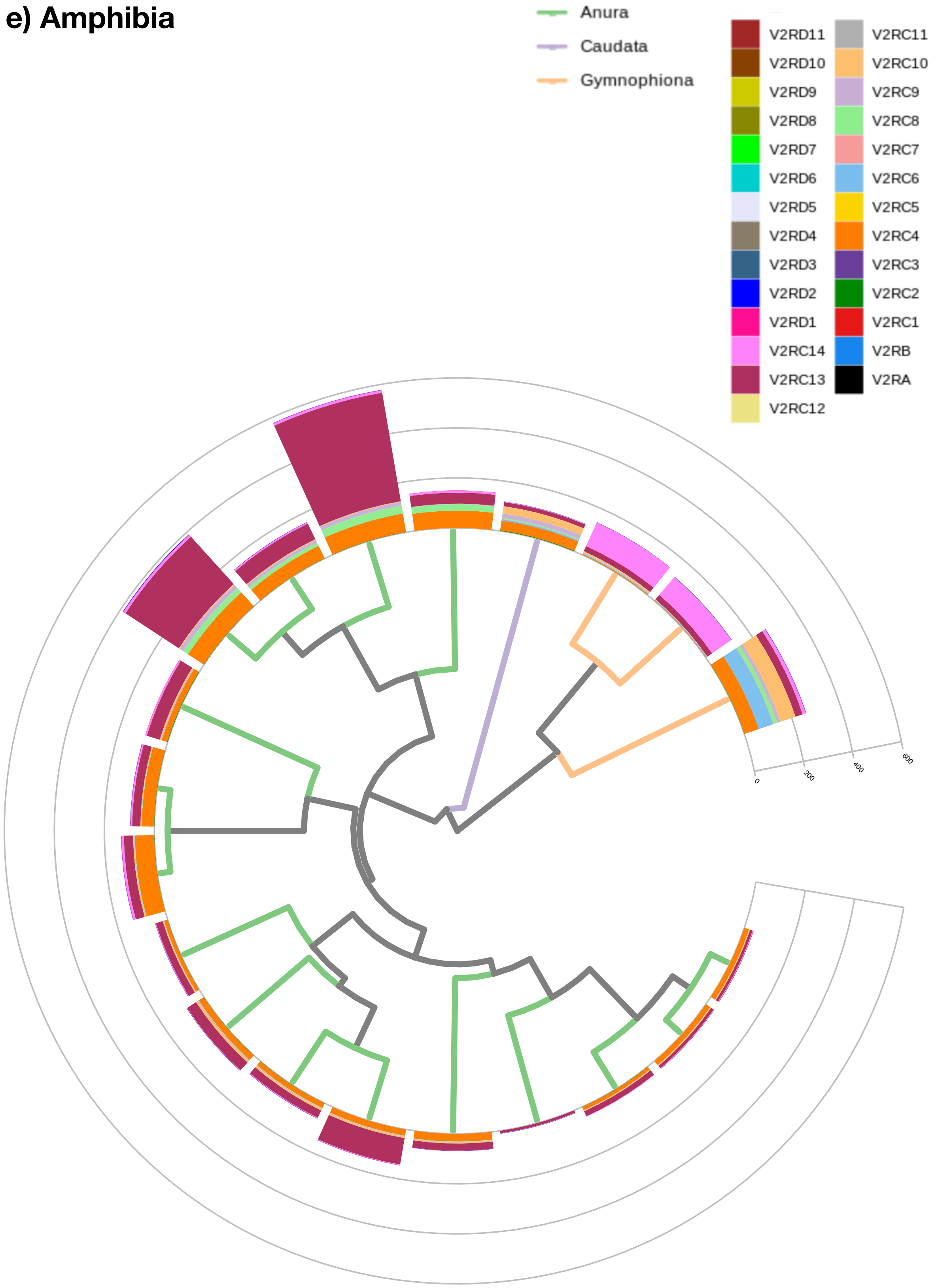

Anura
Caudata
Gymnophiona
V2RD11
V2RD10
V2RD9
V2RD8
V2RD7
V2RD6
V2RD5
V2RD4
V2RD3
V2RD2
V2RD1
V2RC14
V2RC13
V2RC12
V2RC11
V2RC10
V2RC9
V2RC8
V2RC7
V2RC6
V2RC5
V2RC4
V2RC3
V2RC2
V2RC1
V2RB
V2RA
0
200
400
600

f) Mammalia

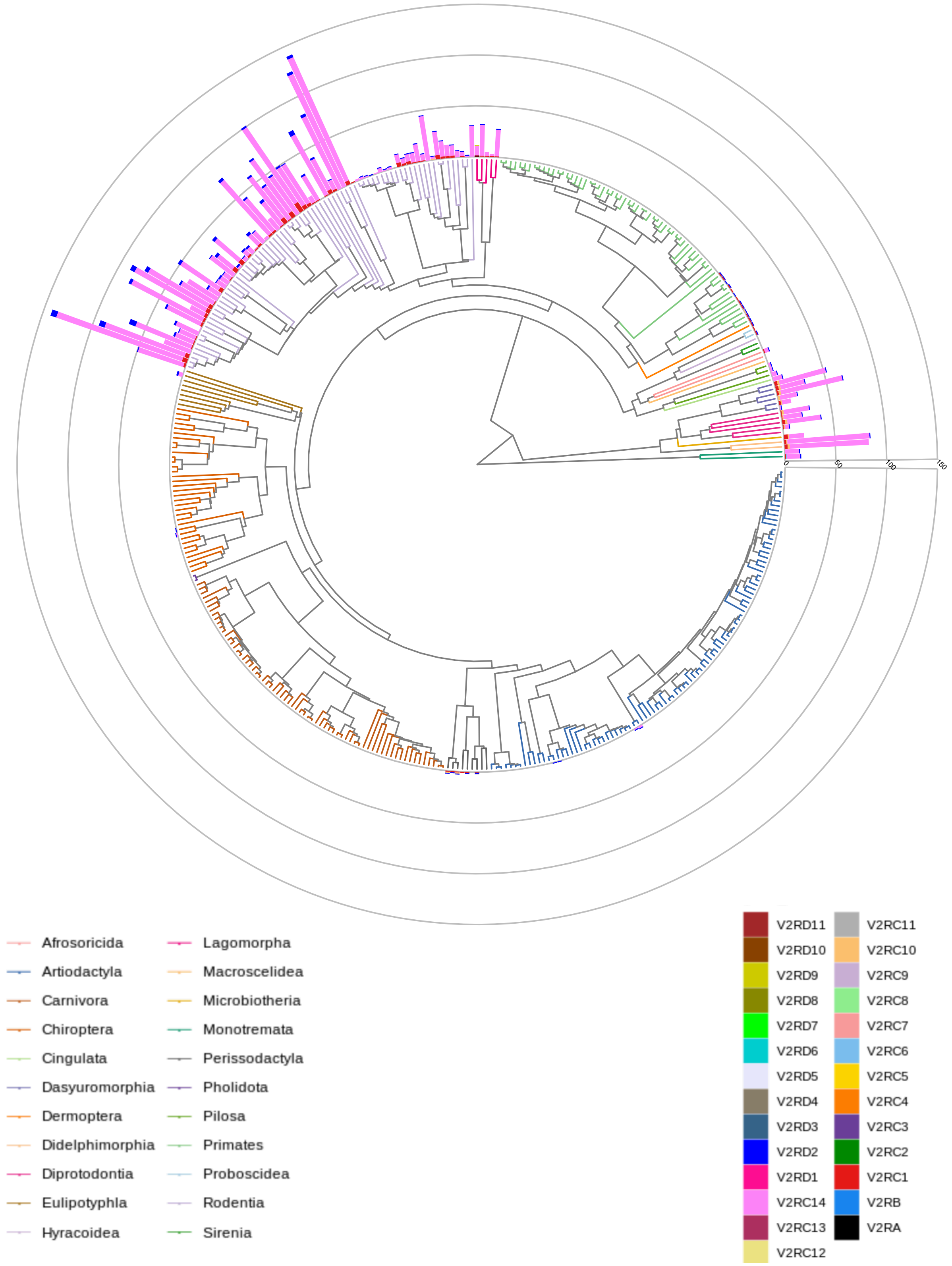

0
50
100
150
Afrosoricida
Artiodactyla
Carnivora
Chiroptera
Cingulata
Dasyuromorphia
Dermoptera
Didelphimorphia
Diprotodontia
Eulipotyphla
Hyracoidea
Lagomorpha
Macroscelidea
Microbiotheria
Monotremata
Perissodactyla
Pholidota
Pilosa
Primates
Proboscidea
Rodentia
Sirenia
V2RD11
V2RD10
V2RD9
V2RD8
V2RD7
V2RD6
V2RD5
V2RD4
V2RD3
V2RD2
V2RD1
V2RC14
V2RC13
V2RC12
V2RC11
V2RC10
V2RC9
V2RC8
V2RC7
V2RC6
V2RC5
V2RC4
V2RC3
V2RC2
V2RC1
V2RB
V2RA

g) Lepidosauria

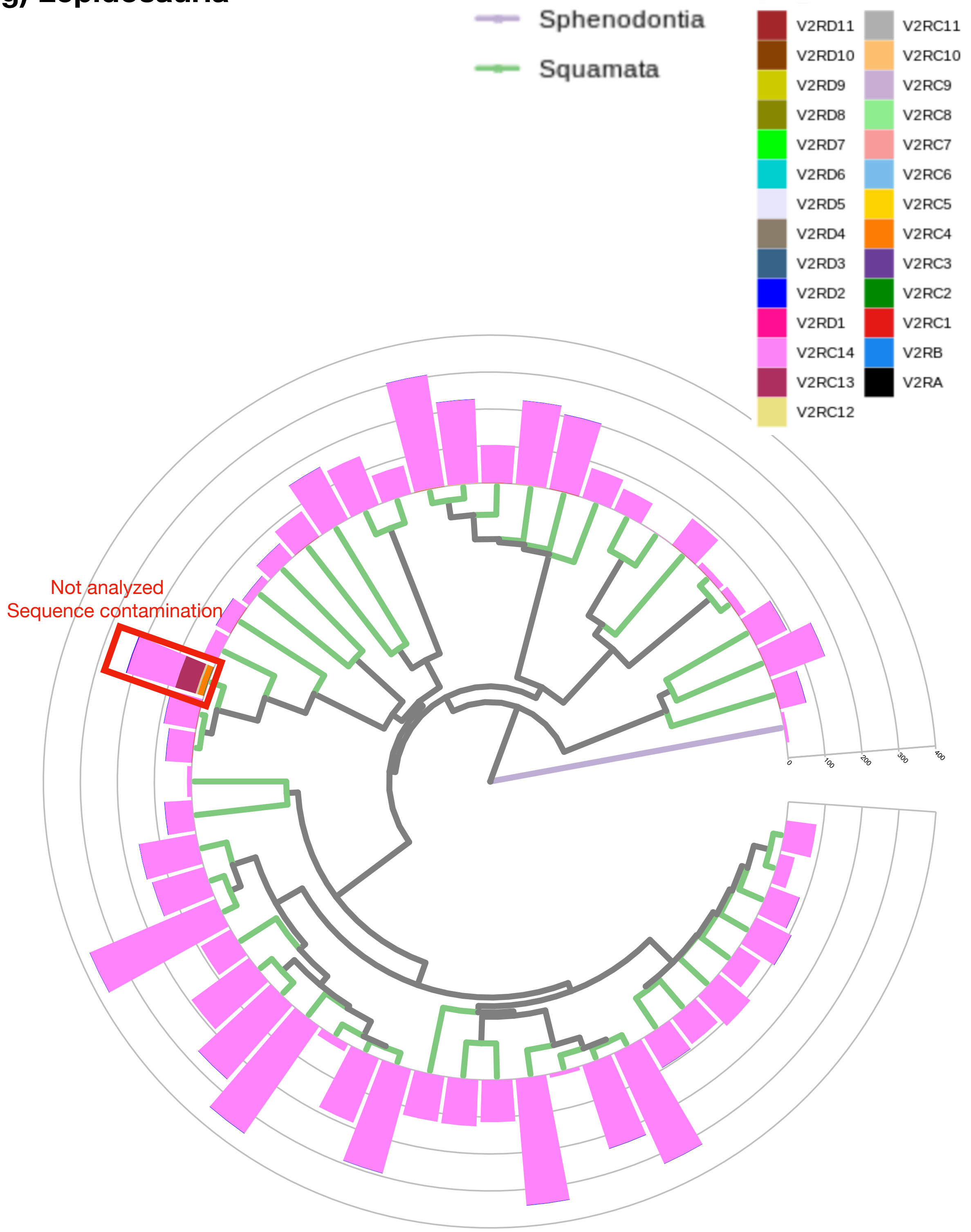

h) Turtles

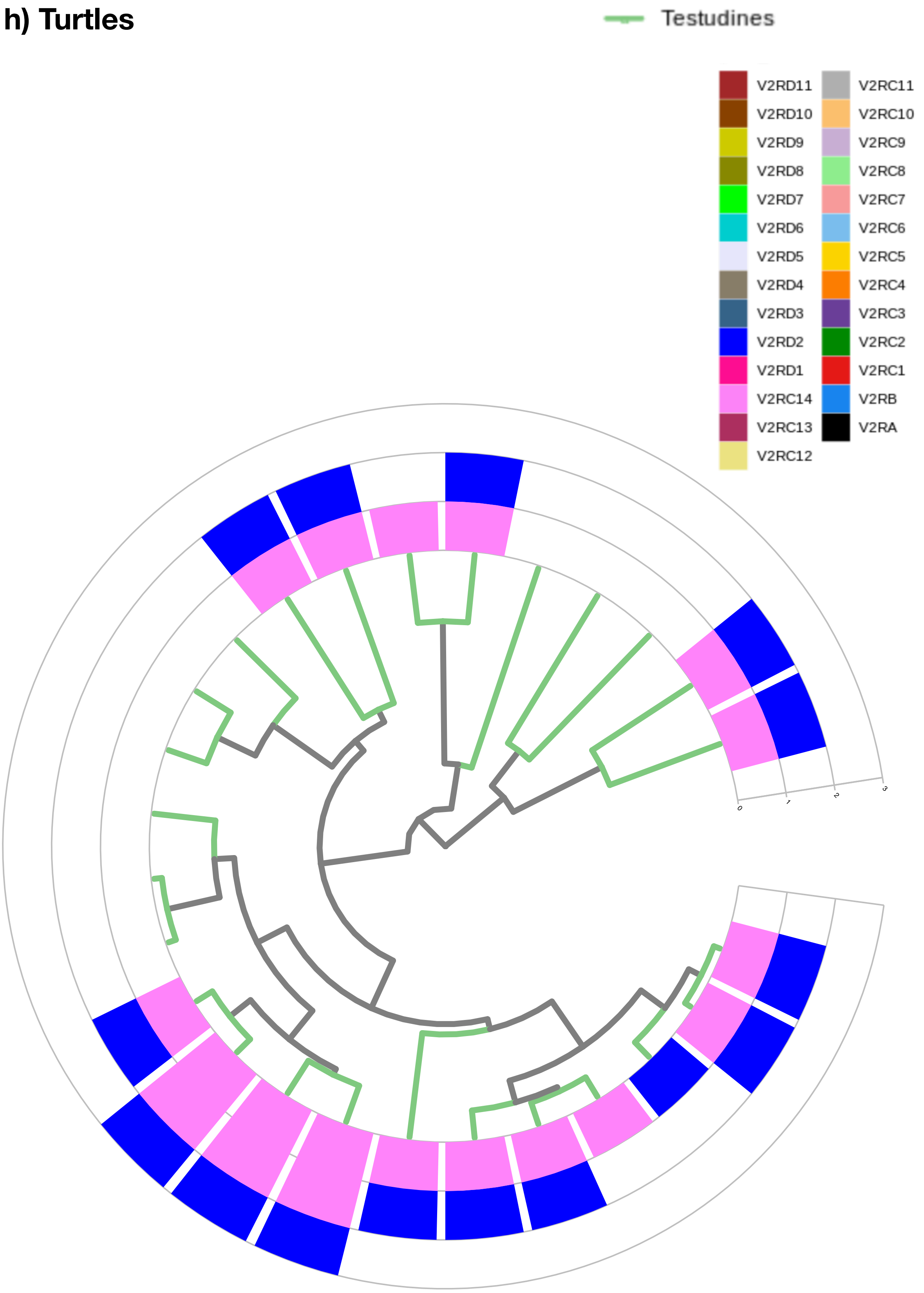

Testudines
V2RD11
V2RD10
V2RD9
V2RD8
V2RD7
V2RD6
V2RD5
V2RD4
V2RD3
V2RD2
V2RD1
V2RC14
V2RC13
V2RC12
V2RC11
V2RC10
V2RC9
V2RC8
V2RC7
V2RC6
V2RC5
V2RC4
V2RC3
V2RC2
V2RC1
V2RB
V2RA
0
1
2
3

i) Aves (with Crocodilia as outgroup)

No V2R genes in birds and crocodiles

Supplementary Fig. 28 | Vertebrate *V2R* gene tree. a, Near maximum likelihood phylogeny of *V2R* genes of vertebrates with branches colored according to the vertebrate (sub)class. **b**, Clade tree generated from (**a**) with local support values indicated at each node (according to a Shimodaira-Hasegawa test). The *V2R* gene tree was consistent with previous studies, in particular the one by Zhang et al.[26]. As our extensive species sample allowed us to define new clades that were only partially or not at all described in previous studies, we also renamed all these clades. Thus, the formerly described *t-V2R* (tetrapods-*V2R*) clade was split into fourteen clades named *V2RC1* to *V2RC14*, while the former *f-V2R* (fish-*V2R*; that is, *OlfC*) clade was split into eleven clades, named *V2RD1* to *V2RD11*. The tree in newick version, as well as all sequences and the alignment file can be found in FigShare. Sub-trees of each vertebrate (sub)class with the number of *V2R* genes are shown in Supplementary Fig. 27. Maximum likelihood phylogeny of *V2R* genes extracted from one species per vertebrate (sub)class is shown in Supplementary Fig. 29-30.

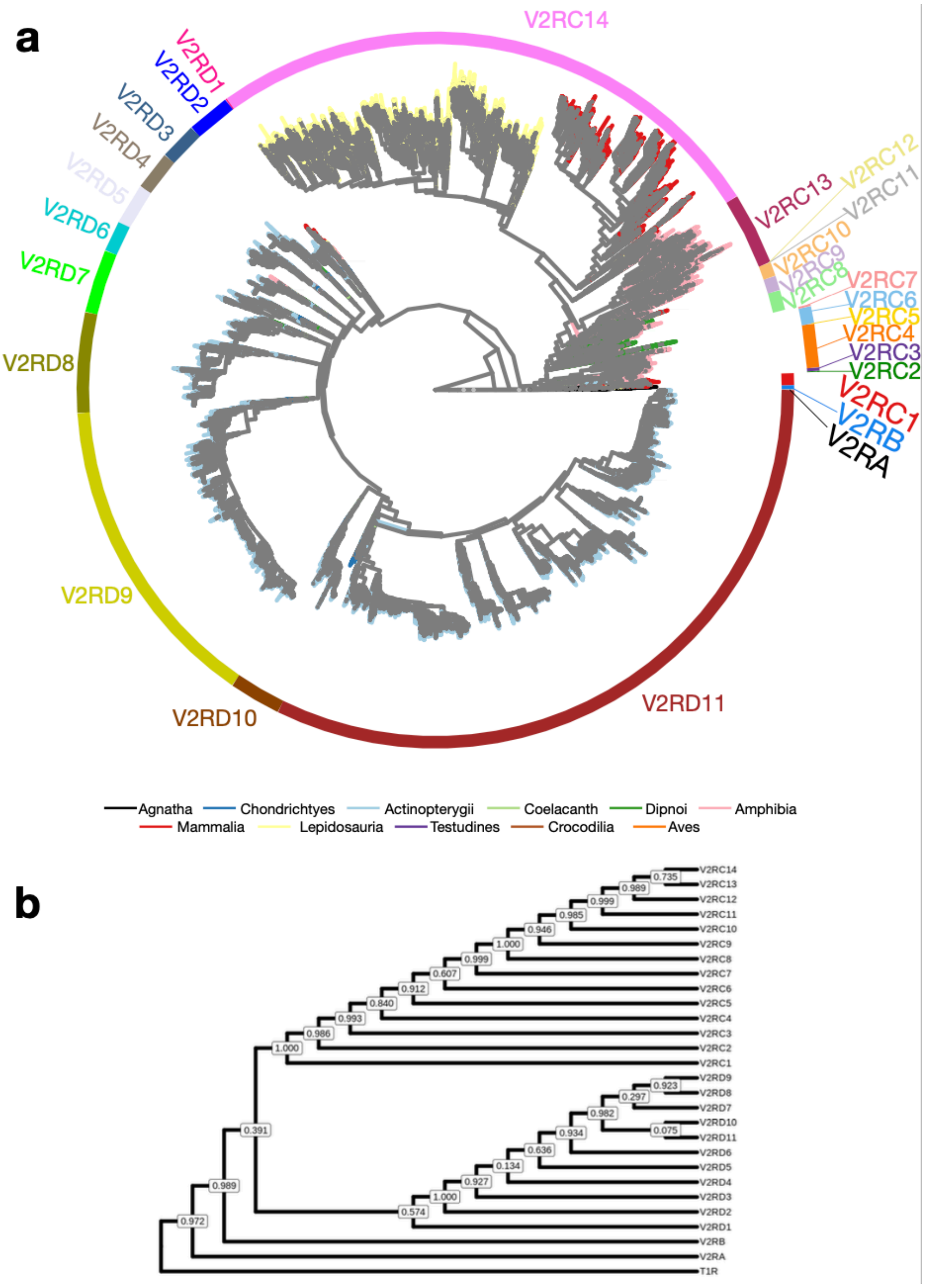

Supplementary Fig. 29 | Maximum likelihood phylogeny of *V2R* genes. Maximum likelihood phylogeny of *V2R* genes extracted from one representative species per vertebrate subclass. Branches are colored according to the vertebrate (sub)class. *V2R* subclades are indicated. *V2RA* is directly indicated inside the phylogenetic tree as there was only one sequence in this clade. The tree was computed using the optimal model found by ModelFinder (JTT+F+R10). The robustness of the nodes was evaluated with 1,000 ultrafast bootstraps and these bootstrap values are reported for each node in Supplementary Fig. 30. *V2RA*, *V2RB* and *V2RC* subclades retrieved in the near-ML topology as well as their relations were retrieved in this ML topology. At the contrary, the *V2RD2* clade, which was in the *V2RD* clade in the near-ML topology, is now the sister clade of all *V2RC* and other *V2RD* genes. When looking at clades that diverged more recently, we also retrieved some differences compared to the near-ML topology, especially for *VRRC8* which is paraphyletic in the ML topology and divided in four different clades.

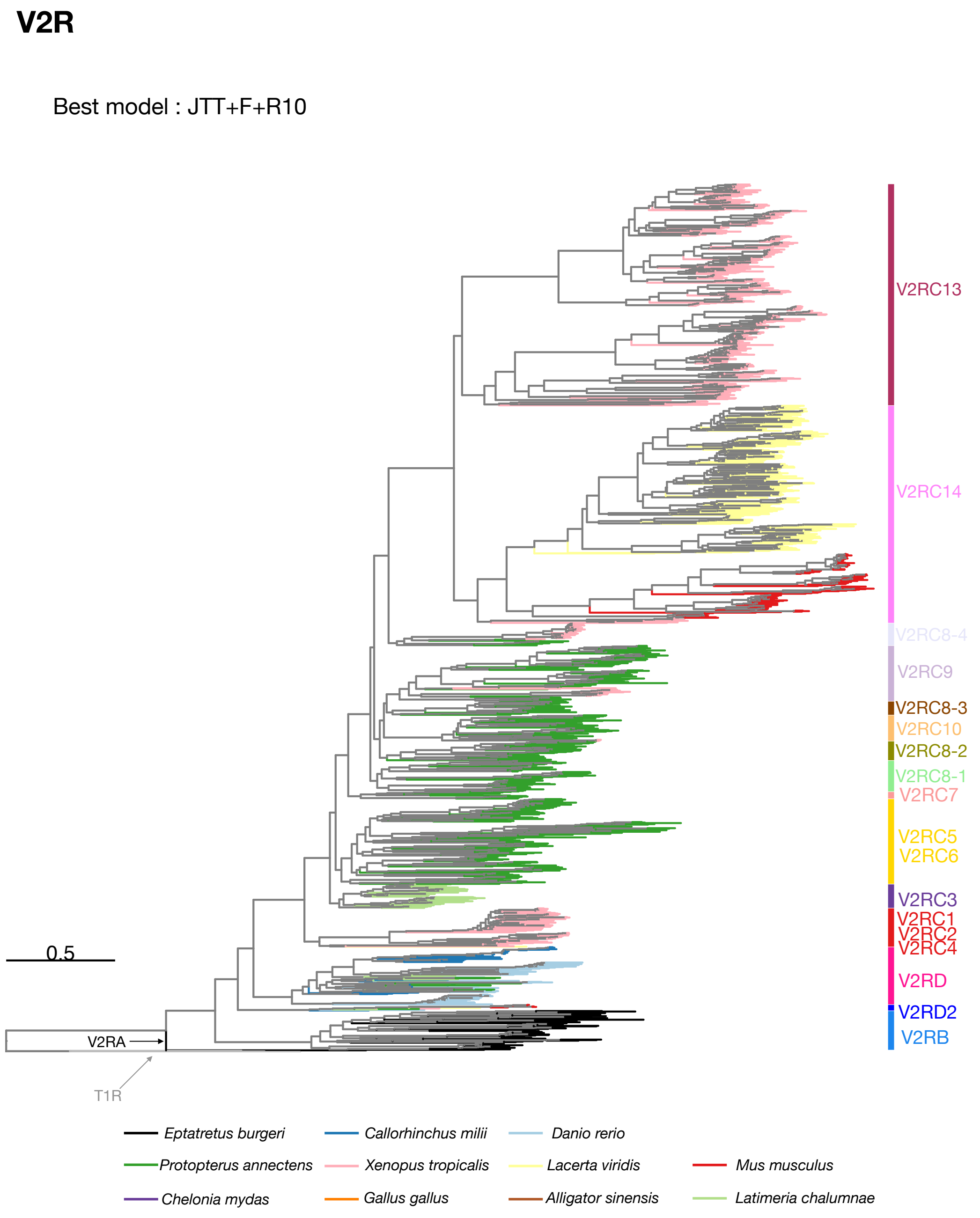

Supplementary Fig. 30 | Maximum likelihood phylogeny of *V2R* genes. Same maximum likelihood phylogeny of *V2R* genes as in Supplementary Fig. 29 but with bootstrap values indicated at each node.

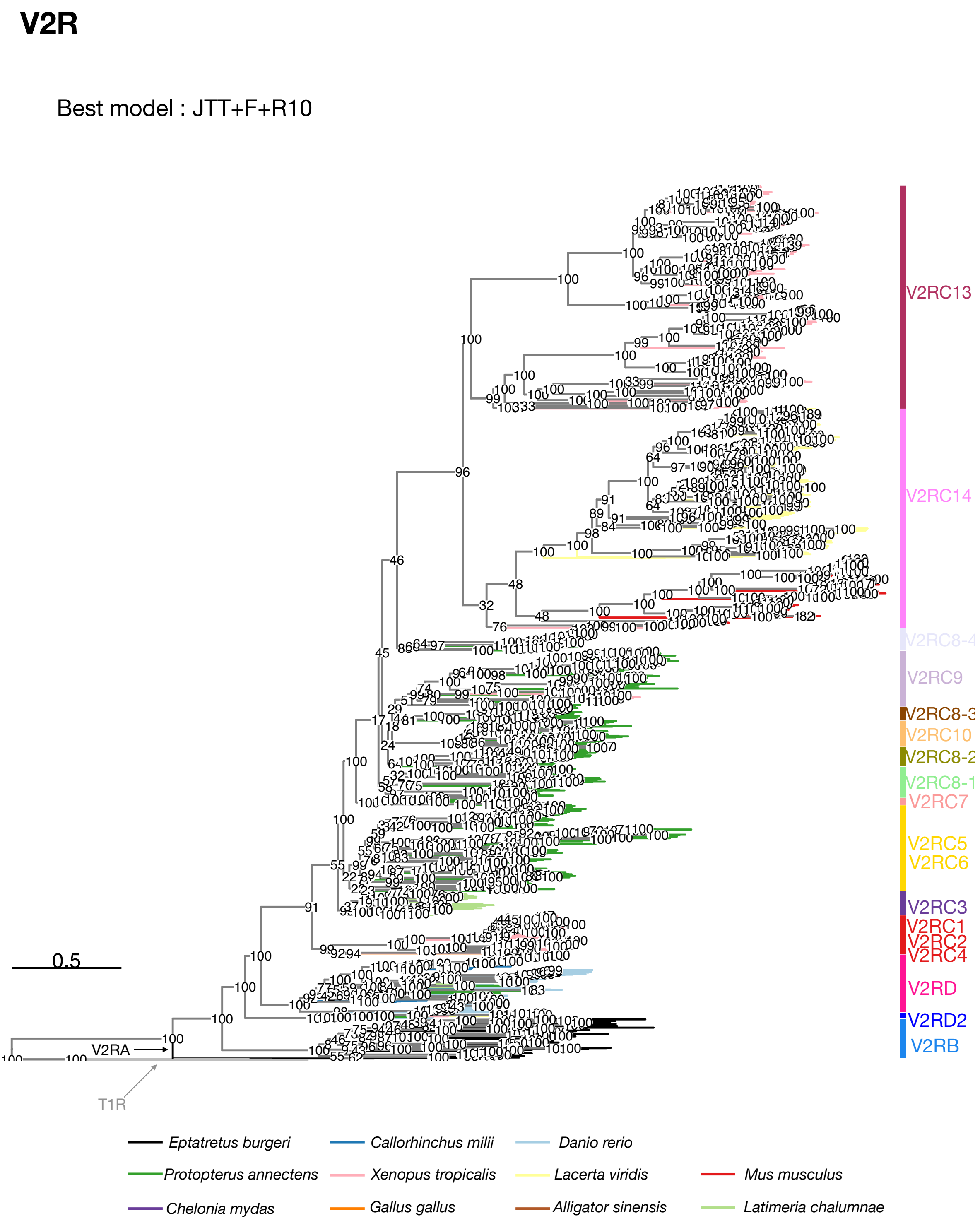

Supplementary Fig. 31 | Co-evolution of the *V1R* gene repertoire and the accessory olfactory bulb (AOB). **a**, Phylogeny of bat species included in our dataset, for which the information on the AOB was available from the literature (absence: light blue; presence: red). The number above each branch corresponds to the difference between the number of *V1R* genes gained and the number of *V1R* genes lost (inferred from a gene tree - species tree reconciliation method). Assuming that a loss of the AOB is not reversible, the phylogeny suggests at least four independent losses, indicated by light blue stars. **b**, Box-plots (first quartile - 1.5; the interquartile range, the first quartile, the mean, the third quartile and the third quartile + 1.5 interquartile range. Dots represent outliers.) showing the number of compete *V1R* genes in bats with (red) and without (blue) an AOB (Two-sided pGLS test. P_{PGLS}; n = 15, P = 1.775e-05). **c**, Different model tested with PAML. The Free ratio model (M3) assumes a different ω per branch. The one ratio model (M1) assumes the same ω ratio for every branch. In the two-ratio model, one ω ratio was assigned to terminal branches corresponding to *V1R* genes of bats lacking an AOB (indicated by blue arrows in **e**), and one ω ratio was assigned to all other branches. "Lnl" corresponds to the likelihood score of each model, and "np" corresponds to the number of parameters. **d**, Comparison between the three models. LRT corresponding the likelihood ratio test statistic, and the p-value was computed using the number of degrees of freedom (difference in the number of parameters between the two models tested). The free-ratio model was significantly better than the two-ratio model and the one ratio model, and the two-ratio model was not significantly better than the one ratio model. **e**, *V1R* gene tree of bats. Branches are colored according to their dN/dS value computed under the free ratio model. The ω ratio computed under the one ratio model is indicated on the top left (ω = 0.7), as well as the ω ratio for *V1R* genes of bats lacking an AOB (ω = 0.77) and the ω ratio of all other branches (ω = 0.69) under the two-ratio model. Blue arrows indicate terminal branches corresponding to *V1R* genes of bats lacking an AOB. **f**, Distribution of ω ratio in terminal branches, under the free ratio model, corresponding to bats with (red) and without (blue) an AOB. **g**, Results of RELAX showing the shift of the ω distribution toward 1 in *V1R* genes of bats lacking an AOB relative to *V1R* genes of bats with an intact AOB. A significant relaxation of selection was detected with RELAX (K = 0.12, p = 0). Source data are provided as a Source Data file.

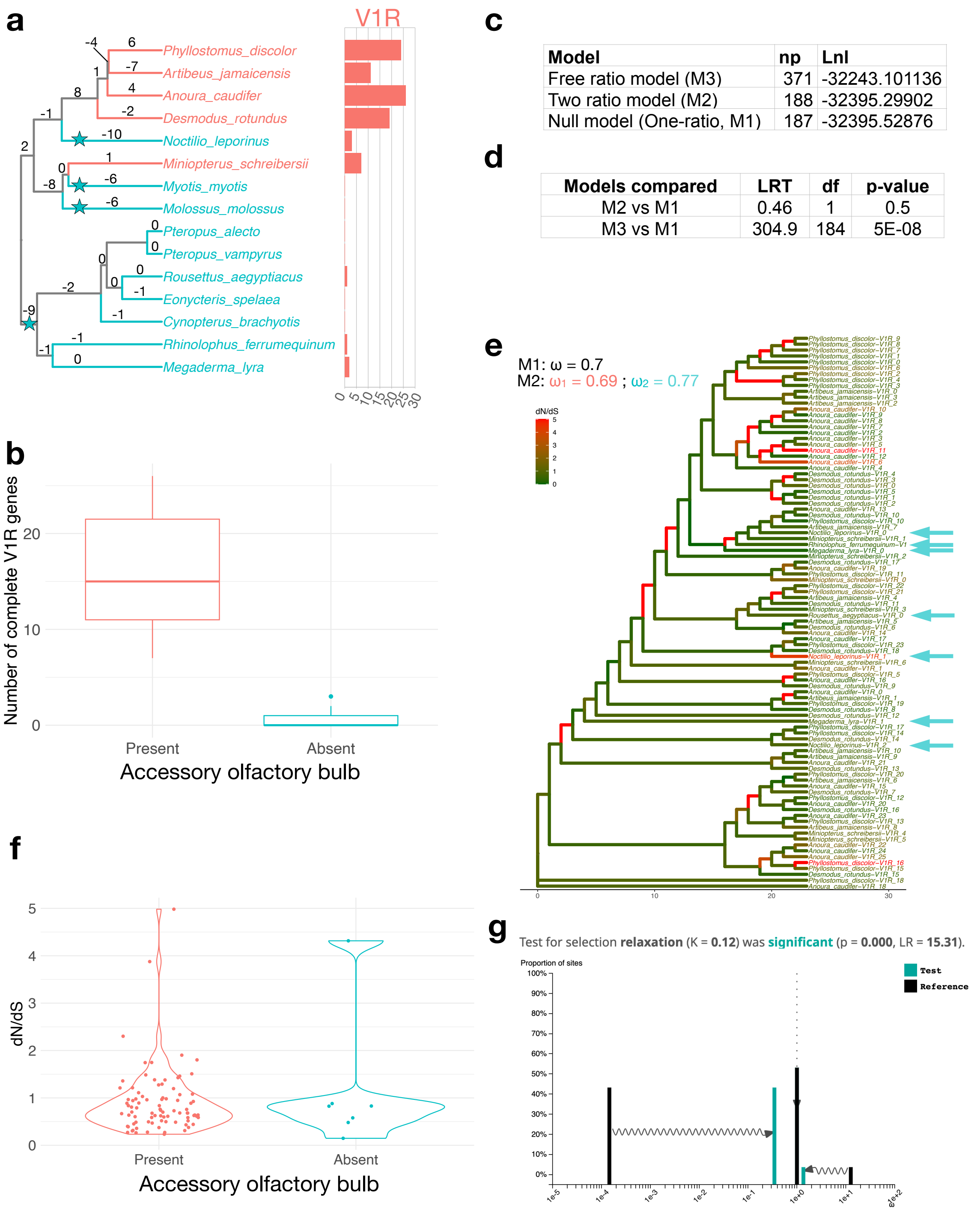

c

Model	np	Lnl
Free ratio model (M3)	371	-32243.101136
Two ratio model (M2)	188	-32395.29902
Null model (One-ratio, M1)	187	-32395.52876

d

Models compared	LRT	df	p-value
M2 vs M1	0.46	1	0.5
M3 vs M1	304.9	184	5E-08

Supplementary Fig. 32 | Phylogenies showing the number of complete *T1R* genes. Vertebrate phylogeny and (sub)class phylogenies displaying the number of complete *T1R* genes (BUSCO80 dataset). **a**, Vertebrata; **b**, Agnatha; **c**, Chondrichthyes; **d**, Actinopterygii; **e**, Amphibia; **f**, Mammalia; **g**, Lepidosauria; **h**, Testudines; **i**, Aves and Crocodilia. Terminal branches and species names are colored according to (sub)class (**a**) or according to order based on the NCBI taxonomy database (**b-i**). *T1R*-subclades are indicated. Source data are provided as a Source Data file.

a) Vertebrates

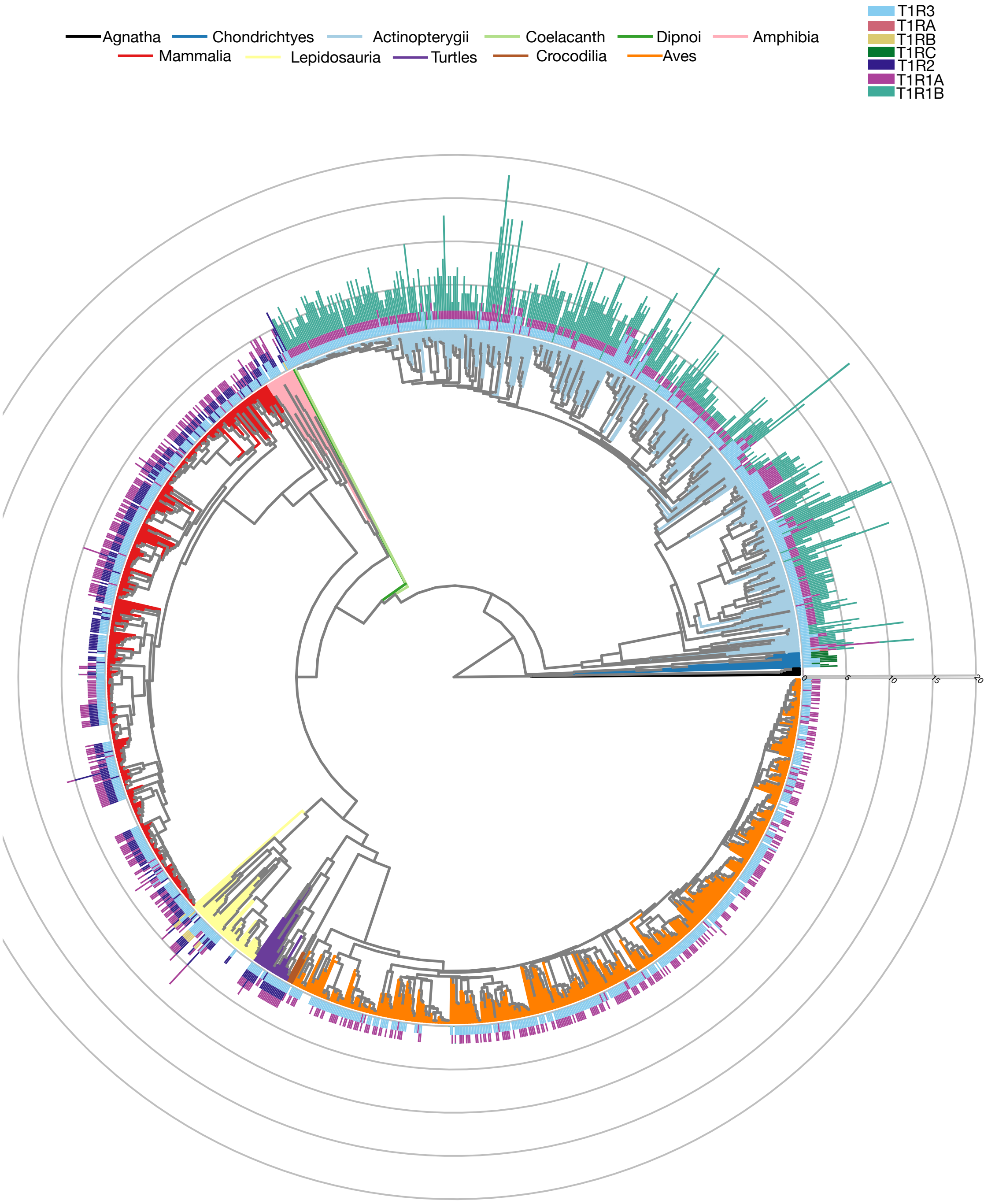

b) Agnatha

No T1R genes in agnathans

c) Chondrichthyes

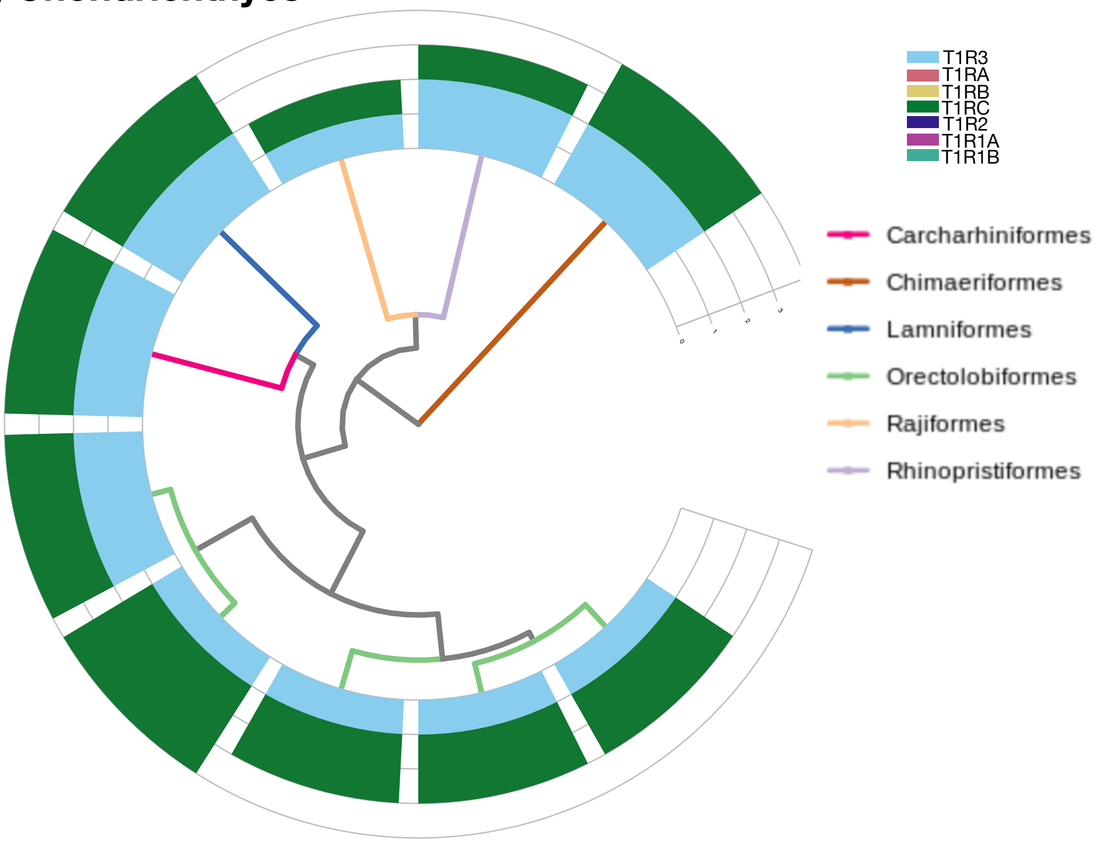

d) Actinopterygii

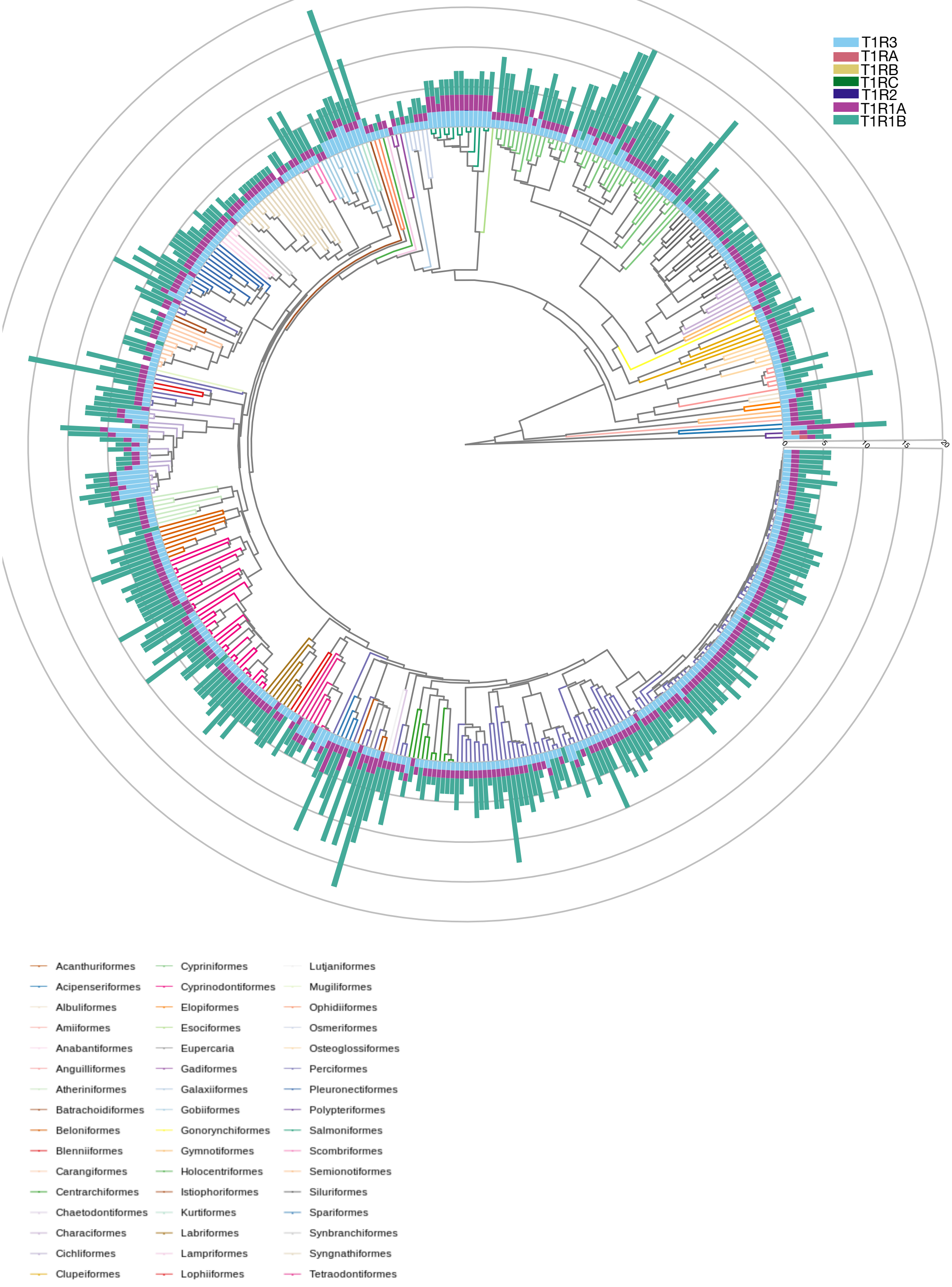

e) Amphibia

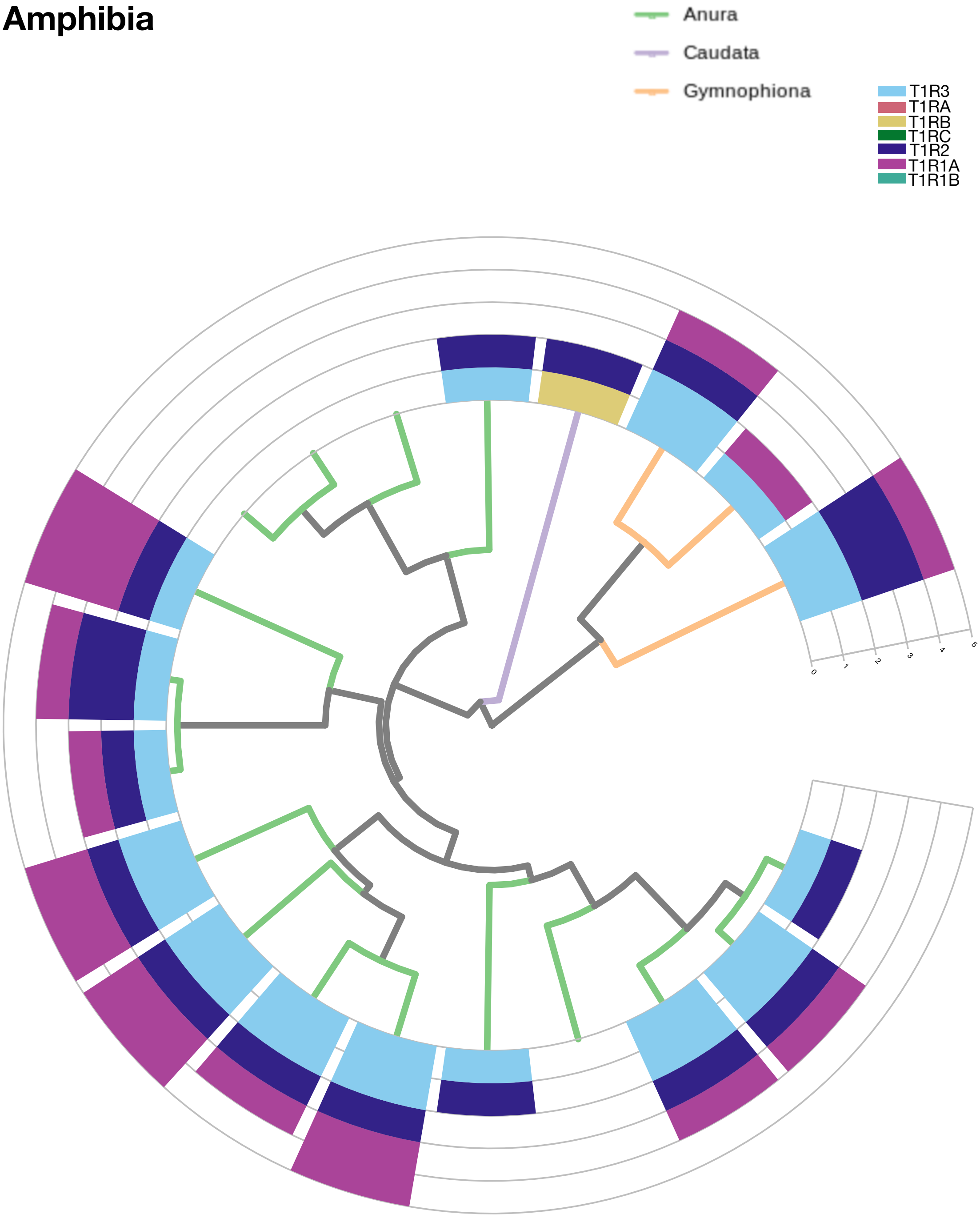
Anura
Caudata
Gymnophiona
T1R3
T1RA
T1RB
T1RC
T1R2
T1R1A
T1R1B

f) Mammalia

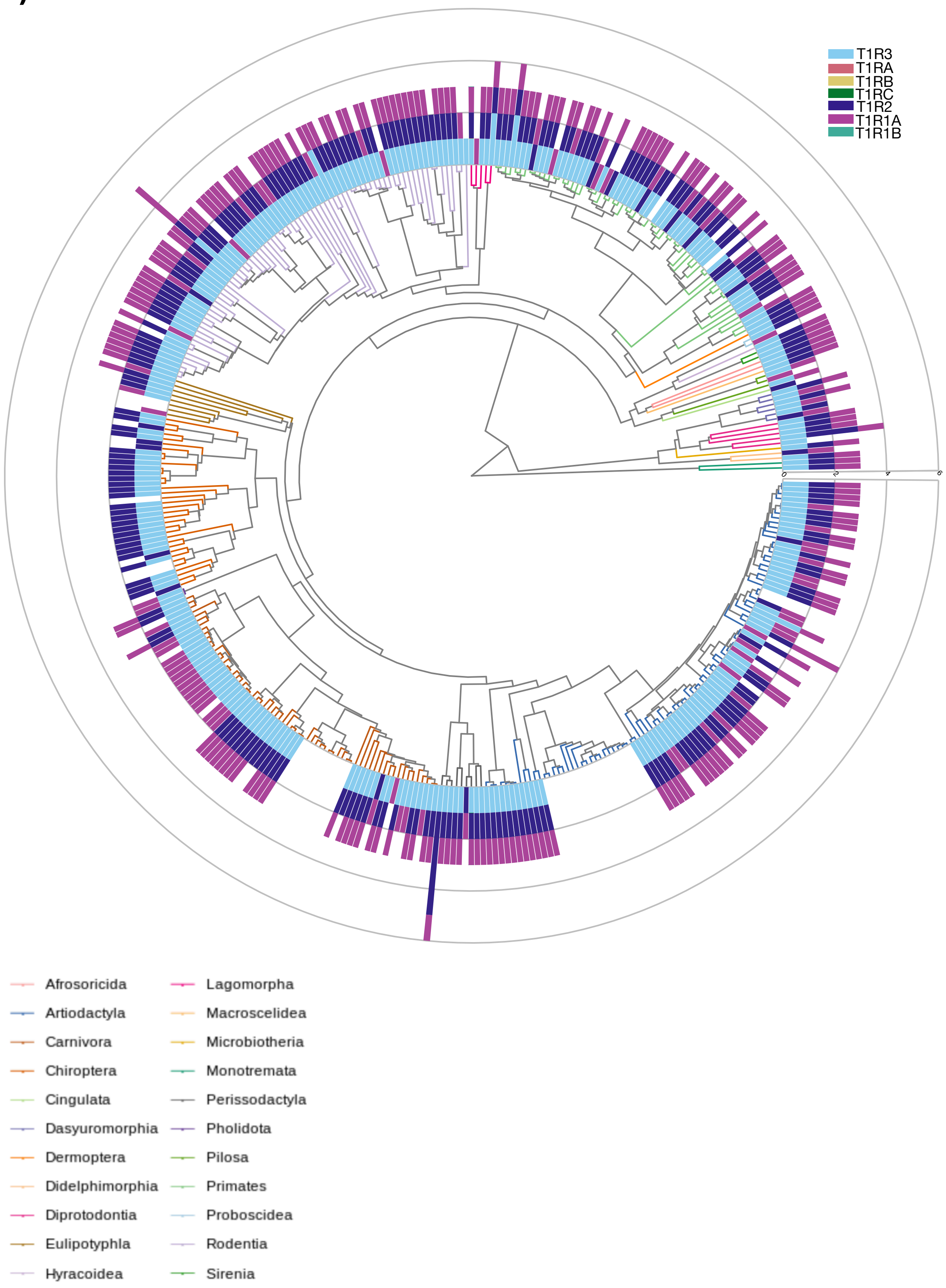
T1R3
T1RA
T1RB
T1RC
T1R2
T1R1A
T1R1B
0
2
4
6
Afrosoricida
Artiodactyla
Carnivora
Chiroptera
Cingulata
Dasyuromorphia
Dermoptera
Didelphimorphia
Diprotodontia
Eulipotyphla
Hyracoidea
Lagomorpha
Macroscelidea
Microbiotheria
Monotremata
Perissodactyla
Pholidota
Pilosa
Primates
Proboscidea
Rodentia
Sirenia

g) Lepidosauria

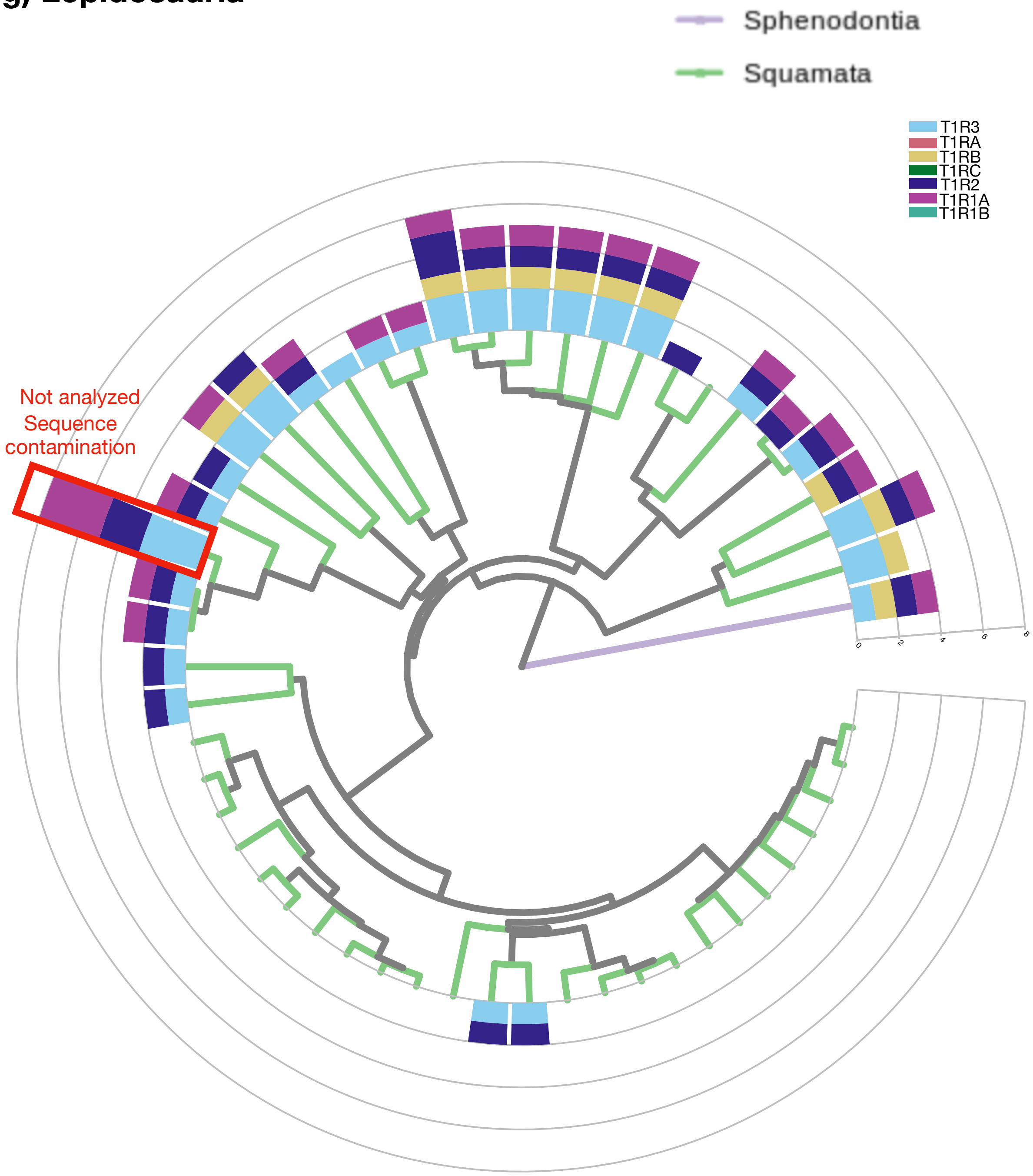

h) Turtles

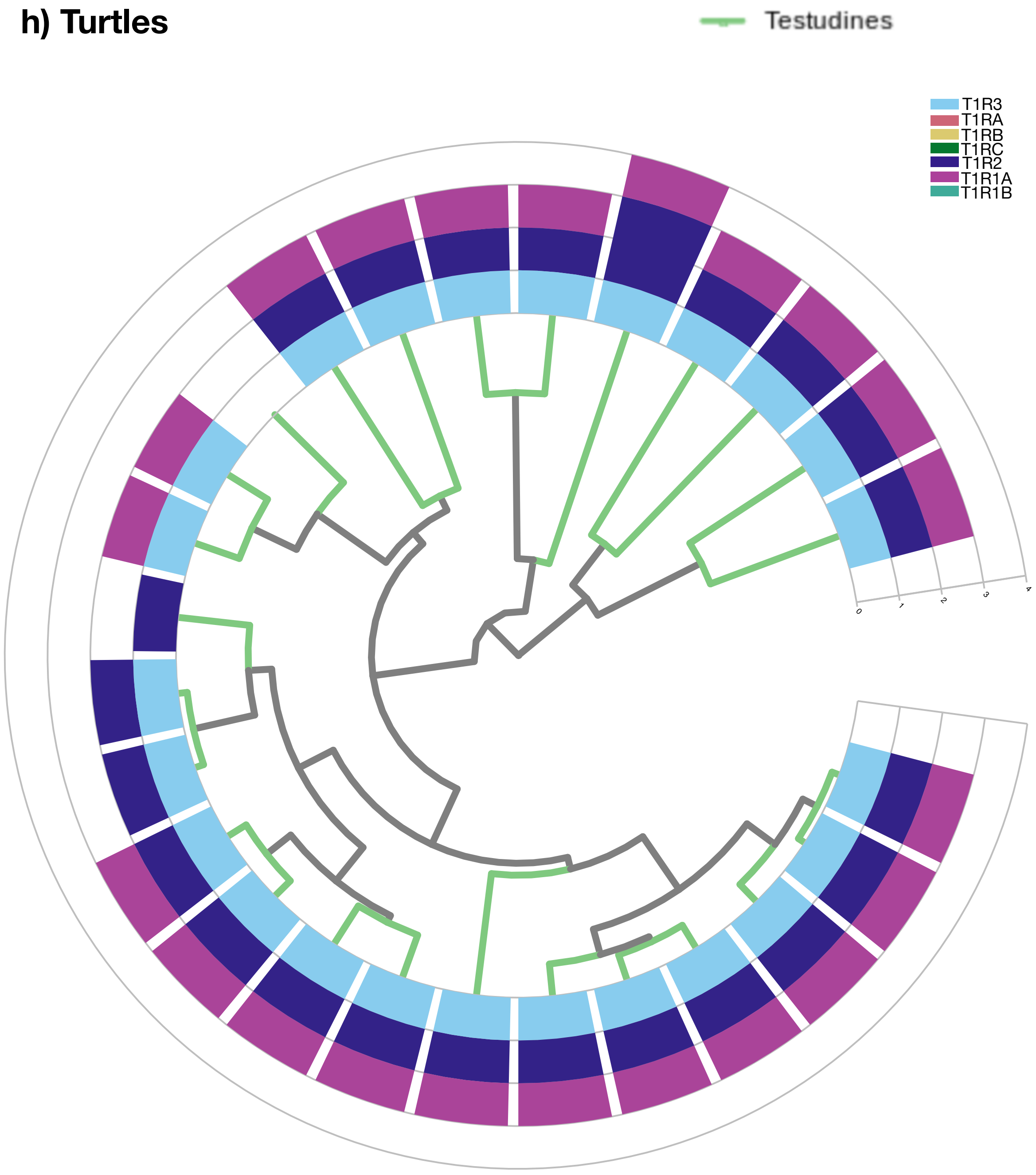

i) Aves (with Crocodilia as outgroup)

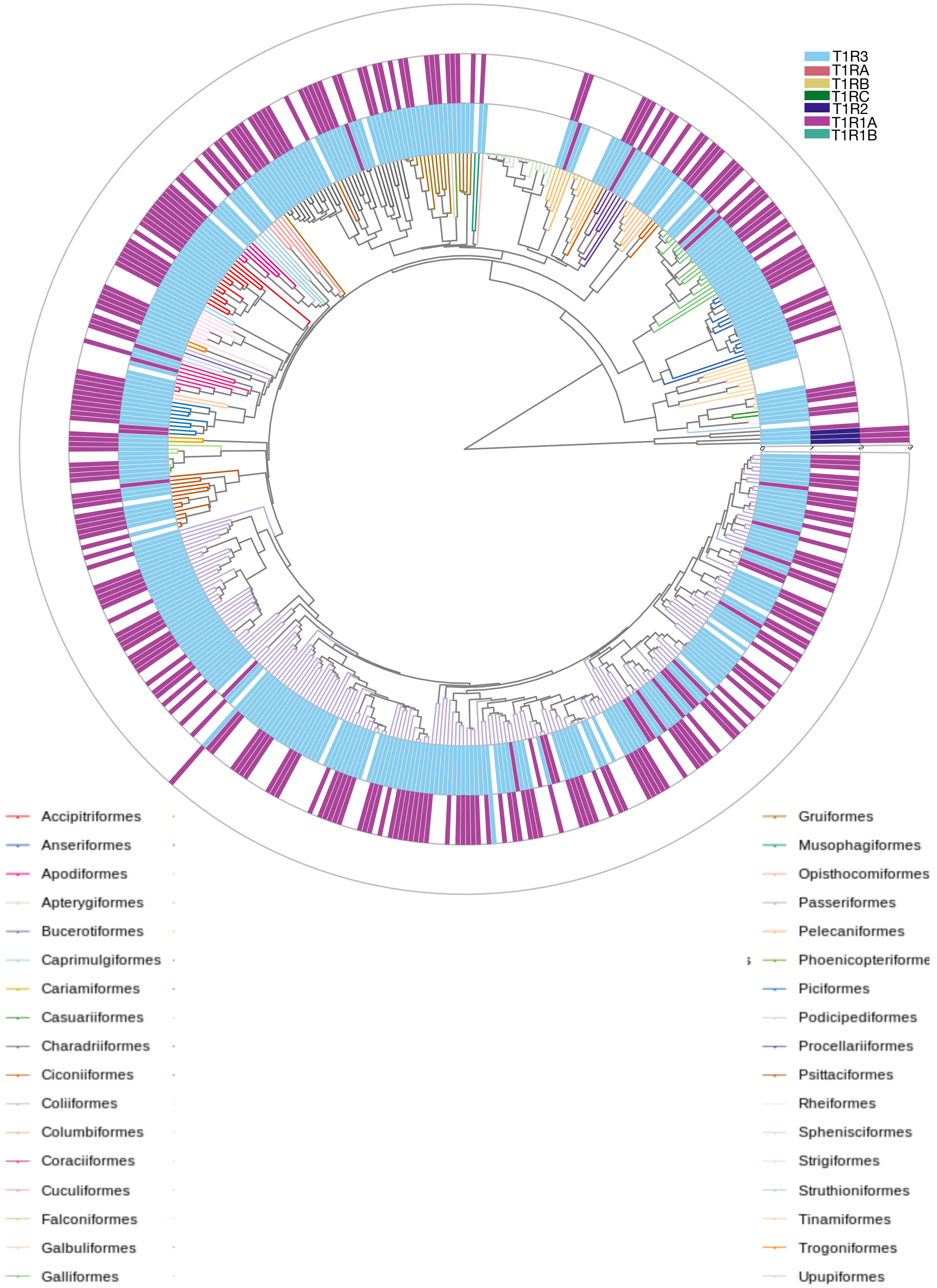

Supplementary Fig. 33 | Vertebrate *T1R* gene tree. **a**, Near maximum likelihood phylogeny of *T1R* genes of vertebrates with branches colored according to the vertebrate (sub)class. **b**, Clade tree generated from (**a**) with local support values indicated at each node (according to a Shimodaira-Hasegawa test). We kept the former clades names *T1R1* (umami receptor subunit), *T1R2* (sweet receptor subunit) and *T1R3*. We also describe here a ray-finned fish specific clade (*T1R1B*), which may be part of *T1R1* or *T1R2* (the support value is too low to distinguish between these scenarios). Furthermore, we found three additional clades: *T1RA*, which is specific to Polypteriformes; *T1RB*, which is specific to amphibians and lepidosaurs; and *T1RC*, which is specific to cartilaginous fishes. Note that the presence of the *T1RC* clade was already suggested by Angotzi et al.[36]. An alternative, but rather similar *T1R* topology has recently been proposed by Nishihara et al[37]. The tree in newick version, as well as all sequences and the alignment file can be found in FigShare. Sub-trees of each vertebrate (sub)class with the number of *T1R* genes are shown in Supplementary Fig. 32. Maximum likelihood phylogeny of *T1R* genes extracted from one species per vertebrate (sub)class is shown in Supplementary Fig. 34.

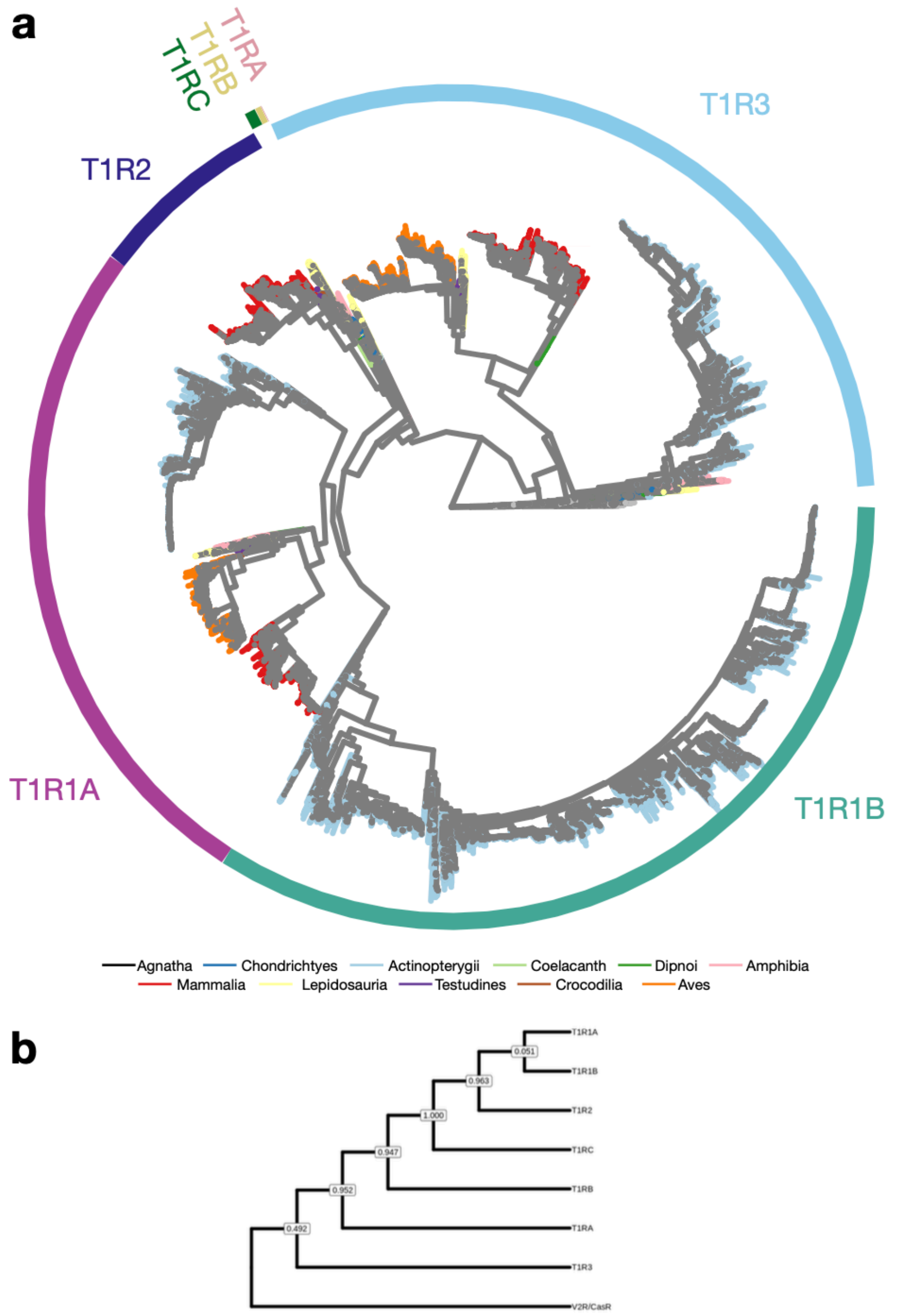

Supplementary Fig. 34 | Maximum likelihood phylogeny of *T1R* genes. Maximum likelihood phylogeny of *T1R* genes extracted from one representative species per vertebrate subclass. Branches are colored according to the vertebrate (sub)class. For ray-finned fishes, two species were sampled: *Danio rerio* and *Polypterus senegalus*. Branches belonging to these species were differentiated by adding "Dr" or "Ps" at the tip of the branches respectively. *T1R* subclades are indicated (*T1RB* and *T1RA* clades were written at the tips of the corresponding branches as there was only one sequence for these clades). The tree was computed using the optimal model found by ModelFinder (LG+R5). The robustness of the nodes was evaluated with 1,000 ultrafast bootstraps and these bootstrap values are indicated at each node. All the clades retrieved using the near-ML method were retrieved as monophyletic, and the exact same topology was retrieved.

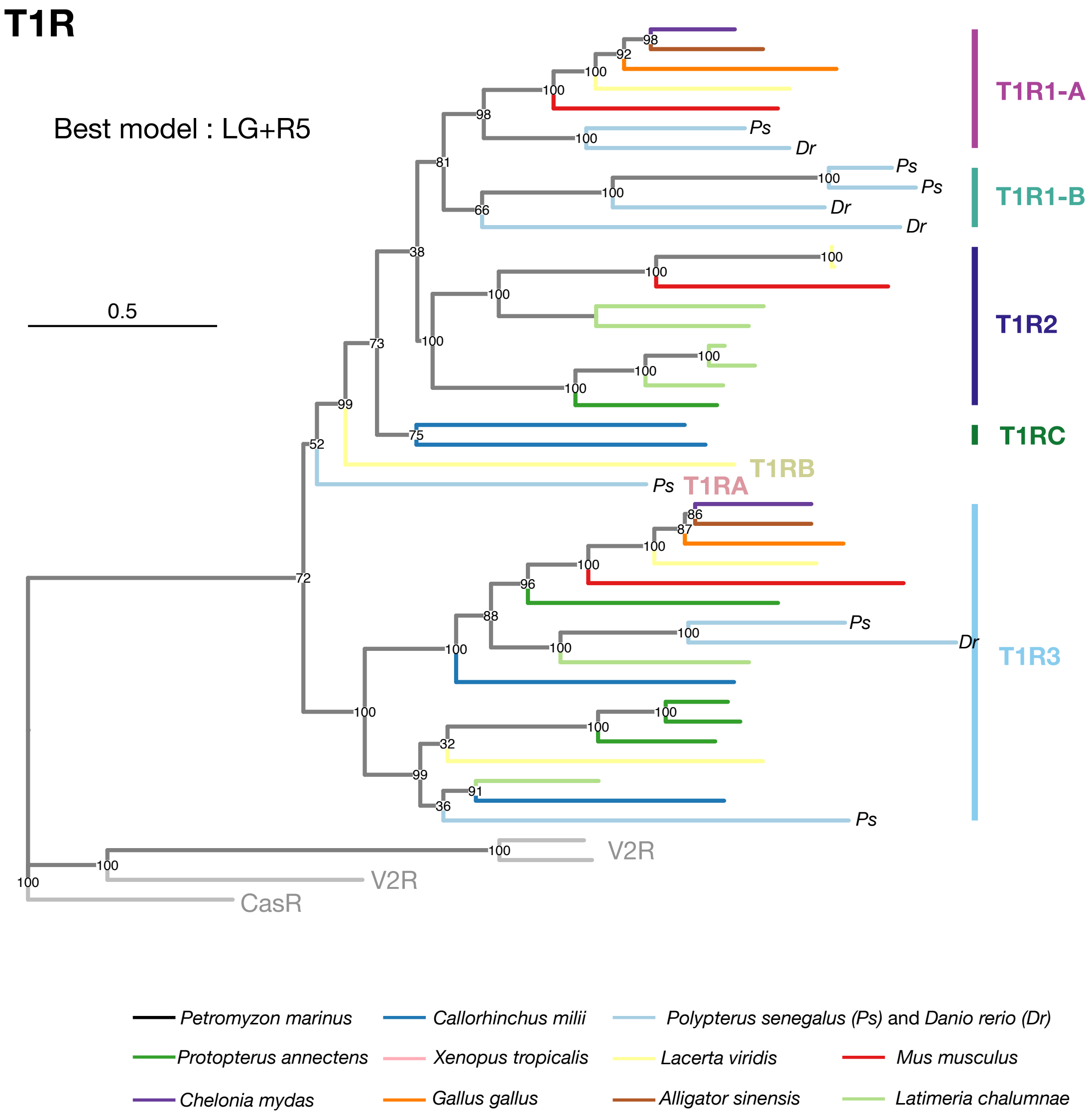

Supplementary Fig. 35 | Evolutionary history of T1R and T2R genes in vertebrates. **a**, Schematic of the T1R gene tree (top) indicating the subclades within the T1R gene family in vertebrates. Gene duplication events leading to subclade divergence are indicated as triangles on the respective branches in the schematic vertebrate phylogeny (left), subclade losses are indicated with the cross symbol below the respective branches. The numbers refer to nodes in the gene tree. Circles on the right indicate the presence of a particular subclade in a given evolutionary lineage, whereby the size of each circle corresponds to the mean number of complete T1R genes per species in the respective subclade and lineage. The T1R gene tree is shown in Supplementary Fig. 33. The T1RA subclade present in non-teleost fish but absent in teleosts is marked with an "*". An alternative, but overall similar, scenario for T1R evolution was proposed by Nishihara et al.[37] **b**, Schematic of the T2R gene tree (top) indicating the subclades within the T2R gene family in vertebrates. Gene duplication events are indicated as triangles on the respective branches in the schematic vertebrate phylogeny (left), subclade losses are indicated with the cross symbol. The numbers refer to nodes in the gene tree. Circles on the right indicate the presence of a particular subclade in a given evolutionary lineage, whereby the size of each circle corresponds to the mean number of complete T2R genes per species in the respective subclade and lineage. The T2R gene tree is shown in Supplementary Fig. 42. Source data are provided as a Source Data file.

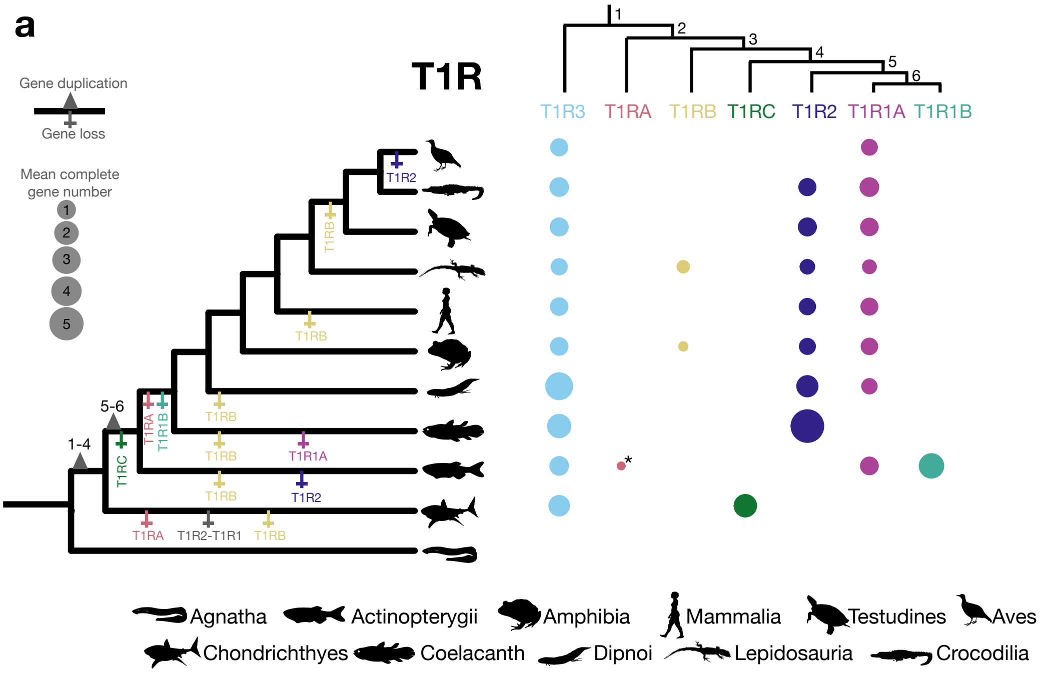

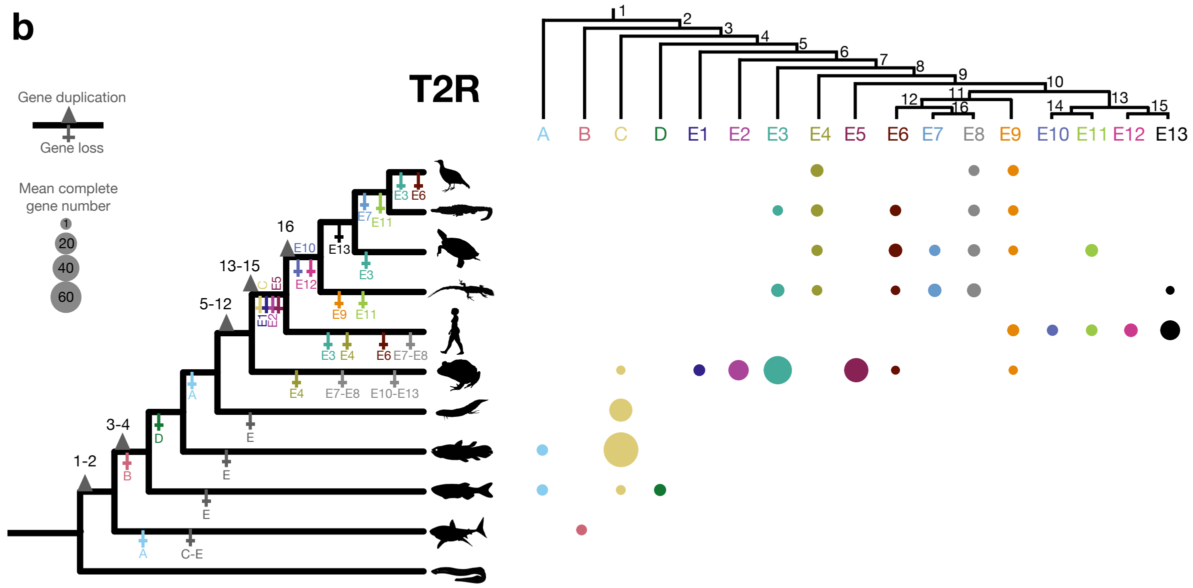

Supplementary Fig. 36 | Phylogenies showing the number of complete *T2R* genes. Vertebrate phylogeny and (sub)class phylogenies displaying the number of complete *T2R* genes (BUSCO80 dataset). **a**, Vertebrata; **b**, Agnatha; **c**, Chondrichthyes; **d**, Actinopterygii; **e**, Amphibia; **f**, Mammalia; **g**, Lepidosauria; **h**, Testudines; **i**, Aves and Crocodilia. Terminal branches and species names are colored according to (sub)class (**a**) or according to order based on the NCBI taxonomy database (**b-i**). *T2R*-subclades are indicated. Source data are provided as a Source Data file.

a) Vertebrates

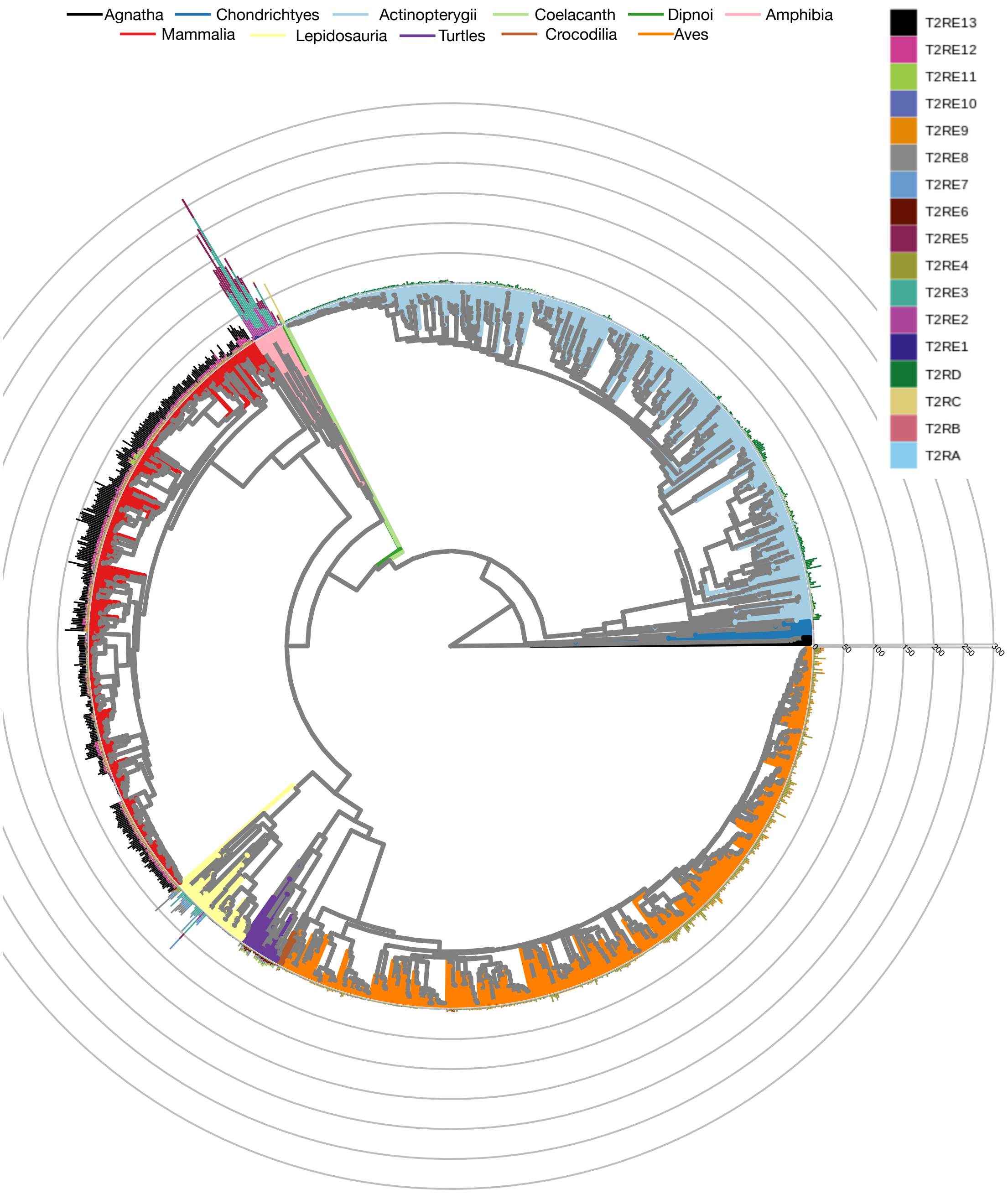

b) Agnatha

No T2R genes in agnathans

c) Chondrichthyes

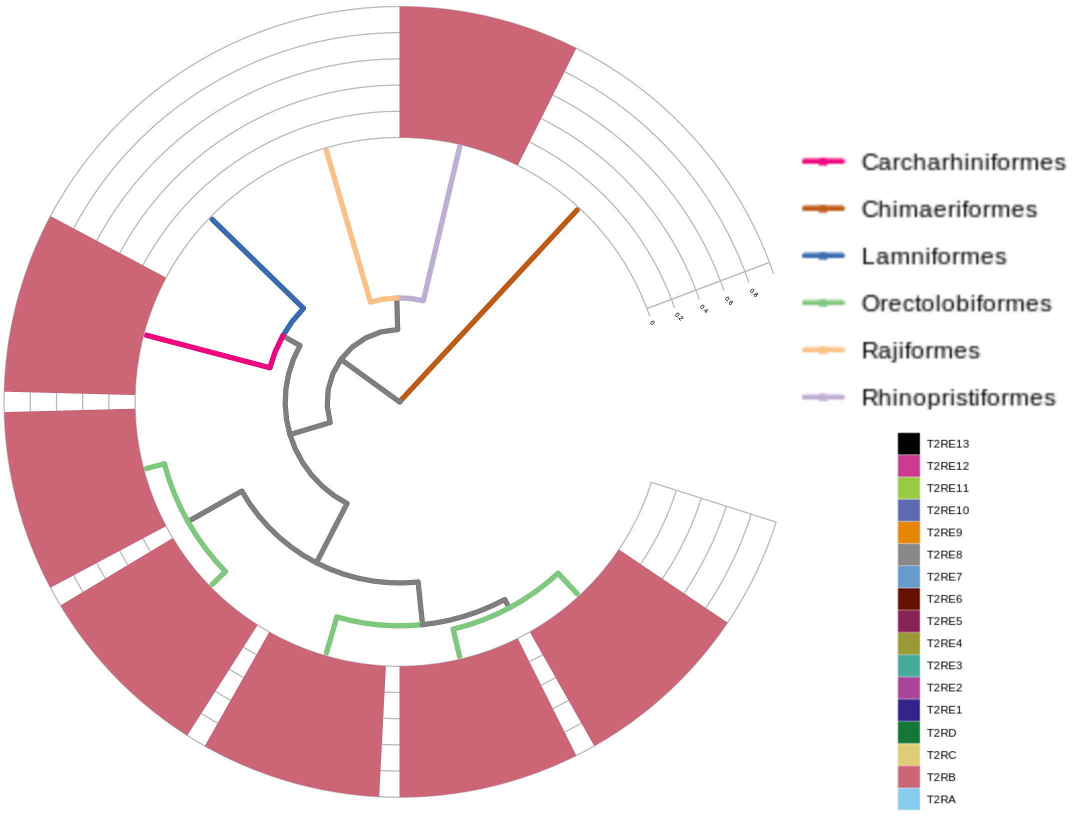

d) Actinopterygii

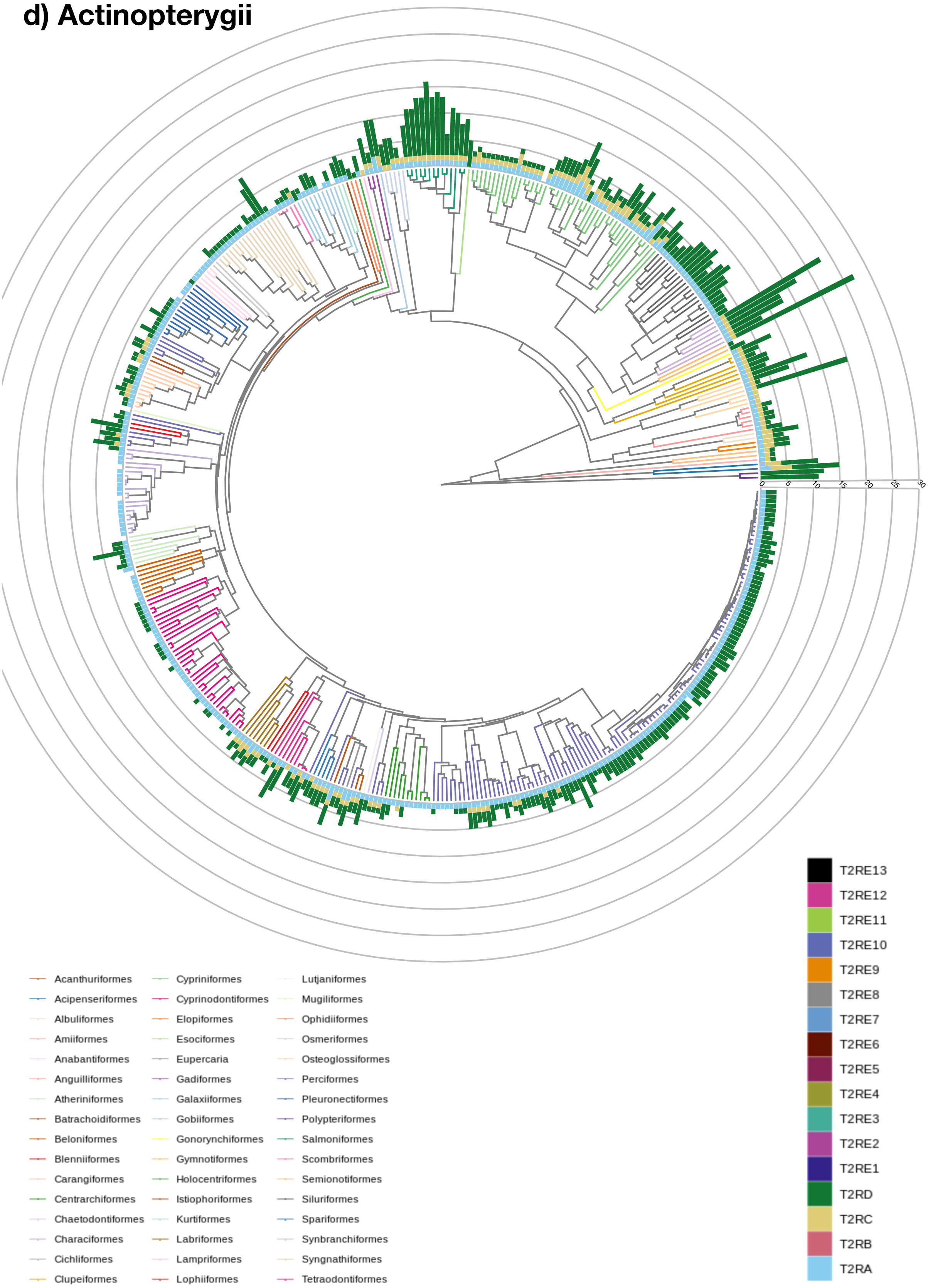

e) Amphibia

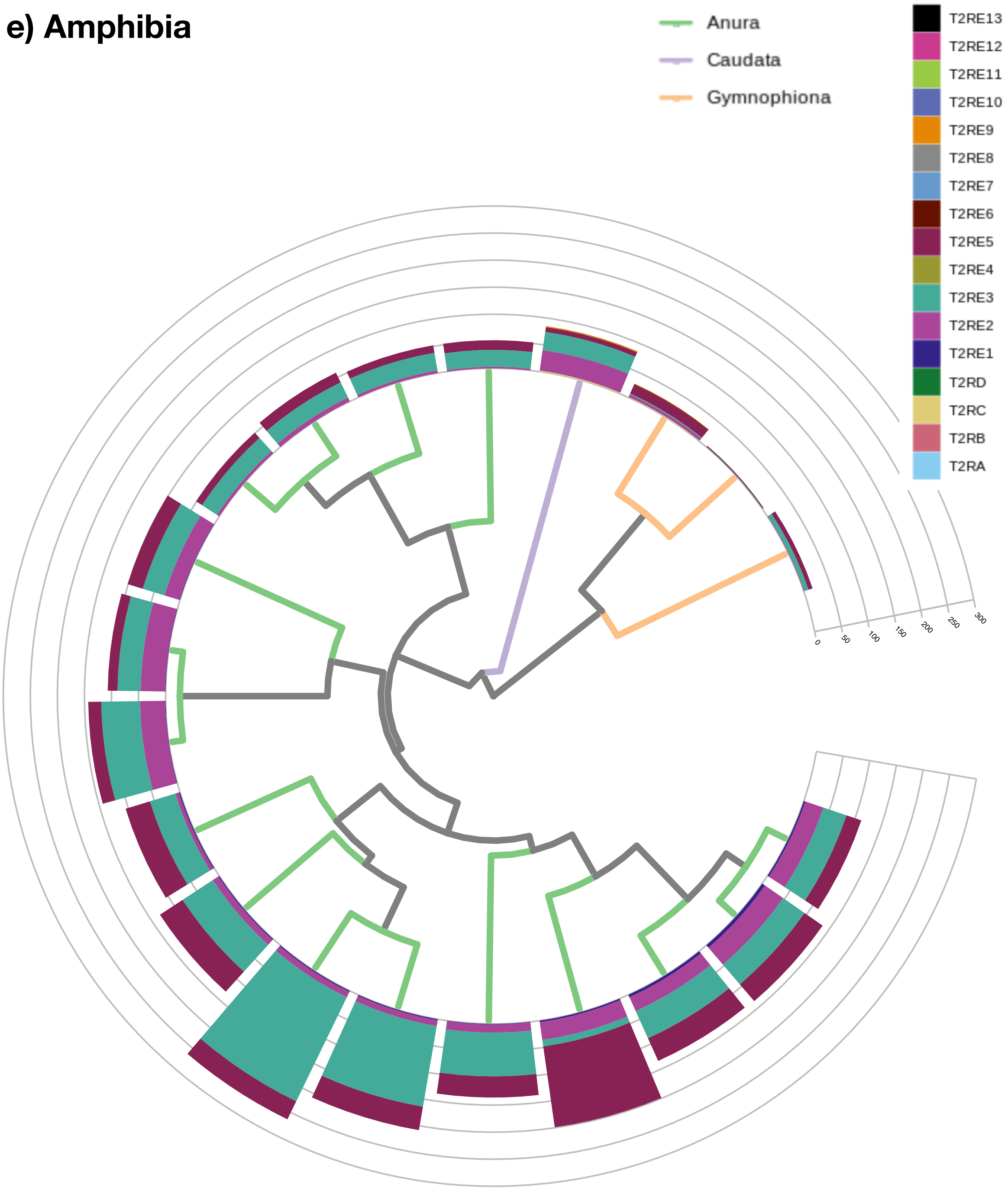
Anura
Caudata
Gymnophiona
T2RE13
T2RE12
T2RE11
T2RE10
T2RE9
T2RE8
T2RE7
T2RE6
T2RE5
T2RE4
T2RE3
T2RE2
T2RE1
T2RD
T2RC
T2RB
T2RA
0
50
100
150
200
250
300

f) Mammalia

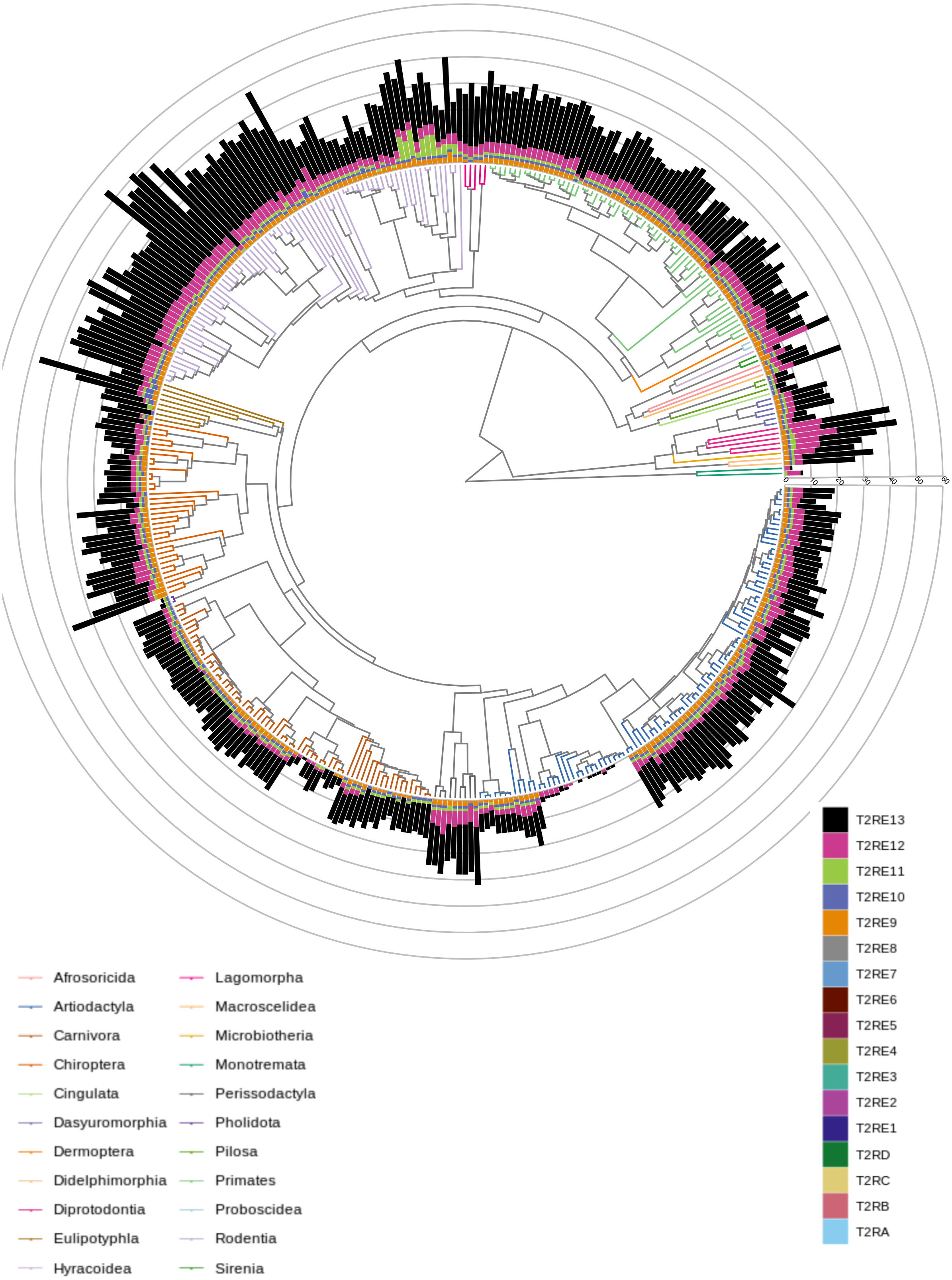

0
10
20
30
40
50
60
Afrosoricida
Artiodactyla
Carnivora
Chiroptera
Cingulata
Dasyuromorphia
Dermoptera
Didelphimorphia
Diprotodontia
Eulipotyphla
Hyracoidea
Lagomorpha
Macroscelidea
Microbiotheria
Monotremata
Perissodactyla
Pholidota
Pilosa
Primates
Proboscidea
Rodentia
Sirenia
T2RE13
T2RE12
T2RE11
T2RE10
T2RE9
T2RE8
T2RE7
T2RE6
T2RE5
T2RE4
T2RE3
T2RE2
T2RE1
T2RD
T2RC
T2RB
T2RA

g) Lepidosauria

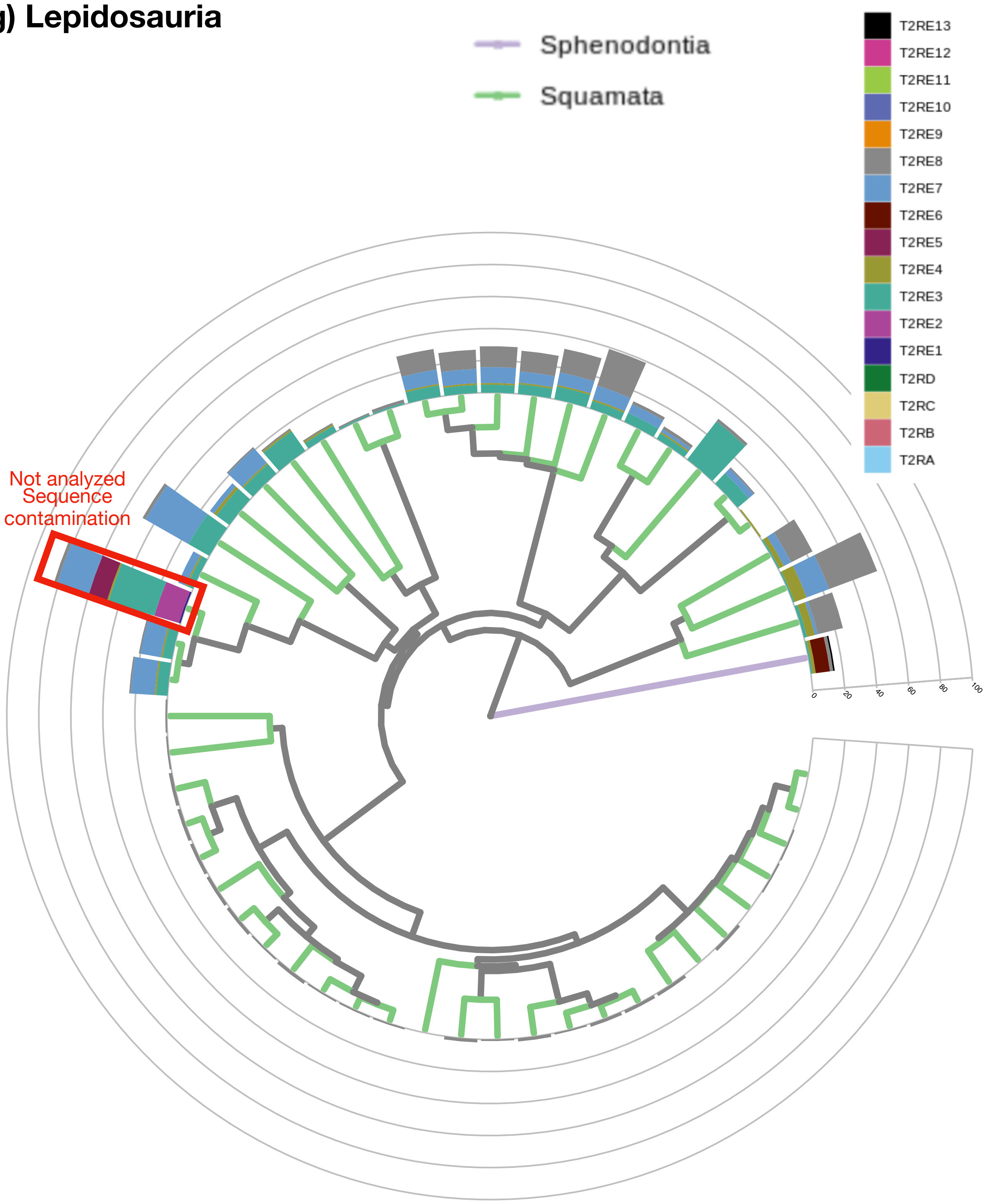

h) Turtles

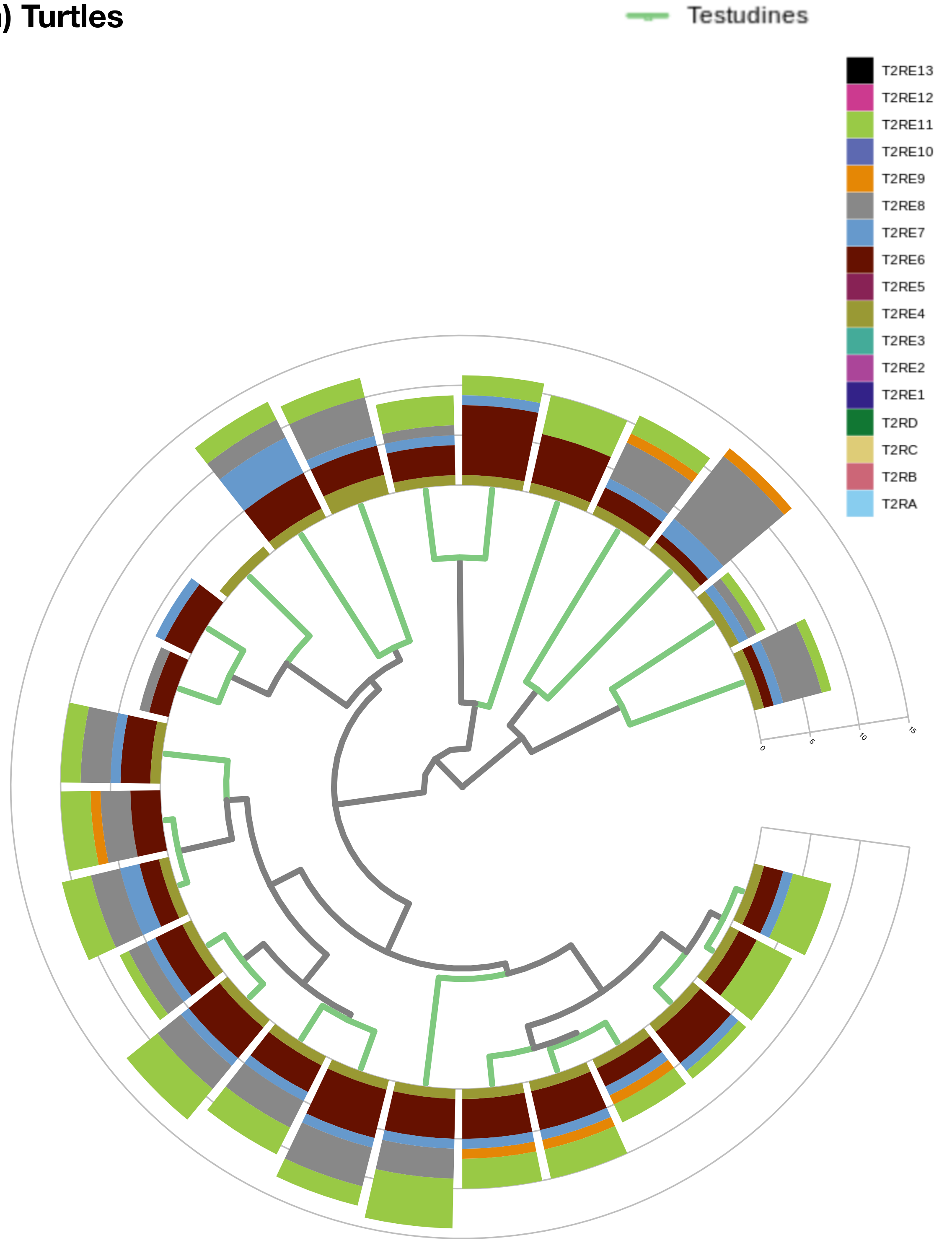
Testudines
T2RE13
T2RE12
T2RE11
T2RE10
T2RE9
T2RE8
T2RE7
T2RE6
T2RE5
T2RE4
T2RE3
T2RE2
T2RE1
T2RD
T2RC
T2RB
T2RA
0
5
10
15

i) Aves (with Crocodilia as outgroup)

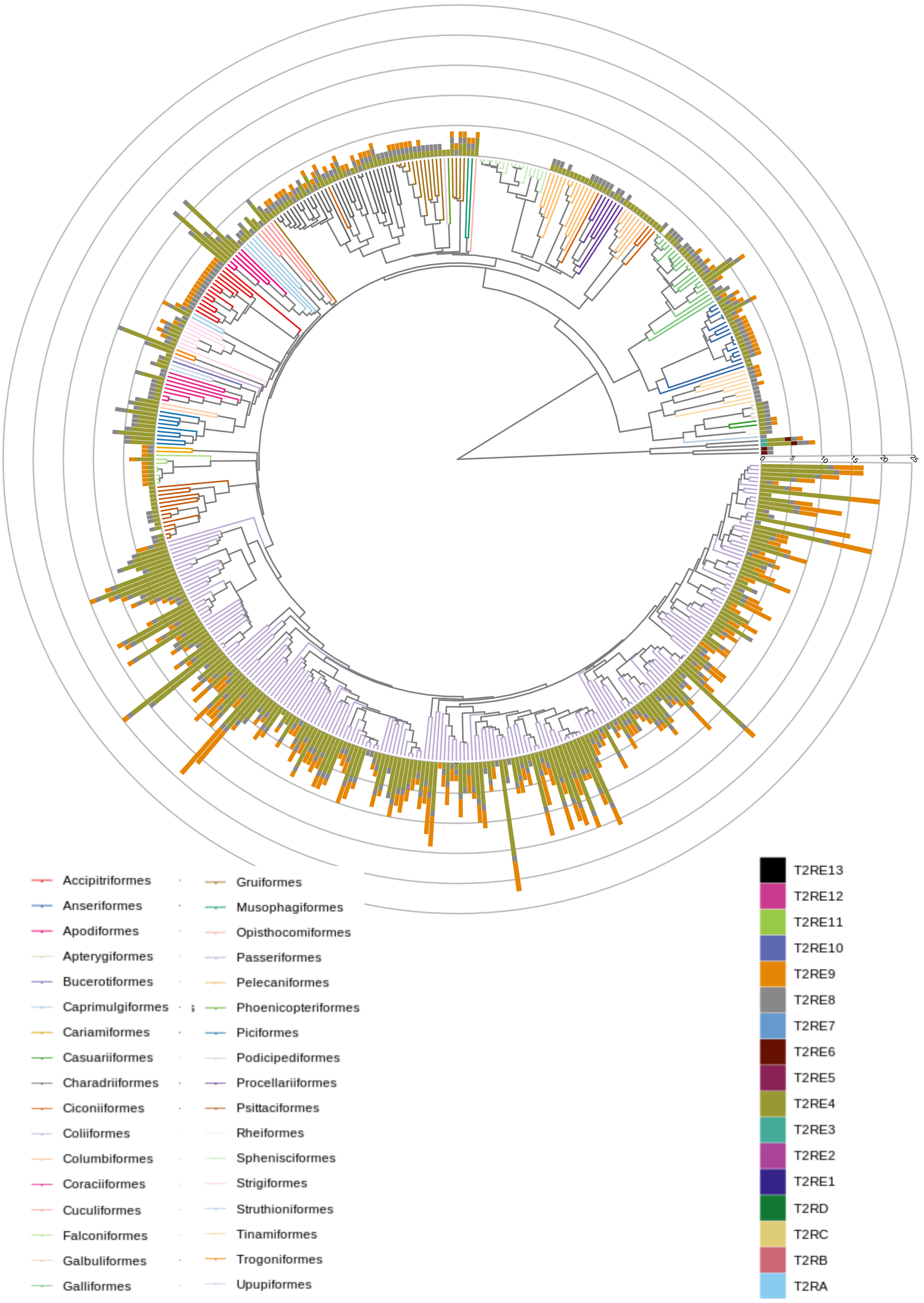

Supplementary Fig. 37 | *T1R* gene losses in mammals. Examples of phylogenies and related contingency tables that we made to infer if the loss of *T1R2* was more likely to happen in carnivore branches of the tree. "*T1R2* intact" refers to the number of branches with an intact *T1R2* gene, while "*T1R2* loss" refers to the number of independent *T1R2* losses. In the first example, two carnivore species have lost their *T1R2* in two independent loss events. In the second example, two carnivore species lack *T1R2*, but there is only one gene loss event.

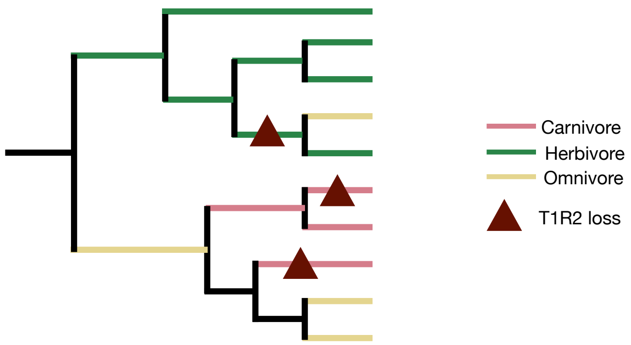

	T1R2 intact	T1R2 loss event
Carnivore	2	2
Herbivore	7	1
Omnivore	4	0

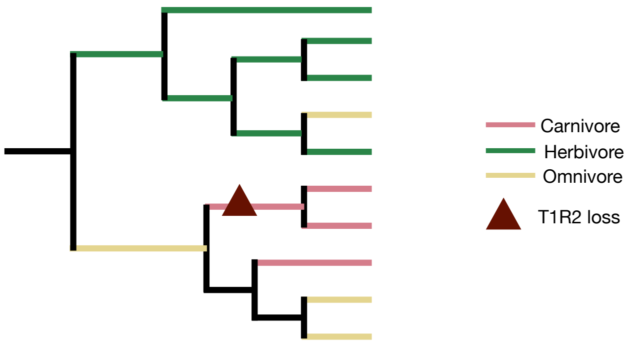

	T1R2 intact	T1R2 loss event
Carnivore	1	1
Herbivore	8	0
Omnivore	4	0

Supplementary Fig. 38 | Simulations of *T1R* gene losses in mammals. Simulation results where *T1R* genes were randomly pseudogenized in the mammal tree, once without taking branch lengths into account for *T1R1* (**a**), *T1R2* (**b**), and *T1R3* (**c**), and once with draw probabilities weighted by branch lengths for *T1R1* (**d**), *T1R2* (**e**), and *T1R3* (**f**). We also show the simulations of *T1R2* loss without cetaceans and pinnipeds, without taking branch length into account (**g**) and when weighting draw probabilities by branch length (**h**). Histograms represents the results of the simulations (with the x-axis representing the number of randomly drawn branches) and the dashed lines representing the observed number of independent *T1R* loss per diet group. The *P*-values reported above each dashed line correspond to the number of simulations where the same number, or more, independent *T1R1*, *T1R2* or *T1R3* losses occurred than the observed number on corresponding branches divided by the total number of simulations (10,000). T1R genes categories of mammals as well as their diet preferences can be found in Supplementary Data 1. Source data are provided as a Source Data file.

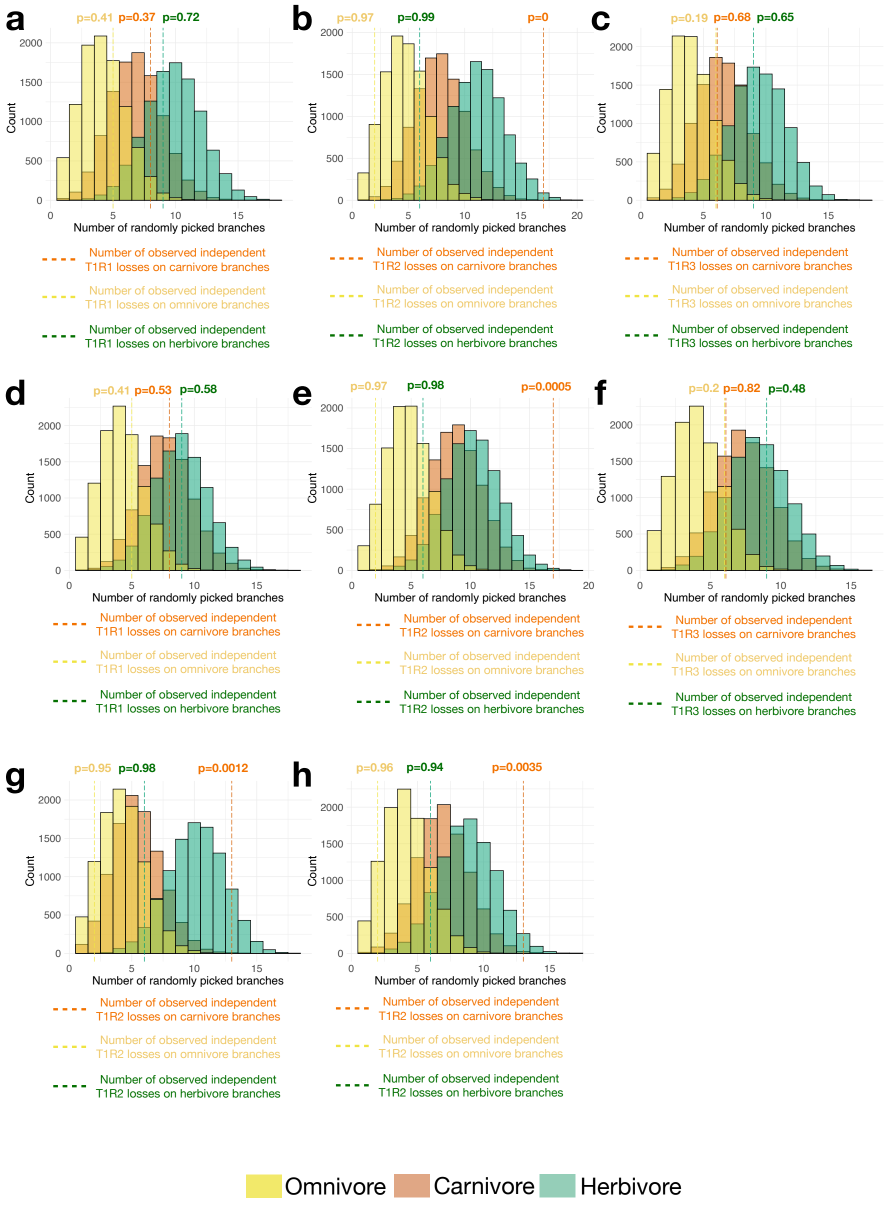

Supplementary Fig. 39 | Natural selection analysis on sirenian *T1R1* genes. PAML was used to compute the dN/dS (= ω) per branch under four different models. **a**, Model 1: Free-ratio model which fits one ω ratio per branch. The mean ω ratio of cetacean, pinniped and sirenian branches is indicated. **b**, Model 2: Two-ratio model with one ω ratio for marine mammals (cetaceans, pinnipeds and sirenians) and one ω ratio for closely related terrestrial mammals. **c**, Model 3: Two-ratio model with one ω ratio for cetaceans and pinnipeds and one ω for sirenians and terrestrial mammals. **d**, Model 4: Three-ratio model with one ω ratio for cetaceans and pinnipeds, one ω ratio for sirenians and one ω ratio for terrestrial mammals. For the free-ratio model, the mean ω ratio of cetaceans, pinnipeds and sirenians is indicated (0.61, 0.64 and 0.36, respectively). **e**, Log-likelihood (Lnl) and number of parameters (np) of each model. The log-likelihood, as well as the ω value found under a null model (one-ratio, assuming the same ω for all the branches) is also indicated. **f**, Log-likelihood ratio test between model 4 and model 2 as well as between model 4 and model 3. The free-ratio model was the best model, with ω values being lower in sirenian branches than in cetaceans and pinnipeds. The three-ratio model was significantly better than the two two-ratio models, indicating a lower negative selection on sirenian *T1R1* genes (ω = 0.37) compared to terrestrial mammals (ω = 0.21), but higher than in other aquatic mammals (ω = 0.57). **g**, RELAX results assigning terrestrial mammals as reference branches and either pinnipeds, cetaceans or sirenians as test branches. While RELAX found evidences of relaxed selection in pinnipeds and cetaceans ($K<1$, *P*-value < 0.5), no evidence of relaxed or positive selection was retrieved in sirenians. Source data are provided as a Source Data file.

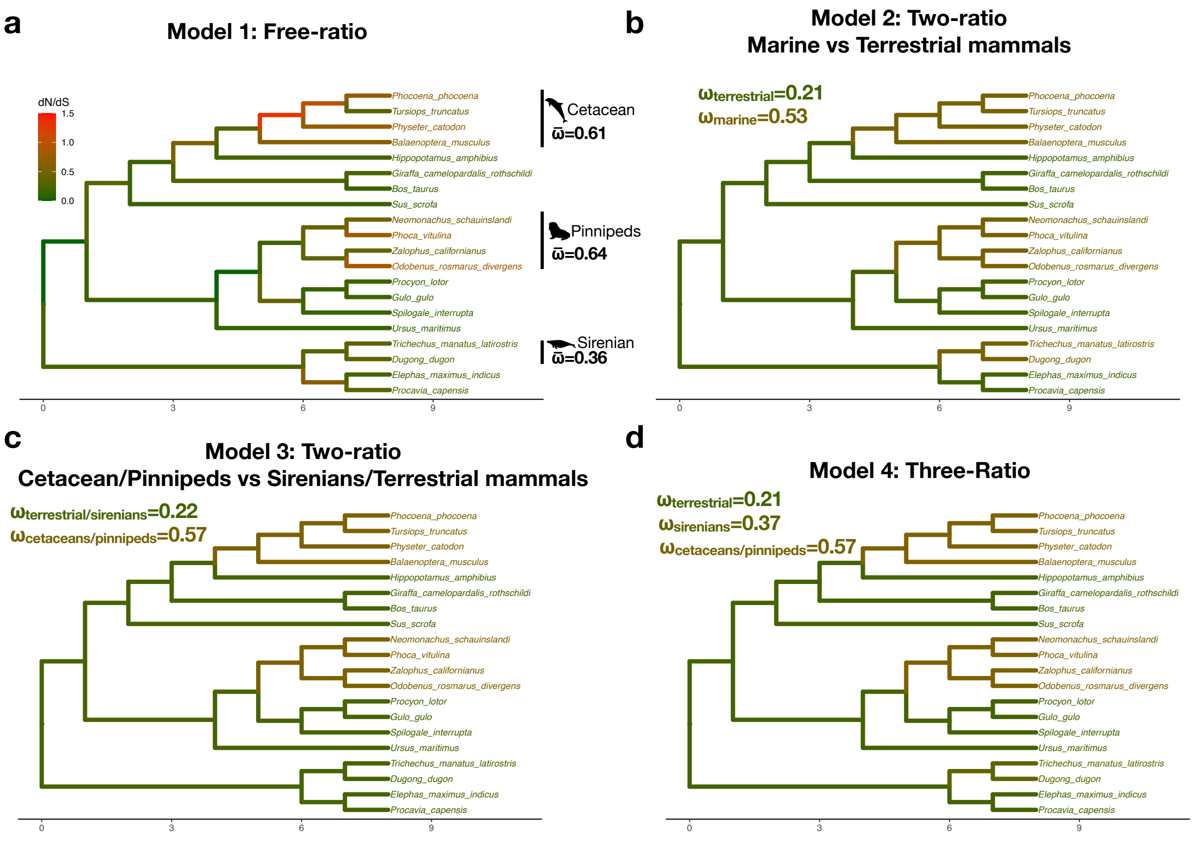

e

Model	np	Lnl
Model 1	77	-16291.3943
Model 4	42	-16319.33553
Model 2	41	-16321.6407
Model 3	41	-16324.17046
Null model (ω=0.27)	40	-16369.12442

f

Models compared	LRT	df	p-value
Model 4 vs Model 2	4.61	1	0.032
Model 4 vs Model 3	9.67	1	0.002

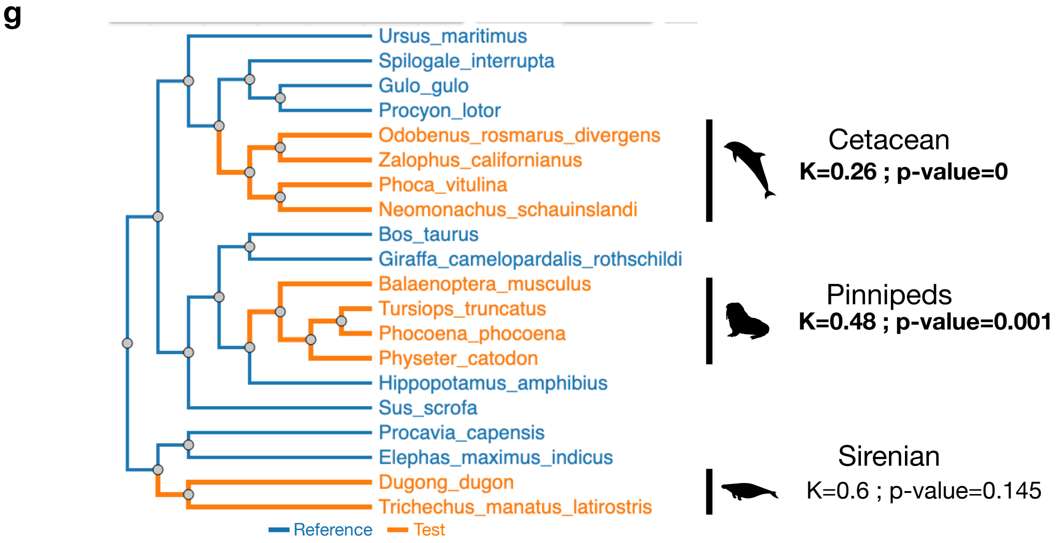

Supplementary Fig. 40 | Natural selection analysis on sirenian T1R2 genes. PAML was used to compute the dN/dS (= ω) per branch under four different models. **a**, Model 1: Free-ratio model which fits one ω ratio per branch. The mean ω ratio of cetacean, pinniped and sirenian branches is indicated. **b**, Model 2: Two-ratio model with one ω ratio for marine mammals (cetaceans, pinnipeds and sirenians) and one ω ratio for closely related terrestrial mammals. **c**, Model 3: Two-ratio model with one ω ratio for cetaceans and pinnipeds and one ω for sirenians and terrestrial mammals. **d**, Model 4: Three-ratio model with one ω ratio for cetaceans and pinnipeds, one ω ratio for sirenians and one ω ratio for terrestrial mammals. For the free-ratio model, the mean ω ratio of cetaceans, pinnipeds and sirenians is indicated (0.35, 0.41 and 0.18, respectively). **e**, Log-likelihood (Lnl) and number of parameters (np) of each model. The log-likelihood, as well as the ω value found under a null model (one-ratio, assuming the same ω for all the branches) is also indicated. **f**, Log-likelihood ratio test between model 4 and model 2 as well as between model 4 and model 3. The free-ratio model was the best model, with ω values being lower in sirenian branches than in cetaceans and pinnipeds. The three-ratio model was significantly better than model 2 but not significantly better than model 3. This indicates that the ω ratio of sirenians (ω=0.16) is not significantly higher than the ω ratio of terrestrial mammals (ω=0.13), but significantly lower than in other aquatic mammals (ω=0.33). **g**, RELAX results assigning terrestrial mammals as reference branches and either pinnipeds, cetaceans or sirenians as test branches. While RELAX found evidences of relaxed selection in pinnipeds and cetaceans ($K<1$, P-value < 0.5), no evidence of relaxed or positive selection was retrieved in sirenians. Source data are provided as a Source Data file.

a

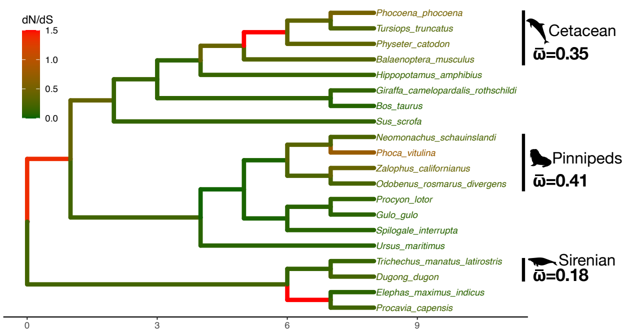

b

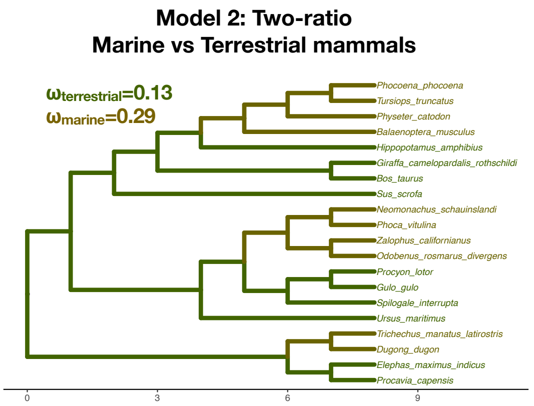

c

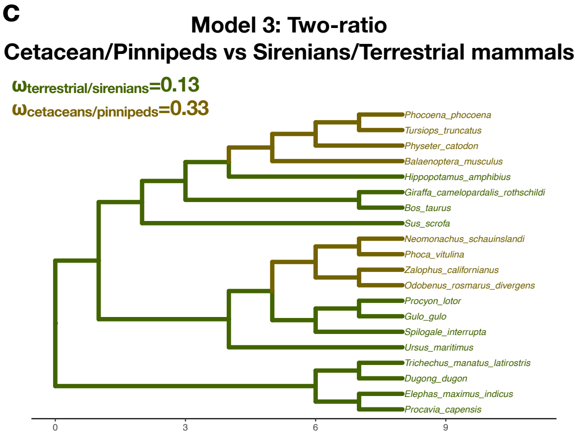

d

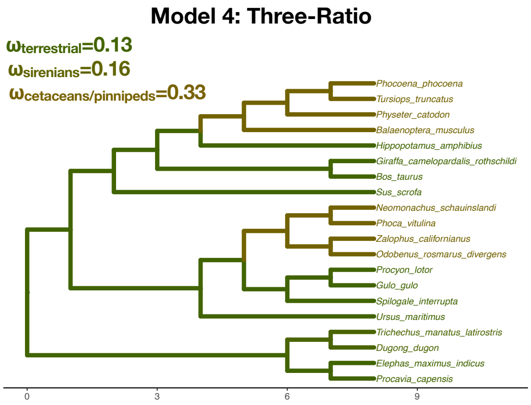

e

Model	np	Lnl
Model 1	77	-16134.61384
Model 4	42	-16187.15928
Model 3	41	-16187.67518
Model 2	41	-16193.66457
Null model (ω=0.17)	40	-16232.39180

f

Models compared	LRT	df	p-value
Model 4 vs Model 2	13.01	1	0.0003
Model 4 vs Model 3	1.03	1	0.31

g

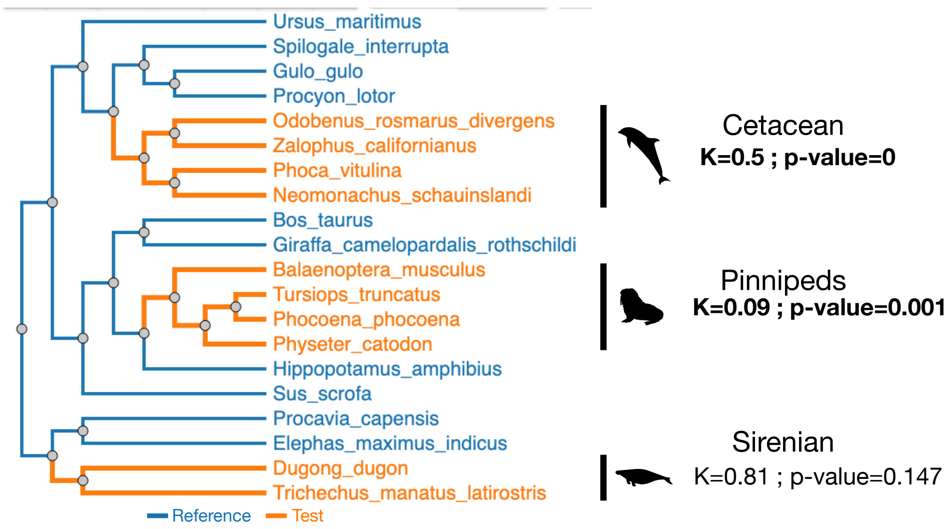

Supplementary Fig. 41 | Natural selection analysis on sirenian *T1R3* genes. PAML was used to compute the dN/dS (= ω) per branch under four different models. **a**, Model 1: Free-ratio model which fits one ω ratio per branch. The mean ω ratio of cetacean, pinniped and sirenian branches is indicated. **b**, Model 2: Two-ratio model with one ω ratio for marine mammals (cetaceans, pinnipeds and sirenians) and one ω ratio for closely related terrestrial mammals. **c**, Model 3: Two-ratio model with one ω ratio for cetaceans and pinnipeds and one ω for sirenians and terrestrial mammals. **d**, Model 4: Three-ratio model with one ω ratio for cetaceans and pinnipeds, one ω ratio for sirenians and one ω ratio for terrestrial mammals. For the free-ratio model, the mean ω ratio of cetaceans, pinnipeds and sirenians is indicated (0.47, 0.42 and 0.22, respectively). **e**, Log-likelihood (Lnl) and number of parameters (np) of each model. The log-likelihood, as well as the ω value found under a null model (one-ratio, assuming the same ω for all the branches) is also indicated. **f**, Log-likelihood ratio test between model 4 and model 2 as well as between model 4 and model 3. The free-ratio model was the best model, with ω values being lower in sirenian branches than in cetaceans and pinnipeds. The three-ratio model was significantly better than model 3 but not significantly better than model 2. This indicates that the ω ratio of sirenians (ω=0.31) is not significantly lower than the ω ratio of aquatic mammals (ω=0.38), but significantly higher than in terrestrial mammals (ω=0.15). **g**, RELAX results assigning terrestrial mammals as reference branches and either pinnipeds, cetaceans or sirenians as test branches. While RELAX found evidences of relaxed selection in pinnipeds and cetaceans (K<1, *P*-value < 0.5), no evidence of relaxed or positive selection was retrieved in sirenians. Source data are provided as a Source Data file.

a　**b**　**c**　**d**

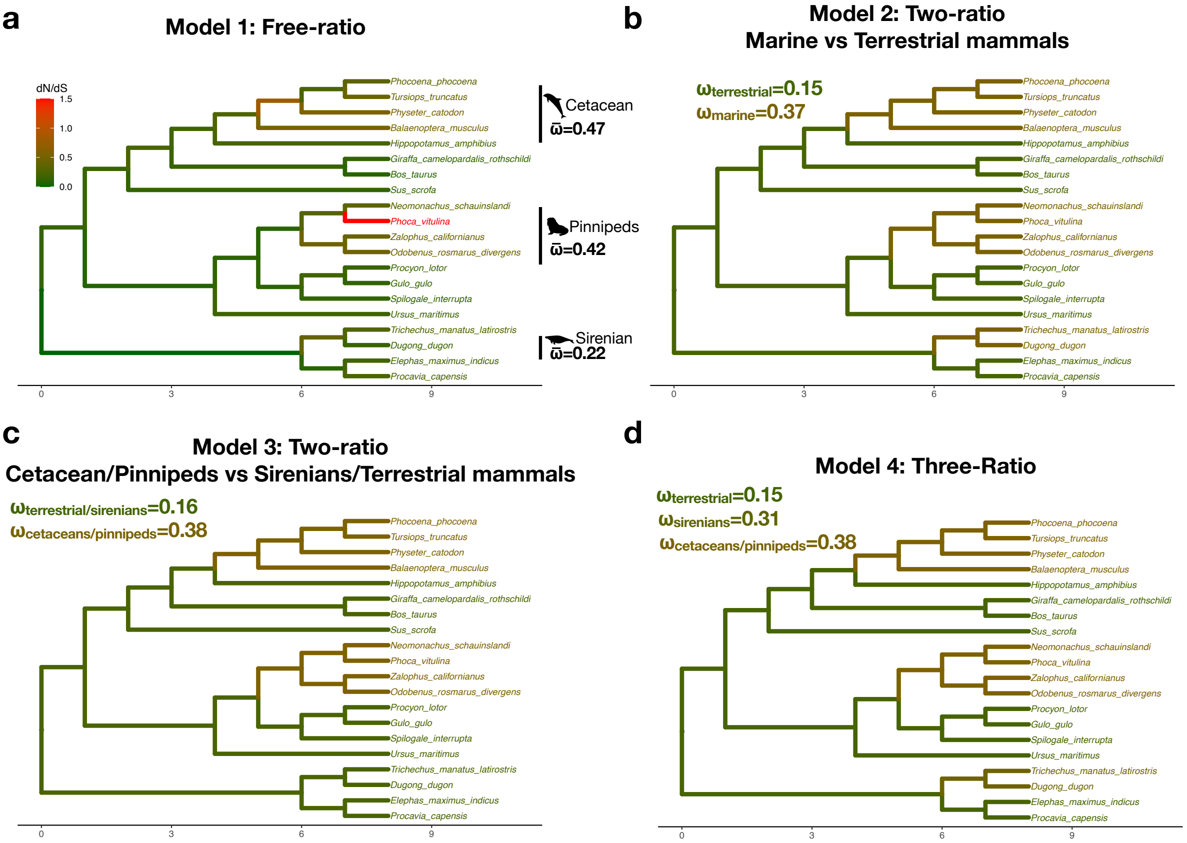

e

Model	np	Lnl
Model 1	77	-20196.35761
Model 4	42	-20234.44752
Model 2	41	-20235.11004
Model 3	41	-20242.62811
Null model (ω=0.21)	40	-20293.35398

f

Models compared	LRT	df	p-value
Model 4 vs Model 2	1.325	1	0.25
Model 4 vs Model 3	16.36	1	5.2e-5

g

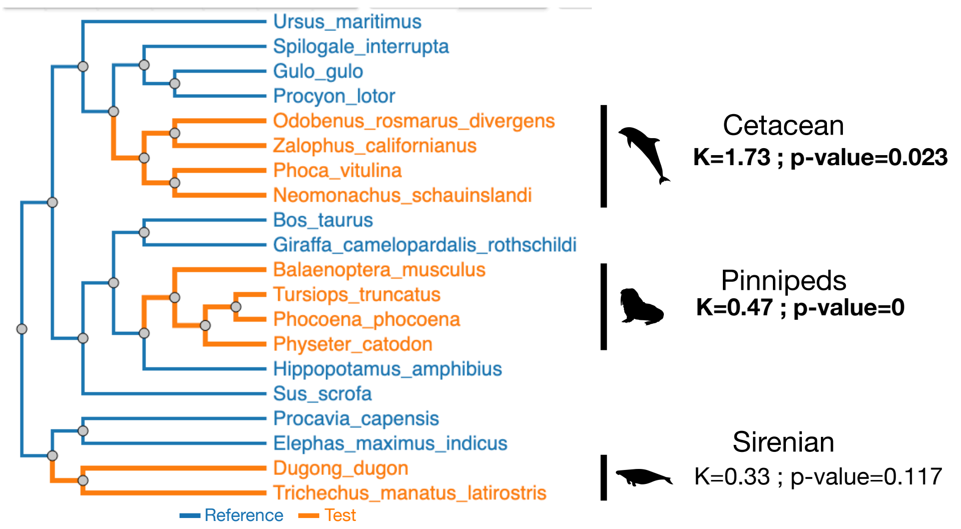

Supplementary Fig. 42 | Vertebrate *T2R* gene tree. a, Near maximum likelihood phylogeny of *T2R* genes of vertebrates with branches colored according to the vertebrate (sub)class. **b**, Clade tree generated from (**a**) with local support values indicated at each node (according to a Shimodaira-Hasegawa test). For *T2R* genes, we completely redefined the nomenclature, as no proper gene topology with representative vertebrate species are available as of yet. The tree in newick version, as well as all sequences and the alignment file can be found in FigShare. Sub-trees of each vertebrate (sub)class with the number of *T2R* genes are shown in Supplementary Fig. 36. Maximum likelihood phylogeny of *T2R* genes extracted from one species per vertebrate (sub)class is shown in Supplementary Figs. 43-44.

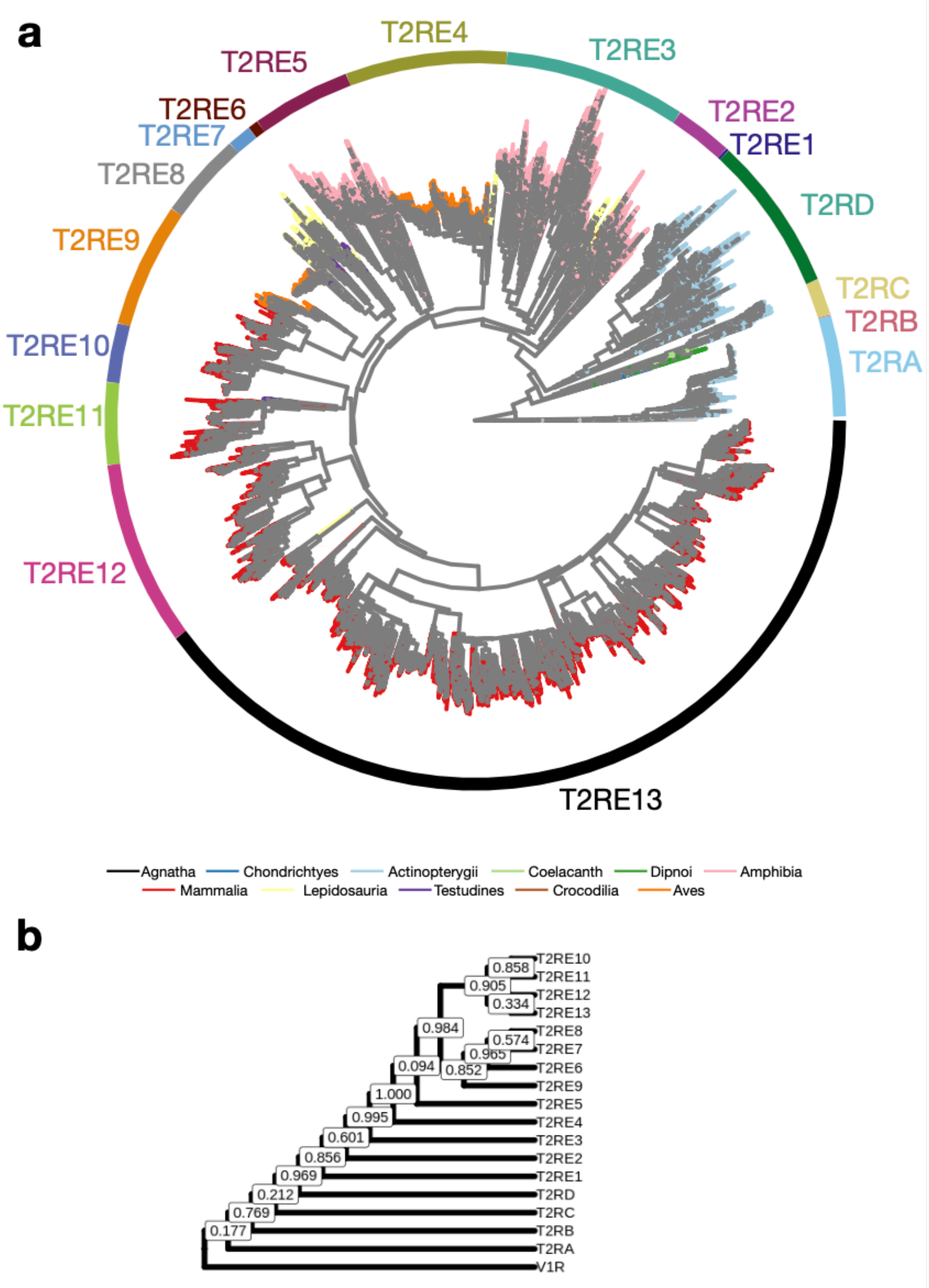

Supplementary Fig. 43 | Maximum likelihood phylogeny of *T2R* genes. Maximum likelihood phylogeny of *T2R* genes extracted from one representative species per vertebrate subclass. Branches are colored according to the vertebrate (sub)class. *T2R* subclades are indicated (*T2RB* is directly indicated at the tip of the corresponding branch as there was only one sequence in this clade). The tree was computed using the optimal model found by ModelFinder (JTT+F+R7). The robustness of the nodes was evaluated with 1,000 ultrafast bootstraps and these bootstrap values are reported for each node on Supplementary Fig. 44. All the clades retrieved using the near-ML method were retrieved as monophyletic, and the exact same topology was retrieved.

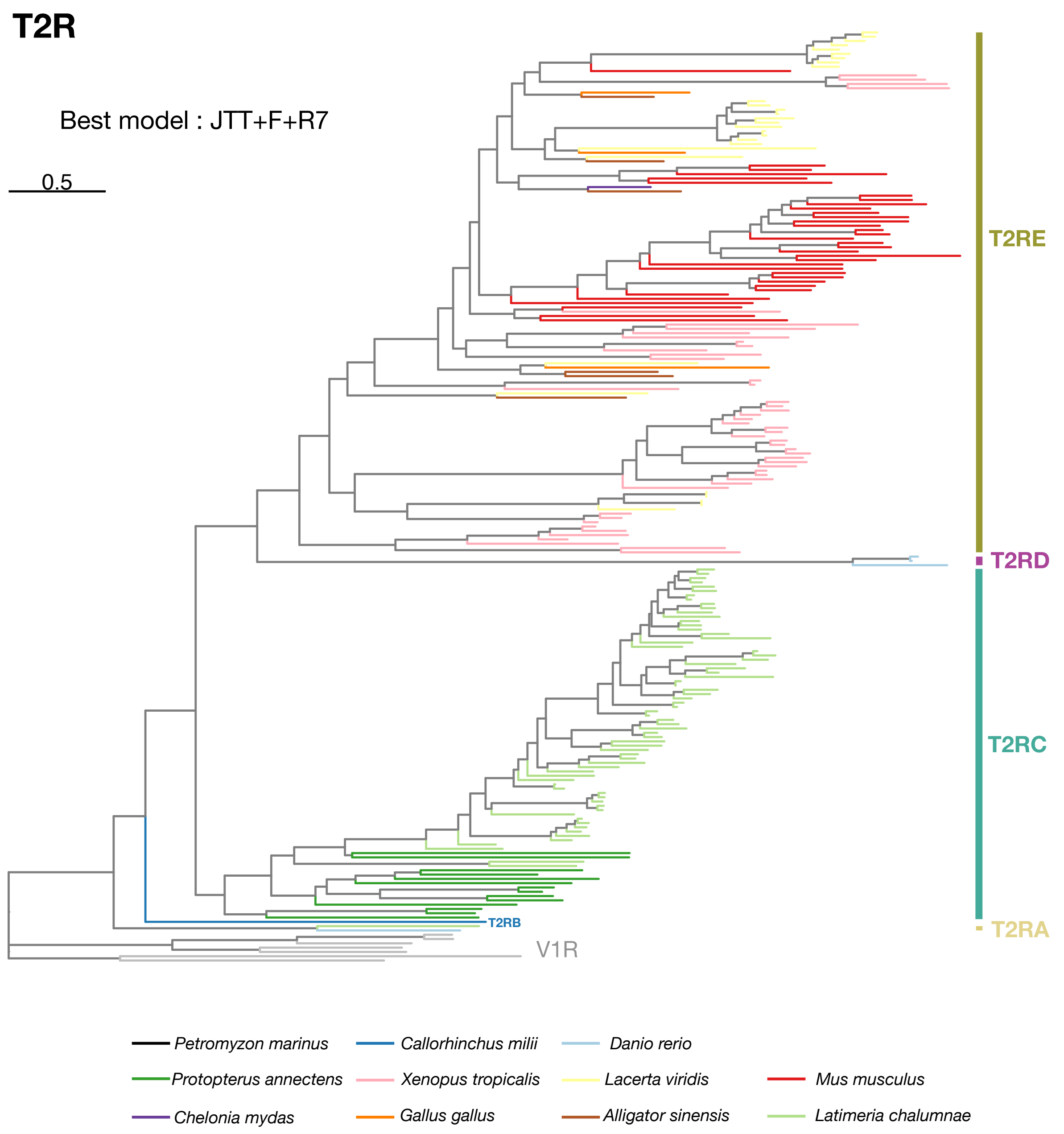

Supplementary Fig. 44 | Maximum likelihood phylogeny of *T2R* genes. Same maximum likelihood phylogeny of *T2R* genes as in Supplementary Fig. 43 but with bootstrap values indicated at each node.

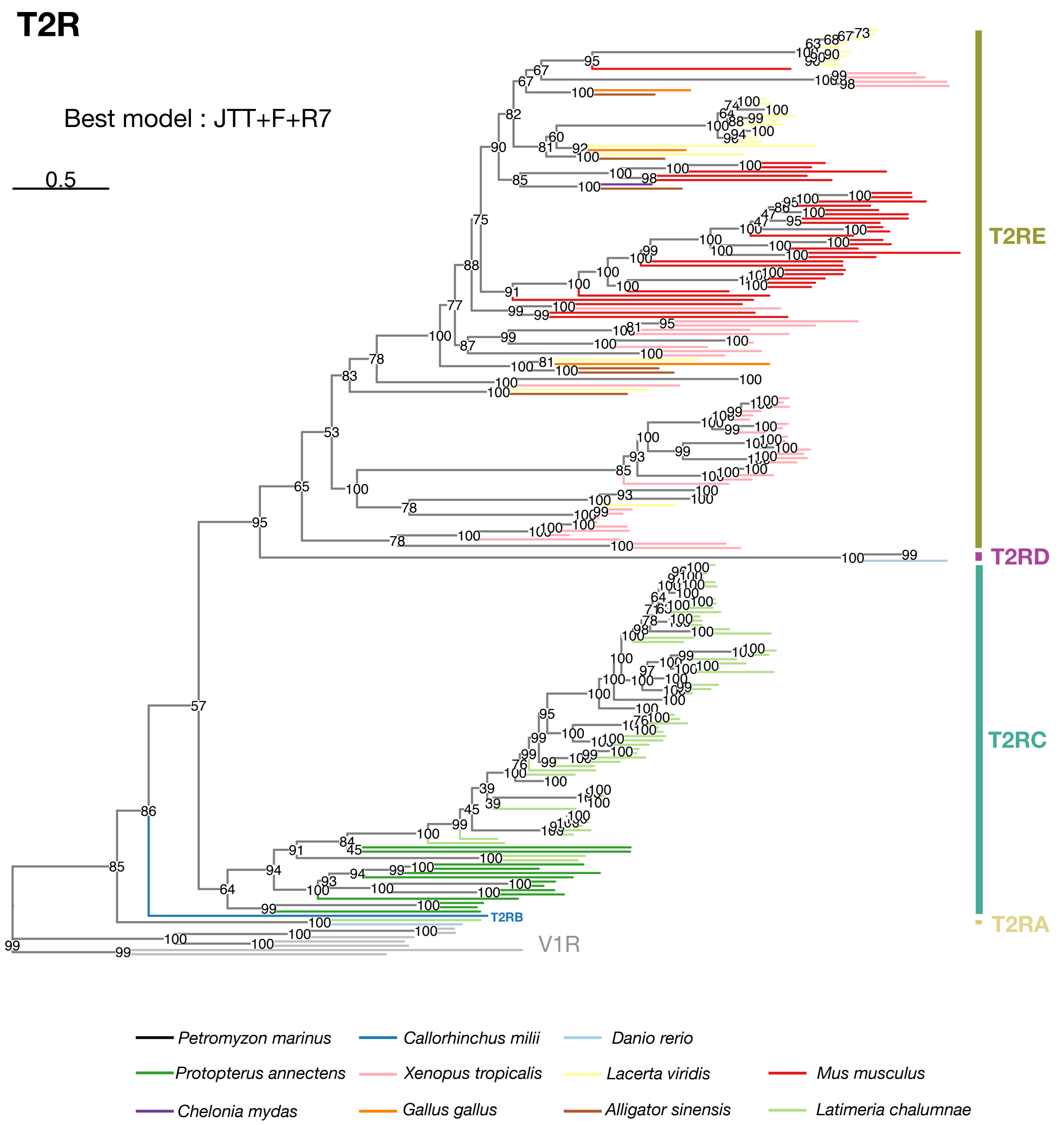

Supplementary Fig. 45 | Association between the number of *T2R* genes and diet preferences in vertebrates. Diet preferences were retrieved for every species from various databases (see Methods). To examine if the *T2R* gene repertoire was shaped by diet preferences in mammals, birds and ray-finned fishes, we performed two-sided pGLS analyses between the number of complete *T2R* genes and the diet. pGLS *P*-values are indicated above each boxplot (first quartile - 1.5; the interquartile range, the first quartile, the mean, the third quartile and the third quartile + 1.5 interquartile range. Dots represent outliers) and each test were performed twice, taking BUSCO80 (left) or BUSCO90 (right) species. For mammals, we further tested the impact of the two carnivore marine clades on the pGLS results. Note that when considering only chromosome-scale assemblies, the pGLS stayed significant in mammals (pGLS *P*-value=0.024) and non-significant in ray-finned fishes and birds (*P*-value=0.37 and *P*-value=0.26 respectively). Source data are provided as a Source Data file.

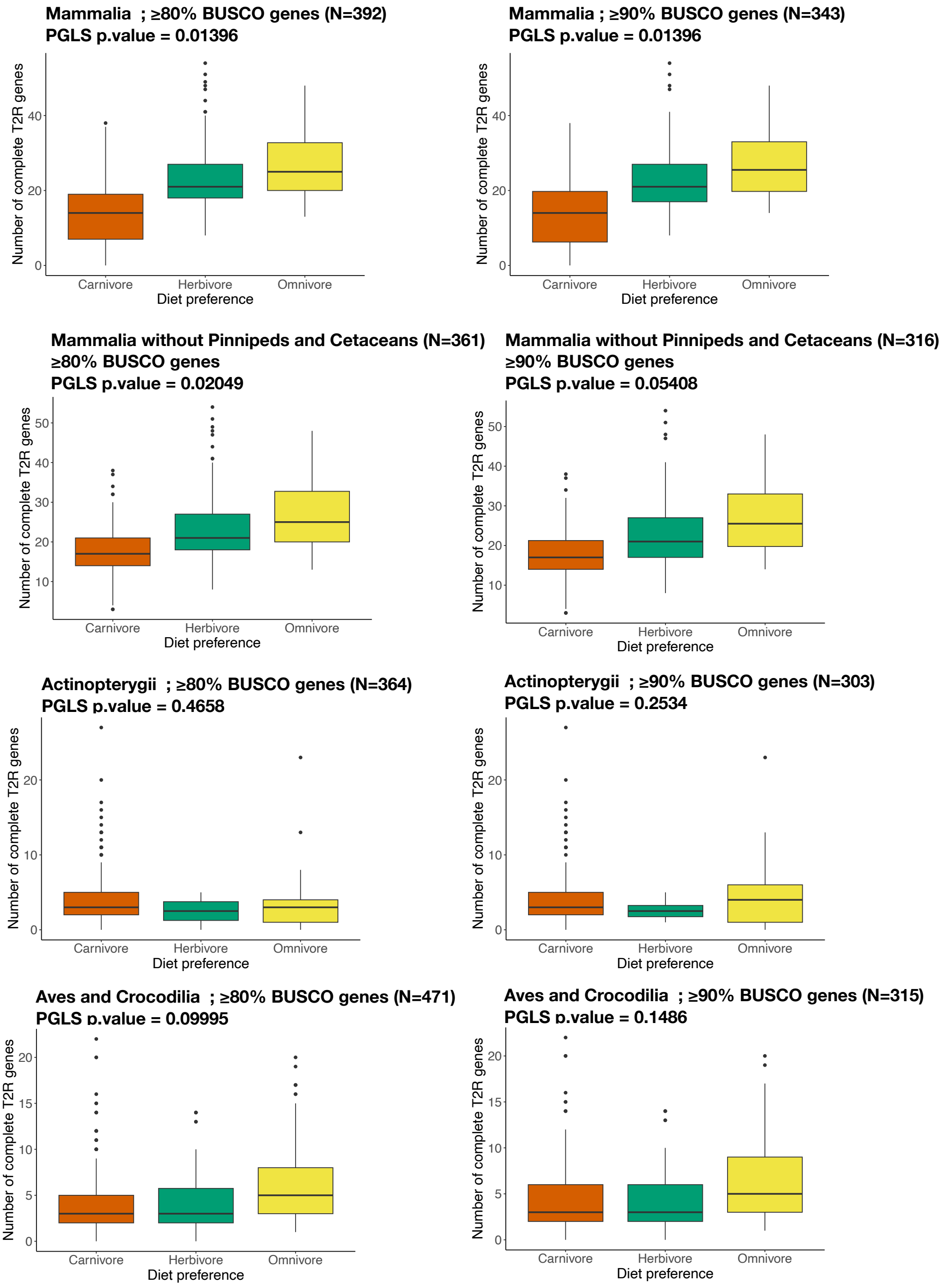

Supplementary Fig. 46 | Significant results from pGLS analysis comparing complete chemoreceptor gene numbers and ecological parameters. a, mammals; **b**, birds; **c**, ray-finned fishes. Due to the stringency of pGLS analysis combined with the high number of tests (increasing the chances to find false positives), we only considered as significant pGLS results with a $P_{\mathrm{PGLS}} < 0.01$, and only pGLS results meeting this criterion are shown for mammals, ray-finned fishes and birds. Results were consistent between the BUSCO80 and BUSCO90 datasets, except for the pGLS between the number of complete *OR* genes and the primary lifestyle of birds, which was not significant with the BUSCO90 dataset (p-value = 0.07) but significant with the BUSCO80 dataset (*P*-value = 2e-3). The pGLS between the number of complete *T1R* genes and the primary aquatic habitat of ray-finned fishes was still significant after removing the only species (*Albula glossodonta*) in the "brack" category (BUSCO80 P_{PGLS} = 2.149e-3; BUSCO90 P_{PGLS} = 4.252e-3). The figure also show that results are consistent when taking only chromosome-scale assemblies, except between the number of *OR* genes and the habitat of birds (P_{PGLS} = 0.15, not shown) and between the number of *OR* genes and the migratory behavior of birds (P_{PGLS} = 0.12, not shown). Aquatic habitat in ray-finned fishes is coded as B…brackisch, F…freshwater, M…marine (allowing for combinations); migratory behavior in birds is coded as S…sedentary, P…partically migratory, M…migratory; habitat in mammals is encoded according to their ForStrat.Value as M…marine, G…ground level including aquatic foraging, S…scansorial, Ar…arboreal, A…aerial (see Supplementary Data 1). Box-plos represent the first quartile - 1.5; the interquartile range, the first quartile, the mean, the third quartile and the third quartile + 1.5 interquartile range. Dots represent outliers. Source data are provided as a Source Data file.

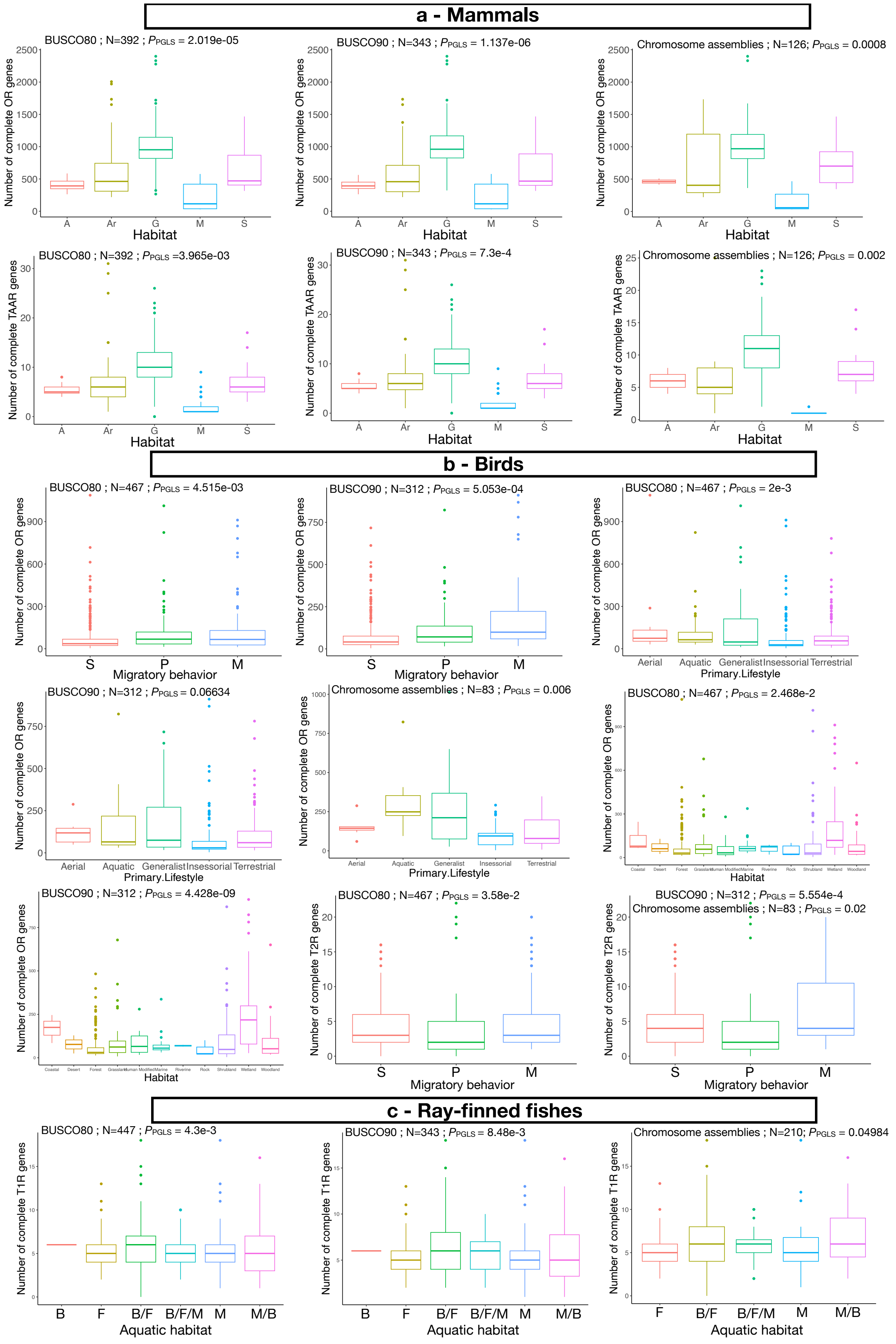
a - Mammals
BUSCO80 ; N=392 ; PPGLS = 2.019e-05
BUSCO90 ; N=343 ; PPGLS = 1.137e-06
Chromosome assemblies ; N=126; PPGLS = 0.0008
Number of complete OR genes
Habitat
A Ar G M S
BUSCO80 ; N=392 ; PPGLS =3.965e-03
BUSCO90 ; N=343 ; PPGLS = 7.3e-4
Chromosome assemblies ; N=126; PPGLS = 0.002
Number of complete TAAR genes
b - Birds
BUSCO80 ; N=467 ; PPGLS = 4.515e-03
BUSCO90 ; N=312 ; PPGLS = 5.053e-04
BUSCO80 ; N=467 ; PPGLS = 2e-3
Migratory behavior
S P M
Primary.Lifestyle
Aerial Aquatic Generalist Insessorial Terrestrial
BUSCO90 ; N=312 ; PPGLS = 0.06634
Chromosome assemblies ; N=83 ; PPGLS = 0.006
BUSCO80 ; N=467 ; PPGLS = 2.468e-2
BUSCO90 ; N=312 ; PPGLS = 4.428e-09
Coastal Desert Forest Grassland Human Modified Marine Riverine Rock Shrubland Wetland Woodland
BUSCO80 ; N=467 ; PPGLS = 3.58e-2
BUSCO90 ; N=312 ; PPGLS = 5.554e-4
Chromosome assemblies ; N=83 ; PPGLS = 0.02
Number of complete T2R genes
c - Ray-finned fishes
BUSCO80 ; N=447 ; PPGLS = 4.3e-3
BUSCO90 ; N=343 ; PPGLS = 8.48e-3
Chromosome assemblies ; N=210; PPGLS = 0.04984
Number of complete T1R genes
Aquatic habitat
B F B/F B/F/M M M/B

Supplementary Fig. 47 | Selection test for *OR* genes in five cetacean species. Unrooted species tree used for dN/dS (= ω ratio) analysis as well as the different models tested. Model 5 allowing a different ω ratio per branch is not shown as it was never significantly better than the other models. The brown branches correspond to terrestrial species outgroups (*Sus scrofa* and *Hippopotamus amphibius*) and the blue branch corresponds to the cetacean species tested: *Tursiops truncatus* (**a**), *Orcinus orca* (**d**), *Monodon monoceros* (**g**), *Physeter catodon* (**j**), *Megaptera novaeangliae* (**m**). The number and proportion of *OR* genes supported by each model is shown as pie chart for *Tursiops truncatus* (**b**), *Orcinus orca* (**e**), *Monodon monoceros* (**h**), *Physeter catodon* (**k**), *Megaptera novaeangliae* (**n**). For each *OR* gene best supported by model 2, a bar plot shows the ω ratio of the terrestrial branches (brown, $\omega_1 = \omega_2$) and the ω ratio of the cetacean species (blue, ω_3): Tursiops *truncatus* (**c**), *Orcinus orca* (**f**), *Monodon monoceros* (**i**), *Physeter catodon* (**l**), *Megaptera novaeangliae* (**o**). The number of *OR* genes best supported by model 2 and showing accelerated ($(\omega_1 = \omega_2) < \omega_3$) or decelerated evolution ($(\omega_1 = \omega_2) > \omega_3$) is reported at the top of the bar plots, their percentage relative to the total number of *OR* genes is provided in parentheses. Source data are provided as a Source Data file.

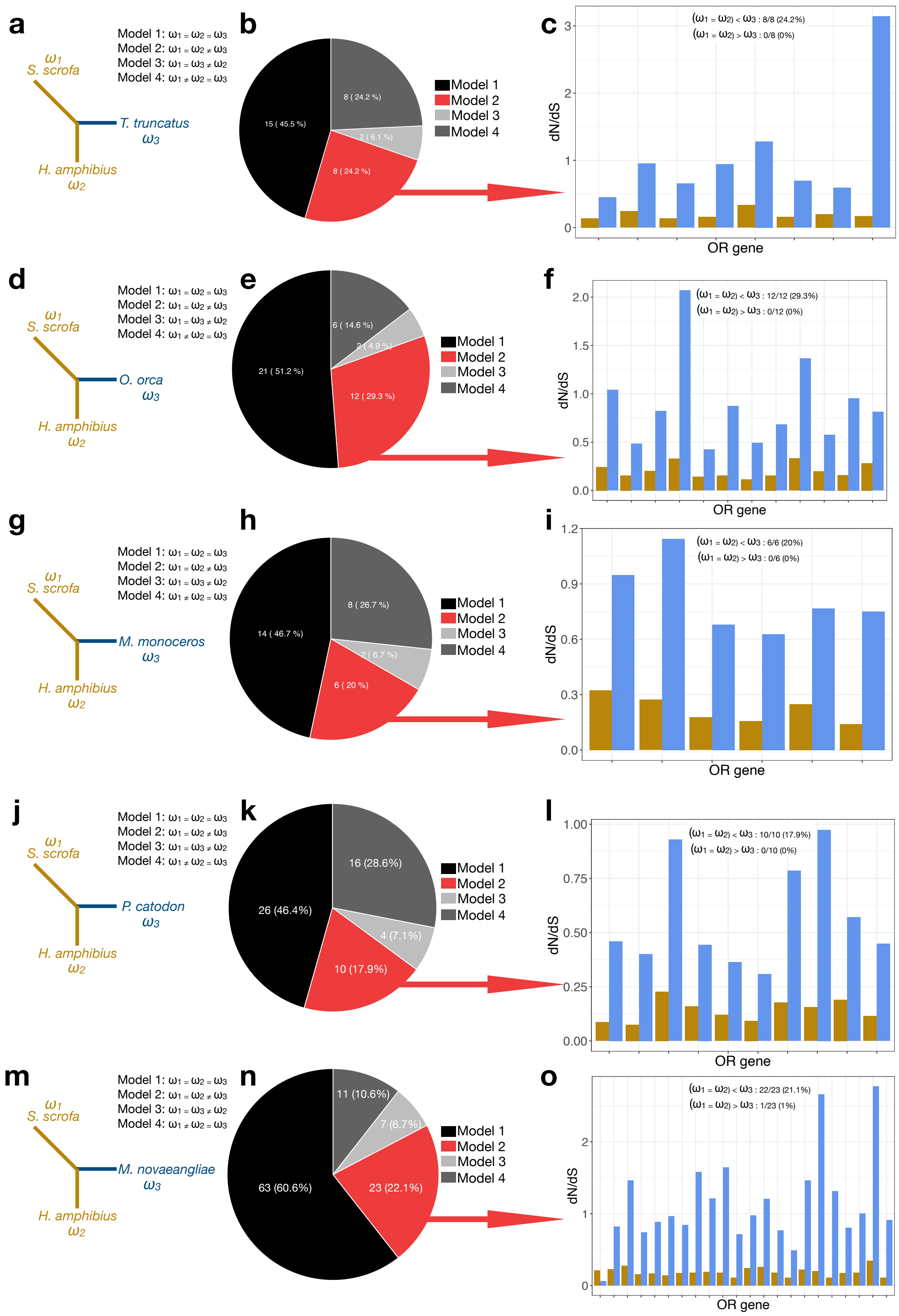

a
Model 1: ω1 = ω2 = ω3
Model 2: ω1 = ω2 ≠ ω3
Model 3: ω1 = ω3 ≠ ω2
Model 4: ω1 ≠ ω2 = ω3
ω1 S. scrofa
T. truncatus ω3
H. amphibius ω2
b
15 (45.5 %)
8 (24.2 %)
2 (6.1 %)
8 (24.2 %)
Model 1
Model 2
Model 3
Model 4
c
(ω1 = ω2) < ω3 : 8/8 (24.2%)
(ω1 = ω2) > ω3 : 0/8 (0%)
dN/dS
OR gene
d
ω1 S. scrofa
O. orca ω3
H. amphibius ω2
e
21 (51.2 %)
6 (14.6 %)
2 (4.9 %)
12 (29.3 %)
f
(ω1 = ω2) < ω3 : 12/12 (29.3%)
(ω1 = ω2) > ω3 : 0/12 (0%)
g
ω1 S. scrofa
M. monoceros ω3
H. amphibius ω2
h
14 (46.7 %)
8 (26.7 %)
2 (6.7 %)
6 (20 %)
i
(ω1 = ω2) < ω3 : 6/6 (20%)
(ω1 = ω2) > ω3 : 0/6 (0%)
j
ω1 S. scrofa
P. catodon ω3
H. amphibius ω2
k
26 (46.4%)
16 (28.6%)
4 (7.1%)
10 (17.9%)
l
(ω1 = ω2) < ω3 : 10/10 (17.9%)
(ω1 = ω2) > ω3 : 0/10 (0%)
m
ω1 S. scrofa
M. novaeangliae ω3
H. amphibius ω2
n
63 (60.6%)
11 (10.6%)
7 (6.7%)
23 (22.1%)
o
(ω1 = ω2) < ω3 : 22/23 (21.1%)
(ω1 = ω2) > ω3 : 1/23 (1%)

Supplementary Fig. 48 | Selection test for *OR* genes in four pinnipeds. Unrooted species tree used for dN/dS (= ω ratio) analysis as well as the different models tested. Model 5 allowing a different ω ratio per branch is not shown as it was never significantly better than the other models. The brown branches correspond to terrestrial species outgroups (*Ursus arctos* and *Spilogale interrupta*) and the blue branch corresponds to the pinnipeds tested: *Mirounga angustirostris* (**a**), *Phoca vitulina* (**d**), *Zalophus californianus* (**g**), *Odobenus rosmarus divergens* (**j**). The number and proportion of *OR* genes supported by each model is shown as pie chart for *Mirounga angustirostris* (**b**), *Phoca vitulina* (**e**), *Zalophus californianus* (**h**), *Odobenus rosmarus divergens* (**k**). For each *OR* gene best supported by model 2, a bar plot shows the ω ratio of the terrestrial branches (brown, $\omega_1 = \omega_2$) and the ω ratio of the pinnipeds species (blue, ω_3): *Mirounga angustirostris* (**c**), *Phoca vitulina* (**f**), *Zalophus californianus* (**i**), *Odobenus rosmarus divergens* (**l**). The number of *OR* genes best supported by model 2 and showing accelerated $((\omega_1 = \omega_2) < \omega_3)$ or decelerated evolution $((\omega_1 = \omega_2) > \omega_3)$ is reported at the top of the bar plots, their percentage relative to the total number of *OR* genes is provided in parentheses. Source data are provided as a Source Data file.

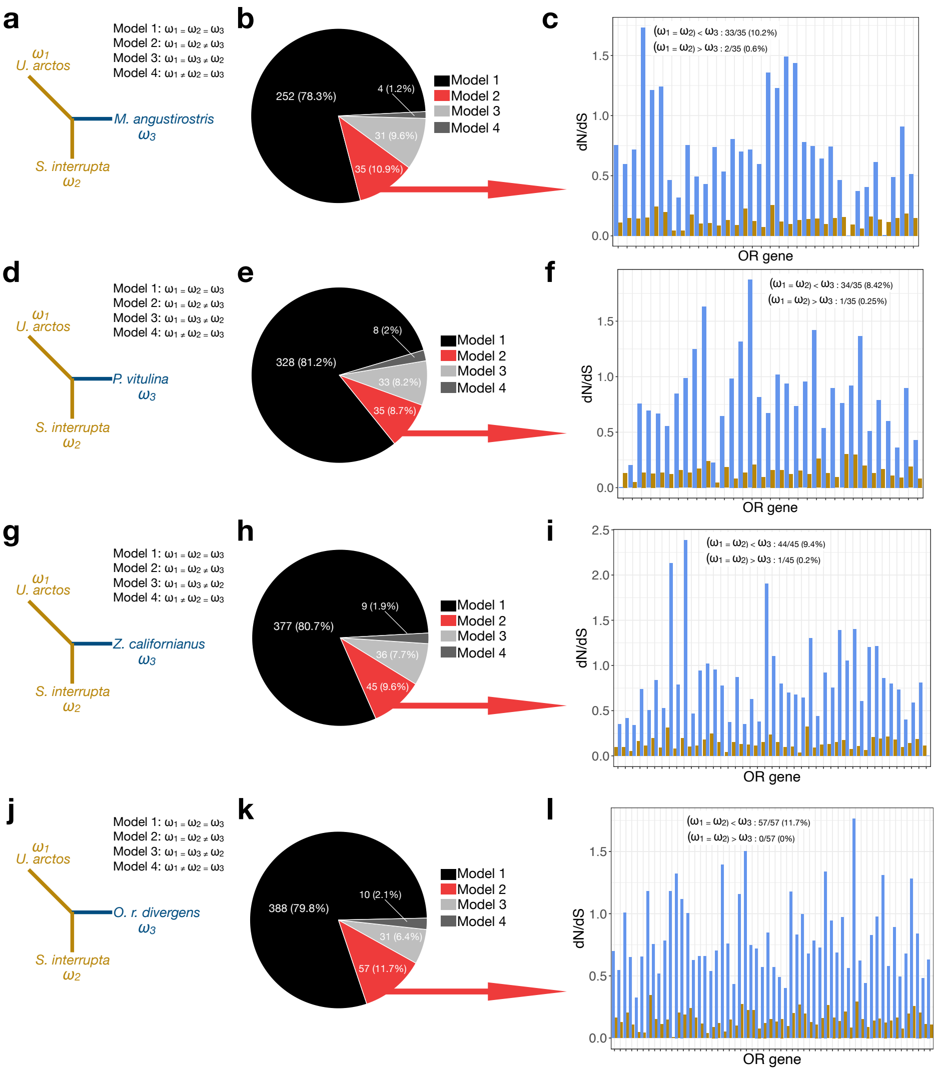

Supplementary Fig. 49 | Selection test for *OR* genes in two sirenian species. Unrooted species tree used for dN/dS (= ω ratio) analysis as well as the different models tested. Model 5 allowing a different ω ratio per branch is not shown as it was never significantly better than the other models. The brown branches correspond to terrestrial species outgroups (*Chrysochloris asiatica* and *Procavia capensis*) and the blue branch corresponds to the sirenian species tested: *Dugong dugon* (**a**), *Trichechus manatus latirostris* (**d**). The number and proportion of *OR* genes supported by each model is shown as pie chart for *Dugong dugon* (**b**), *Trichechus manatus latirostris* (**e**). For each *OR* gene best supported by model 2, a bar plot shows the ω ratio of the terrestrial branches (brown, $\omega_{1=}$ ω_2) and the ω ratio of the sirenian species (blue, ω_3): *Dugong dugon* (**c**), *Trichechus manatus latirostris* (**f**). The number of *OR* genes best supported by model 2 and showing accelerated $((\omega_1 = \omega_2) < \omega_3)$ or decelerated evolution $((\omega_1 = \omega_2) > \omega_3)$ is reported at the top of the bar plots, their percentage relative to the total number of *OR* genes is provided in parentheses. Source data are provided as a Source Data file.

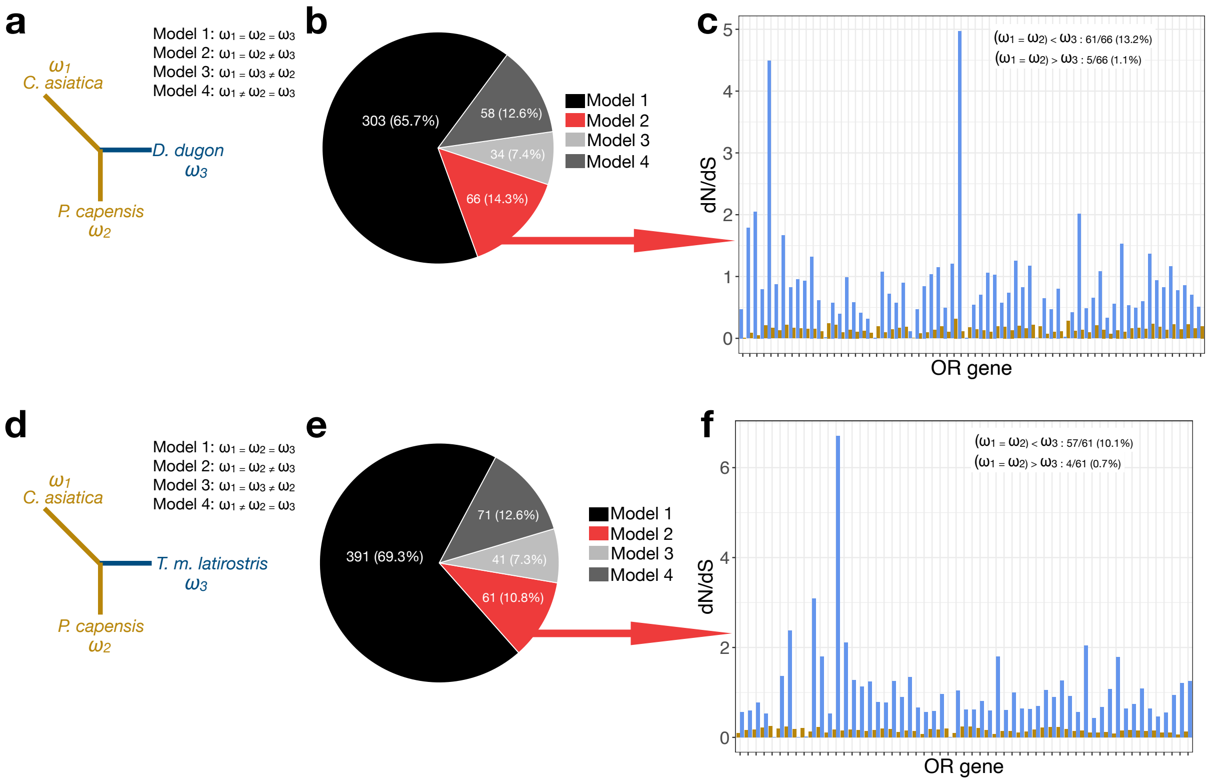

Supplementary Fig. 50 | Selection test for *OR* genes in four *sphenisciformes*. Unrooted species tree used for dN/dS (= ω ratio) analysis as well as the different models tested. Model 5 allowing a different ω ratio per branch is not shown as it was never significantly better than the other models. The brown branches correspond to terrestrial species outgroups (*Theristicus caerulescens* and *Nycticorax nycticorax*) and the blue branch corresponds to the *sphenisciformes* tested: *Pygoscelis adeliae* (**a**), *Eudyptula minor* (**d**), *Megadyptes antipodes antipodes* (**g**), *Eudyptes robustus* (**j**). The number and proportion of *OR* genes supported by each model is shown as pie chart for *Pygoscelis adeliae* (**b**), *Eudyptula minor* (**e**), *Megadyptes antipodes antipodes* (**h**), *Eudyptes robustus* (**k**). For each *OR* gene best supported by model 2, a bar plot shows the ω ratio of the terrestrial branches (brown, $\omega_1 = \omega_2$) and the ω ratio of the sphenisciformes species (blue, ω_3): *Pygoscelis adeliae* (**c**), *Eudyptula minor* (**f**), *Megadyptes antipodes antipodes* (**i**), *Eudyptes robustus* (**l**). The number of *OR* genes best supported by model 2 and showing accelerated $((\omega_1 = \omega_2) < \omega_3)$ or decelerated evolution $((\omega_1 = \omega_2) > \omega_3)$ is reported at the top of the bar plots, their percentage relative to the total number of *OR* genes is provided in parentheses. Source data are provided as a Source Data file.

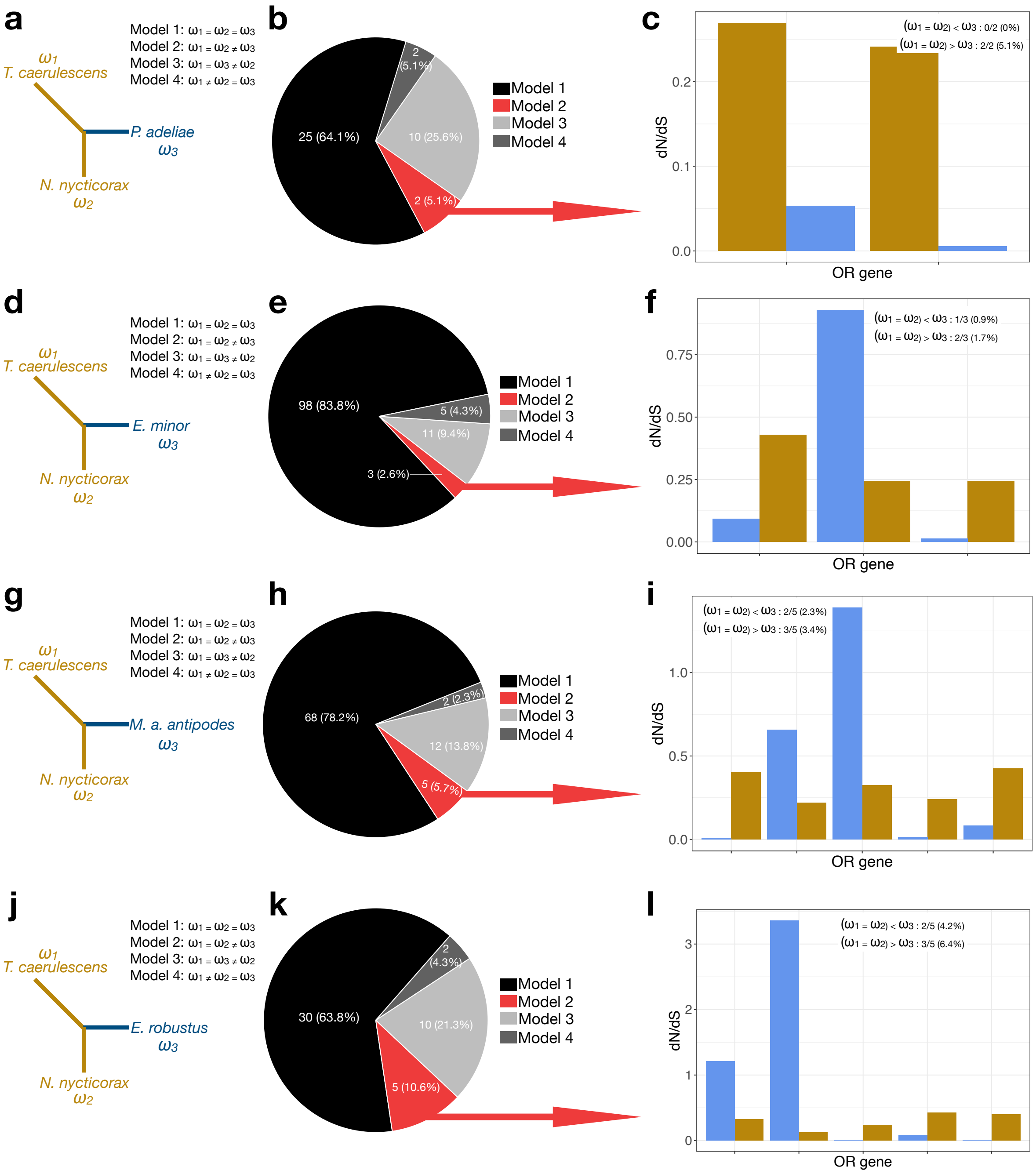
a
Model 1: ω1 = ω2 = ω3
Model 2: ω1 = ω2 ≠ ω3
Model 3: ω1 = ω3 ≠ ω2
Model 4: ω1 ≠ ω2 = ω3
ω1
T. caerulescens
P. adeliae
ω3
N. nycticorax
ω2
b
25 (64.1%)
2 (5.1%)
10 (25.6%)
2 (5.1%)
Model 1
Model 2
Model 3
Model 4
c
(ω1 = ω2) < ω3 : 0/2 (0%)
(ω1 = ω2) > ω3 : 2/2 (5.1%)
dN/dS
OR gene
d
Model 1: ω1 = ω2 = ω3
Model 2: ω1 = ω2 ≠ ω3
Model 3: ω1 = ω3 ≠ ω2
Model 4: ω1 ≠ ω2 = ω3
ω1
T. caerulescens
E. minor
ω3
N. nycticorax
ω2
e
98 (83.8%)
5 (4.3%)
11 (9.4%)
3 (2.6%)
Model 1
Model 2
Model 3
Model 4
f
(ω1 = ω2) < ω3 : 1/3 (0.9%)
(ω1 = ω2) > ω3 : 2/3 (1.7%)
dN/dS
OR gene
g
Model 1: ω1 = ω2 = ω3
Model 2: ω1 = ω2 ≠ ω3
Model 3: ω1 = ω3 ≠ ω2
Model 4: ω1 ≠ ω2 = ω3
ω1
T. caerulescens
M. a. antipodes
ω3
N. nycticorax
ω2
h
68 (78.2%)
2 (2.3%)
12 (13.8%)
5 (5.7%)
Model 1
Model 2
Model 3
Model 4
i
(ω1 = ω2) < ω3 : 2/5 (2.3%)
(ω1 = ω2) > ω3 : 3/5 (3.4%)
dN/dS
OR gene
j
Model 1: ω1 = ω2 = ω3
Model 2: ω1 = ω2 ≠ ω3
Model 3: ω1 = ω3 ≠ ω2
Model 4: ω1 ≠ ω2 = ω3
ω1
T. caerulescens
E. robustus
ω3
N. nycticorax
ω2
k
30 (63.8%)
2 (4.3%)
10 (21.3%)
5 (10.6%)
Model 1
Model 2
Model 3
Model 4
l
(ω1 = ω2) < ω3 : 2/5 (4.2%)
(ω1 = ω2) > ω3 : 3/5 (6.4%)
dN/dS
OR gene

Supplementary Fig. 51 | Selection test for *OR* genes in three marine snakes. Unrooted species tree used for dN/dS (= ω ratio) analysis as well as the different models tested. Model 5 allowing a different ω ratio per branch is not shown as it was never significantly better than the other models. The brown branches correspond to terrestrial species outgroups (*Notechis scutatus* or *Pseudonaja textilis* and *Pseudonaja textilis* or *Bungarus multicinctus*) and the blue branch corresponds to the marine snake tested: *Hydrophis cyanocinctus* (**a**), *Hydrophis curtus* (**d**), *Laticauda colubrina* (**g**). The number and proportion of *OR* genes supported by each model is shown as pie chart for *Hydrophis cyanocinctus* (**b**), *Hydrophis curtus* (**e**), *Laticauda colubrina* (**h**). For each *OR* gene best supported by model 2, a bar plot shows the ω ratio of the terrestrial branches (brown, $\omega_1 = \omega_2$) and the ω ratio of the marine snake species (blue, ω_3): *Hydrophis cyanocinctus* (**c**), *Hydrophis curtus* (**f**), *Laticauda colubrina* (**i**). The number of *OR* genes best supported by model 2 and showing accelerated ($(\omega_1 = \omega_2) < \omega_3$) or decelerated evolution ($(\omega_1 = \omega_2) > \omega_3$) is reported at the top of the bar plots, their percentage relative to the total number of *OR* genes is provided in parentheses. Source data are provided as a Source Data file.

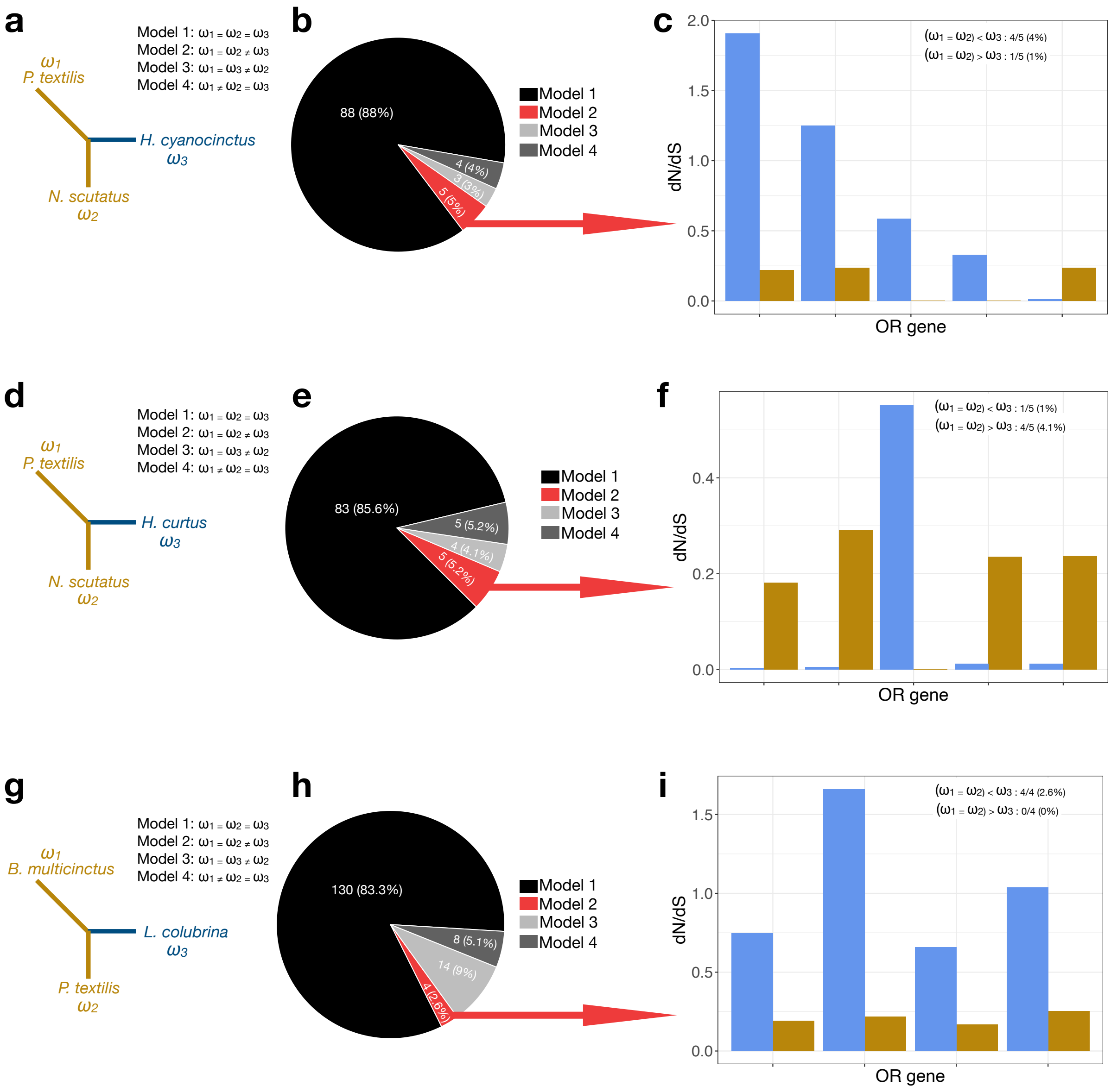

Supplementary Fig. 52 | Selection test for *OR* genes in three marine turtles. Unrooted species tree used for dN/dS (= ω ratio) analysis as well as the different models tested. Model 5 allowing a different ω ratio per branch is not shown as it was never significantly better than the other models. The brown branches correspond to terrestrial species outgroups (*Dermatemys mawii* and *Gopherus evgoodei*) and the blue branch corresponds to the marine turtle tested: *Dermochelys coriacea* (**a**), *Caretta caretta* (**d**), *Chelonia mydas* (**g**). The number and proportion of *OR* genes supported by each model is shown as pie chart for *Dermochelys coriacea* (**b**), *Caretta caretta* (**e**), *Chelonia mydas* (**h**). For each *OR* gene best supported by model 2, a bar plot shows the ω ratio of the terrestrial branches (brown, $\omega_1 = \omega_2$) and the ω ratio of the marine turtle species (blue, ω_3): *Dermochelys coriacea* (**c**), *Caretta caretta* (**f**), *Chelonia mydas* (**i**). The number of *OR* genes best supported by model 2 and showing accelerated ($(\omega_1 = \omega_2) < \omega_3$) or decelerated evolution ($(\omega_1 = \omega_2) > \omega_3$) is reported at the top of the bar plots, their percentage relative to the total number of *OR* genes is provided in parentheses. Source data are provided as a Source Data file.

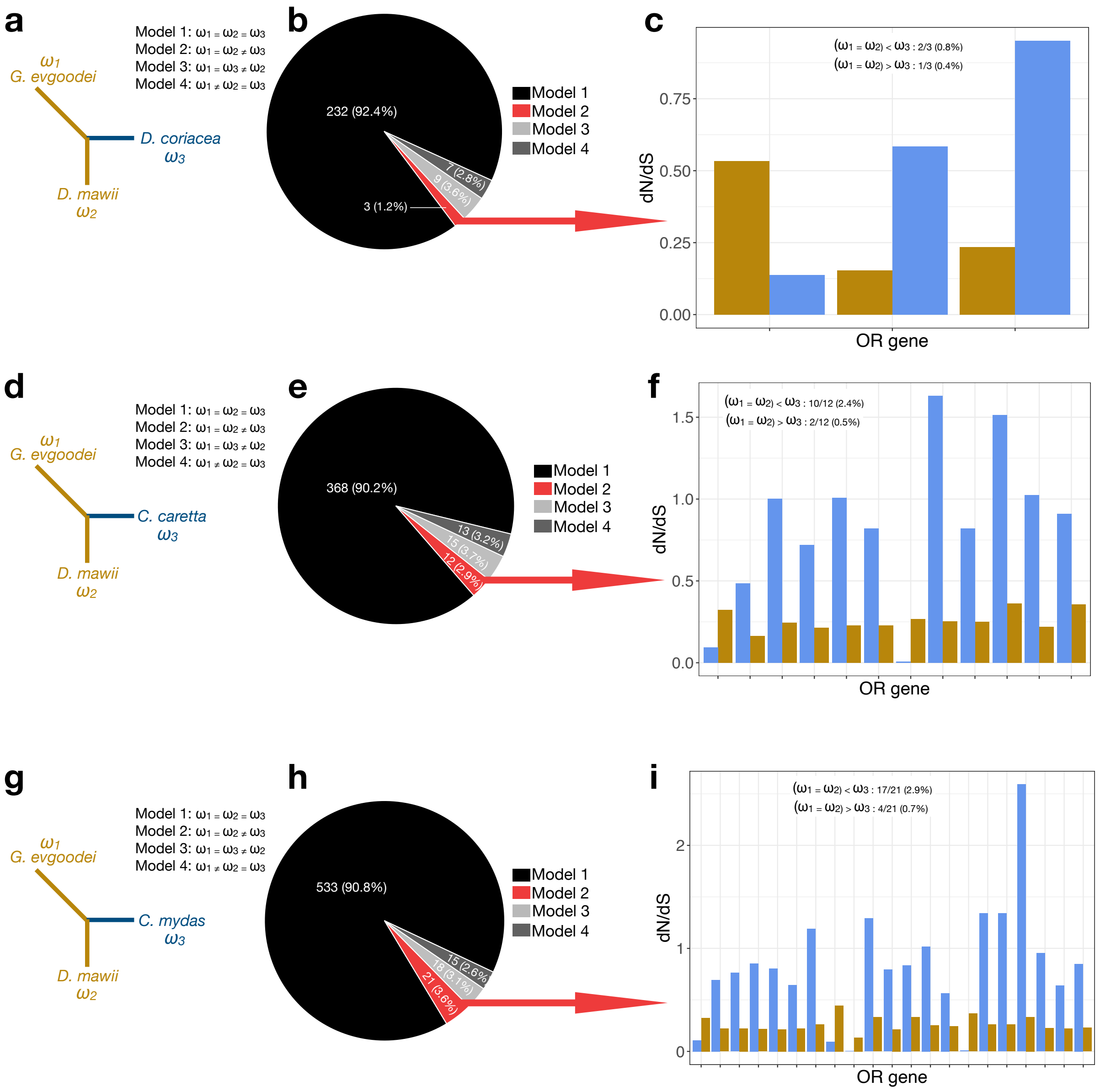

Supplementary Fig. 53 | Correlations between the number of olfactory receptors and the olfactory organ. Two-sided pGLS between the number of complete genes and olfactory organ sizes in mammals (**a**), birds (**b**) and ray-finned fishes (**c**). Some of these correlations were already suggested in previous studies but with much lower sample sizes: N = 19 for the correlation between the number of complete *OR* genes and the relative olfactory bulb size of mammals in[55] (N = 35 for the BUSCO80 and BUSCO90 dataset, and N = 11 for the chromosome-scale assemblies dataset in our study); N = 9 for the number of complete *OR* genes and the olfactory bulb volume of birds in[56] (N = 60, N = 46 and N = 24 for the BUSCO80, BUSCO90 and chromosome-scale assemblies dataset, respectively, in our study); N = 72 for the number of complete *OR*, *V2R* or total number of complete olfactory receptors and the number of lamellae in the olfactory epithelium (OE) of ray-finned fishes in[24] (N = 91, N = 80 and N=57 for the BUSCO80, BUSCO90 and chromosome-scale assemblies dataset, respectively, in our study). Olfactory bulb sizes of birds and mammals, as well as the mean number of lamellae in the OE of ray-finned fishes can be found in Supplementary Data 1. Source data are provided as a Source Data file.

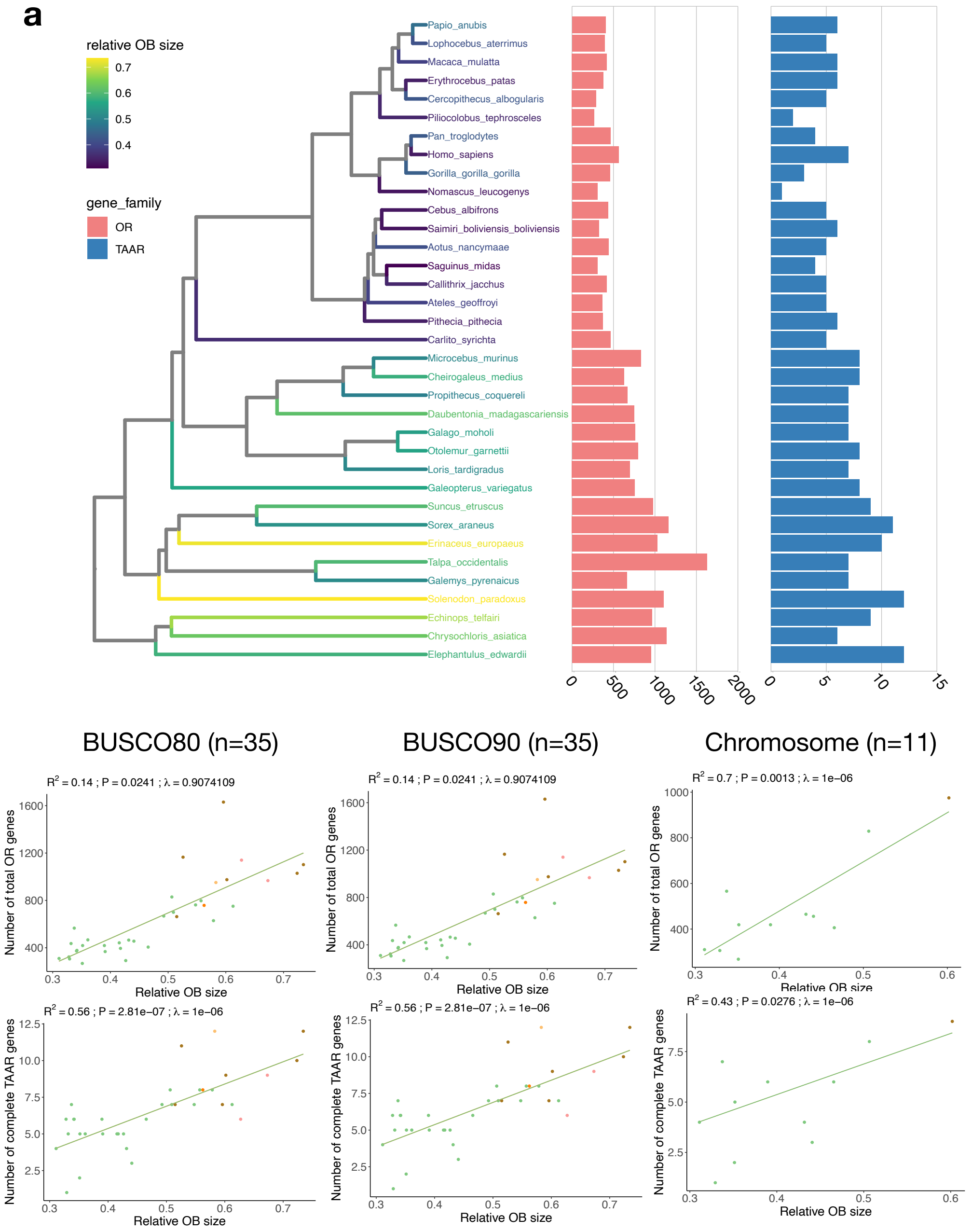
a
relative OB size
0.7
0.6
0.5
0.4
gene_family
OR
TAAR
Papio_anubis
Lophocebus_aterrimus
Macaca_mulatta
Erythrocebus_patas
Cercopithecus_albogularis
Piliocolobus_tephrosceles
Pan_troglodytes
Homo_sapiens
Gorilla_gorilla_gorilla
Nomascus_leucogenys
Cebus_albifrons
Saimiri_boliviensis_boliviensis
Aotus_nancymaae
Saguinus_midas
Callithrix_jacchus
Ateles_geoffroyi
Pithecia_pithecia
Carlito_syrichta
Microcebus_murinus
Cheirogaleus_medius
Propithecus_coquereli
Daubentonia_madagascariensis
Galago_moholi
Otolemur_garnettii
Loris_tardigradus
Galeopterus_variegatus
Suncus_etruscus
Sorex_araneus
Erinaceus_europaeus
Talpa_occidentalis
Galemys_pyrenaicus
Solenodon_paradoxus
Echinops_telfairi
Chrysochloris_asiatica
Elephantulus_edwardii
0
500
1000
1500
2000
0
5
10
15
BUSCO80 (n=35)
BUSCO90 (n=35)
Chromosome (n=11)
R2 = 0.14 ; P = 0.0241 ; λ = 0.9074109
R2 = 0.14 ; P = 0.0241 ; λ = 0.9074109
R2 = 0.7 ; P = 0.0013 ; λ = 1e−06
Number of total OR genes
Relative OB size
R2 = 0.56 ; P = 2.81e−07 ; λ = 1e−06
R2 = 0.56 ; P = 2.81e−07 ; λ = 1e−06
R2 = 0.43 ; P = 0.0276 ; λ = 1e−06
Number of complete TAAR genes
Relative OB size

b

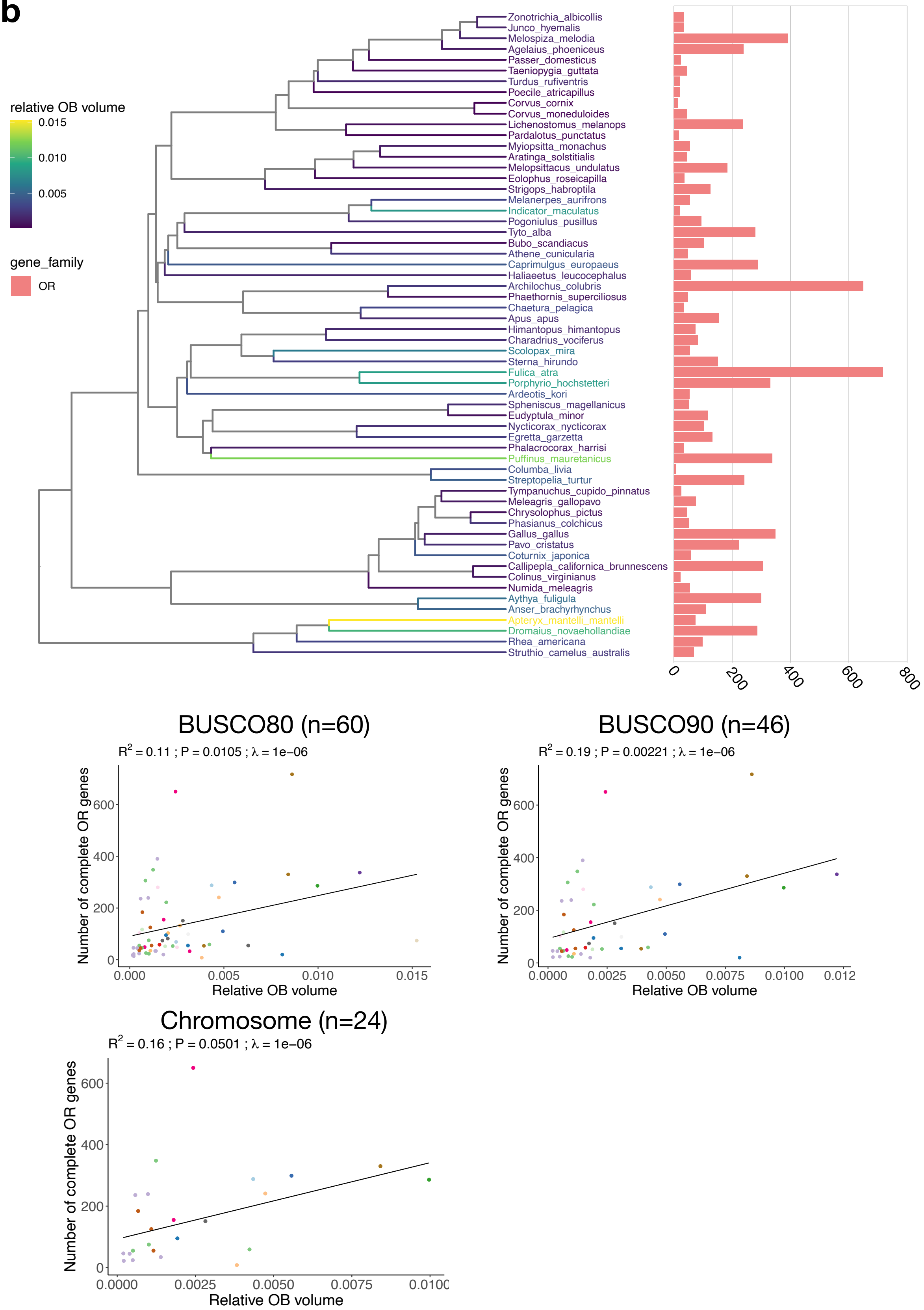
relative OB volume
0.015
0.010
0.005
gene_family
OR
Zonotrichia_albicollis
Junco_hyemalis
Melospiza_melodia
Agelaius_phoeniceus
Passer_domesticus
Taeniopygia_guttata
Turdus_rufiventris
Poecile_atricapillus
Corvus_cornix
Corvus_moneduloides
Lichenostomus_melanops
Pardalotus_punctatus
Myiopsitta_monachus
Aratinga_solstitialis
Melopsittacus_undulatus
Eolophus_roseicapilla
Strigops_habroptila
Melanerpes_aurifrons
Indicator_maculatus
Pogoniulus_pusillus
Tyto_alba
Bubo_scandiacus
Athene_cunicularia
Caprimulgus_europaeus
Haliaeetus_leucocephalus
Archilochus_colubris
Phaethornis_superciliosus
Chaetura_pelagica
Apus_apus
Himantopus_himantopus
Charadrius_vociferus
Scolopax_mira
Sterna_hirundo
Fulica_atra
Porphyrio_hochstetteri
Ardeotis_kori
Spheniscus_magellanicus
Eudyptula_minor
Nycticorax_nycticorax
Egretta_garzetta
Phalacrocorax_harrisi
Puffinus_mauretanicus
Columba_livia
Streptopelia_turtur
Tympanuchus_cupido_pinnatus
Meleagris_gallopavo
Chrysolophus_pictus
Phasianus_colchicus
Gallus_gallus
Pavo_cristatus
Coturnix_japonica
Callipepla_californica_brunnescens
Colinus_virginianus
Numida_meleagris
Aythya_fuligula
Anser_brachyrhynchus
Apteryx_mantelli_mantelli
Dromaius_novaehollandiae
Rhea_americana
Struthio_camelus_australis
0
200
400
600
800
BUSCO80 (n=60)
R² = 0.11 ; P = 0.0105 ; λ = 1e−06
Number of complete OR genes
Relative OB volume
0.000
0.005
0.010
0.015
BUSCO90 (n=46)
R² = 0.19 ; P = 0.00221 ; λ = 1e−06
Number of complete OR genes
Relative OB volume
0.0000
0.0025
0.0050
0.0075
0.0100
0.012
Chromosome (n=24)
R² = 0.16 ; P = 0.0501 ; λ = 1e−06
Number of complete OR genes
Relative OB volume
0.0000
0.0025
0.0050
0.0075
0.0100

c

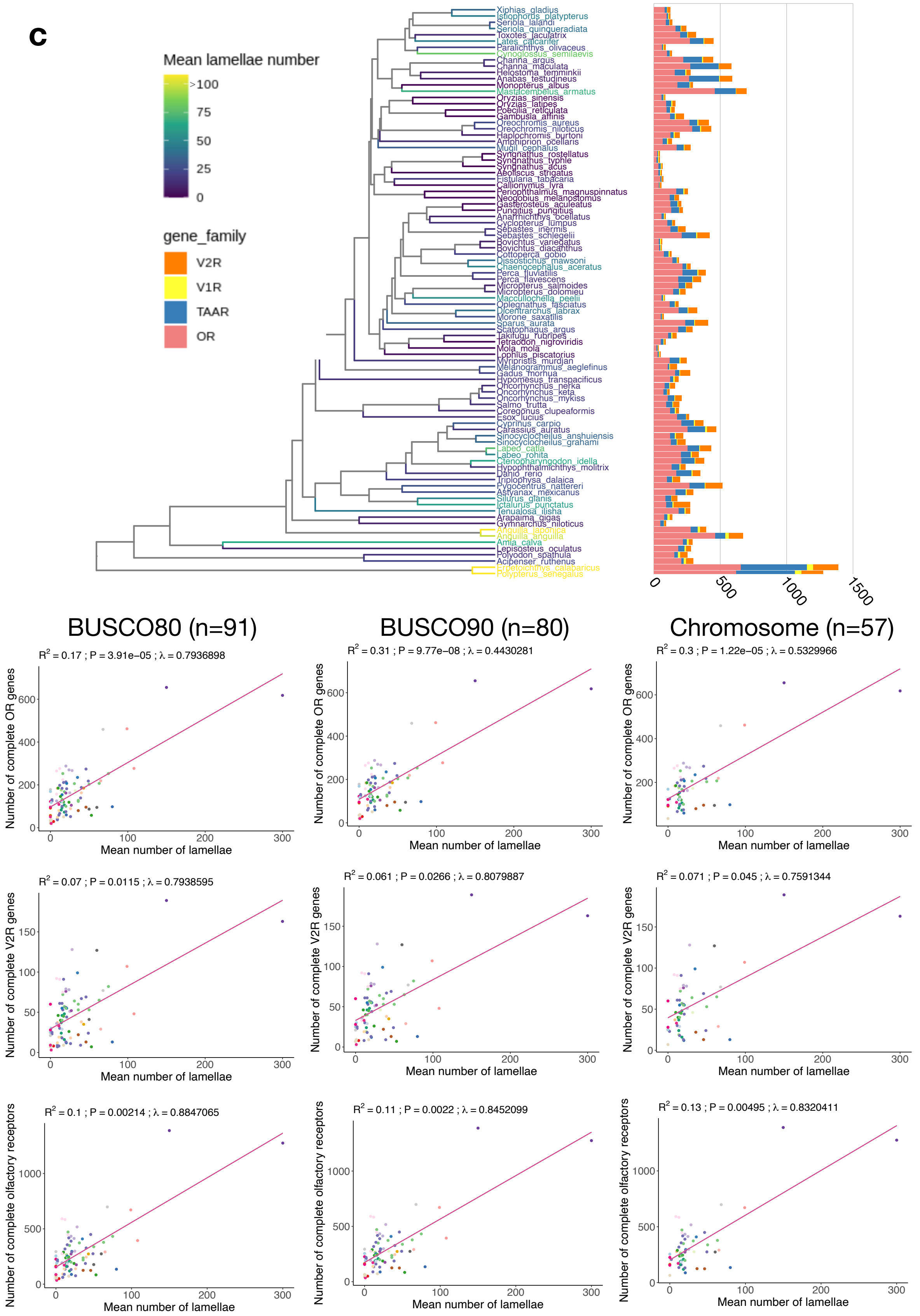

Mean lamellae number
>100
75
50
25
0
gene_family
V2R
V1R
TAAR
OR
Xiphias_gladius
Istiophorus_platypterus
Seriola_lalandi
Seriola_quinqueradiata
Toxotes_jaculatrix
Lates_calcarifer
Paralichthys_olivaceus
Cynoglossus_semilaevis
Channa_argus
Channa_maculata
Helostoma_temminkii
Anabas_testudineus
Monopterus_albus
Mastacembelus_armatus
Oryzias_sinensis
Oryzias_latipes
Poecilia_reticulata
Gambusia_affinis
Oreochromis_aureus
Oreochromis_niloticus
Haplochromis_burtoni
Amphiprion_ocellaris
Mugil_cephalus
Syngnathus_rostellatus
Syngnathus_typhle
Syngnathus_acus
Aeoliscus_strigatus
Fistularia_tabacaria
Callionymus_lyra
Periophthalmus_magnuspinnatus
Neogobius_melanostomus
Gasterosteus_aculeatus
Pungitius_pungitius
Anarrhichthys_ocellatus
Cyclopterus_lumpus
Sebastes_inermis
Sebastes_schlegelii
Bovichtus_variegatus
Bovichtus_diacanthus
Cottoperca_gobio
Dissostichus_mawsoni
Chaenocephalus_aceratus
Perca_fluviatilis
Perca_flavescens
Micropterus_salmoides
Micropterus_dolomieu
Maccullochella_peelii
Oplegnathus_fasciatus
Dicentrarchus_labrax
Morone_saxatilis
Sparus_aurata
Scatophagus_argus
Takifugu_rubripes
Tetraodon_nigroviridis
Mola_mola
Lophius_piscatorius
Myripristis_murdjan
Melanogrammus_aeglefinus
Gadus_morhua
Hypomesus_transpacificus
Oncorhynchus_nerka
Oncorhynchus_keta
Oncorhynchus_mykiss
Salmo_trutta
Coregonus_clupeaformis
Esox_lucius
Cyprinus_carpio
Carassius_auratus
Sinocyclocheilus_anshuiensis
Sinocyclocheilus_grahami
Labeo_catla
Labeo_rohita
Ctenopharyngodon_idella
Hypophthalmichthys_molitrix
Danio_rerio
Triplophysa_dalaica
Pygocentrus_nattereri
Astyanax_mexicanus
Silurus_glanis
Ictalurus_punctatus
Tenualosa_ilisha
Arapaima_gigas
Gymnarchus_niloticus
Anguilla_japonica
Anguilla_anguilla
Amia_calva
Lepisosteus_oculatus
Polyodon_spathula
Acipenser_ruthenus
Erpetoichthys_calabaricus
Polypterus_senegalus
0
500
1000
1500
BUSCO80 (n=91)
BUSCO90 (n=80)
Chromosome (n=57)
R2 = 0.17 ; P = 3.91e−05 ; λ = 0.7936898
R2 = 0.31 ; P = 9.77e−08 ; λ = 0.4430281
R2 = 0.3 ; P = 1.22e−05 ; λ = 0.5329966
Number of complete OR genes
Mean number of lamellae
R2 = 0.07 ; P = 0.0115 ; λ = 0.7938595
R2 = 0.061 ; P = 0.0266 ; λ = 0.8079887
R2 = 0.071 ; P = 0.045 ; λ = 0.7591344
Number of complete V2R genes
Mean number of lamellae
R2 = 0.1 ; P = 0.00214 ; λ = 0.8847065
R2 = 0.11 ; P = 0.0022 ; λ = 0.8452099
R2 = 0.13 ; P = 0.00495 ; λ = 0.8320411
Number of complete olfactory receptors
Mean number of lamellae

Supplementary Fig. 54 | Impact of alternative species tree topologies on the pGLS analysis in mammals. Each pGLS analysis presented in this study was re-computed with 50 alternative species tree topologies and the BUSCO80 dataset for the gene families *TAAR* versus *OR* (a), *V2R* versus *OR* (**b**), *V1R* versus *OR* (**c**), *V2R* versus *TAAR* (**d**), *V1R* versus *TAAR* (**e**), *V1R* versus *V2R* (**f**), OLR versus TR (**g**), *T2R* versus diet (**h**), *OR* versus olfactory bulb (OB) size (**i**), *TAAR* versus OB size (**j**), *OR* versus habitat (**k**), and *TAAR* versus habitat (**l**). The alternative species trees were computed based on 20 concatenated BUSCO genes, and using three calibrations points retrieved on TimeTree.org (Supplementary Data 1). Each plot represents the pGLS R^2 (y-axis) with these 50 alternative trees depending on their Robison-Foulds distance to the reference tree used in the study (x-axis). Red dots correspond to a significant P_{PGLS} (<0.05), whereas blue dots correspond to non-significant P_{PGLS}-values (≥0.05). The horizontal lines correspond to the pGLS R^2 obtained with the reference tree. Above each plot, the number of significant P_{PGLS}-values is reported. Whenever the reference topology yielded a significant P_{PGLS}-value, alternative topologies were also significant and had a very similar R^2, except when comparing the number of complete *OR* genes with OB size (10/50 alternative topologies were significant). For pGLS with a categorical predictor (**h**, **k**, **l**), only the P_{PGLS}-values based on the Robinson-Foulds distance are reported. Source data are provided as a Source Data file.

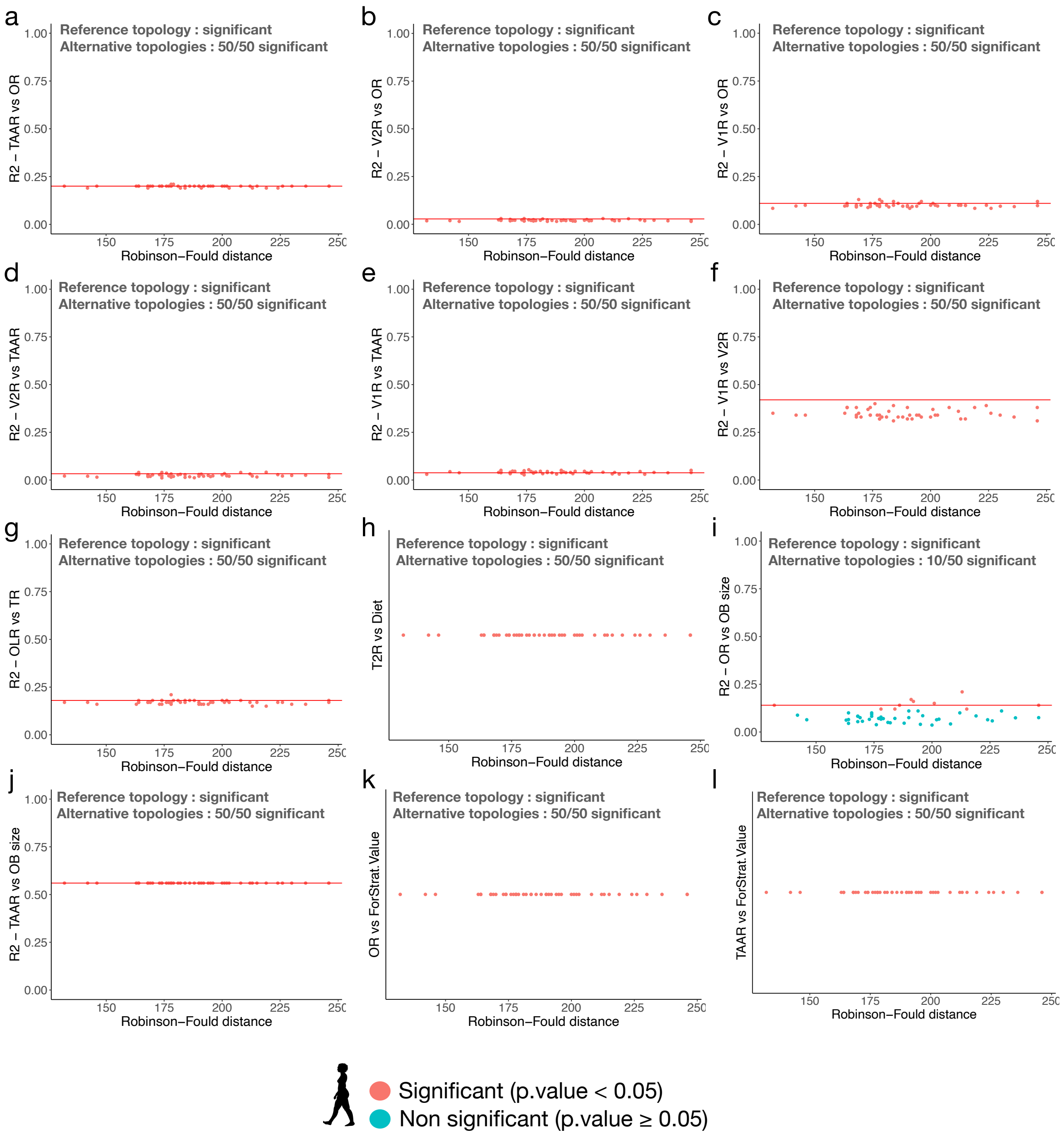

Supplementary Fig. 55 | Impact of alternative species tree topologies on the pGLS analysis in birds. Each pGLS analysis presented in this study was re-computed with 50 alternative species tree topologies and the BUSCO80 dataset for *TAAR* versus *OR* (**a**), OLR versus TR (**b**), *T2R* versus diet (**c**), *OR* versus olfactory bulb (OB) size (**d**), *OR* versus migration (**e**), *T2R* versus migration (**f**), *OR* versus habitat (**g**), and *OR* versus primary lifestyle (**h**). The alternative species trees were computed based on 20 concatenated BUSCO genes, and using three calibrations points retrieved on TimeTree.org (Supplementary Data 1). Each plot represents the pGLS R^2 (y-axis) with these 50 alternative trees depending on their Robinson-Foulds distance to the reference tree used in the study (x-axis). Red dots correspond to a significant P_{PGLS} (<0.05), whereas blue dots correspond to non-significant P_{PGLS}-values (≥0.05). The horizontal lines correspond to the pGLS R^2 obtained with the reference tree. Above each plot, the number of significant P_{PGLS}-values is reported. Whenever the reference topology yielded a significant P_{PGLS}-value, alternative topologies were also significant and had a very similar R^2, except when comparing the number of complete *T2R* genes with migratory behavior (19/50 alternative topologies were significant) and the number of complete *OR* genes with habitat (10/50 alternative topologies were significant). Similarly, when the reference topology yielded a non-significant P_{PGLS} (**c**), only one out of fifty alternative topologies was significant. For pGLS with a categorical predictor (**c**, **e**, **f**, **g**, **h**), only the P_{PGLS}-values based on the Robinson-Foulds distance are reported. Source data are provided as a Source Data file.

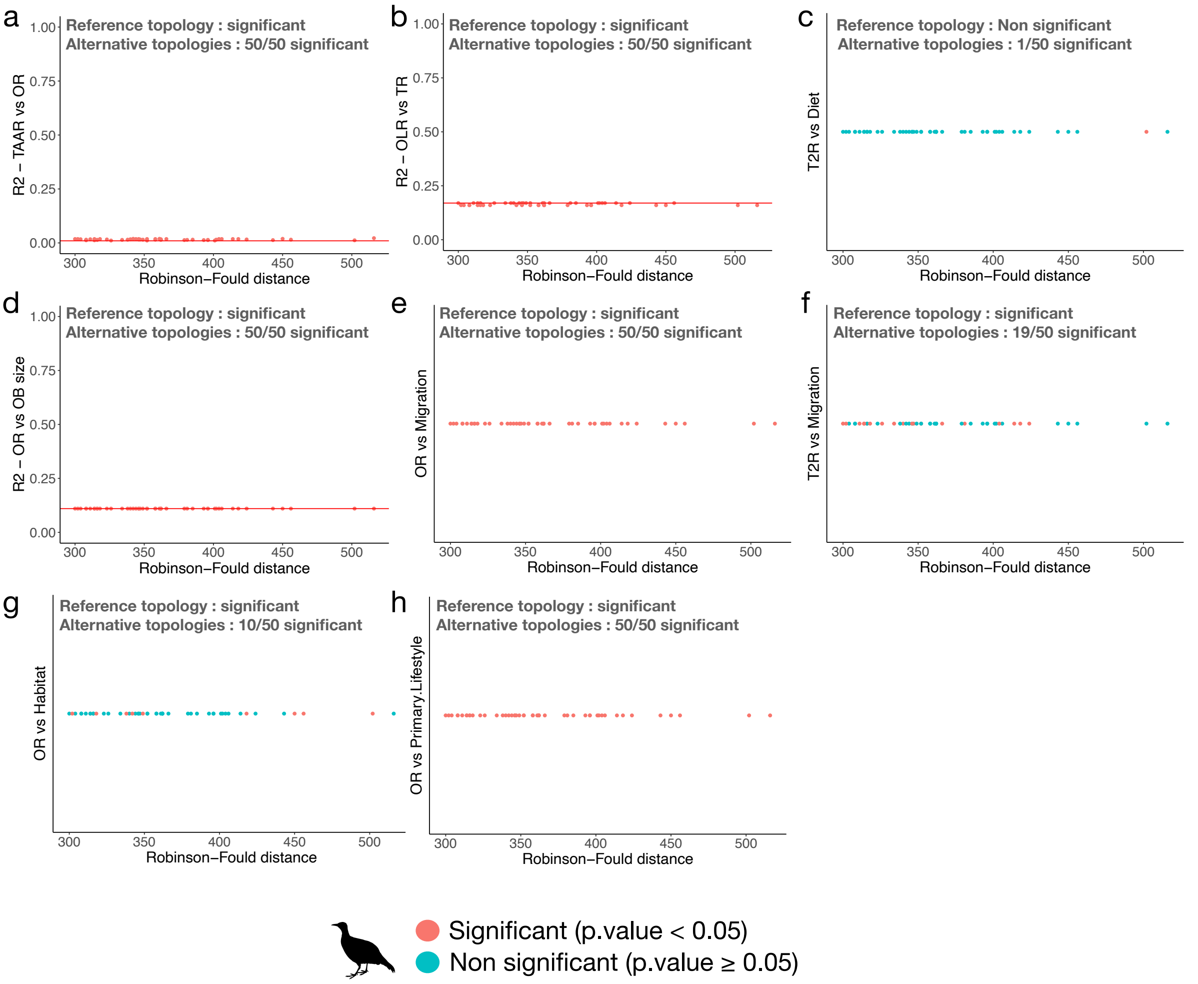

Supplementary Fig. 56 | Impact of alternative species tree topologies on the pGLS analysis in ray-finned fish. Each pGLS analysis presented in this study was re-computed with 50 alternative species tree topologies and the BUSCO80 dataset for *TAAR* versus *OR* (**a**), *V2R* versus *OR* (**b**), *V1R* versus *OR* (**c**), *V2R* versus *TAAR* (**d**), *V1R* versus *TAAR* (**e**), *V1R* versus *V2R* (**f**), *T1R* versus *T2R* (**g**), OLR versus TR (h), *T2R* versus diet (**i**), *OR* versus number of lamellae in the olfactory epithelium (**j**), *V2R* versus number of lamellae (**k**), OLR versus number of lamellae (**l**), and *T1R* versus habitat (**m**). The alternative species trees were computed based on 20 concatenated BUSCO genes, and using three calibrations points retrieved on TimeTree.org (Supplementary Data 1). Each plot represents the pGLS R^2 (y-axis) with these 50 alternative trees depending on their Robinson-Foulds distance to the reference tree used in the study (x-axis). Red dots correspond to a significant P_{PGLS} (<0.05), whereas blue dots correspond to non-significant P_{PGLS}-values (≥0.05). The horizontal lines correspond to the pGLS R^2 obtained with the reference tree. Above each plot, the number of significant P_{PGLS}-values is reported. Whenever the reference topology yielded a significant P_{PGLS}-value, alternative topologies were also significant and had a very similar R^2. For pGLS with a categorical predictor (**i**, **m**), only the P_{PGLS}-values based on the Robinson-Foulds distance are reported. Source data are provided as a Source Data file.

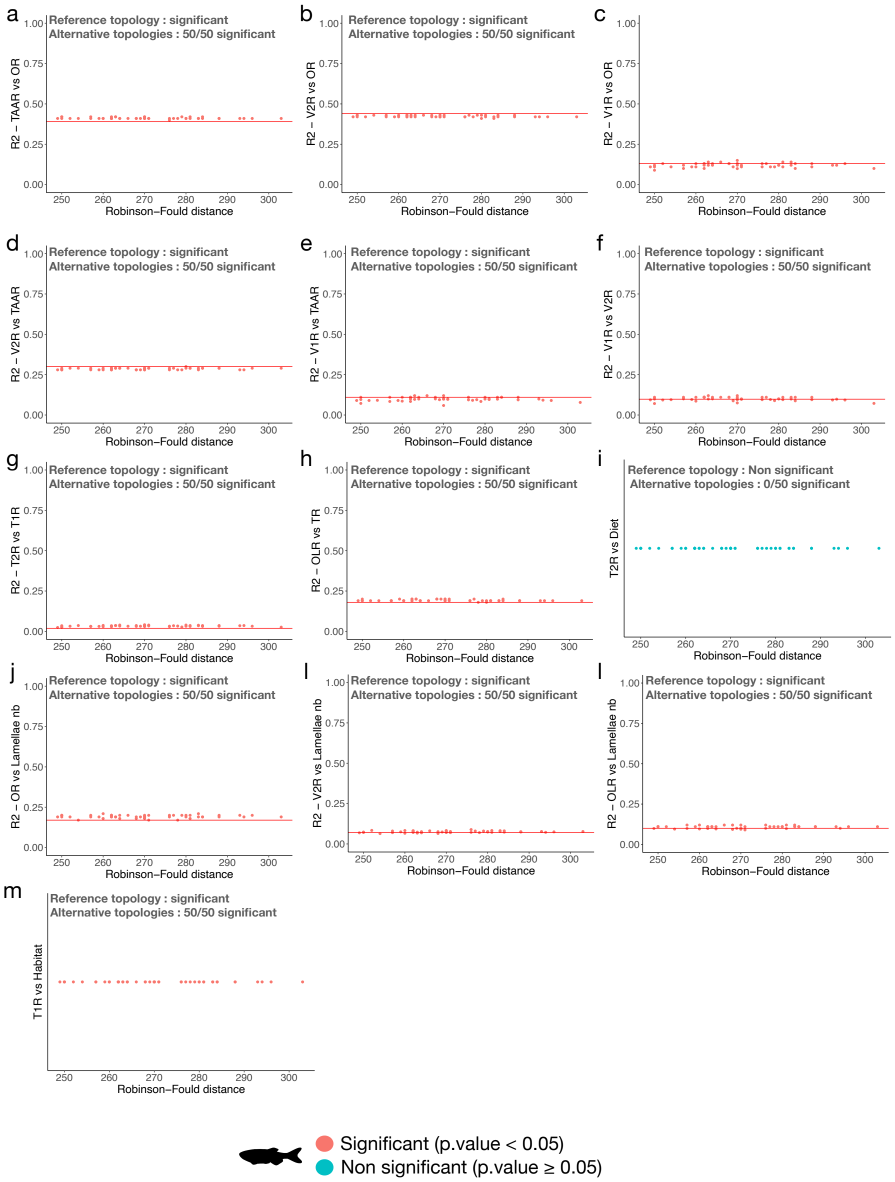

Supplement: Supplementary file 1 — Supplementary Information [file 41467_2024_45500_MOESM1_ESM.pdf]
