# Supplementary material for: Diversity and evolution of the vertebrate chemoreceptor gene repertoire
Source: Nat Commun. 2024 Feb 15;15:1421. doi: 10.1038/s41467-024-45500-y (PMC10869828; doi:10.1038/s41467-024-45500-y)
Supplement: Supplementary file 2 — Description of Additional Supplementary Files [file 41467_2024_45500_MOESM2_ESM.pdf]

Description of Additional Supplementary Files

File Name: Supplementary Data 1
Description: Data used in this manuscript. a, BUSCO raw results for 2,210 vertebrate genome assemblies; b, Number of chemoreceptors per species; c, Species name changes performed in reference species trees; d, Outgroup sequences used for each chemoreceptor phylogenetic tree; e, Number of chemoreceptors retrieved in previous studies and in this study; f, Phylopic link for animal silhouettes used in this manuscript; g, Absence or presence of an accessory olfactory bulb in bats; h, Diet preference of vertebrates; i, Olfactory bulb size of mammals; j, Olfactory bulb size of birds; k, Mean number of lamellae in the olfactory epithelium of ray-finned fishes; l, Manual verification and re-classification of T1R genes in mammals; m, Metadata of ecological data used in this manuscript for mammals, birds and rayfinned fishes; n, pGLS results between chemoreceptors and ecological data; o, Ecological data for ray-finned fishes; p, Ecological data for birds; q, Ecological data for mammals; r, blastn results and DNA identity between lepidosaur chemoreceptors and amphibian chemoreceptors; s, Calibration points used for mammals, birds and ray-finned fishes species trees generated from fifty random concatenated alignments.
