# Supplementary material for: Diversity and evolution of the vertebrate chemoreceptor gene repertoire
Source: Nat Commun. 2024 Feb 15;15:1421. doi: 10.1038/s41467-024-45500-y (PMC10869828; doi:10.1038/s41467-024-45500-y)
Supplement: Supplementary file 4 — Reporting Summary [file 41467_2024_45500_MOESM4_ESM.pdf]

nature portfolio

Corresponding author(s): Maxime Policarpo
Walter Salzburger

Last updated by author(s): Dec 10, 2023

Reporting Summary

Nature Portfolio wishes to improve the reproducibility of the work that we publish. This form provides structure for consistency and transparency in reporting. For further information on Nature Portfolio policies, see our Editorial Policies and the Editorial Policy Checklist.

Statistics

For all statistical analyses, confirm that the following items are present in the figure legend, table legend, main text, or Methods section.

n/a	Confirmed	
☐	☒	The exact sample size (*n*) for each experimental group/condition, given as a discrete number and unit of measurement
☒	☐	A statement on whether measurements were taken from distinct samples or whether the same sample was measured repeatedly
☐	☒	The statistical test(s) used AND whether they are one- or two-sided<br>*Only common tests should be described solely by name; describe more complex techniques in the Methods section.*
☐	☒	A description of all covariates tested
☐	☒	A description of any assumptions or corrections, such as tests of normality and adjustment for multiple comparisons
☐	☒	A full description of the statistical parameters including central tendency (e.g. means) or other basic estimates (e.g. regression coefficient) AND variation (e.g. standard deviation) or associated estimates of uncertainty (e.g. confidence intervals)
☐	☒	For null hypothesis testing, the test statistic (e.g. *F*, *t*, *r*) with confidence intervals, effect sizes, degrees of freedom and *P* value noted<br>*Give P values as exact values whenever suitable.*
☒	☐	For Bayesian analysis, information on the choice of priors and Markov chain Monte Carlo settings
☒	☐	For hierarchical and complex designs, identification of the appropriate level for tests and full reporting of outcomes
☐	☒	Estimates of effect sizes (e.g. Cohen's *d*, Pearson's *r*), indicating how they were calculated

Our web collection on statistics for biologists contains articles on many of the points above.

Software and code

Policy information about availability of computer code

Data collection	Chemoreceptor gene mining was performed using custom scripts (Bash, Python, R and perl) available on GitHub (https://github.com/MaximePolicarpo/Vertebrate_Chemoreceptors_mining).
Data analysis	All the softwares used for data analysis are described and cited in the Methods section. The following softwares used: genome_updater, R 4.2.0 (R packages : ape v5.0, bio3d, caper, phytools, ggtree, ggplot2, dplyr, tidyverse), Python 3.9.5 (python packages: Dendropy), BUSCO v5.1.2, BLAST 2.12.0, EMBOSS 6.2.0, SAMtools 1.15, MAFFT 7.467, IQ-TREE 2.0, PAL2NAL , EXONERATE, Phobius, TMHMM, FastTree2, PastML, BayesTraits, AMAS, trimAl, Notung 2.9, PAML.

For manuscripts utilizing custom algorithms or software that are central to the research but not yet described in published literature, software must be made available to editors and reviewers. We strongly encourage code deposition in a community repository (e.g. GitHub). See the Nature Portfolio guidelines for submitting code & software for further information.

Data

Policy information about availability of data

All manuscripts must include a data availability statement. This statement should provide the following information, where applicable:

- Accession codes, unique identifiers, or web links for publicly available datasets
- A description of any restrictions on data availability
- For clinical datasets or third party data, please ensure that the statement adheres to our policy

All data and results from this study are available on FigShare (https://doi.org/10.6084/m9.figshare.c.6972150.v1). This includes nucleotide sequences of all chemoreceptor sequences (fasta) and their clades (txt); chemoreceptor alignments (fasta); chemoreceptor phylogenetic trees (newick); all species phylogenetic trees (newick) used in this study; PastML results for mammals diet preferences; fifty random concatenated alignments (fasta); and calibrated species trees (newick) for mammals, birds and ray-finned fishes. External databases and datasets were used in this study: EltonTraits, PanTHERIA, AVONET, Fishbase, MammalDIET.

Research involving human participants, their data, or biological material

Policy information about studies with human participants or human data. See also policy information about sex, gender (identity/presentation), and sexual orientation and race, ethnicity and racism.

Reporting on sex and gender	N/A
Reporting on race, ethnicity, or other socially relevant groupings	N/A
Population characteristics	N/A
Recruitment	N/A
Ethics oversight	N/A

Note that full information on the approval of the study protocol must also be provided in the manuscript.

Field-specific reporting

Please select the one below that is the best fit for your research. If you are not sure, read the appropriate sections before making your selection.

☐ Life sciences ☐ Behavioural & social sciences ☒ Ecological, evolutionary & environmental sciences

For a reference copy of the document with all sections, see nature.com/documents/nr-reporting-summary-flat.pdf

Ecological, evolutionary & environmental sciences study design

All studies must disclose on these points even when the disclosure is negative.

Study description	We developed a novel gene mining approach to extract chemoreceptor genes in more than 2’000 publicly available vertebrate genome assemblies. By computing phylogenetic trees with these chemoreceptors, we reconstruct their evolutionary origin and history. We show that chemoreceptor evolution is highly dynamic in vertebrates and that the diversity of chemoreceptor genes has been shaped by lineage-specific expansions and losses. We further elucidate ecological parameters associated with these chemoreceptor gene family dynamics in three vertebrate (sub)classes (mammals, birds and ray-finned fishes).
Research sample	The analyses were performed using publicly available genome assemblies stored in refseq and GenBank databases.
Sampling strategy	The sampling was exhaustive as we took all the genome assemblies available on genbank and refseq.
Data collection	Maxime Policarpo collected sequences of chemoreceptor genes using custom scripts (Bash, Python, R and perl) available on GitHub (https://github.com/MaximePolicarpo/Vertebrate_Chemoreceptors_mining ; DOI: 10.5281/zenodo.10301608.), which are described in the Methods section.
Timing and spatial scale	All the genome assemblies were downloaded as of 31 July 2022.
Data exclusions	Data exclusions were based on genome assemblies completeness measured with BUSCO v5.1.2. One genome was also excluded because a likely sequence contamination was detected, and this is fully described in the Methods section.
Reproducibility	The data was analysed exclusively by bioinformatics methods and are 100% reproducible.
Randomization	There was no randomization because such a procedure was not relevant to the study.

Blinding | There was no blinding because such a procedure was not relevant to the study.

Did the study involve field work? ☐ Yes ☒ No

Reporting for specific materials, systems and methods

We require information from authors about some types of materials, experimental systems and methods used in many studies. Here, indicate whether each material, system or method listed is relevant to your study. If you are not sure if a list item applies to your research, read the appropriate section before selecting a response.

Materials & experimental systems

n/a	Involved in the study
☒	☐ Antibodies
☒	☐ Eukaryotic cell lines
☒	☐ Palaeontology and archaeology
☒	☐ Animals and other organisms
☒	☐ Clinical data
☒	☐ Dual use research of concern
☒	☐ Plants

Methods

n/a	Involved in the study
☒	☐ ChIP-seq
☒	☐ Flow cytometry
☒	☐ MRI-based neuroimaging

Plants

Seed stocks | *Report on the source of all seed stocks or other plant material used. If applicable, state the seed stock centre and catalogue number. If plant specimens were collected from the field, describe the collection location, date and sampling procedures.*

Novel plant genotypes | *Describe the methods by which all novel plant genotypes were produced. This includes those generated by transgenic approaches, gene editing, chemical/radiation-based mutagenesis and hybridization. For transgenic lines, describe the transformation method, the number of independent lines analyzed and the generation upon which experiments were performed. For gene-edited lines, describe the editor used, the endogenous sequence targeted for editing, the targeting guide RNA sequence (if applicable) and how the editor was applied.*

Authentication | *Describe any authentication procedures for each seed stock used or novel genotype generated. Describe any experiments used to assess the effect of a mutation and, where applicable, how potential secondary effects (e.g. second site T-DNA insertions, mosiacism, off-target gene editing) were examined.*
